# Velamentous cord insertion: results from a rapid review of incidence, risk factors, adverse outcomes and screening

**DOI:** 10.1186/s13643-020-01355-0

**Published:** 2020-06-23

**Authors:** Amy Buchanan-Hughes, Anna Bobrowska, Cristina Visintin, George Attilakos, John Marshall

**Affiliations:** 1 Costello Medical, Cambridge, UK; 2 UK National Screening Committee, London, UK; 3 Institute for Women’s Health, University College London, London, UK; 4 Fetal Medicine Unit, University College London Hospitals NHS Foundation Trust, London, UK

**Keywords:** Velamentous cord insertion, Vasa praevia, Ultrasound, Screening, Abnormal placental cord insertion, Adverse pregnancy outcomes, Obstetrics

## Abstract

**Background:**

Velamentous cord insertion (VCI) is an umbilical cord attachment to the membranes surrounding the placenta instead of the central mass. VCI is strongly associated with vasa praevia (VP), where umbilical vessels lie in close proximity to the internal cervical os. VP leaves the vessels vulnerable to rupture, which can lead to fatal fetal exsanguination. Screening for VP using second-trimester transabdominal sonography (TAS) to detect VCI has been proposed. We conducted a rapid review investigating the quality, quantity and direction of evidence available on the epidemiology, screening test accuracy and post-screening management pathways for VCI.

**Methods:**

MEDLINE, Embase and the Cochrane Library were searched on 5 July 2016 and again on 11 October 2019, using general search terms for VP and VCI. Only peer-reviewed articles reporting on the epidemiology of VCI, the accuracy of the screening test and/or downstream management pathways for VCI pregnancies were included. Quality and risk of bias of each included study were assessed using pre-specified tools.

**Results:**

Forty-one relevant publications were identified; all but one were based on non-UK pregnancy cohorts, and most included relatively few VCI cases. The estimated incidence of VCI was 0.4–11% in singleton pregnancies, with higher incidence in twin pregnancies (1.6–40%). VCI incidence was also increased among pregnancies with one or more other risk factors, including in vitro fertilisation pregnancies or nulliparity. VCI incidence among women without any known risk factors was unclear.

VCI was associated with adverse perinatal outcomes, most notably pre-term birth and emergency caesarean section in singleton pregnancies, and perinatal mortality in twins; however, associations varied across studies and the increased risk was typically low or moderate compared with pregnancies without VCI.

In studies on limited numbers of cases, screening for VCI using TAS had good overall accuracy, driven by high specificity. No studies on post-screening management of VCI were identified.

**Conclusions:**

Literature on VCI epidemiology and outcomes is limited and low-quality. The accuracy of second-trimester TAS and the benefits and harms of screening cannot be determined without prospective studies in large cohorts. Modelling studies may indicate the feasibility and value of studying the epidemiology of VCI and the potential impact of detecting VCI as part of a population screening programme for VP.

## Introduction

The umbilical cord usually runs from the centre of the placenta to the fetus. However, two main types of abnormal cord insertion have been identified [1, 2]: (1) velamentous cord insertion (VCI), where the cord attaches only to the membranes surrounding the placenta rather than the central placental mass, leaving umbilical vessels unprotected by Wharton’s jelly, and (2) marginal cord insertion (MCI), where the cord insertion site is on the periphery of the placental mass and is supported by very little placental tissue. VCI is associated with a relatively increased risk of complications [3] and has been reported to occur in approximately 0.4 to 2.4% of singleton pregnancies and in as many as 40% of twin pregnancies [4–9].

VCI sometimes coincides with vasa praevia (VP), a rare but serious pregnancy condition in which the unprotected umbilical vessels lie in close proximity to the internal cervical os. The vessels are therefore prone to compression and rupture when the surrounding membranes rupture during birth. VP is reported to occur in 0.06% of all UK pregnancies [10]. A high mortality rate associated with undiagnosed VP has been reported due to rapid fetal exsanguination [11–16].

The UK National Screening Committee (UK NSC) regularly reviews the evidence relating to the introduction, modification and cessation of national population screening programmes [17], and VP is one of the conditions of interest. Transabdominal ultrasound screening (TAS) for VP during the second trimester has been proposed to identify a group of women who would be offered a transvaginal ultrasound scan (TVS) to test for VP, followed by a recommendation to give birth by caesarean section (CS) if VP is suspected [18, 19]. Algorithms for the detection of VP vary but tend to focus on the mid-trimester ultrasound scan; a strong association between VP and VCI has led to the proposal that the detection of VCI is included in the panel of universal screening targets at this point of pregnancy [18–23].

Overdetection, defined as the detection of a marker of risk for a condition that would have otherwise not caused any adverse outcomes, is a significant concern in any screening programme. This is because identification and consequent treatment in these cases may cause harm or result in unnecessary use of resources [24]. Likewise, detecting an abnormality or marker of risk for a condition for which there is no intervention available, as a by-product of a screening programme for a target condition, might not be appropriate because it may cause the affected individuals anxiety without offering them any treatment options.

The UK NSC’s previous appraisals of screening for VP did not conclude with a recommendation for implementing VP screening. This was partly due to concern that screening would find pregnancies affected by VCI without VP. Published estimates suggest that although most women with VP will have VCI, only 2% of women with VCI will also have VP. Therefore, most cases of screen-detected VCI would not have VP. Detecting VCI through VP screening would be a departure from the current UK clinical practice, resulting in this condition being routinely detected without management guidelines for it in place [25]. This aspect of screening for VP has received little attention, and if screening for VP is to be considered in its entirety, the implications of detecting VCI should be explored in greater depth.

As a preliminary step towards this, the UK NSC commissioned a rapid review to gauge the volume, quality and direction of the evidence relating to the epidemiology and broad risks associated with VP and VCI, in populations similar to the UK pregnancy cohort. The review also explored the evidence relating to the accuracy of second-trimester ultrasound screening and downstream management pathways for VP and VCI. This paper describes the literature identified on VCI (including studies identified in a subsequent VCI-focused update) and comments on the implications of implementing a screening programme for VP based on detecting VCI, as currently proposed, in the UK. The entirety of the original review, including the results pertaining to VP, is available on the Legacy Screening website [22].

## Methods

### Literature review

The UK NSC has a formal process for assessing the viability, effectiveness and appropriateness of introducing national screening programmes [17]. This process includes the identification and appraisal of evidence relating to a subset of 20 criteria [26]. The process uses rapid review methods with the presentation of results in a narrative summary rather than a statistical meta-analysis.

In line with this process, an a priori protocol was developed, with input from clinical experts, to identify the evidence required to inform the discussion on criterion 1 (regarding the prevalence and severity of the condition), criterion 4 (regarding the reliability of the screening test for the condition) and criteria 9 and 10 (regarding the need for downstream management pathways to be in place for people found to have the condition) (Table 1).

**Table 1:** Key questions for the VP and VCI evidence summary and relationship to UK NSC screening criteria

Criterion		Key questions
1	The condition should be an important health problem as judged by its frequency and/or severity. The epidemiology, incidence, prevalence and natural history of the condition should be understood, including development from latent to declared disease and/or there should be robust evidence about the association between the risk or disease marker and serious or treatable disease.	What is the incidence of VP in the UK? (Q1)
		What percentage of VP cases identified in the second trimester will resolve by late pregnancy? (Q2)
		What is the risk of adverse perinatal outcomes in pregnancies associated with VP? (Q3)
		What is the incidence of VCI in the UK? (Q6)
		What is the risk of adverse perinatal outcomes in pregnancies associated with VCI? (Q7)
4	There should be a simple, safe, precise and validated screening test.	How effective is second-trimester TAS for detecting VP? (Q4)
		How effective is second-trimester TAS for detecting VCI? (Q8)
9 and 10	9: There should be an effective intervention for patients identified through screening, with evidence that intervention at a pre-symptomatic phase leads to better outcomes for the screened individual compared with usual care. Evidence relating to wider benefits of screening, for example those relating to family members, should be taken into account where available. However, where there is no prospect of benefit for the individual screened then the screening programme should not be further considered. 10: There should be agreed evidence-based policies covering which individuals should be offered interventions and the appropriate intervention to be offered.	What is the most effective management pathway for women with screen-detected VP? (Q5)
		What is the most effective management pathway for women with screen-detected VCI? (Q9)

*TAS* transabdominal ultrasound scan, *VCI* velamentous cord insertion, *VP* vasa praevia

MEDLINE, Embase and the Cochrane Library were searched on 5 July 2016, using general search terms for VP and VCI, without date limits (Supplementary Table 1). Reference lists of relevant systematic reviews were hand-searched for additional relevant articles not already identified through the database searches. In order to report on the most up-to-date evidence base, the review was updated with searches run on 11 October 2019, applying a date limit from 2016 onwards. In the update searches, review of titles/abstracts and full texts, data extraction and quality assessments were conducted using the same methodology and eligibility criteria as in the original review. However, given the focus of this report, only the eligibility criteria relating to VCI were applied to the updated searches.

Each abstract was reviewed against the pre-specified eligibility criteria (Supplementary Tables 2, 3, 4) by one reviewer. A second independent reviewer provided input in cases of uncertainty and checked a random 20% of the first reviewer’s screening decisions. The full texts of articles included at the abstract screening stage were also reviewed against the eligibility criteria by one reviewer, who determined whether the article was relevant to one or more of the key review questions (Table 1). Again, a second independent reviewer provided input in cases of uncertainty and validated 20% of the first reviewer’s decisions on the full texts. In both the abstract and full-text review stages, any disagreements between the primary and secondary reviewers were resolved by discussion until a consensus was met. Studies excluded at the full-text review stage during the original review are listed in the report of the original review (Additional file 1), whereas studies excluded at the full-text review during the update are listed in Supplementary Table 5.

Many of the identified studies were conducted in the 10 years before the original search date (i.e. 2006 to 2016). Therefore, as a practical measure for conducting the rapid review, the questions on epidemiology and adverse perinatal outcomes associated with VCI were limited to studies published since 2006. No date limits were applied for studies of screening tests or management pathways.

Data from included studies were extracted into pre-specified extraction tables in Microsoft Word. Study quality was assessed by one reviewer, and this was verified by a second reviewer. Simple calculations (e.g. the number of non-VCI pregnancies with adverse outcomes) as well as calculations of odd ratios (ORs; using the online MedCalc tool [27]) were performed as part of the data extraction process. For publications that reported data on screening tests but did not report test accuracy measures, some measures of test accuracy (sensitivity, specificity, positive predictive value [PPV] and negative predictive value [NPV]) were calculated from information provided in the publication using MedCalc [27]. In line with the UK NSC’s approach to evidence summaries, results were not pooled across studies but were synthesised narratively taking into account the volume, quality, applicability and consistency of the evidence.

A detailed description of the protocol used for this rapid review has been published in an online report of the review [22] (the report has also been provided as Additional file 1).

### Quality and risk of bias assessment

Pre-specified tools used to assess the quality and risk of bias of each study included in the review were the following: JBI Critical Appraisal Checklist for Studies Reporting Prevalence Data for epidemiological studies [28], Centre for Evidence Based Medicine Prognostic Studies Critical Appraisal Worksheet for prognostic studies [29] and Quality Assessment of Diagnostic Accuracy Studies (QUADAS-2) tool for diagnostic accuracy studies [30]. For the question on management pathways, checklists were pre-specified for randomised controlled trials (RCTs) [31], interventional non-RCTs [32], cohort studies [33] and case-control studies [34]; however, no studies of these designs were identified in the rapid review.

When considering outcomes reported as odds ratios (ORs) or other measures of relative risk, an OR from 0.5 to 2 was considered a weak effect, an OR from 0.2 to 0.5 or 2 to 5 was considered a moderate effect and an OR < 0.2 or > 5 was considered a strong effect.

## Results

**Fig. 1:**
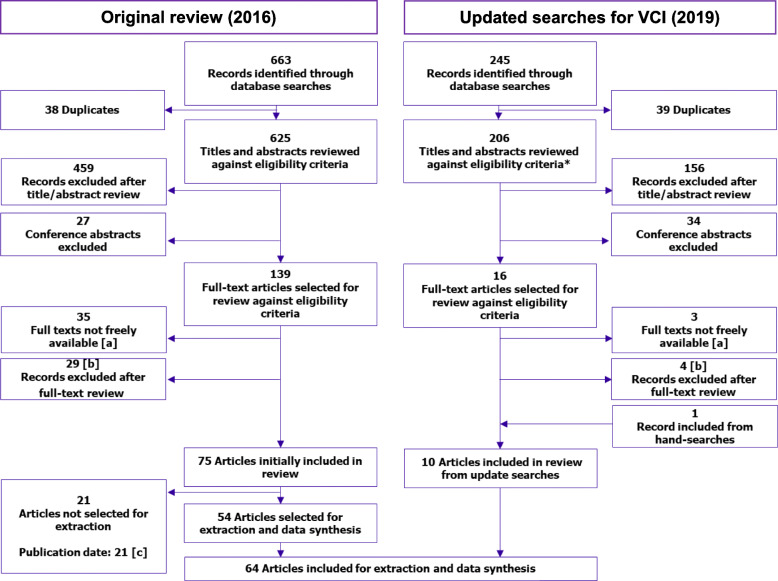
PRISMA flow summary of the rapid review results. *Given the focus of this review, only the eligibility criteria relating to VCI were applied. [**a**] Searches for full-text articles were carried out at Cambridge University Library. Some articles were not freely available at this library. One article (Francois 2003 (ref [10] in Supplementary References)) was included on the basis of the abstract alone, but for the remainder of the articles, it was judged that they would not contain any additional pivotal data from relevant populations that would affect the conclusions of this review. [**b**] Eight articles (original) and two articles (update) were hand-searched, but not included in their own right. [**c**] Two would also have been excluded for country if they were not already excluded for date

Database searches yielded 625 unique results in the original review and 206 in the update, of which 54 (original review) and 9 (update) were included in the evidence synthesis (Fig. 1; the completed PRISMA checklist has been provided [see Additional file 2]). One relevant article was identified through hand-searching the reference lists of relevant systematic reviews during the update of the rapid review, giving a total of 64 records ultimately included.

Of the 64 publications selected for inclusion in the review, 48 reported data relating to the questions about the epidemiology, the adverse outcomes, the screening test and post-detection management pathways for VCI (Table 2). Twenty-seven of 54 publications in the original review reported data for VP (Supplementary Table 6; note that some publications reported relevant information on both VCI and VP). Two systematic reviews on pre-term birth and one on adverse pregnancy outcomes in singleton pregnancies with VCI were identified [35–37] and were hand-searched to identify primary studies meeting our eligibility criteria.

**Table 2:** Summary of publications with relevant VCI data

Study	Study design	Country	Years of study	Criterion 1 – Epidemiology	Criterion 4 – Screening	Criteria 9 and 10 – Management pathways
Baumfeld 2016 [57]	Retrospective	Israel	1988 to 2012	Epidemiology, adverse outcomes	-	-
Bronsteen 2013 [49], Lee 2000 [71]	Retrospective	USA	1990 to 2010	Epidemiology, natural history, adverse outcomes	Test accuracy	[b]
Brouillet 2014 [72]	Retrospective	France	2006	Epidemiology	-	-
Bukowski 2017 [60]	Case-control	USA	2006 to 2008	Adverse outcomes	-	-
Chu 2013 [61]	Retrospective	USA	2010 to 2011	Adverse outcomes	-	-
Cirstoiu 2016 [40]	Retrospective	Romania	2010 to 2016	Epidemiology, adverse outcomes	-	-
Costa-Castro 2013 [42], Lopriore 2007 [73], Lopriore 2012 [74]	Retrospective	Portugal, the Netherlands	2002 to 2012	Epidemiology, adverse outcomes	-	-
Costa-Castro 2016 [44]	Retrospective	Portugal, the Netherlands	2005 to 2015	Epidemiology, adverse outcomes	-	-
Couck 2018 [75]	Retrospective	Belgium	2002 to 2016	Epidemiology	-	-
De Paepe 2010a [45], De Paepe 2010b [76]	Prospective	USA	2001 to 2008	Epidemiology	-	-
De Paepe 2011 [46]	Prospective	USA	2009 to 2011	Epidemiology	-	-
Di Salvo 1998 [63]	Prospective	USA	1992 to 1995	Epidemiology [a]	Test accuracy	-
Ebbing 2013 [4], Ebbing 2015 [1],Ebbing 2017 [3]	Retrospective	Norway	1999 to 2011	Epidemiology, adverse outcomes	-	-
Hack 2008 [58]	Prospective	The Netherlands	1998 to 2007	Epidemiology, adverse outcomes	-	-
Hack 2009 [48]	Prospective	The Netherlands	1998 to 2008	Epidemiology, adverse outcomes	-	-
Hasegawa 2005 [65], Hasegawa 2006 [69]	Prospective, retrospective	Japan	2002 to 2004	Epidemiology, adverse outcomes [a]	Test accuracy	-
Hasegawa 2009a [5], Hasegawa 2009b [77]	Retrospective cohort	Japan	2005 to 2006	Epidemiology, adverse outcomes	-	-
Hasegawa 2011 [39]	Retrospective cohort	Japan	2006 to 2009	Epidemiology	-	-
Heller 2014 [50]	Retrospective	USA	2007 to 2011	Epidemiology, adverse outcomes	-	-
Ismail 2017 [62]	Prospective	Ireland	2016	Epidemiology, adverse outcomes	-	-
Kalafat 2018 [38]	Retrospective	UK	2000 to 2016	Epidemiology	-	-
Kanda 2011 [51]	Retrospective	Japan	2002 to 2007	Epidemiology, adverse outcomes	Test accuracy	[b]
Kent 2011 [47]	Prospective	Ireland	2007 to 2009	Epidemiology, adverse outcomes	-	-
Lepais 2014 [78]	Retrospective	France	2005 to 2009	Epidemiology	-	-
McNamara 2014 [6]	Retrospective	Canada	1978 to 2007	Epidemiology, adverse outcomes	-	-
Melcer 2017 [55]	Retrospective	Israel	2005 to 2016	Epidemiology	-	-
Nomiyama 1998 [66]	Prospective	Japan	1993 to 1996	Epidemiology [a]	Test accuracy	-
Pinar 2014 [59]	Case-control	USA	2006 to 2008	Epidemiology	-	-
Pretorius 1996 [16]	Prospective	USA	1992 to 1993	Epidemiology [a]	Test accuracy	-
Raisanen 2012 [7]	Retrospective	Finland	2000 to 2011	Epidemiology, adverse outcomes	-	-
Rebarber 2014 [52]	Retrospective	USA	2005 to 2012	Epidemiology, natural history, adverse outcomes	-	[b]
Sepulveda 2006 [67]	Prospective	Chile	NR	Epidemiology [a]	Test accuracy	-
Sepulveda 2003 [68]	Prospective	Chile	2001 to 2002	Epidemiology [a]	Test accuracy	-
Smorgick 2010 [53]	Retrospective	Israel	1998 to 2007	Epidemiology, adverse outcomes	-	-
Suzuki 2015 [8]	Prospective	Japan	2002 to 2011	Epidemiology, adverse outcomes	-	-
Swank 2016 [54]	Retrospective	USA	2000 to 2012	Epidemiology, natural history, adverse outcomes	-	-
Walker 2012 [79]	Retrospective	Canada	2000 to 2010	Epidemiology	-	-
Waszak 2016 [43]	NR	Poland	NR	Epidemiology, adverse outcomes	-	-
Yanaihara 2018 [41]	NR	Japan	2015 to 2017	Epidemiology	-	-
Yerlikaya 2016 [9]	Case-control	Austria	2003 to 2013	Epidemiology, adverse outcomes	-	-

[a] Studies were completed before 2000 so epidemiology data were not extracted, but other relevant data from the study were extracted. [b] Management pathways reported descriptively but not analytically; *NR* not reported

### Epidemiology

The rapid review identified 37 publications reporting the incidence of VCI that concluded in, or after, 2006 (Table 2). The results of the risk of bias assessment for each study are given in Supplementary Table 7. The studies identified in this review were conducted in 15 countries. Importantly, only one study reported VCI incidence in the UK [38]; this was a study in dichorionic (DC) and monochorionic (MC) twins, with VCI incidence in at least one twin at 6.3% (DC twins) and 19.2% (MC twins). Only one study from Japan and one from Romania reported VCI incidence in cohorts that included all pregnancies. The studies found that VCI occurred in 21/1311 (1.6%) (Japan) and in 43/18,500 (0.23%) (Romania) of all cord insertions [39, 40]. The other studies reported an overall incidence in singleton pregnancies only, or in specific pregnancy sub-groups, e.g. multiple pregnancies.

**Fig. 2:**
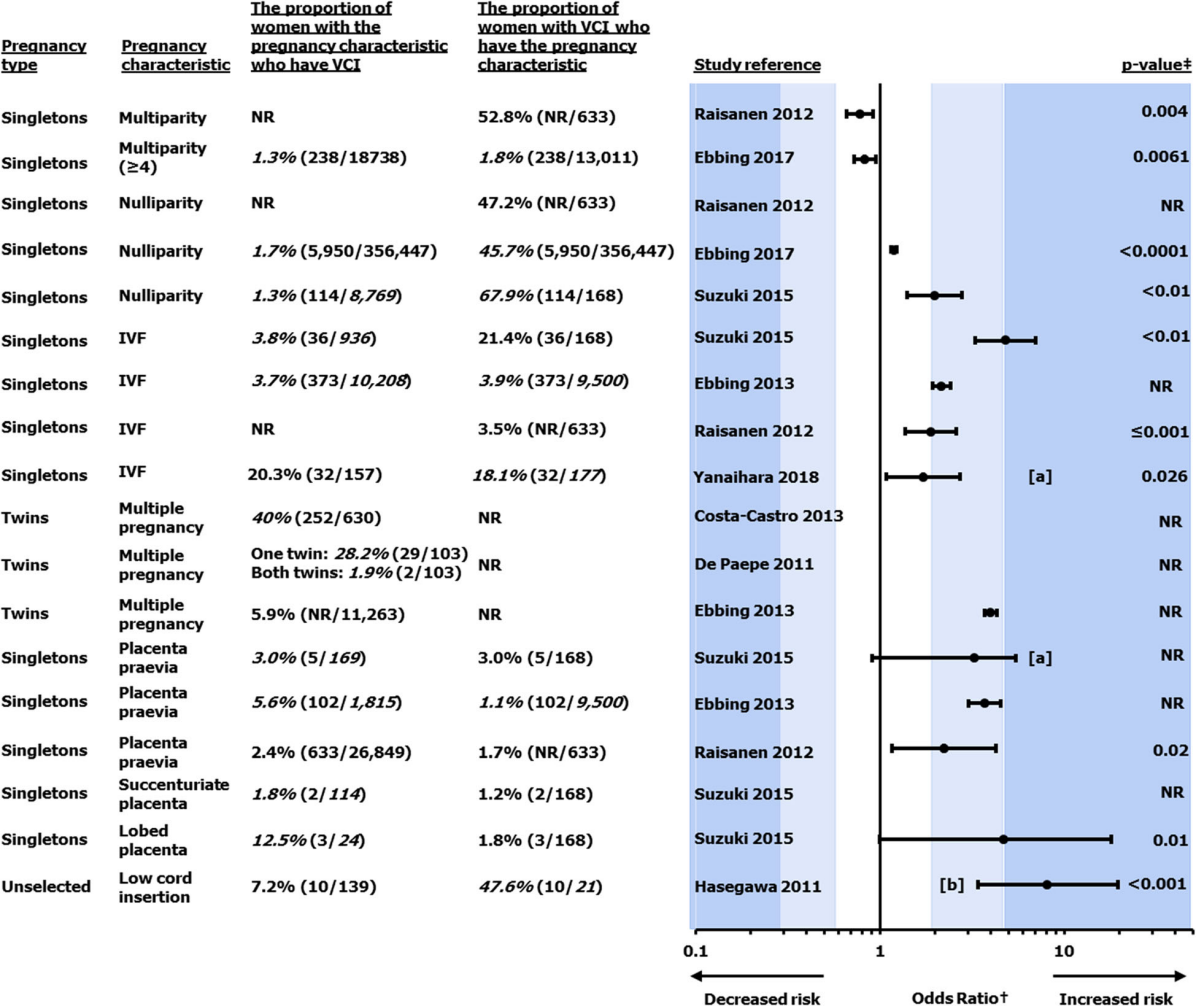
The association between VCI and various pregnancy characteristics. Numbers in italics were calculated by the review authors. Low cord insertion is umbilical cord insertion in the lower third of the uterus, or not visible [36].^**†**^Odds ratio (unless stated otherwise) of the pregnancy characteristic in pregnancies with and without VCI. ^**‡**^*p* value of the odds ratio. [**a**] Adjusted odds ratio. [**b**] Relative risk. IVF, in vitro fertilisation; NR, not reported; VCI, velamentous cord insertion

The incidence of VCI was reported to be low among singleton pregnancies, ranging from 0.4% [9] to 11.0% [41], with 11.0% appearing to be an outlier, given the second highest reported incidence value being 3.6% [41]. Incidence was reported to be higher in twin pregnancies (Fig. 2), ranging from 1.6 to 40% per pregnancy (affecting at least one fetus) [42, 43]. The reported incidence of VCI per fetus in twin pregnancies ranged from 3.5 to 20.7% [42, 44–48]. The highest VCI incidence of 40% per pregnancy was observed in monochorionic diamniotic (MCDA) twins [42].

Although the evidence was limited in volume, this review identified varying degrees of association between VCI and other pregnancy characteristics. However, regardless of the strength of the association found (in terms of the OR of VCI in pregnancies with vs without each characteristic), the overall concurrence of these factors with VCI in absolute terms remains relatively low (Fig. 2). VCI was reported to have a strong association with low cord insertion [39], a variable (weak to strong) association with in vitro fertilisation (IVF) [4, 7, 8, 41], a moderate association with placenta praevia [4, 7, 8] and a weak to moderate association with nulliparity [3, 8] (Fig. 2). Limited evidence from one small study (*n* = 24) suggests an association between VCI and a placenta defined as “lobed”; however, considering the sample size and width of confidence intervals, the strength of this evidence is low (Fig. 2) [8].

#### The association between VCI and VP

Eight studies reported the proportion of VP cases that also had VCI and indicated a strong association between the two (Table 3) [8, 49–55]. Two studies were in singleton pregnancies exclusively [8, 55], while the other six studies were in unselected pregnancies. The proportion of VP cases with concomitant VCI ranged from 20/49 (40.8%) to 3/3 (100%), with a median reported percentage of 76% [8, 49–55]. However, although most VP cases were reported to have concomitant VCI, the reverse was not true: only a minority of VCI pregnancies were reported to have VP. In Hasegawa’s 2010 publication, they reported a VP incidence of 9/84 (10.7%) among women with VCI [56], and in 2015, Suzuki et al. reported a VP incidence of 3/168 (1.8%) among women with VCI and 0/16,797 (0%) among women without VCI [8].

**Table 3:** Association between VP and VCI

Study	VCI cases in VP	VP in cases with VCI
Bronsteen et al. [49]	43/48 (89.6%)	NR
Hasegawa et al. [56]	NR	9/84 (10.7%)
Heller et al. [50]	3/3 (100%)	NR
Kanda et al. [51]	8/10 (80%)	NR
Melcer et al. [55]	21/32 (65.6%)	NR
Rebarber et al. [52]	21/29 (72%)	NR
Smorgick et al. [53]	10/19 (52.7%)	NR
Suzuki and Kato [8]	3/*3* (100%)	3/168 (1.8%)
Swank et al. [54]	*20/49 (40.8%)*	NR

Values in italics were calculated by the review authors

*NR* not reported

### Risk of adverse perinatal outcomes in women with VCI

Information on adverse perinatal outcomes in VCI pregnancies was provided in 19 of the publications identified in the review. The most commonly reported outcomes were neonatal and fetal deaths (reported in 12 publications), low birthweight (reported in 10 publications), low Apgar scores (reported in 6 publications), rates of emergency CS (reported in 6 publications) and pre-term birth or gestational age at birth (reported in 6 publications).

#### Neonatal and fetal death

**Fig. 3:**
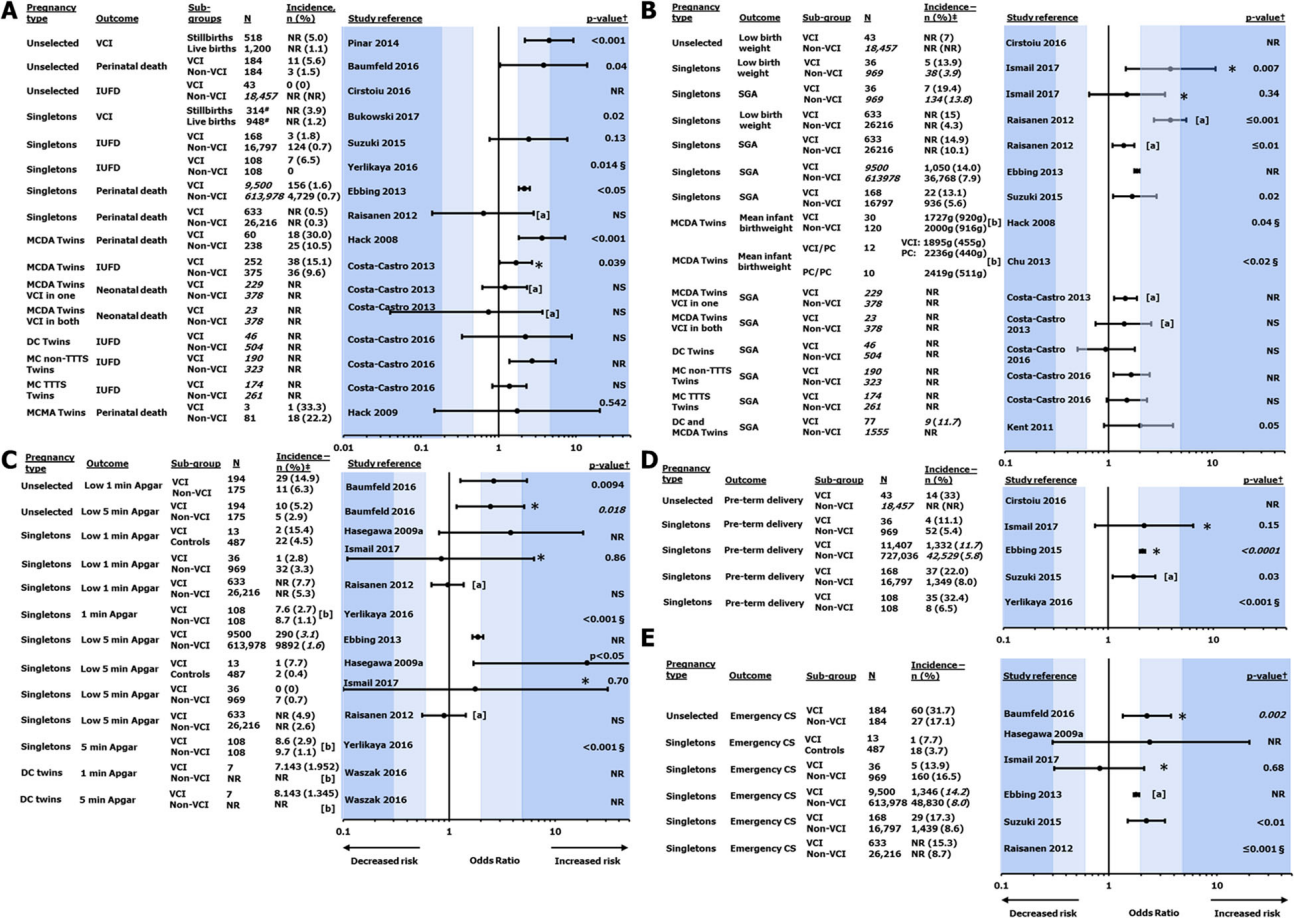
Incidence and risk of adverse perinatal outcomes in pregnancies with and without VCI. Numbers in italics were calculated by the review authors. Increased/decreased risk indicates increased/decreased with VCI, respectively. ^†^*p* value of the odds ratio unless stated otherwise. ^‡^Unless stated otherwise. ^§^*p* value for the difference in incidence unless stated otherwise. *Odds ratio calculated by the reviewer. ^#^Weighted number of cases (adjusted for missing data) presented. [**a**] Adjusted odds ratio. [**b**] Mean (SD). CS, caesarean section; DC, dichorionic; IUFD, intrauterine fetal death; MC, monochorionic; MCDA, monochorionic diamniotic; MCMA, monochorionic monoamniotic; NR, not reported; NS, not significant; PC, paracentral; SD, standard deviation; SGA, small for gestational age; TTTS, twin-twin transfusion syndrome; VCI, velamentous cord insertion

Neonatal and fetal deaths (intrauterine fetal death [IUFD], stillbirth or perinatal death) in pregnancies with VCI were reported in 12 publications. Five studies were conducted in singletons, 4 in twins and 3 in an unselected population that included both singleton and twin pregnancies [4, 7–9, 40, 42, 44, 48, 57–60]. In general, IUFD and neonatal or perinatal mortality were consistently reported to be higher in VCI than in non-VCI pregnancies (Fig. 3a).

Two studies evaluated the risk of IUFD in singleton pregnancies with and without VCI. A study of 16,797 singleton pregnancies, 168 of which had VCI, found that IUFD occurred in 1.8% of the VCI cases, compared to 0.7% non-VCI pregnancies, but the difference was not statistically significant [8]. However, a small case-control study of 108 VCI and 108 non-VCI singleton pregnancies found that IUFD was significantly more frequent in VCI cases (6.5% vs 0%, *p* = 0.014) [9].

Two studies evaluated the risk of perinatal death in singleton pregnancies with and without VCI. The larger of the two was a registry study with almost 9500 VCI cases and more than 600,000 non-VCI controls and found a moderate association between VCI and perinatal death; significantly more VCI pregnancies than non-VCI pregnancies (1.6% vs 0.7%) resulted in perinatal death [4]. The second of the 2 studies evaluating perinatal death in 26,849 pregnancies also found that perinatal death was more frequent among the 633 VCI cases (0.5%) than among the non-VCI cases (0.3%), but this difference was not found to be significant [7].

The 4 studies evaluating the relationship between VCI and neonatal and fetal deaths in twins varied in terms of the types of twins studied and tended to be smaller in size than the studies on singletons [42, 44, 48, 58]. Perinatal mortality was reported to be significantly higher in VCI than in non-VCI (30% vs 10.5%) MCDA twins [48, 58], and IUFD was found to be significantly more common in MC twins with VCI and without twin-twin transfusion syndrome (TTTS) [44].

#### Low birthweight and infants small for gestational age

A total of 10 studies reported birthweight as an outcome in VCI cases: 2 studies reported birthweight in twin pregnancies with VCI [58, 61], 3 looked at the proportion of VCI infants with low birthweight [7, 40, 62] and 7 examined the relative risk of infants being small for gestational age (SGA) at birth between VCI and non-VCI singleton or twin pregnancies [4, 7, 8, 42, 44, 47, 62]. In general, the evidence suggests a relationship between VCI pregnancies and the infants being SGA or having a low birthweight (Fig. 3b).

Two of the 3 studies evaluating low birthweight were performed in singletons and reported a greater incidence in VCI pregnancies: 13.9 to 15.0% of VCI pregnancies and 3.9 to 4.3% of non-VCI infants had low birthweight [7, 62]. The third study was performed in an unselected pregnancy cohort but only reported incidence of low birthweight (7%) among VCI pregnancies [40]. Three studies reporting on infants from singleton pregnancies being SGA reported that VCI was a significant risk factor, with a similar percentage of infants (13.1 to 19.4%) from VCI pregnancies reported to be SGA in each study [4, 7, 8, 62]. However, the evidence from studies on twin pregnancies was less clear. The 2 studies evaluating birthweight of infants reported this to be significantly lower among twins from pregnancies with VCI; however, they were either delivered pre-term or the gestational age at birth was not specified, which limits the generalisability of the results [58, 61]. The incidence of SGA infants was generally reported to be greater in twins from VCI pregnancies [42, 44, 47].

#### Low Apgar scores

Seven studies assessed the association between VCI and low neonatal Apgar scores: 5 in singletons, one in unselected pregnancies and one in DC twins [4, 5, 7, 9, 43, 57, 62]. Apgar scores at both 1 and 5 min were evaluated in all but one study, which included 5-min scores only [4]. Association between VCI and low Apgar scores was found in 2 out of 5 studies at 1 min, and 3 out of 6 studies at 5 min (Fig. 3c) [4, 5, 9, 57].

Definitions of “low” Apgar scores varied across studies (< 7 or ≤ 7), and while most studies evaluated the low Apgar score risk with OR, one case-control study compared mean and standard deviation (SD) of 1-min and 5-min Apgar scores between VCI and non-VCI pregnancies, finding both to be significantly lower in neonates from VCI pregnancies [9]. Another study only reported mean and SD of Apgar scores originating from DC twin pregnancies complicated by VCI [43]. Additionally, among the other studies that found low Apgar scores to be more common in VCI cases [4, 5, 57], one may have included multiple pregnancies [57]. In Raisanen et al.’s study, when the OR of low Apgar scores was adjusted for maternal characteristics and prior pregnancy complication history, the association with VCI was no longer significant [7].

#### Pre-term birth

Four studies reported on the association between VCI and pre-term birth (without specifying whether these were indicated or spontaneous) in singleton or unselected pregnancies [4, 8, 9, 62], and a further study reported the rate of pre-term birth in VCI pregnancies without comparison to non-VCI pregnancies [40]. Three out of 4 studies found a significantly higher incidence in VCI singleton pregnancies (11.7 to 32.4%) than in non-VCI controls (5.4 to 8.0%), though the effect was weak to moderate. In the fourth study, the OR showed a moderate but not statistically significant association between VCI and pre-term birth (Fig. 3d). This is consistent with the results of the systematic literature reviews (SLRs) by Vahanian et al. and de los Reyes et al., who found a moderate significant association of VCI with pre-term birth in singleton pregnancies [35, 37]. One study reported a linear regression of gestational age (GA) at birth for twin pregnancies where neither twin had VCI vs pregnancies where one or both twins had VCI. For dichorionic (DC) twins and MC twins without TTTS, GA was significantly lower (by approximately 2 weeks) when at least one twin had VCI; however, for MC twins with TTTS, the difference between VCI and non-VCI pregnancies was not significant [44].

#### Emergency CS

Six studies (1 in an unselected population, 5 in singletons) examined the proportion of VCI pregnancies delivered by emergency CS [4, 5, 7, 8, 57, 62], and 4 of those reported that significantly more VCI pregnancies (between 14.2 and 31.7%) resulted in birth by emergency CS, compared with 8.0 to 17.1% of non-VCI pregnancies (Fig. 3e) [4, 7, 8, 57]. Two studies found no significant difference in the rates of delivery by emergency CS between VCI and non-VCI pregnancies [62] or between VCI and normal pregnancies [5], respectively.

Very low numbers of studies evaluated the frequency of other adverse perinatal outcomes of VCI: abnormal fetal heart rate patterns (2 studies), admission to neonatal intensive care units (3 studies), fetal growth restriction (one study), placental abruption (2 studies) and pre-eclampsia (2 studies).

### Screening tests for VCI

Seven publications identified in this review reported the accuracy of second-trimester TAS for diagnosing VCI [16, 63–68]. Hasegawa’s 2006 report [69] is based on a cohort that overlaps with their 2005 report [65]; therefore, the 2006 report was included in the evidence synthesis to evaluate TAS test accuracy, but the 2005 publication was not considered further. The results of the quality and applicability assessment on the other 6 studies using TAS as part of a routine screening protocol are summarised in Table 4. Nomiyama et al., Pretorius et al., and Sepulveda et al. enrolled consecutive samples of patients and were at low risk of bias in this domain [16, 66, 67]. While all studies included in the review used second-trimester TAS as part of a routine screening protocol, the screening algorithm varied within and between studies: 3 used TVS when TAS was insufficient and only Nomiyama et al. [66] reported using the same screening methods for all participants, which is why it was the only study deemed to have low risk of bias in this respect. The studies’ risk of bias also varied because of differences in the reference standard (postnatal confirmation of VCI diagnosis) and the timing of screening (Table 4).

**Table 4:** Second trimester TAS for VCI screening studies. Summary characteristics

	Di Salvo 1998 [63]	Hasegawa 2006 [69]	Nomiyama 1998 [66]	Pretorius 1996 [16]	Sepulveda 2006 [67]	Sepulveda 2003 [68]
**Participant Selection**	Unclear [RoB: unclear]	Retrospective enrollment of pregnancies [RoB: high]	Prospectively enrolled consecutive samples of pregnancies [RoB: low]	Prospectively enrolled consecutive samples of pregnancies [RoB: low]	Prospectively enrolled consecutive samples of pregnancies [RoB: low]	Excluded pregnancies with inadequate amniotic fluid volume [RoB: high]
**Index Test/Technique**	Grey-scale TAS, with colour. Doppler imaging at the discretion of the sonologist [RoB: high]	Grey-scale TAS, with colour. Doppler imaging at the discretion of the sonologist [RoB: high]	Colour Doppler TAS. If inconclusive, 3rd trimester repeat testing, using Doppler TVS if needed. The same screening methods used for all patients [RoB: low]	TAS. Use of colour Doppler unclear [RoB: high]	Grey-scale TAS. Colour Doppler imaging at the discretion of the sonologist; supplemented with use of TVS if TAS was inconclusive [RoB: high]	Colour Doppler TAS, with colour Doppler TVS used if insufficient image quality was obtained from TAS [RoB: high]
**Reference Standard**	VCI confirmed by postnatal examination [RoB: high]	VCI confirmed by postnatal examination for all participants [RoB: unclear]	VCI confirmed by postnatal examination for all participants Placental cord insertion was obtained from medical records [RoB: high]	VCI confirmed by postnatal examination, performed by a pathologist blinded to the screening result [RoB: low]	VCI confirmed by postnatal examination for all participants [RoB: unclear]	VCI confirmed by postnatal examination for all participants [RoB: unclear]
**Participant Flow**	Postnatal exam for participants with perinatal complications or in multiple gestation pregnancies Long interval between the index test and the reference standard [RoB: high]	Long interval between the index test and the reference standard [RoB: high]	Long interval between the index test and the reference standard [RoB: high]	Many specimens were not sent for pathologic study by the obstetricians Long interval between the index test and the reference standard [RoB: high]	Long interval between the index test and the reference standard [RoB: high]	Long interval between the index test and the reference standard [RoB: high]
**Ultrasound Timing (GA, weeks)**	13 to 39	18 to 20	18 to 20	≥15	11 to 14	16 to 40

*GA* gestational age, *RoB* risk of bias, *TAS* transabdominal sonography, *TVS* transvaginal sonography, *VCI* velamentous cord insertion

Second-trimester TAS was consistently reported as having a high specificity (> 99.8% in all studies) and PPV (100% in 4 studies, and > 83% in all studies) (Table 5). However, there was considerable variation in sensitivity between studies (between 25 and 100%); the largest and highest quality study reported a low sensitivity of 62.5% [69]. Two of the 3 studies reporting a sensitivity of 100% used TVS when TAS was insufficient, meaning that the sensitivity of TAS alone could not be determined in these cases [67, 68]. One study reported that although researchers were reliably able to detect an abnormal cord insertion, the accuracy of diagnosing the specific type of abnormality (MCI vs VCI) was lower [63].

**Table 5:** Second trimester TAS for VCI screening studies. Test accuracy measures

Measure	Di Salvo et al. [63]	Hasegawa et al. [69]	Nomiyama et al. [66]	Pretorius et al. [16]	Sepulveda [67]	Sepulveda et al. [68]
**Sensitivity**	1/4 (25%)	25/40 (62.5%)	5/5 (100%)	2/6 (33.3%)	5/5 (100%)	7/7 (100%)
**Specificity**	50/50 (100%)	3406/3406 (100%)	580/581 (99.8%)	122/122 (100%)	528/528 (100%)	824/825 (99.8%)
**PPV**	1/1 (100%)	25/25 (100%)	5/6 (83%)	2/2 (100%)	5/5 (100%)	7/8 (87.5%)
**NPV**	50/53 (94.3%)	3406/3421 (99.6%)	580/580 (100%)	122/126 (96.8%)	528/528 (100%)	824/824 (100%)
**Accuracy**	51/54 (94.4%)	3431/3446 (99.6%)	585/586 (99.8%)	124/128 (96.9%)	533/533 (100%)	831/832 (99.9%)

*TAS* transabdominal sonography, *VCI* velamentous cord insertion, *NPV* negative predictive value, *PPV* positive predictive value

### Management pathways for VCI

No studies were identified that formally evaluated management pathways for women with VCI.

## Discussion

This rapid review aimed to establish the volume, quality and direction of the data that have been published on the epidemiology of VCI, the risk of adverse perinatal outcomes in pregnancies with VCI, screening tests for VCI and any management pathways that exist for identified cases of VCI.

A moderate number of studies were found to provide evidence on the epidemiology of VCI; the majority of which were cohort studies with a small number of case-control studies and were almost equally split in terms of prospective vs retrospective study design. Some studies were large enough to allow quantitative examinations, with statistical analyses of the incidence of VCI and its association with pregnancy characteristics and complications. However, others included only small numbers of women and therefore had very small numbers of VCI cases.

Based on the evidence identified in this review, the overall VCI incidence in the general population has been estimated to be between 0.23 and 1.6%, and VCI has been shown to have a strong association with VP, a strong association with twin pregnancy, a variable (weak to strong) association with IVF, a moderate association with placenta praevia and a weak to moderate association with nulliparity. These associations were found to be statistically significant in the large studies.

Most estimates of VCI incidence reported in the studies identified in this rapid review are likely to be reliable because almost all studies confirmed VCI by placental examination after birth with a specific investigation of cord insertion. Except for low cord insertion, abnormal placental forms and placenta praevia, the majority of the other pregnancy characteristics identified in this review (e.g. twin pregnancy, IVF and nulliparity) are scored objectively. Therefore, it is likely that the incidence of these pregnancy characteristics has been reported reliably.

The risks of some adverse perinatal outcomes in pregnancies with VCI, such as IUFD, perinatal death and infants born SGA, among others, were reported to be statistically significant in some studies, while others failed to demonstrate these associations. However, while the strength of these associations varied, the absolute incidence of most of the adverse perinatal outcomes among pregnancies with VCI was generally low. Despite the reasonably high quality of some of the studies evaluating the risk of adverse perinatal outcomes in VCI pregnancies, there was some heterogeneity in the results. For example, there was variation in the way in which IUFD and perinatal mortality were reported, with conflicting estimates of the strength of their association with VCI. Moreover, most of the reported ORs were not adjusted for the pregnancy characteristics that may have increased the probability of adverse perinatal outcomes, for example IVF, placenta praevia or various other potential confounders that were not considered in the current review. In terms of design, most studies evaluating adverse perinatal outcomes were either case-control or retrospective studies; case-control studies can be subject to selection bias and cannot be used to estimate absolute incidence, while retrospective studies can be limited by selection bias and variable reporting, which limits confidence in their estimates.

Two systematic reviews and meta-analyses on the risk of certain adverse outcomes in singleton pregnancies with VCI were identified during the update to the review [36, 37]. Due to the inclusion of studies conducted pre-2006, these were hand-searched, rather than included in the review in their own right. However, they found similar results to the rapid review, showing a statistically significant but weak association with pre-term birth, CS and SGA in one meta-analysis [37] and a moderate association with emergency CS in the other [36]. Neither publication reported meta-analyses of other outcomes, such as perinatal death.

The strong association between VP and VCI is the reason why the detection of this abnormal cord insertion is a key feature of proposed screening algorithms for VP. Only 7 studies, on 6 unique cohorts, reporting on screening tests for VCI were identified. All used second-trimester TAS, but between studies, there was inconsistency in the timing of the test and whether TVS was used when TAS was insufficient. Uneven application of both the index test and the reference standard, postnatal examination, limits the quality of the studies in the review. In addition, the sample size in most studies was low, which also limits the robustness of any conclusions that can be drawn from this evidence. Where identified, the specificity was consistently high, but the sensitivity of the screening tests varied considerably between the studies and was generally low. Furthermore, the sensitivity of TAS may not have been accurately estimated for various reasons: (1) although abnormal cord insertion was reported to be reliably detected, the inability to differentiate between MCI and VCI may have led to inaccurate classification [63]; (2) since ultrasound scans took place several weeks before birth, some cases of abnormal cord insertion correctly categorised as MCI during pregnancy may have progressed to VCI before birth [16], resulting in a lower estimate of TAS sensitivity; and (3) uneven application of the reference standard could have led to an overestimate of sensitivity. Overall, in studies identifying a limited number of cases, screening for VCI using TAS appears to have good overall accuracy, leading to the hypothesis that the test is reliable. However, this good accuracy is driven by high specificity. Well-designed prospective studies in larger cohorts of women would allow this hypothesis to be explored further and extend the issues covered to include the clinical utility of the test.

It is unsurprising that this rapid review did not identify any studies that formally evaluated management pathways for VCI, given the relatively low risks of adverse perinatal outcomes associated with VCI. However, the lack of a management strategy would be a concern if screening for VP were to be introduced, because screen-detected VCI would be a new form of routinely detected placental “abnormality” in up to 2% of singleton pregnancies [7]. This could lead to a cohort of women who may be at an increased risk of some adverse perinatal outcomes, but for whom there is currently no evidence-based risk-reducing intervention or management pathway. It is also unclear if and how identification of VCI would affect the quality of life in overdiagnosed expectant mothers. Screening for VCI is likely to also identify some cases of MCI; this rapid review did not consider the epidemiology or adverse perinatal outcomes associated with MCI. However, one of the studies included when updating the review found that, compared with VCI, MCI is associated with a lower risk of pre-labour rupture of membranes (PROM), pre-term PROM, short umbilical cord and spontaneous pre-term birth [3].

A limitation of this review was its use of rapid methods (a detailed examination of the strengths and limitations of the methodology has been published recently [70]): although the evidence was identified using thorough searches of medical databases, and a quality assessment was undertaken for each study, UK NSC evidence summaries stop short of meta-analyses. However, recently published meta-analyses have found results consistent with the rapid review for a subset of the outcomes of interest in singleton pregnancies [36, 37]. 

## Conclusions

The current rapid review has established that the evidence base exploring the epidemiology of VCI is limited in volume. Only one study, in twin pregnancies, was in a UK population, and each study included a relatively small number of VCI cases. The reported estimates for some adverse perinatal outcomes suggest a weak to moderate association with VCI, and for others, the estimates were conflicting between studies. The accuracy of the test for VCI has not been well established, and no management pathway is available for women with VCI detected by screening, nor for those with MCI who would also be detected if screening was to be implemented. These factors limit the strength of the evidence required to inform a UK NSC recommendation in this area. Further meta-analyses on a wider range of outcomes in subgroups of the pregnant population may help explore the level of risk from VCI more thoroughly. However, well-designed prospective studies in larger cohorts of women would be required to produce more robust estimates of VCI incidence and risks, test accuracy and the practicality of second-trimester testing for VCI. Modelling studies may be the first step towards understanding whether such studies would be achievable and of value.

## Supplementary information

**Additional file 1.** Screening for vasa praevia in the second trimester of pregnancy. The report of the rapid review described in this manuscript.

**Additional file 2.** PRISMA 2009 Checklist. The completed PRISMA checklist for this manuscript.

**Additional file 3.** Supplementary data

## Data Availability

The datasets used and/or analysed during the current study are available from the corresponding author on reasonable request.

## Abbreviations

CS: Caesarean section
DC: Dichorionic
IUFD: Intrauterine fetal death
GA: gestational age
IVF: In vitro fertilisation
MC: Monochorionic
MCDA: Monochorionic diamniotic
MCI: Marginal cord insertion
NPV: Negative predictive value
OR: Odds ratio
PPV: Positive predictive value
PROM: Patient-reported outcome measure
QUADAS-2: Quality Assessment of Diagnostic Accuracy Studies-2
RCT: Randomised controlled trial
SGA: Small for gestational age
SLR: Systematic literature review
TVS: Transabdominal ultrasound scan
TTTS: Twin-twin transfusion syndrome
TVS: Transvaginal ultrasound scan
UK NSC: UK National Screening Committee
VCI: Velamentous cord insertion
VP: Vasa praevia
