# Supplementary material for: Velamentous cord insertion: results from a rapid review of incidence, risk factors, adverse outcomes and screening
Source: Syst Rev. 2020 Jun 23;9:147. doi: 10.1186/s13643-020-01355-0 (PMC7313176; doi:10.1186/s13643-020-01355-0)
Supplement: Supplementary file 1 — Additional file 1. Screening for vasa praevia in the second trimester of pregnancy. The report of the rapid review described in this manuscript. [file 13643_2020_1355_MOESM1_ESM.pdf]

UK National Screening Committee

Screening for vasa praevia in the second trimester of pregnancy

External review against programme appraisal criteria for the UK National Screening Committee (UK NSC)

Version date: Final – June 2017

Costello Medical Consulting Ltd.

Review completion: March 2017

The UK NSC advises Ministers and the NHS in all four UK countries about all aspects of screening policy. Its policies are reviewed on a 3 yearly cycle. Current policies can be found in the policy database at http://legacy.screening.nhs.uk/screening-recommendations.php and the policy review process is described in detail at https://www.gov.uk/guidance/evidence-and-recommendations-nhs-population-screening#evidence-review-process

Template v2.0, August 2016

Summary

Plain English summary

This review looked at evidence about screening for vasa praevia using ultrasound (scanning) in mid pregnancy.

Vasa praevia is a rare but serious condition. It occurs when blood vessels from the umbilical cord lie across the cervix (the entrance to the womb) during pregnancy. When this happens the birth of the baby can damage the blood vessels. This can lead to heavy bleeding and sometimes to the death of the baby. A diagnosis of vasa praevia during pregnancy allows planning of a caesarean section. This may increase the chance of survival for the baby.

Screening all women for vasa praevia has been suggested. This could be useful in finding pregnancies at risk and preventing negative outcomes. A proposed screening method is to look for the condition at the fetal anomaly scan performed between 18+0 and 20+6 weeks. If there is a suspicion of vasa praevia, transvaginal scanning would be needed to confirm the condition. This is an intrusive, but usually painless, procedure that involves insertion of a probe into the vagina.

Some groups of women have a higher chance of their pregnancy being affected by vasa praevia. For example, when:

- the umbilical cord attaches to the membranes at the edge of the placenta (velamentous cord insertion)
- the placenta forms in two or more parts rather than one and blood vessels run between them
- the placenta is low in the womb
- the pregnancy is a result of in vitro fertilisation (IVF)

The UK National Screening Committee last looked at the evidence about vasa praevia in 2013. At that time, they decided that there was not enough evidence that screening every pregnancy for vasa praevia would be more helpful than harmful.

This was because:

- there was not enough information about the number of babies affected by it in the UK
- vasa praevia can be found by ultrasound testing but there is insufficient knowledge about the accuracy of the test
- a caesarean section to deliver the baby early would usually be recommended to prevent the effects of vasa praevia. However, this can bring its own complications
- some women may be advised to have an unnecessary, early caesarean section
- other women may be reassured but have a problem during delivery anyway

There was also a concern that screening would find pregnancies affected by velamentous cord insertion but there was no guidance on how to manage this.

The aim of this review was to look at the evidence to decide whether the current recommendation should change. The evidence found in this review is not enough to change the previous recommendation about screening for vasa praevia. There is still not enough evidence that screening every pregnancy for vasa praevia would be more helpful than harmful.

This review did not find any new evidence on how many pregnancies are affected by vasa praevia in the UK. There is only a limited amount of evidence from other countries. Also, there is not enough good evidence about the accuracy of the screening test for vasa praevia or velamentous cord insertion. This could lead to over-detection of the condition and unnecessary treatment. This review also did not find any new evidence about the best way of treating women with vasa praevia or velamentous cord insertion throughout the pregnancy.

Executive summary

Purpose of the review

This rapid review examines evidence on screening for vasa praevia (VP) during the second trimester of pregnancy, in populations similar to the UK pregnancy cohort.

Background

VP is a rare but serious condition where exposed blood vessels from the umbilical cord lie across the cervical os during pregnancy. If a case is undiagnosed and the baby is born naturally, the blood vessels can rupture, which could lead to fetal exsanguination, which can be fatal. Ultrasound screening for VP during the second trimester has been proposed to identify a group of women who would be offered a caesarean section (CS) to prevent the adverse consequences of the condition.

Published screening and management algorithms have identified a number of high-risk groups which have been associated with VP. These included multiple pregnancy, *in vitro* fertilisation (IVF) pregnancy, low-lying placenta in early pregnancy, and velamentous cord insertion (VCI). The first two groups are identifiable without screening and recommendations for testing for VP in these groups do not fall within the whole-population remit of the UK National Screening Committee (NSC). Second-trimester ultrasound assessment of placental localisation is recommended within current UK guidelines. Therefore, recommendations for the identification and onward management of associated risks in pregnancies with low-lying placenta is also not within the UK NSC's remit.

The situation relating to VCI is different. VCI occurs when the umbilical cord attaches to the membranes surrounding the placenta rather than to the central placental mass, leaving blood vessels exposed and unprotected by Wharton's jelly. A recent systematic review suggested that because of the strong association between VP and VCI it should be considered a marker rather than a risk factor VP. This aspect of the natural history of VP is an important issue but it is not the focus of this review.

However, because of the association between this cord abnormality and VP, detection of VCI in the general pregnant population is a central feature of screening algorithms. Ultrasound screening for VP in the general pregnant population, as currently proposed, would therefore involve identification of both aberrant vessels and the cord insertion site, both as means of identifying the risk of VP at delivery. This would represent a major departure from current UK practice.

The current review explores the evidence relating to the epidemiology and broad risk associations, the test accuracy, and management pathways for both VP and VCI.

CONFIDENTIAL

Previous recommendation

The UK NSC most recently considered the evidence for VP screening in 2013. The 2013 evidence review concluded that there was insufficient evidence to recommend universal routine antenatal screening for VP. The current position is that a national screening programme for VP at 18 to 20 weeks is not recommended.

Although the current literature suggests that VP is now detectable by ultrasound there is insufficient information on the case definition, natural history and epidemiology of the condition. There is also uncertainty on the accuracy and practical application of the test and there is no agreed management pathway for those with confirmed VP and for those with some risk factors in the absence of VP. In this context there is uncertainty about the balance of benefit and harm to be derived from screening all pregnant women with a view to offering CS to those at risk.

The questions providing the focus for this review address some of the gaps in the evidence identified in this statement.

Findings and gaps in the evidence

This review found that the overall incidence of VP is low, with estimates ranging from 0.02% to 0.04% in the largest, highest-quality studies. Among pregnancies with one or more risk factors for VP, the incidence is substantially higher. This review includes a meta-analysis of risk factors and markers providing consistent evidence that VCI, low cord insertion, abnormal placental forms, placenta praevia or low-lying placenta, and IVF have a positive association with VP. However, multiple pregnancy is not significantly associated with VP incidence, and no identified publications reported the association between multiparity and VP incidence. At least 80% of VP cases are reported to have one or more risk factors and the majority of reports identify the presence of VCI. Cases of VP that are not diagnosed prenatally are associated with severe adverse perinatal outcomes, including higher risk of emergency CS and perinatal mortality.

Proposals for screening for VP focus on the second-trimester ultrasound scan using transabdominal sonography (TAS) as the primary screening test. However, this review did not identify any studies examining the accuracy of TAS. Some combined it with transvaginal sonography (TVS) routinely, and others used TVS in cases of uncertainty or to confirm a suspected diagnosis of VP. As such, women with negative TAS results did not always receive a confirmatory test. Test accuracy characteristics were only reported for the composite testing pathways. There were also no studies that confirmed diagnoses for all participants (both screen-positive and screen-negative) at birth. Because of this, key performance measures of the primary screening test, such as sensitivity and specificity, could not be reliably established.

Up to a quarter of VP cases diagnosed in the second trimester have been reported to resolve before delivery, but it is unclear from published studies whether these cases were false positives on the initial screening or cases that genuinely resolved during pregnancy. Therefore, it is currently unclear how many of the VP cases identified during second-trimester screening resolve before birth.

VCI is relatively uncommon among singleton pregnancies, with an incidence of approximately 1.0% to 2.4%. Incidence in twin pregnancies is higher, ranging from 5.9% to 40%. Incidence of VCI is increased among pregnancies with one or more risk factors, particularly twin pregnancies, IVF pregnancies or in nulliparous women. The incidence of VCI among women without any known risk factors is unclear.

CONFIDENTIAL

VCI is associated with a range of adverse perinatal outcomes, most notably pre-term delivery and emergency CS in singleton pregnancies, and perinatal mortality in twins. Although these outcomes are serious, the increased risk is typically low or moderate compared to pregnancies without VCI.

The evidence base relating to TAS-based screening for VCI is confined to a limited number of studies. In a similar pattern to the VP test studies, not all women received the same screening or confirmatory test, there was variation in the timing of testing between and within studies and the number of cases identified was very small. The studies report a varied sensitivity, which was low in the largest study. However, the test appears to have very high specificity and positive predictive value (PPV). Among screen-detected cases there is no published evidence on optimal management pathways. If a systematic population screening programme for VP was recommended this could lead to an increase in the number of VCI cases detected as a by-product of screening. However, the value of this is uncertain given the low to moderate risk associations and the absence of agreed evidence-based management pathways.

Recommendations on screening

This review has not found sufficient evidence to support a change in the overall recommendation for VP screening. Key gaps in the evidence relating to the epidemiology, the test and the management pathway remain which are unlikely to be resolved without large scale, well designed, prospective studies.

Limitations

This rapid review was conducted in line with the UK NSC requirements for evidence summaries, as described at https://www.gov.uk/government/publications/uk-nsc-evidence-review-process/appendix-f-requirements-for-uk-nsc-evidence-summaries. These requirements are mostly in line with published guidelines for systematic reviews, but allowing for some methodological compromises. The most significant compromise made in this review was the exclusion of non-peer-reviewed evidence, which may have led to the exclusion of relevant evidence that has only been published in congress presentations or government reports. However, this is unlikely to miss any pivotal studies, as these would likely be published in peer-reviewed journals.

CONFIDENTIAL

Abbreviations

Abbreviation	Definition
**95% CI**	95% confidence interval
**BMI**	Body mass index
**CS**	Caesarean section
**DC**	Dichorionic
**FHR**	Fetal heart rate
**FTV**	Fetal thrombotic vasculopathy
**GA**	Gestational age
**IUFD**	Intra-uterine fetal demise
**IQR**	Inter-quartile range
**IVF**	*In vitro* fertilisation
**MCDA**	Monochorionic diamniotic
**MCI**	Marginal cord insertion
**MCMA**	Monochorionic monoamniotic
**NA**	Not applicable
**NR**	Not reported
**NPV**	Negative predictive value
**OR**	Odds ratio
**PPV**	Positive predictive value
**PRISMA**	Preferred Reporting Items for Systematic Reviews and Meta-Analyses
**SD**	Standard deviation
**SGA**	Small for gestational age
**TAS**	Transabdominal sonography
**TTTS**	Twin to twin transfusion syndrome
**TVS**	Transvaginal sonography
**VCI**	Velamentous cord insertion
**VP**	Vasa praevia
**VTS**	Vanishing twin syndrome

A glossary of terms relating to epidemiology and screening is provided in Appendix 6. Words and phrases included in the glossary are underlined in the body of the report.

CONFIDENTIAL

Contents

Summary ... **1**
Plain English summary ... 1
Executive summary ... 2
Abbreviations ... **5**
Contents ... **6**
1 Introduction and approach ... **8**
1.1 Background ... 8
1.1.1 Vasa praevia and velamentous cord insertion ... 8
1.1.2 Current policy context and previous review ... 9
1.2 Objectives ... 10
1.3 Methods ... 11
2 Synthesis of evidence ... **12**
2.1 Overall results ... 12
2.2 Question-level synthesis for vasa praevia ... 12
2.2.1 Criterion 1 – Epidemiology of vasa praevia ... 12
2.2.1 Criterion 4 – Screening tests for vasa praevia ... 21
2.2.2 Criteria 9 and 10 – Management pathways for vasa praevia ... 25
2.3 Question-level synthesis for velamentous cord insertion ... 27
2.3.1 Criterion 1 – Epidemiology of velamentous cord insertion ... 27
2.3.2 Criterion 4 – Screening tests for velamentous cord insertion ... 37
2.3.3 Criteria 9 and 10 – Management pathways for velamentous cord insertion ... 40
3 Review summary ... **42**
3.1 Conclusions and implications for policy ... 42
Do findings indicate whether screening should be recommended? ... 42
3.1.1 Summary of findings ... 42
3.1.2 Are there gaps in the evidence highlighted by the review? ... 43
3.1.3 Is further work warranted? ... 44
3.2 Limitations ... 44
3.2.1 Limitations of the available evidence ... 44
3.2.2 Limitations of the review methodology ... 44
4 Appendices ... **46**
Appendix 1 – Search strategy ... 46
Electronic databases ... 46
Search terms ... 46
Appendix 2 – Study selection ... 47
Review process ... 47
Eligibility criteria ... 47
Appraisal for quality and risk of bias ... 50
Appendix 3 – Included and excluded studies ... 51
PRISMA flowchart ... 51

CONFIDENTIAL

Publications included after review of full-text articles 52
Publications excluded after review of full-text articles.. 57
Appendix 4 – Study-level synthesis of results 59
Data extraction 59
Appraisal for quality and risk of bias 134
Appendix 5 – UK NSC reporting checklist for evidence summaries 140
Appendix 6 – Glossary of key terms 142
5 References 144

CONFIDENTIAL

1 Introduction and approach

1.1 Background

1.1.1 Vasa praevia and velamentous cord insertion

Vasa praevia (VP) is a pregnancy complication in which the fetal blood vessels cross or run near the internal cervical os (the opening to the birth canal) beneath the fetus.[1] Their location between the fetus and birth canal opening leaves them particularly at risk of rupture or compression, potentially leading to fetal exsanguination. This can happen at any time during the pregnancy, but is most likely during labour.[2] Due to this, preterm delivery by elective caesarean section (CS) is generally recommended for pregnancies complicated by VP.

VP is very rare. According to data from the Office of National Statistics showing 723,913 live births in England and Wales in 2011, and the reported incidence of approximately 1 in 2000 to 1 in 6000 pregnancies, it is expected to occur in approximately 117 to 350 pregnancies per year in England and Wales. However, it can lead to very severe outcomes for the baby. The classic presentation is painless vaginal bleeding and fetal distress or death following rupture of the membranes.[2]

Whereas the exact cause of VP is unclear, several risk factors have been proposed to be associated with development of VP, which include multiple pregnancy, pregnancies arising from *in vitro* fertilisation (IVF), low-lying placenta in early pregnancy, succenturiate placental lobes, bilobed or multilobed placentas, and velamentous cord insertion (VCI). Second-trimester ultrasound assessment of placental localisation is recommended within current UK guidelines.[3]

The situation relating to VCI is different. VCI is a defect in the manner and location of the umbilical cord insertion site. The umbilical cord is made up of a vein and two arteries enclosed by Wharton's jelly, a gelatinous material primarily made of mucopolysaccharides, and a layer of amnion. Together, the Wharton's jelly and amnion layer protect the umbilical blood vessels. The usual route of the blood vessels of the umbilical cord is from insertion in the middle of the placenta, to the blood vessels of the baby. In VCI, the umbilical cord inserts on the fetal chorioamniotic membranes rather than on the placental disc.[4] This leaves a part of the umbilical vessels unprotected by Wharton's jelly and more likely to rupture.[5]

A binary classification, based on pathological appearance, has been proposed for VP. Type 1 occurs as a consequence of a VCI into a placenta, whereas type 2 results from a multilobed placenta where vessels that connect the main placental plate with a succenturiate lobe are running over or near the internal cervical os.[2, 6] Type 1 VP is significantly more common than type 2 and a recent systematic review and meta-analysis suggested that because of the strong association between VP and VCI it should be considered a marker, rather than a risk factor, for VP.[7] This aspect of the natural history of VP is an important issue but it is not the focus of this review.

However, because of the association between this cord abnormality and VP, detection of VCI in the general pregnant population is a central feature of proposed screening algorithms. Ultrasound screening for VP in the general pregnant population, as currently proposed, would therefore involve identification of both aberrant vessels and the cord insertion site, both as

CONFIDENTIAL

means of identifying the risk of VP at delivery. This would represent a major departure from current UK practice.[8]

An example of a screening algorithm is shown in Figure 1 below, as proposed in Derbala 2007.[9] This algorithm builds on the routine second-trimester ultrasound scan using transabdominal sonography (TAS), performing additional colour Doppler TAS and/or colour Doppler transvaginal sonography (TVS) in pregnancies with additional risk factors or if there is any uncertainty after the routine TAS scan. A later algorithm proposed by Sinkey 2015 recommended that "ultrasound evaluation of the placental location and the relationship between the placenta and internal cervical os should be included at the second-trimester ultrasound scan, and the placental cord insertion site should be documented when technically possible," and pregnancies with low-lying placenta at the second-trimester scan should have follow-up testing in the third trimester.[10] The main aim of antenatal screening for VP is to prevent perinatal death, as well as other adverse perinatal outcomes.

Figure 1. Example of a proposed screening algorithm for VP (adapted from Derbala 2007[9])

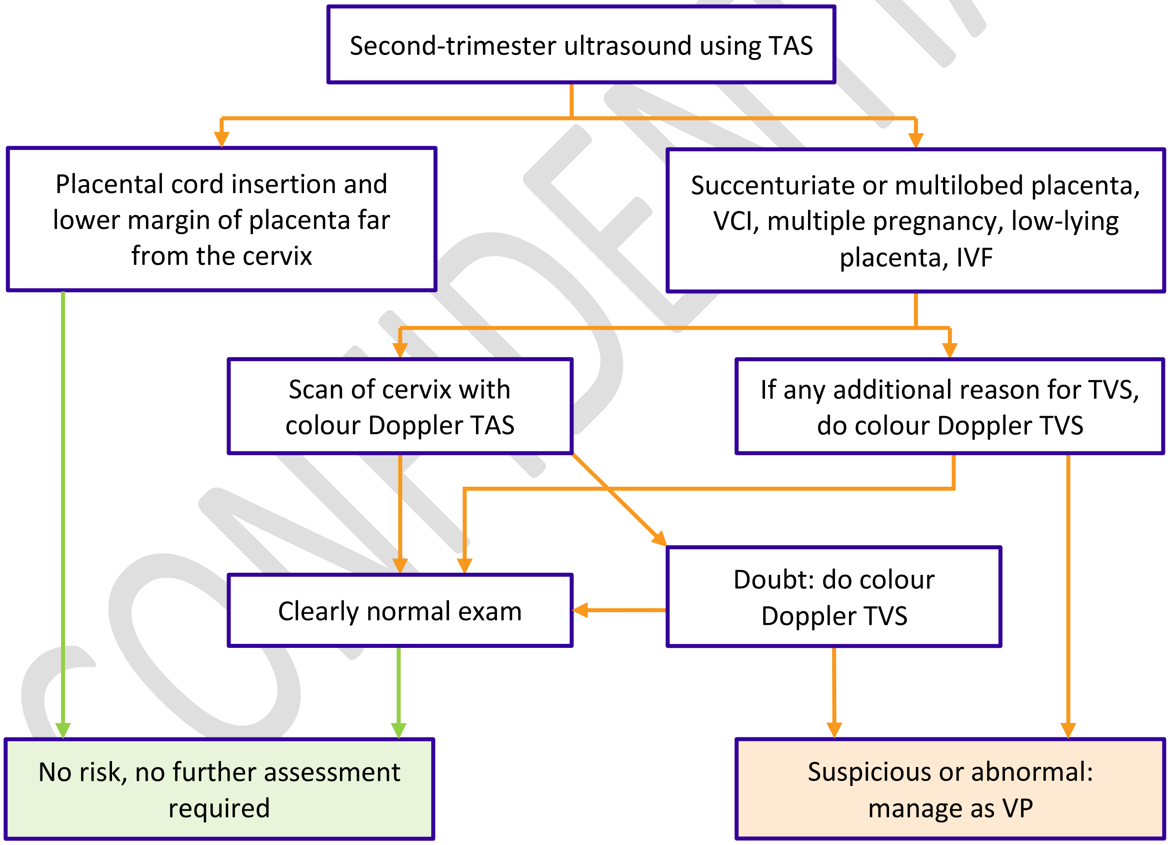

Abbreviations: IVF, in vitro fertilisation; TAS, transabdominal sonography; TVS, transvaginal sonography; VCI, velamentous cord insertion; VP, vasa praevia

CONFIDENTIAL

1.1.2 Current policy context and previous review

The UK NSC most recently considered the evidence for VP screening in 2013.[11] Universal routine antenatal screening for VP is not recommended at present. The 2013 evidence review highlighted a number of limitations that would make a prospective screening programme impractical.[11] The review noted that, while it is reported that VP can be detectable by

ultrasound, there is still insufficient information on the case definition, natural history and epidemiology of the condition.[11] Furthermore, there is uncertainty on the accuracy and practical application of the test and there is not an agreed management pathway for women that have been detected by screening as having VP, and for those with some risk factors in the absence of VP.[11] In this context there is uncertainty about the balance of benefit and harm to be derived from screening all pregnant women with a view to offering CS to those at risk.[11]

However, the previous review suggested that testing might be considered in pregnancies regarded to be at high risk of VP, such as multiple pregnancies, pregnancies arising from IVF, or those with a low-lying placenta in early pregnancy, succenturiate placental lobes, bilobed or multilobed placentas or VCI.[11]

The 2013 evidence review highlighted the fact that the binary classification of VP cases into type 1 and type 2 has tended to focus attention on universal screening for VCI.[11] However, other placental and cord variants are associated with VP, and VCI often occurs in parallel with other risk factors (such as IVF and placenta praevia) that are currently identifiable within existing guidelines and management pathways for pregnant women in the UK. While some of these groups can be identified within currently recommended UK practice, identification of women with VCI as a VP risk group would require a step change in practice relating to ultrasound examination in pregnancy.[8] The previous review did not formally assess the issues relating to the use of VCI as part of a screening strategy. If systematic population screening for VP was implemented, cases of VCI without VP would also be likely to be identified as a by-product of this programme. However, the incidence of VCI and burden of disease associated with it have not been considered separately in previous evidence reviews. Therefore, in addition to re-evaluating the evidence for screening for VP, this review aims to evaluate evidence on the epidemiology of VCI, the accuracy of second-trimester TAS for detecting VCI, and management strategies for diagnosed cases of VCI.

1.2 Objectives

This review aims to update the 2013 evidence review and assess whether there is any evidence for reconsidering the current screening recommendations for VP.

The specific questions addressed in this review are shown in Table 1 below, along with the relationship of each question to the UK NSC's Screening Criteria and the number of publications that were identified as providing relevant evidence for each question.

Table 1. Key questions for the evidence summary, and relationship to UK NSC screening criteria

Criterion		Key questions	# Publications included	
			VP	VCI
1	The condition should be an important health problem as judged by its frequency and/or severity. The epidemiology, incidence, prevalence and natural history of the condition should be understood, including development from latent to declared disease and/or there should be robust evidence about the association between the risk or disease marker and serious or treatable disease.	What is the incidence of VP in the UK?	25	-
		What percentage of VP cases identified in the second trimester will resolve by late pregnancy?	3	-
		What is the risk of adverse perinatal outcomes in pregnancies associated with VP?	13	-
		What is the incidence of VCI in the UK?	-	23
		What is the risk of adverse perinatal outcomes in pregnancies associated with	-	15

CONFIDENTIAL

		VCI?		
4	There should be a simple, safe, precise and validated screening test.	How effective is second-trimester TAS for detecting VP?	4	-
		How effective is second-trimester TAS for detecting VCI?	-	7
9 and 10	9: There should be an effective intervention for patients identified through screening, with evidence that intervention at a pre-symptomatic phase leads to better outcomes for the screened individual compared with usual care. Evidence relating to wider benefits of screening, for example those relating to family members, should be taken into account where available. However, where there is no prospect of benefit for the individual screened then the screening programme should not be further considered.<br><br>10: There should be agreed evidence-based policies covering which individuals should be offered interventions and the appropriate intervention to be offered.	What is the most effective management pathway for women with screen-detected VP?	0[a]	-
		What is the most effective management pathway for women with screen-detected VCI?	-	0

[a] Two 'studies of interest' were identified and discussed, although they did not strictly meet the eligibility criteria

1.3 Methods

The current review was conducted by Costello Medical Consulting Ltd., in collaboration with the UK NSC. Database searches were conducted on 5th July 2016 to identify studies relevant to the questions detailed in Table 1. The search strategy is presented in Appendix 1, and the methods of study selection (including full eligibility criteria and quality assessment checklists used) are detailed in Appendix 2.

CONFIDENTIAL

2 Synthesis of evidence

2.1 Overall results

Database searches yielded 625 unique results, of which 54 were ultimately selected for inclusion in the evidence synthesis. No additional relevant articles were identified through hand-searching the reference lists of relevant systematic reviews.

Appendix 3 contains a full PRISMA flow diagram (Figure 2), along with a table of the included publications and details of which questions these publications were identified as being relevant to (VP in Table 12, VCI in Table 13). A study-level summary of data extracted from each included publication is presented in Appendix 4, with publications stratified by condition (VP or VCI) and topic (epidemiology; performance of screening methods; management pathways). Where the reviewers have performed calculations on the data presented in the publications (for example, to calculate the sensitivity or specificity of a diagnostic test), this has been clearly indicated in the tables. Results of the quality assessments are also presented in Appendix 4.

2.2 Question-level synthesis for vasa praevia

2.2.1 Criterion 1 – Epidemiology of vasa praevia

Criterion 1 of the UK NSC Screening Criteria states that: 'The condition should be an important health problem as judged by its frequency and/or severity. The epidemiology, incidence, prevalence and natural history of the condition should be understood, including development from latent to declared disease and/or there should be robust evidence about the association between the risk or disease marker and serious or treatable disease.'

The 2013 evidence review identified no publications reporting the epidemiology of VP in the UK population specifically, either in the general population or in pregnancies with specific risk factors.[11]

In this review, the updated evidence relating to this criterion was addressed through three questions, discussed in sections 2.2.1.1, 2.2.1.1.2 and 2.2.1.3 respectively. Full details of all included studies are presented in Appendix 4, Table 16, and quality assessments are presented in Appendix 4, Table 21.

2.2.1.1 Question 1 – What is the incidence of vasa praevia in the UK?

2.2.1.1.1 Description of the evidence

In total, 30 publications conducted in 14 countries reported the incidence of VP in either a general pregnancy cohort or among pregnancies with specific risk factors.

VP is related to a number of risk factors and the rate of these risk factors appears to have changed over time.[12, 13] One large 20-year study found a significant increase in VP incidence between the period from 1988 to 1997 (0.7 per 10,000 pregnancies) and the period from 1998 to 2007 (2.6 per 10,000 pregnancies, $p<0.05$).[14] The study suggested that this may have been the result of either an increased proportion of pregnancies with VP risk factors in more recent years (such as conception by IVF), or more precise recording of cases.[14] Therefore, only studies completed since 2000 were considered to provide up-to-date, relevant data for this review.

CONFIDENTIAL

Five studies were completed before 2000 and are not considered further in this section. Results from one systematic literature review (SLR) and 24 primary publications on studies that completed since 2000, reporting on 17 unique cohorts of pregnant women, are considered below. Ten of these primary studies were included in the SLR and meta-analysis, but they have also been considered separately here where they present additional data not included in the meta-analysis.

Overall incidence

The 2013 evidence review found no publications reporting the incidence of VP in the UK. Since the previous review was conducted,[11] one relevant study has been published.[15] Donegan 2014 was a retrospective study examining clinical database records of 20,074 pregnant women from the UK who received pertussis vaccination during pregnancy from October 2012 to March 2013.[15] No cases of VP were reported in this cohort.[15] However, VP was only an event of secondary interest in this study and it is not clear whether women were being specifically monitored for VP during their pregnancy.

Studies including populations analogous to the UK pregnancy cohort were also included in this review. Overall incidence of VP was reported in one study from Spain,[16] two studies from the USA,[6, 17] four studies from Japan,[18-21] one study from Australia[22] and two studies from Israel.[14, 23] In addition, a recently-published SLR and meta-analysis (Ruiter 2016) reported the mean incidence of VP as calculated from 13 studies conducted in Israel, Japan, Spain and the USA, 10 of which presented data from studies completed since the year 2000.[7]

Reported incidence ranged from 0% in the UK study,[15] and a small Australian study,[22] to 0.31% in a small Japanese study,[19] with the largest studies reporting an incidence of approximately 0.02% to 0.04%.[14, 17, 23] The Ruiter 2016 meta-analysis calculated a mean incidence of VP of 0.60 per 1,000 pregnancies based on the pre-2000 and post-2000 studies.[7] Among the study from the UK and the ten studies from other comparable countries identified in this review (including all post-2000 studies captured in Ruiter 2016), there were a total of 176 cases of VP among 436,448 deliveries (0.039%, 3.9 cases per 10,000 deliveries).

As a number of the primary studies were retrospective database reviews, and it was often unclear how systematically VP cases were identified during the study period, it is likely that the overall VP incidence rates reported in these studies are underestimates. Ruiter 2016 reported that the overall methodological quality of the analysed studies was low and publication bias was a significant problem.[7] The SLR also identified a large difference in reported VP incidence between prospective and retrospective studies; the authors suggest that this might be because prospective studies focus more on detecting VP, and retrospective studies may have incomplete patient data.[7]

Incidence associated with risk factors

Although the incidence of VP is low overall, there are a number of risk factors that substantially increase the likelihood of developing VP. A recently-published systematic review and meta-analysis (Ruiter 2016) examined the association between various risk factors and the incidence of VP.[7] Ruiter 2016 included 13 studies reporting on 569,410 women with 325 cases of VP.[7] In these included studies, at least 83% of the VP cases had one or more risk factors, except for one study that examined a single risk factor (assisted reproductive technologies such as IVF), in which 63% of VP cases had this risk factor.[23] Ruiter 2016 also suggested that given the strong association of VCI with VP, VCI could be considered a marker for VP rather than a risk factor.[7]

The incidence of VP in pregnancies with known risk factors is discussed below.

CONFIDENTIAL

Velamentous cord insertion

Proportion of VCI cases that have VP: The previous review for the UK NSC reported that 2% of VCI cases will have VP.[11] In Hasegawa 2010, there was a VP incidence of 9/84 (10.7%) among women with VCI.[24] Suzuki 2015 reported a VP incidence of 3/168 (1.79%) among women with VCI and 0/16,797 (0%) among women without VCI (odds ratio [OR] not calculated).[21] Overall, the incidence of VP among pregnancies with VCI is reported to be between 1% and 10%.

Proportion of VP cases that have VCI: Seven studies reported the proportion of VP cases that also had VCI.[6, 14, 17, 20, 21, 25, 26] The proportion ranged from 20/49 (40.8%)[26] to 3/3 (100%),[25] with a median reported percentage of 80%.

Relative incidence of VP among women with and without VCI: Ruiter 2016 identified two studies (one of which was completed before 2000[27]) that stratified the incidence of VP according to the presence or absence of VCI. The OR for VP in women with VCI compared to normal cord insertion was found to be 672 (95% CI 112 to 4,034). This represents a substantially increased risk among women with VCI and led the authors to suggest that VCI might be a marker for VP rather than a risk factor.[7]

Conclusion: Overall, despite the small number of studies available, the evidence identified consistently supports a strong association between VCI and VP, in agreement with the proposed suggestion from Ruiter 2016 that VCI is a marker for VP.[7] It should, however, be noted that the majority of studies were retrospective and of low quality.

Umbilical cord insertion in the lower third of the uterus

Proportion of women with low cord insertion who have VP: The absolute incidence of VP among pregnancies with low cord insertion identified in the second trimester was 9/26 (34.6%) in Hasegawa 2010[24] and 1/35 (2.9%) in Hasegawa 2006.[19]

Proportion of VP cases that have low cord insertion: One further study identified in this review found that 10/10 (100%) of VP cases had cord insertion to the lower uterine segment during the second trimester, but the proportion of non-VP cases with low cord insertion was not reported.[20]

Relative incidence of VP among women with and without low cord insertion: Ruiter 2016 used these two studies to calculate an OR of 279.28 (95% CI 1.51 to 51,547.34, p=0.03) for the incidence of VP in pregnancies with cord insertion in the lower third of the uterus, compared to umbilical cord insertion in the middle or upper third of the uterus.[7] Although this effect size is very large, the confidence intervals are wide and there was high heterogeneity between the two studies included in the meta-analysis.[19, 24]

Conclusion: While the evidence suggests an association between VP and low cord insertion, it should be noted that the very limited number of studies, the small number of VP cases and the high level of variability in the reported incidence estimates limit the possibility of drawing robust conclusions. The strength of the association is unclear.

Succenturiate placenta or bilobed placenta

Proportion of women with abnormal placental forms who have VP: The incidence of VP among women with abnormal placental forms ranged from 1/92 (1.1%) to 4/94 (4.3%) across the three relevant studies identified in this review.[16, 24, 28]

Proportion of VP cases that have abnormal placental forms: Six studies reported the proportion of VP cases that had abnormal placental forms, with estimates ranging from 3/19 (15.8%) to 4/10 (40%).[6, 14, 16, 24, 26, 29]

CONFIDENTIAL

Relative incidence of VP among women with and without abnormal placental forms: Ruiter 2016 identified two studies[16, 30] that compared the incidence of VP between women with and without succenturiate or bilobed placenta, finding a strong association with an overall OR of 71.50 (95% CI 14.64 to 349.25, p<0.00001).[7] An updated analysis of one study[30] has been reported,[28] including a larger cohort of women, but the newer publication reports a similar OR to the earlier publication so would not have substantially changed the conclusion of the meta-analysis. An additional study identified in this review reported incidence of VP among women with 'abnormal placental forms' including multilobed, succenturiate and accessory placental forms, finding an OR of 34.0 (95% CI 9.4 to 122.6) for abnormal placental forms compared to normal placental forms,[24] which is consistent with the OR reported in Ruiter 2016.

Conclusion: There is a small body of evidence available that is consistent in suggesting that the incidence of VP is markedly higher in individuals with succenturiate or bilobed placentas than in individuals without abnormal placental forms.

Placenta praevia or low-lying placenta

Proportion of women with placenta praevia or low-lying placenta who have VP: Five studies reported the incidence of VP among women who had placenta praevia or low-lying placenta at any time during pregnancy,[16, 24, 25, 31, 32] with estimates ranging from 4/771 (0.5%)[32] to 4/113 (3.5%)[24] among cases of low-lying placenta or placenta praevia. Heller 2014 found VP in 3/20 women (15%) with low-lying placentas that had not cleared by birth, with VP diagnosed by ultrasound 0 to 1 days before birth.[25]

Proportion of VP cases that have placenta praevia or low-lying placenta: Eight studies reported the proportion of VP cases that had low-lying placenta or placenta praevia.[6, 14, 17, 20, 24, 26, 29, 33] The proportion ranged from 5/19 (26.3%)[14] to 9/10 (90%),[20] with a total of 202 cases of low-lying placenta or placenta praevia among the 346 VP cases (60.1%). However, Oyelese 2004 noted that while 95/153 (62.1%) VP cases had low-lying placenta during the second trimester, only 31/153 (20.3%) still had placenta praevia at birth.[29]

Relative incidence of VP among women with and without placenta praevia or low-lying placenta: Ruiter 2016 identified four studies that stratified the incidence of VP according to the presence or absence of placenta praevia.[7] No additional relevant studies were identified in the current review. The overall OR for VP in pregnancies with placenta praevia compared to pregnancies with a normally-positioned placenta was calculated as 18.97 (95% CI 6.13 to 58.68, p<0.00001), which represents a strong association and a substantial increase in the risk of VP among women with placenta praevia.

Conclusion: The evidence identified suggests a strong association between placenta praevia or low-lying placenta and VP.

Multiple pregnancy

Proportion of women with multiple pregnancy who have VP: Four studies reported the incidence of VP in multiple pregnancies,[16, 24, 28, 34] ranging from 0% in Hasegawa 2010[24] (N=157) and Suzuki 2010[28] (N=592), to 0.4% in first-born dichorionic twins and 1.1% in cases of vanishing twin syndrome in Evron 2015.[34]

Proportion of VP cases that are in multiple pregnancies: Six studies[14, 16, 24, 28, 33, 35] reported the proportion of VP cases that occurred in multiple pregnancies, ranging from 0% in two studies [24, 28] to 2/9 (22.2%) in another.[16] Overall there were seven multiple pregnancies among 680 VP cases (1.0%).

CONFIDENTIAL

Relative incidence of VP among women with and without multiple pregnancy: Ruiter 2016 identified three studies[16, 24, 33] that compared the incidence of VP in multiple pregnancies vs single pregnancies.[7] The overall OR from a meta-analysis of these three studies was 2.66 (95% CI 0.80 to 8.79, p=0.11) indicating that the incidence of VP in multiple pregnancies is not statistically significantly different to the incidence in singleton pregnancies. One study included in the meta-analysis, Baulies 2007,[16] found that although the incidence of VP was higher among multiple pregnancies, it was not an independent risk factor after adjusting for IVF, abnormal placental forms, and second-trimester placenta praevia. Two additional relevant studies were identified in this review.[28, 35] Baumfeld 2016 reported an OR of 0.81 (95% CI 0.05 to 13.44), implying a non-significantly lower risk of VP in multiple pregnancies.[35] Suzuki 2010 reported an incidence of 0/592 (0%) in twin pregnancies, compared to 3/11,311 (0.027%) in singleton pregnancies (OR not calculated).[28]

Conclusion: Overall, this evidence does not support an independent association between multiple pregnancies and VP. This contradicts the conclusion of the previous review.[11]

IVF pregnancy

Proportion of women with IVF pregnancies who have VP: Two studies reported a VP incidence of 4/824 (0.48%)[16] and 4/1173 (0.34%)[23] respectively among pregnancies resulting from IVF.

Proportion of VP cases that are in IVF pregnancies: Five studies reported the proportion of VP cases that were in pregnancies resulting from assisted reproductive technologies.[6, 14, 16, 20, 23] Although these studies were small, they reported consistent rates of IVF among VP cases, ranging from 4/12 (33.3%)[23] to 4/9 (44.4%).[16]

Relative incidence of VP among women with and without IVF pregnancies: Ruiter 2016 identified two studies[16, 23] that stratified the incidence of VP according to whether assisted reproductive technologies, including IVF, were used.[7] The combined OR was 18.95 (95% CI 6.61 to 54.34, p<0.00001) which is a strong association and a substantially increased risk compared to natural conception. No additional studies on this risk factor were identified in this rapid review.

Conclusion: A small number of studies reporting on a small number of VP cases are consistent in suggesting that pregnancies conceived through IVF or other assisted reproductive technologies have a higher incidence of VP than pregnancies conceived naturally.

Multiparity

Ruiter 2016 did not identify any studies that stratified the incidence of VP according to parity,[7] and this review also did not find any evidence relating to the association between parity and VP.

Conclusion: There is no evidence that parity is related to the incidence of VP.

2.2.1.1.2 Evidence summary

Only one UK study was found, which did not identify any VP cases.[36] A study by the UK Obstetric Surveillance System has been completed (surveillance period December 2014 to November 2015) but has not yet been published

However, studies on populations considered to be analogous to the UK population were identified that provide data on both the overall incidence of VP and the association of various risk factors with VP. Estimates of incidence in the general pregnancy population ranged from 0.02% to 0.04% in the largest studies.

In general, the evidence base tended to be limited in volume with many studies additionally identifying only a small number of VP cases. Ruiter 2016 reported that the overall

CONFIDENTIAL

methodological quality of the analysed studies was low and publication bias was a significant problem. The SLR also identified a large difference in reported VP incidence between prospective and retrospective studies. The authors also noted that the small number of cases of VP and the small number of individuals with each risk factor limited the statistical power, leading to wide confidence intervals on ORs.[7]

Nevertheless, many of the studies were consistent in concluding that while the incidence of VP is low among pregnancies as a whole, it may be substantially higher among women with certain risk factors. Publications identified in this review, including a recently-published systematic review and meta-analysis, provide evidence that an association may exist between VP and the following:

- VCI – very strong association, could instead be considered a 'marker' of VP
- Low cord insertion – association, unclear strength
- Abnormal placental forms – strong association
- Placenta praevia or low-lying placenta – strong association
- Pregnancies conceived through IVF or other assisted reproductive technology – strong association

Over 80% of cases had one or more of these risk factors. On the other hand, the studies identified in this review and Ruiter 2016 did not find evidence of a statistically significant association between VP and multiple pregnancy. No studies reported an association between VP and multiparity. However, it should be noted that the limitations of the evidence base restrict the certainty with which positive and negative conclusions on VP risk factors can be drawn. Without further primary research in large, unselected, cohorts it is unlikely that the results of the current scientific literature with regard to risk factors for VP could be validated or that more robust estimates of effect sizes could be provided.

Neither the 2013 evidence review nor this update identified any studies investigating the interplay of VCI with other risk factors when considering cases of VP.[11]

2.2.1.2 Question 2 – What percentage of vasa praevia cases identified in the second trimester will resolve by late pregnancy?

2.2.1.2.1 Description of the evidence

Three studies reported data relevant to this question.[6, 17, 26]

In Bronsteen 2013, 5/79 (6.3%) cases diagnosed by ultrasound performed at, or after, 15 weeks of gestation had subsequent ultrasound examinations and delivery information that did not support the VP diagnosis.[17] It is not clear whether these were VP cases that resolved, or false positive results from screening. It is also not clear what proportion of the ultrasound examinations were performed in the second trimester.

In Rebarber 2014, 5/29 (17.2%) prenatally diagnosed VP cases had resolved before birth.[6] All five cases that resolved were from cases diagnosed during the second trimester (5/21, 23.8%), and no cases that were diagnosed during the third trimester resolved (0/8, 0%).[6]

Swank 2016 identified 68 cases of VP, of which 4/68 (5.9%) resolved on repeat ultrasound at 21 weeks (1 case), 32 weeks (2 cases) and 33 weeks (1 case) of gestation.[26] A further 15/64 (23.4%) cases could not be verified at birth, so overall up to 19/68 (27.9%) cases may have resolved. The timing of the initial ultrasound in each of these cases was not reported.

CONFIDENTIAL

The key issue in all of these studies is that it is unclear whether cases that were identified on ultrasound and could not later be confirmed were genuine cases of VP that resolved, or false positives in the initial screen. Additionally, Rebarber 2014 was the only study that specifically reports the proportion of cases diagnosed during the second trimester that resolved before birth (23.8%),[6] whereas two studies may have included cases diagnosed during the first trimester (Swank 2016[26]) or third trimester (Bronsteen 2013[17] and Swank 2016[26]). However, the estimates reported in each of these studies represent an approximate upper bound for the number of cases that resolve before birth.

2.2.1.2.2 Evidence summary

Overall there is a limited amount of data available on this research question, with a major issue additionally identified with regard to data interpretation. Therefore, this aspect of the natural history of VP, which is key to understanding the potential benefits of VP testing in the second trimester, remains poorly understood. However, reported values can be assumed to represent a likely upper bound of approximately 6.3% to 27.9% on the number of VP cases that resolve by late pregnancy.

2.2.1.3 Question 3 – What is the risk of adverse perinatal outcomes in pregnancies associated with vasa praevia?

2.2.1.3.1 Description of the evidence

Relevant perinatal outcomes were reported in 13 publications. The most commonly reported outcomes were rates of emergency CS (9 publications), neonatal and fetal deaths (8 publications), pre-term birth or gestational age (GA) at birth (7 publications), and low Apgar scores (7 publications).

Abnormal intrapartum fetal heart rate patterns

Two publications reported the rate of abnormal intrapartum fetal heart rate (FHR) patterns.

Baumfeld 2016 reported FHR patterns in 33 VP fetuses and 29 non-VP fetuses.[35] A significantly higher proportion of VP fetuses had abnormal baseline heart rates (27.3% vs 6.9%, p=0.04), accelerations (48.5% vs 24.1%, p=0.05) and decelerations (60.6% vs 31.0%, p=0.02).[35] There was no significant difference between VP and non-VP fetuses in the percentage with each heart rate category (1st category 36.4% vs 48.3%; 2nd category 63.6% vs 51.7%; 3rd category 0% vs 0%; p=0.34) or the percentage with abnormal heart rate variability (39.4% vs 34.5%, p=0.69).[35]

In Kanda 2011, 1/10 (10%) cases had an ominous FHR pattern as the indication for delivery, and 4/10 (40%) cases had non-reassuring fetal status (such as occasional late decelerations and variable decelerations) as the indication for delivery.[20]

Admission to neonatal intensive care unit

Two publications reported the rate of admission to the neonatal intensive care unit.[17, 18]

6/67 (9.0%) infants in Bronsteen 2013 had hypovolemic shock at birth requiring intubation and neonatal intensive care unit admission.[17] Hasegawa 2015 reported that 11/15 (73%) of women admitted to the hospital and 2/6 (33%) of cases managed as outpatients required admission to the neonatal intensive care unit, for an overall rate of 13/21 (61.9%).[18]

Fetal growth restriction

Hasegawa 2015 reported that 5/15 (33%) of cases admitted to the hospital and 0/6 (0%) of cases managed as outpatients were small for gestational age (defined in the study as 'light for date'), for an overall rate of 5/21 (23.8%).[18] However, this was not compared to the proportion of small for gestational age infants among non-VP cases.

Low Apgar scores at 1 and 5 minutes

Seven publications reported Apgar scores at 1 minute and 5 minutes.[14, 16, 18, 20, 29, 35, 37]

Six studies reported Apgar scores in VP infants only, without comparing to cases without VP.[14, 16, 18, 20, 29, 37] In two small studies, all VP infants had normal Apgar scores at 1 and 5 minutes.[16, 37] Two studies, including one larger study of 100 VP infants, reported median Apgar scores of 8 and 9 and 1 and 5 minutes respectively, implying that most infants had normal scores.[18, 29] One study reported 6/10 (60%) infants with a low score (<8) at 1 minute, and 2/10 (20%) with a low score at 5 minutes,[20] and another study reported very low Apgar scores (<5) at 1 minute in 7/19 (36.8%) cases.[14]

Only one study compared Apgar scores for VP and non-VP infants. In Baumfeld 2016, 36.1% of 37 VP infants had low Apgar scores at 1 minute compared to 6.7% of 30 non-VP infants (OR 7.91, 95% CI 1.62 to 38.71, p=0.01), which is a significantly worse performance for VP infants. At 5 minutes, 22.2% of VP infants had low Apgar scores compared to 0% of non-VP infants (OR not calculated).[35] Therefore, although less than half of VP infants had low Apgar scores, the proportion was significantly higher in VP cases than non-VP controls.

Low birth weight

Six publications reported birth weight among VP cases.[6, 14, 16, 18, 20, 37] Two reported a mean or median birth weight less than 2500 g,[18, 20] and among the remaining cohorts the highest mean birth weight was 2675 g.[16] Overall this evidence suggests that infants born from pregnancies affected by VP tend to have a low birth weight, although in most of the studies the majority of pregnancies were delivered pre-term and correlation of birth weight to gestational age at birth was not performed. As none of the studies reported the birth weights for infants born from unaffected pregnancies for comparison, it is unclear if VP is associated with low birthweight.

Neonatal and fetal deaths

Eight publications reported neonatal and fetal mortality rates.

Four publications reported no perinatal deaths among VP pregnancies.[6, 16, 23, 24] In Baumfeld 2016, where cases were undetected during pregnancy and diagnosed during labour, there were 3 stillbirths and 1 intrapartum death in the VP group and no fetal deaths in the control group, but the difference was not significant (OR 8.19, 95% CI 0.42 to 158.55, p=0.16).[35] Two further studies reported mortality rates of 3/67 (4.5%)[17] and 1/19 (5.3%).[14] In many of these studies, VP was diagnosed prenatally in most or all cases and could therefore be managed accordingly.

One study compared perinatal mortality rates between cases that were or were not diagnosed prenatally.[29] The overall perinatal mortality rate in this study was 55/155 (35.5%), of which 30 were stillbirths and 25 neonatal deaths. Mortality was significantly lower among cases that were diagnosed prenatally (3.3% vs 56.4%, p<0.001).[29]

The results of these studies are striking; perinatal mortality among undiagnosed cases of VP is high, but can be reduced to less than 5% through appropriate management of cases if identified prenatally. Management pathways are discussed further in section 2.2.2.

Placental abruption

No publications reported placental abruptions.

Pre-term birth, including emergency CS

Eleven publications reported rates of pre-term birth, including emergency and elective CS.[6, 14, 16-18, 20, 23, 25, 26, 29, 35]

CONFIDENTIAL

Nine studies reported rates of emergency CS, which ranged from 7/60 (11.7%)[17] to 19/29 (65.5%).[35] Across all of these studies, emergency CS was required in 140/368 (38.0%) pregnancies.[6, 14, 16-18, 25, 26, 29, 35]

Two studies compared the rates of emergency CS between VP and non-VP pregnancies. One found a significantly higher risk in VP pregnancies (19/29 [65.5%] vs 7/25 [28.0%], OR 4.89, 95% CI 1.53 to 15.61, p=0.01)[35] while another small study found a large but non-significant increase in risk (1/3 [33%] vs 1/17 [5.9%], OR 8.00, 95% CI 0.35 to 184.38, p=0.19).[25]

A noteworthy factor reducing the risk of emergency CS among VP pregnancies was prenatal diagnosis of VP. Oyelese 2004 reported that in cases with prenatal diagnosis, where an earlier planned delivery was scheduled, there was a significantly reduced rate of emergency CS in 17/61 (27.9%) compared with 63/93 (67.7%) of cases without prenatal diagnosis (OR 0.18, 95% CI 0.09 to 0.37, p<0.0001).[29] Smorgick 2010 reported a non-significantly reduced rate of emergency CS in 5/10 (50%) in the group with prenatal diagnosis compared to 7/9 (77.8%) of cases in the group with no prenatal diagnosis (OR 0.29, 95% CI 0.04 to 2.11, p=0.22).[14]

Seven studies reported GA at birth or the proportion of pregnancies delivered pre-term.[6, 14, 16-18, 20, 23] In two studies all infants were delivered pre-term,[6, 18] and in Bronsteen 2013 all twin pregnancies were delivered pre-term.[17] The lowest reported proportion of pre-term deliveries was 4/9 (44%) in Baulies 2007.[16]

2.2.1.3.2 Evidence summary

According to the evidence identified in this review, the most frequent perinatal outcomes associated with VP are low birth weight, pre-term birth, and the need for emergency CS. A particular concern is perinatal mortality, which may occur in over half of VP cases if undiagnosed. It should be noted that most studies did not report comparable outcomes in a non-VP control group and did not adjust for potentially confounding risk factors, preventing estimation of the increased risk among VP cases. Therefore, further research in this area would be beneficial.

Analysis of evidence relevant to criterion 1 – Epidemiology of vasa praevia
**Quantity:** Overall, a relatively small number of studies were identified that provided evidence on the epidemiology of VP. Furthermore, many of these studies identified only a small number of VP cases as a result of the low incidence of VP. For some potential risk factors (such as multiparity) or outcomes (such as placental abruption), no studies were identified.
**Quality:** In the one study from the UK, it is not clear whether women were specifically monitored for VP, so the quality of the incidence estimate is low. A number of studies were retrospective database reviews and it was unclear how systematically VP cases were identified during the study period, so VP incidence is likely to be an underestimate overall. Ruiter 2016 identified a large difference in reported VP incidence between prospective and retrospective studies.[7] The meta-analysis reported in Ruiter 2016 provided higher-quality evidence for the estimates of relative incidence among women with and without risk factors. Estimates of adverse perinatal outcomes were not usually adjusted for other risk factors, which limits their quality.
**Applicability:** Only one study from the UK was identified. Other studies included in the review were from non-UK populations so the applicability may be lower, although they were all from countries pre-specified as being similar to the UK pregnancy cohort.

Consistency: The small sample sizes led to considerable variability between studies, with reported incidence associated with a risk factor typically varying by a factor of 10 among included studies. Ruiter 2016 noted that the small number of VP cases and the small number of individuals with each risk factor limited the statistical power, leading to wide confidence intervals on ORs.[7] However, the direction of the evidence was generally consistent.

Conclusion

While the scientific literature identified is broadly consistent in its conclusions on the epidemiology of VP, the limitations of the current evidence base mean that without further primary research in the form of large, prospective, UK-based studies, a better understanding of the potential impact of screening for VP in the second trimester is unlikely to be generated. Such studies would serve to substantiate the results of the smaller, mostly retrospective studies currently available, and could explore their applicability to the UK. They would also serve to fill important data gaps, for example with regard to the resolution of VP cases diagnosed in the second trimester by late pregnancy.

Summary: Criterion 1 **not met** for VP

2.2.2 Criterion 4 – Screening tests for vasa praevia

Criterion 4 of the UK NSC Screening Criteria states that: 'There should be a simple, safe, precise and validated screening test.'

The 2008 evidence review concluded that there was "a lack of evidence relating to the sensitivity of ultrasound for detecting VP", and in the 2013 evidence review "no studies were retrieved by the literature search which significantly alter this view".[11] Overall, the specificity was reported to be high and the false positive rate was reported to be low.

The proposed screening algorithm for VP would use TAS as the primary screening test, followed by confirmatory TVS for women with positive test results.[9] The focus of this review was therefore to identify studies reporting the accuracy of TAS when used as part of a routine screening protocol.

The updated evidence relating to this criterion was addressed through one question, discussed in section 2.2.2.1. Full details of all included studies are presented in Appendix 4, Table 17, and full quality assessments are presented in Appendix 4, Table 22.

2.2.2.1 Question 4 – How effective is second-trimester transabdominal ultrasonography for detecting vasa praevia?

2.2.2.1.1 Description of the evidence

Nine publications including eight unique cohorts of women reported the accuracy of ultrasound for diagnosing VP. However, only four studies used TAS as part of a routine screening protocol.[2, 17, 20, 38] The other studies were excluded from this review because they only carried out testing in at-risk women,[6] they did not have defined protocols for detecting VP,[14, 26] they only used TVS without TAS,[16] or because they reported on pregancies already considered in the four included studies (Lee 2000,[37] which reports a cohort overlapping with Bronsteen 2013[17]).

Additionally, a published systematic review (Ruiter 2015) examined studies reporting the accuracy of ultrasound for the diagnosis of VP.[39] The systematic review found that the accuracy of ultrasound is high when performed transvaginally and combined with colour Doppler. However, the systematic review did not report separate outcomes for studies using TAS rather

than TVS and did not perform any quantitative analyses due to insufficient data owing to heterogeneity.[39]

The quality and applicability of each identified study that used TAS as part of a routine screening protocol is shown in Table 2 below.

Table 2. Summary of QUADAS-2 assessments for VP screening studies

Study reference	Bronsteen 2013[17]	Catanzarite 2001[2]	Kanda 2011[20]	Nomiyama 1998[38]
PARTICIPANT SELECTION				
**Risk of bias**	High	Low	High	Low
**Concern about applicability**	Low	Low	Low	Low
INDEX TESTS				
**Risk of bias**	High	High	High	Low
**Concern about applicability**	High	High	High	High
REFERENCE STANDARD				
**Risk of bias**	High	High	High	High
**Concern about applicability**	Low	Low	Low	Low
PARTICIPANT FLOW				
**Risk of bias**	High	High	High	High

Participant selection

All four studies included all pregnant women screened at the study centre during the study period, with no studies making any inappropriate exclusions. The QUADAS-2 assessment does not explicitly distinguish between prospective and retrospective studies. However, two of the studies were conducted retrospectively through the review of medical records,[17, 20] and have therefore been assessed as having high risk of bias for this review.

Index test

All four studies used TAS as part of a routine screening protocol.[2, 17, 20, 38] However, they only reported composite outcomes from TAS and TVS, and none reported outcomes for TAS alone as the primary test. This limits the applicability of the results to the review question, which relates to the effectiveness of TAS as the index test.

An alternative approach would be to consider confirmatory TVS as part of the index test. However, generally only women with suspected VP after TAS received confirmatory TVS, so not all women received the same index test.

Three studies had high risk of bias because the participants did not all receive the same index test or combination of tests.[2, 17, 20] In two studies, confirmatory colour Doppler TAS and/or TVS were performed for some but not all patients.[14, 17] In the same two studies, the diagnostic criteria for VP were ambiguous or not reported,[17, 20] leading to further risk of bias.[14, 17] In one study, screening techniques changed over the study period.[2] Only one study used the same test for all patients and therefore had low risk of bias in this domain.[38]

Ultrasound techniques

Although all four studies used TAS, each study used a different overall test strategy:

- Bronsteen 2013 also used TVS and colour Doppler evaluation 'when needed to assist in the pregnancy evaluation'[17]
- In Catanzarite 2001, techniques changed over time;[2] initially, grey-scale TAS was used routinely with TVS in cases of uncertainty, and colour Doppler imaging was only used when VP was suspected. During the study routine imaging of cord insertion was introduced, and towards the end of the study routine colour Doppler or colour power angiography were added specifically to detect VP

CONFIDENTIAL

- In Kanda 2011, suspected VP diagnoses were confirmed with colour Doppler TAS or TVS[20]
- Nomiyama 1998 used routine colour Doppler TAS to assess cord insertion.[38] When VCI was found, the sonographer determined whether or not VP was present. All cases of VCI had repeat scans at 30 and 36 weeks; at this stage, if cord insertion could not be visualised by TAS, colour Doppler TVS was used instead

Ultrasound timing

The timing of screening varied between the four studies:

- Bronsteen 2013 performed the first ultrasound examination at 15 weeks or later
- Catanzarite 2001 performed scans at multiple times, and if the diagnosis of VP was suspected prior to 26 weeks of gestation, it was confirmed by TVS or by repeat TAS after 26 weeks
- Kanda 2011 screened for VP as part of second-trimester screening between 20 and 25 weeks of gestation, but for women attending the hospital in the third trimester (defined as 26 or more weeks of gestation), screening was performed as for the other women
- In Nomiyama 1998, all women were scanned at 18 to 20 weeks, with repeat scans at 30 and 36 weeks for all VCI cases

Reference standard

The target condition as defined by the reference standard in each study was VP, so there is no concern about the applicability of the studies in this regard. However, all four studies had a high risk of bias relating to the reference standard used. There is no gold standard method for confirming a diagnosis of VP antenatally or postnatally, or for identifying missed cases, and the method used in the four included studies was unclear. Where details were given, the reference standard was reported as 'outcome after delivery'[17] or 'based on the obstetrician's operative findings and placental pathological examination'.[2]

Participant flow

Participant flow led to a high risk of bias in all four studies. In particular, two studies reported that not all participants received a reference standard,[17, 38] and it was unclear in the other two studies whether all participants had received a reference standard.[2, 20] This adds further complication to the issue raised under 'Index Test' above that not all patients received the same index test.

The calculation of sensitivity requires accurate determination of the number of false negative cases; that is, pregnancies that appear not to have VP on screening but do have VP; likewise, the calculation of specificity requires accurate determination of the number of true negative cases. However, it was generally unclear in the studies whether women without a prenatal diagnosis of VP routinely received obstetric or placental examination to diagnose missed cases. Cases leading to adverse perinatal outcomes such as bleeding may be more likely to be noted in delivery reports, but asymptomatic cases would be unlikely to be identified. This may lead to an overestimation of the sensitivity of screening.

The interval between the index test and the reference standard was a further issue, as it is unclear whether cases that were identified on ultrasound and could not later be confirmed were genuine cases of VP that resolved, or false positives in the initial screen.

CONFIDENTIAL

Results

Measures of test accuracy that were reported in the four studies, or could be calculated from data presented in each publication, are shown in Table 3.

Table 3. Test accuracy measures in VP screening studies

Measure	Bronsteen 2013[17]	Catanzarite 2001[2]	Kanda 2011[20]	Nomiyama 1998[38]
**Sensitivity**	After initial scan (15+ wks): 26/58 *(44.8%)*<br><br>[Overall DR: 56/58 (96.6%)]	*13/15 (86.7%)*	Routine screening: 7/10 *(70%)*<br><br>[Overall DR: 9/10 *(90%)*]	1/1 *(100%)*
**Specificity**	NR	*33,192/33,193 (99.997%)*	NR	*585/586 (99.8%)*
**PPV**	[Overall 56/61 (91.8%)]	*13/14 (92.9%)*	7/7 *(100%)*	1/2 *(50%)*
**NPV**	NR	*33,192/33,194 (99.994%)*	NR	*585/585 (100%)*
**Accuracy**	NR	*33,205/33,208 (99.99%)*	NR	*586/587 (99.8%)*

Abbreviations: DR, detection rate; NPV, negative predictive value; NR, not reported; PPV, positive predictive value
Values in *italics* were calculated by a reviewer

These results should be interpreted in light of the quality concerns highlighted above, particularly the issues around the reference standard which likely lead to an overestimation of the true test accuracy. Also, some values and measures of test accuracy were calculated from information provided in the publications.

Among the four studies that used TAS as part of a routine screening protocol,[2, 17, 20, 38] the lowest sensitivity was 26/58 (44.8%).[17] The highest reported sensitivity was 1/1 (100%),[38] with the remaining two studies reporting sensitivities of 13/15 (86.7%) and 7/10 (70%).[2, 20]

Specificity was very high, ranging from 585/586 (99.8%)[38] to 93,855/93,858 (99.997%),[37] although two studies did not report specificity or sufficient details to calculate it. Positive predictive value (PPV) ranged from 1/2 (50%)[38] to 9/9 (100%).[16] The largest study with routine TAS reported a PPV of 56/61 (91.8%).[17]

This PPV implies that approximately one in 12 cases found on screening may be false positives, highlighting the need for third-trimester confirmation. Furthermore, results from Bronsteen 2013 and Kanda 2011 highlight that routine second-trimester screening alone is likely to miss a number of VP cases.[17, 20] In Bronsteen 2013, the largest study of mid-trimester screening, 56/58 (96.6%) cases were diagnosed prenatally.[17] However, when only the first ultrasound examination for each participant is taken into account (performed at 15 weeks or later), sensitivity was only 26/58 (44.8%). Of the 32 false negative cases at the first examination, 8 were missed and 24 had low-lying placenta, which precluded a diagnosis of VP. After the first ultrasound examination without a placenta covering the cervix, sensitivity was 45/58 (77.6%). Follow-up testing for suspected and inconclusive cases could therefore improve the overall accuracy of prenatal diagnosis.

2.2.2.1.2 Evidence summary

The question for this criterion asks about the use of second-trimester TAS for screening for VP. However, none of the included studies used TAS alone: some combined it routinely with TVS, and others used TVS in cases of uncertainty or to confirm a suspected diagnosis of VP. As such, women with negative TAS results did not always receive a confirmatory test. Screening test performance characteristics therefore refer to composite TAS and TVS, with TVS used in TAS positive women only. Furthermore, there is no reliable reference standard for the composite test results. Therefore, from the identified evidence it is not possible to reliably determine the accuracy of second-trimester TAS as the primary screening test.

CONFIDENTIAL

Overall, the reported sensitivity of second-trimester screening for diagnosing VP was variable, and it is not clear whether false negatives would have been systematically identified at delivery in these studies. The true sensitivity may therefore be lower than reported. However, guidelines suggest carrying out follow-up tests such as third-trimester TVS in women with VP diagnosed in the second trimester, which would be likely to increase the accuracy of the diagnosis.

Analysis of evidence relevant to criterion 4 – Screening tests for vasa praevia

Quantity: A limited number of studies on a small number of participants were identified. The overall screening protocol varied between these studies so the evidence for each strategy is very limited.

Quality: The quality of included studies was low because the index tests were inconsistent, not uniformly applied and most did not involve prospective routine screening for VP. There is no gold standard method for confirming VP antenatally or postnatally, and no study reported performing postnatal examinations routinely for all participants, so the measures of test accuracy are likely to be unreliable.

Applicability: None of the included studies reported the accuracy of TAS independently of TVS assessment. The four included studies all used confirmatory TVS and/or colour Doppler if needed, and for TAS positive women only composite results were reported.

Consistency: Specificity and PPV were relatively consistent, but sensitivity was variable between studies.

Conclusion

Four studies used second-trimester TAS routinely, with confirmatory TVS and/or colour Doppler when needed, and subsequent repeat testing in three studies. The reported screening test performance measures refer to the complete testing pathway, so there is no evidence relating to the accuracy of second-trimester TAS alone. There is uncertainty about the accuracy of screening due to the lack of prospective studies and the low quality of included studies. Overall, based on the current evidence there is insufficient evidence to support this criterion.

Summary: Criterion 4 **not met** for VP

2.2.3 Criteria 9 and 10 – Management pathways for vasa praevia

Criteria 9 and 10 of the UK NSC Screening Criteria state that:

- 'There should be an effective intervention for patients identified through screening, with evidence that intervention at a pre-symptomatic phase leads to better outcomes for the screened individual compared with usual care. Evidence relating to wider benefits of screening, for example those relating to family members, should be taken into account where available. However, where there is no prospect of benefit for the individual screened then the screening programme shouldn't be further considered.'
- 'There should be agreed evidence based policies covering which individuals should be offered interventions and the appropriate intervention to be offered.'

The 2013 evidence review identified mainly guidelines, case reports, modelling and decision analysis studies considering the impact of VP management strategies such as: delivery (and the timing of delivery) by CS; the administration of prophylactic steroids to aid lung maturity; early

hospitalisation of pregnant women; and *in vivo* operative fetoscopic laser ablation.[11] As such, the 2013 evidence review noted that, while delivery by CS to manage VP was recommended in guidelines and other publications, the recommendation was based on “clinical pragmatism and natural logic”.[11] It was also noted that “the low frequency of diagnosed cases of VP likely… [precludes] …any prospective trial to address the proper timing of delivery”.[11]

The evidence relating to these criteria was addressed through one question, discussed in section 2.2.3.1.

2.2.3.1 Question 5 - What is the most effective management pathway for women with screen-detected vasa praevia?

The previous evidence review indicated that prenatal diagnosis may be advantageous for preventing negative outcomes, however, details of the management pathway, such as in hospital vs outpatient management, or the timing of hospital admission or planned delivery, remained unclear.[11]

This review looked for analytical studies (interventional or observational) investigating any of the following management strategies:

- Timing for late pregnancy follow-up ultrasound
- Interventions in the third trimester to avoid negative outcomes
- Timing for elective CS
- Commencement of antenatal corticosteroids for fetal lung development if CS is recommended

No studies comparing outcomes relating to the management strategies of interest were identified. However, two comparative observational studies that may be of interest for considering possible VP management pathways were identified. Oyelese 2004 compared outcomes for women with and without a prenatal diagnosis of VP,[29] and Hasegawa 2015 compared inpatient and outpatient management.[18] These studies suggest that a prenatal VP diagnosis is associated with better perinatal outcomes than VP diagnosed at birth. An assumption could also be made that in prenatally diagnosed VP cases an elective CS before rupture of membranes could prevent adverse perinatal outcomes, a conclusion that was also reached in the previous UK NSC evidence review[11]. However, the optimal timing of hospitalisation or CS remains unclear.

The evidence obtained from each study was of low quality. Oyelese 2004[29] was particularly affected by selection bias and Hasegawa 2015[18] was particularly affected by confounding. Both studies were retrospective, with Hasegawa 2015[18] in particular resembling a description of a case series rather than a comparative study. Regardless of the fact that a prospective clinical trial to assess the effectiveness of management pathways may not be practical due to low VP incidence, the UK NSC screening criteria cannot be met without high-quality evidence on the effectiveness of management pathways for VP.

2.2.3.1.1 Description of the evidence

No relevant studies were identified that compared outcomes relating to any management strategies of interest.

CONFIDENTIAL

2.2.3.1.2 Evidence summary

Similarly to the findings of the previous review,[11] no studies were identified that specifically addressed this question. Given the continued lack of robust evidence derived from prospective studies, neither criterion 9 nor 10 have been met for VP.

Analysis of evidence relevant to criteria 9 and 10 – management pathways for vasa praevia
**Quantity:** Not applicable.
**Quality:** Not applicable.
**Applicability:** Not applicable.
**Consistency:** Not applicable.
**Summary:** Criterion 9 **not met**; criterion 10 **not met** for VP

2.3 Question-level synthesis for velamentous cord insertion

2.3.1 Criterion 1 – Epidemiology of velamentous cord insertion

Criterion 1 of the UK NSC Screening Criteria states that: 'The condition should be an important health problem as judged by its frequency and/or severity. The epidemiology, incidence, prevalence and natural history of the condition should be understood, including development from latent to declared disease and/or there should be robust evidence about the association between the risk or disease marker and serious or treatable disease.'

The evidence relating to this criterion was addressed through two questions, discussed in sections 2.3.1.1 and 2.3.1.1.2 respectively. Full details of all included studies are presented in Appendix 4, Table 19, and quality assessments are presented in Appendix 4, Table 24.

As a practical measure for conducting this rapid review, given the large number of studies that have been conducted within the last 10 years, any studies reporting on the incidence or perinatal outcomes of VCI pregnancies that concluded prior to 2006 were excluded.

2.3.1.1 Question 6 – What is the incidence of velamentous cord insertion in the UK?

2.3.1.1.1 Description of the evidence

In total, 23 publications conducted in 11 countries, and that concluded in or after 2006, reported the incidence of VCI either overall or in pregnancies with specific risk factors (Table 13).

Overall incidence

There were no studies reporting on VCI incidence in the UK. VCI incidence was reported in four studies from Japan, two on a mixed cohort from Portugal and the Netherlands, two additional studies from the Netherlands, two from Norway, two from the USA, one from Austria and one from Finland.

Only one study reported VCI incidence in an overall cohort that included all pregnancies. This study was conducted in Japan and found that VCI occurred in 21/1,311 (1.6%) of all cord insertions.[40]

Singleton pregnancies

Six studies from four countries examined the incidence of VCI in singleton pregnancies, which ranged between 108/27,818 (0.4%) and 633/26,849 (2.4%).[1, 21, 41-44] In those six studies, there were a total of 13,373 VCI cases in 783,343 singleton pregnancies, giving an overall VCI incidence

CONFIDENTIAL

of 1.70%.[1, 21, 41-44] This is similar to the conclusions of the previous UK NSC review that found the reported VCI incidence to be 1% in singleton pregnancies.[11]

Twin pregnancies

Ten studies were conducted on eight separate cohorts of twins.[1, 4, 45-52] VCI incidence was reported per pregnancy in five cohorts and ranged from 5.9% in 11,263 to 252/630 (40%) twin pregnancies.[1, 45] VCI incidence per insertion was reported for six cohorts and ranged from 46/1302 (3.5%) to 393/1,896 (20.7%).[4, 45, 47, 48, 51, 53] In three cohorts incidence was only described per insertion.[4, 48, 51, 52]

Only one study included all types of twin pregnancies and reported a VCI incidence of 5.9% among 11,263 twins.[43]

Dichorionic (DC) twins: Amongst two cohorts of DC twins, one study found that 46/550 (8.4%) of pregnancies were affected by VCI, with a per insertion incidence of 50/1,100 (4.5%) and another study reported VCI occurrence in 46/1,302 (3.5%) of insertions, but did not report a per pregnancy incidence.[48, 53]

Monochorionic diamniotic (MCDA) twins: Incidence amongst MCDA twins was reported in six cohorts, with VCI found in approximately 30/150 (20%) to 252/630 (40%) of pregnancies, with per insertion incidence rates varying from 31/330 (9.4%) to 393/1,896 (20.7%).[45, 46, 48, 51, 53]

Monochorionic monoamniotic (MCMA) twins: In one small study in MCMA twins, the reported VCI incidence was 3/84 (3.6%) per insertion, with the per pregnancy incidence rate not reported.[47]

Incidence associated with risk factors

VCI is a relatively uncommon condition in a singleton pregnancy and a more common one in twin pregnancies. Importantly, VCI is strongly associated with VP and a median of 80% of VP cases have been reported to have VCI (Section 2.2.1.1).

A systematic screening programme for VP would be likely to identify additional cases of VCI without VP as a part of the protocol of the screening process. Understanding the epidemiology of these cases and the associated risks would be important for understanding the implications of detecting VCI.

The incidence of, and odds ratios for, VCI in discrete sub groups within the pregnant population is discussed below.

Umbilical cord insertion in the lower third of the uterus, or not visible

Proportion of lower cord insertion among women with VCI: The absolute incidence of VCI among women with low cord insertion as diagnosed between 9 and 13 weeks of gestation by ultrasound was 10/139 (7.7%) in Hasegawa 2011.[40]

Proportion of VCI cases that have lower cord insertion: 10/21 (47.6%) VCI cases had low cord insertion at ultrasound performed between 9 and 13 weeks' gestation.[40]

Relative incidence of VCI among women with and without lower cord insertion: The relative risk (RR) for VCI in women with low cord insertion compared with normal cord insertion (as diagnosed by ultrasound between 9 and 13 weeks of gestation) was 8.1 (95% CI 3.4 to 19.6, p<0.001).[40] Although this was reported as a RR rather than OR, VCI is a rare outcome in singleton pregnancies, so the RR is approximately equal to the OR.

CONFIDENTIAL

Conclusion: Limited evidence from a small study suggests that there may be a strong association between low cord insertion between 9 and 13 weeks of gestation and VCI.

Succenturiate placenta or bilobed placenta

Proportion of succenturiate or bilobed placentas cases that have VCI: Suzuki 2015 reported a VCI incidence of 2/114 (1.75%) among women with a succenturiate placenta and 3/24 (12.5%) among women with a placenta defined in the study as "lobed";[21] the full definition of a "lobed placenta" was not clear.

Proportion of VCI cases that have succenturiate or bilobed placenta: 2/168 (1.2%) VCI cases had a succenturiate placenta and 3/168 (1.8%) of VCI cases had a "lobed placenta".[21]

Relative incidence of VCI among women with and without a succenturiate or bilobed placenta: A multivariate analysis found VCI to be independently associated with a "lobed placenta" with an OR of 4.70 (95% CI 1.0 to 18, p=0.01), whereas no significant association was found for VCI and a succenturiate placenta (p=0.41).[21] Although there is evidence that a "lobed" but not succenturiate placenta may be a risk factor for VCI, the reliability of this evidence is limited, as it is based on a single study where, as suggested by the authors, the sample size may not have been adequate to detect a difference and where criteria for the diagnosis of succenturiate or lobed placenta were not described.

Conclusion: Limited evidence from a small study suggests an association between VCI and a placenta defined as "lobed", however, considering the sample size and width of confidence intervals, the strength of this evidence is low.

Placenta praevia or low-lying placenta

Proportion of placenta praevia or low lying placenta cases that have VCI: Two studies reported VCI incidence in women with placenta praevia; this was 5/169 (3.0%) in Suzuki 2015[21] and 102/1,815 (5.6%) in Ebbing 2013.[1] Suzuki 2015 also reported a VCI incidence of 3/108 (2.8%) in women with low-lying placenta.[21]

Proportion of VCI cases that have placenta praevia or low-lying placenta: Three studies reported the proportion of VCI cases that had placenta praevia that ranged from 1.1% to 3%.[1, 21, 43] Raisanen 2012 reported that 1.7% of 633 VCI cases had placenta praevia,[43] Ebbing reported this to be 102/9,500 (1.1%)[1] and Suzuki 2015 reported a proportion of 2/168 (3%).[21] No studies reported the proportion of VCI cases that had low-lying placenta.

Relative incidence of VCI among women with and without placenta praevia or low-lying placenta: All three studies also reported a positive association between VCI and placenta praevia, with similar and statistically significant ORs ranging from 2.24 to 2.60.[1, 21, 43] No studies reported the association between VCI and low-lying placenta.

Conclusion: There is consistent evidence of a moderate association between placenta praevia and VCI. Between 1.1% and 3% of VCI cases have placenta praevia. There is insufficient evidence to draw conclusions of the association between the occurrence of low-lying placenta and VCI as only one study reported the incidence of VCI in women with a low-lying placenta, and this was not compared to the incidence in women without low-lying placenta.

Multiple pregnancy

Proportion of multiple pregnancies that have VCI: Apart from one study where all pregnancies were included[40] and one study that separately studied singleton and twin pregnancies,[1] all VCI incidence studies were specifically conducted either in singletons or in twins. As discussed in the 'Overall Incidence' section above, the incidence, though investigated in different studies, is

CONFIDENTIAL

pronouncedly lower amongst singleton compared with twin pregnancies. In the one study that separately reported VCI incidence, VCI was found in 1.5% (11,407/623,478) singleton pregnancies and in 5.9% of 11,263 twin pregnancies.[1]

Proportion of VCI cases that are in multiple pregnancies: In a case-control study of VCI pregnancies, Baumfeld 2016 identified 38/184 VCI cases (19.9%) in multiple pregnancies.[35]

Relative incidence of VCI between single and multiple pregnancies: As reported by Ebbing 2013, there was an increased risk for VCI in twins compared with singleton pregnancies with an OR of 4.0 (95% CI 3.7 to 4.3),[1] suggesting a strong association.

Conclusion: Limited evidence suggests a strong association between twin pregnancy and VCI and, based on limited evidence, around one in five VCI cases could be found in multiple pregnancies.

IVF pregnancy

Proportion of IVF cases that have VCI: There was only one study reporting VCI incidence amongst pregnancies conceived by assisted reproductive technologies; this identified 373/10,208 cases of VCI (3.7%) in singleton pregnancies conceived by assisted reproductive technologies.

Proportion of VCI cases that are in IVF pregnancies: Three studies reported on the proportion of VCI cases that originated from assisted reproductive technologies. [11, 29, 34] Two reports suggested that between 3.5% (n=633) and 373/9,500 (3.9%) of VCI cases originated from assisted reproductive technologies, whereas one reported a much higher proportion of 36/168 (21.4%).[11, 29, 34] This marked difference could originate from differences in study sample size or differences in assisted reproductive technology procedures between European and Asian fertility treatments; alternatively, it could suggest a role of genetic background in the modulation of the effect of assisted reproductive technology on VCI epidemiology.

Relative incidence of VCI between naturally conceived and IVF pregnancies: Three studies reported a moderate to strong association of assisted reproductive technologies with VCI, with ORs ranging from 1.89 to 4.82.[1, 21, 43] Thus, there is consistent evidence that pregnancies conceived with assisted reproductive technology have a higher risk of developing VCI, although the reported strength of the association varied.

Conclusion: There is consistent evidence of an association between assisted reproductive technologies and VCI. However, the strength of the association varied in the included studies. The reported proportion of VCI cases in assisted reproductive technology conceptions ranged between 3.5% and 21.4%.

Multiparity

Proportion of pregnancies of different parity that have VCI: Two studies reported the absolute VCI incidence stratified by parity. Suzuki 2015 found VCI to occur in 114/8,769 (1.3%) pregnancies among nulliparous women.[21] Ebbing 2015 reported VCI incidence for different levels of parity. In this cohort of singleton pregnancies, VCI incidence was highest in nulliparous women 5,155/324,568 (1.7%) and decreased to 213/16,960 (1.24%) in women with a parity of 4 or higher.[54]

Proportion of VCI cases that are in nulliparous pregnancies: Of all VCI pregnancies, the reported proportion that occurred in nulliparous women ranged from 45.2% (5,155/11,407) and 47.2% (n=633) in two studies,[43, 54] to 67.8% (114/168) in Suzuki 2015.[21] The markedly higher

CONFIDENTIAL

proportion reported by Suzuki 2015 could partly be explained by an overall higher proportion of nulliparous women (51.5%) in their cohort, as compared with between 40% and 45% of women being nulliparous in the other two studies.

Relative incidence of VCI between multiparous and nulliparous women: Three studies investigated the impact of parity on VCI incidence and found that multiparity was protective against VCI (OR 0.78, 95% CI 0.66 to 0.92, p=0.004), whereas nulliparity increased the risk of VCI (OR 1.99, 95% CI 1.4 to 2.8, p<0.01).[21, 43] In addition, one study found that the higher the parity, the lower the risk of VCI with a parity of four or higher being most protective (OR 0.59, 95% CI 0.50 to 0.69).[54]

Conclusion: Evidence from a limited number of studies is consistent that lower parity, and particularly nulliparity, is associated with a weak to moderate, statistically significant risk of developing VCI. The risk appears to decrease with increasing parity.

2.3.1.1.2 Evidence summary

No studies reporting on the epidemiology of VCI in the UK population were identified. Incidence of VCI in singleton pregnancies is reported to be between 0.4% and 2.4%, with estimates ranging from 1.0% to 2.4% in the largest, highest-quality studies. The incidence of VCI in twin pregnancies is significantly higher, ranging from 5.9% to 40% per pregnancy with at least one fetus having VCI, and with the highest VCI incidence rate of 20% to 40% observed in MCDA twins. Given that the incidence of VCI per cord insertion in twins (3.5% to 20.7%) is also higher than in singletons, twin pregnancy appears to be a risk factor for VCI.

The incidence of VCI is substantially higher among cases with a risk factor. The identified data was consistent that the following factors have a positive association with VCI, although the evidence was limited for several risk factors:

- Twin pregnancies – strong association
- Low cord insertion – strong association
- Placenta defined as "lobed" – association, unclear strength
- Placenta praevia – moderate association
- IVF – moderate to strong association
- Nulliparity – weak to moderate association

These conclusions are based on a limited number of studies, some of which had a small sample size. For example, the risk of VCI in low cord insertion pregnancies was investigated in a small study, and authors of the study examining the risk of VCI in pregnancies with abnormal placental forms declared that their sample size may not have been large enough to detect a difference. Sample sizes of studies reporting IVF, twin pregnancy, nulliparity and placenta praevia as risk factors for VCI were large, and the association between VCI and these factors was highly statistically significant.

Based on the available evidence, it is not possible to say overall how many VCI cases will be found in women with one or more of the risk factors listed above. The proportion of VCI cases in women with a less common risk factor such as lobed placenta or placenta praevia is low (1% to 4% of VCI cases) but the proportion of VCI cases found in nulliparous women, IVF pregnancies or multiple pregnancies can range from 3% to 47.6%. The association between risk factors was not reported, so the proportion of cases occurring in low-risk women cannot be estimated from the available data.

CONFIDENTIAL

As noted in section 2.2.1.1.2 above, the 2013 evidence review highlighted the fact that the binary classification of VP cases into type 1 and type 2 has tended to focus attention on universal screening for VCI.[11] However, other placental and cord variants can result in VP, and VCI can be combined with other risk factors (such as IVF and placenta praevia) that are currently identifiable within existing guidelines and management pathways for pregnant women in the UK. Neither the 2013 evidence review nor this update identified any studies investigating the interplay of VCI with other risk factors when considering cases of VP.[11] Therefore, it is not clear to what extent screening all women for VCI would contribute to the detection of additional cases of VP.

2.3.1.2 Question 7 – What is the risk of adverse perinatal outcomes in pregnancies associated with velamentous cord insertion?

2.3.1.2.1 Description of the evidence

Fifteen publications reported on relevant perinatal outcomes in VCI cases, including a comparison between the incidence of such outcomes in VCI cases and non-VCI controls. The most commonly reported outcomes were neonatal and fetal deaths (11 publications), low birth weight (8 publications), low Apgar scores (5 publications), rates of emergency CS (5 publications) and pre-term delivery or GA at delivery (4 publications).

Abnormal fetal heart rate patterns

Two studies reported on abnormal fetal heart rate (FHR) patterns and both compared VCI to non-VCI fetuses in two cohorts.[35, 41, 55] In Baumfeld 2016, FHR patterns were reported for 153 VCI fetuses and 154 non-VCI controls.[35] A significantly higher proportion of VCI fetuses had abnormal baseline FHR, abnormal variability and absent decelerations but there were no significant differences in the percentage with each heart rate category, proportion with absent accelerations or present decelerations, or percent showing a sinusoidal pattern.[35] The second study[41] found that of 13 VCI and 487 non-VCI fetuses in the first stage of labour, more VCI cases had total and atypical variable decelerations (VD) and VD without acceleration. In the second stage of labour, more VCI than non-VCI fetuses experienced loss of variability during VD, but did not differ in total or atypical VD, and in VD without acceleration.[41] There were no significant differences between VCI and non-VCI fetuses in other types of VD.[41] A second report on part of this cohort (12 VCI cases, 466 controls) additionally measured early deceleration, plate deceleration and prolonged deceleration during the first and second stages of labour, but found no differences in these parameters between VCI and non-VCI fetuses.[55]

Conclusion: Based on the available evidence, VCI may increase the risk of abnormal FHR patterns. The evidence is based on one case-control study and one study that only included 13 VCI cases, which may limit its reliability.

Admission to neonatal intensive care unit

Two studies reported on the percentage of singletons from VCI pregnancies that were admitted to neonatal intensive care units (NICUs). The first study reported that 1,359/9,500 (14.3%) of VCI infants were admitted to a NICU compared with 50,192/613,978 (8.18%) of non-VCI infants (adjusted OR 1.83, 95% CI 1.72 to 1.94).[1] Similarly, the second study found that of 25,317 infants, 17.2% of VCI vs 9.0% of non-VCI infants were admitted to a neonatal unit (adjusted OR 1.38, 95% CI 1.06 to 1.80, $p \leq 0.05$).[43]

Conclusion: There is consistent evidence from a limited number of studies that VCI in singleton infants poses a risk for admission to a neonatal care unit, although the association is only weak to moderate.

CONFIDENTIAL

Fetal growth restriction

Yerlikaya 2016 reported that 'growth retardation' was not significantly different between fetuses with VCI and those without VCI (6/108 [5.6%] vs 5/108 [4.6%], p=1.000).[44]

Conclusion: One small study found no increase in the risk of fetal growth restriction (FGR) among fetuses with VCI. This very limited evidence base is not sufficient to conclude whether VCI increases the risk of FGR.

Low Apgar scores at 1 and 5 minutes

Four studies reported Apgar scores at 1 and 5 minutes and one additional study reported Apgar scores at 5 minutes only.[1, 35, 41, 43, 44] All studies were conducted in singletons or in a predominantly singleton cohort and defined low Apgar scores as either <7 or ≤7.

Two out of four studies found the proportion of VCI (7.7% in n=633 and 15.9% in n=13) and non-VCI infants (5.3% in n=26,216 and 4.5% in n=788) with low 1 minute Apgar scores not to be significantly different.[41, 43] Whereas one of these is a large registry study,[43] the other report carries a caveat that infants with cord abnormalities other than VCI were excluded from the control group and analysed separately.[41] Conversely, another two studies (both case-control with a total of 302 VCI cases across the two studies) found a significant positive association between VCI and low 1 minute Apgar scores, but only one reported an OR (7.91, 95% CI 1.62 to 38.71, p=0.01).[35, 44]

Four out of five studies found a significant association between VCI and 5 minute lower Apgar scores.[1, 35, 41, 44] The largest of the four studies found 290/9,500 (3.1%) VCI infants and 9,892/613,978 (1.6%) non-VCI infants had low 5 minute Apgar scores (OR 1.87, 95% CI 1.66 to 2.10).[1] However, another large study found no increased risk of low 5 min Apgar scores for VCI (4.9% in n=633) vs non-VCI (2.6% in n=26,216) infants (adjusted OR 0.89, 95% CI 0.56 to 1.43).[43]

Conclusion: The evidence on the association of VCI with lower 1 or 5 min Apgar scores is inconsistent. ORs ranged from non-significant to strong association. Without conducting a meta-analysis, which is out of the scope of this review, it is difficult to confidently gauge this association.

Low birth weight

A total of eight studies reported birth weight as an outcome in VCI cases.[1, 21, 43, 45, 46, 48, 53, 56] Two studies reported birth weight in twin pregnancies with VCI,[43, 53] one looked at the proportion of VCI infants with low birthweight,[43] and six examined the risk of infants being small for gestational age (SGA) at delivery between VCI and non-VCI singleton or twin pregnancies.[1, 21, 43, 45, 48, 53]

One study in singletons reported that 15.0% of VCI (n=633) and 4.3% of non-VCI (n=26,216) infants had low birth weight (adjusted OR 3.93, 95% CI 2.75 to 5.62, p≤0.001).[43] Three singleton studies reported that VCI is a significant risk factor for SGA, with a similar percentage of VCI infants being SGA (13.1% to 14.9%).[1, 21, 43] In the largest of these studies, 1,050/7,488 (14.0%) of VCI and 36,768/467,482 (7.9%) of non-VCI infants were SGA (OR 1.87, 95% CI 1.66 to 2.10).[1]

In twin pregnancies, two studies found a significantly lower birth weight among VCI infants, however, these were either delivered pre-term or the gestational age at delivery was not specified, limiting the generalisability and reliability of the results.[46, 56] Two publications were unable to demonstrate VCI as a risk for SGA among DC or MCDA twins.[45, 53] A third study found SGA in 4/31 (12.9%) MCDA and 5/45 (11.1%) DC twins with VCI, with VCI being a significant risk factor for MCDA twins (OR 4.0, 95% CI 1.1 to 14.3, p=0.02) but not DC twins.[48]

CONFIDENTIAL

Conclusion: There is consistent evidence to conclude that there is an association between VCI and low birth weight or SGA in singleton pregnancies, although the reported strength of the association varies from weak to strong. The evidence for these outcomes in twin pregnancies is unclear.

Neonatal and fetal deaths

Altogether, there were 10 publications reporting on mortality; four were conducted in singletons, four in twins and two in an unselected population that included both singleton and twin pregnancies.[1, 21, 35, 43-47, 53, 57]

In a large study of singleton pregnancies, VCI was not a statistically significant risk factor for intrauterine fetal death (IUFD),[i] which occurred in 3/168 (1.8%) VCI and 124/16,797 (0.7%) non-VCI pregnancies (OR 2.44, 95% CI 0.77 to 7.76, p=0.13),[21] though a smaller case-control study found a significant difference in IUFD incidence between VCI (7/108, 6.5%) and non-VCI fetuses (0/108, p=0.014).[44] VCI was significantly associated with stillbirths in one reasonably sized case control study as it occurred in 5.0% of stillbirths and in 1.1% of live births (OR 4.50, 95% CI 2.18 to 9.27, p<0.001) in an unselected population.[57] Two out of three studies in singletons reporting on perinatal mortality found an increased risk of VCI for this outcome.[1, 35, 43] Two of the three studies reporting on perinatal mortality were large registry studies. Of those, one found a higher risk for perinatal death in VCI (156/9,500, 1.6%) than in non-VCI pregnancies (4,729/613,978, 0.8%, OR 2.14, 95% CI 1.83 to 2.52)[1] indicating a moderate association, but the other found no association, as 0.5% VCI (n=633) and 0.3% non-VCI (n=26,216) fetuses died perinatally (adjusted OR 0.64, 95% CI 0.14 to 2.86).[43]

In twins, IUFD was more common in MCDA twins with VCI (38/252, 15.1%) than without VCI (36/375, 9.6%) (OR 1.67, 95% CI 1.03 to 2.72, p=0.039), but VCI was not a significant risk factor for IUFD or neonatal mortality when DC and MCDA twins were studied together.[45, 53] Perinatal mortality was significantly higher in VCI (18/60, 30%) than in non-VCI (25/238, 10.5%) MCDA twins but not in MCMA twins.[46, 47]

Conclusion: There is insufficient or conflicting evidence to conclude whether VCI is a risk factor for IUFD, neonatal or perinatal mortality in singletons. Limited evidence suggests that VCI is associated with stillbirth in all pregnancies, and IUFD and perinatal mortality in MCDA twin pregnancies, but not with neonatal mortality in twins.

Placental abruption

Two studies in singleton pregnancies reported the incidence of placental abruption in VCI pregnancies. Ebbing 2013 observed placental abruption in 98/9,500 (1.0%) cases of VCI and 2,443/613,978 (0.4%) non-VCI cases (OR 2.60, 95% CI 2.12 to 3.18).[1] Suzuki 2015 found that placental abruption occurred in 5/168 (3.0%) VCI and in 101/16,797 (0.6%) non-VCI pregnancies (OR 5.07, 95% CI 2.0 to 13, p<0.01).[21]

Conclusion: There is a limited number of studies that suggest a moderate to strong association between VCI and placental abruption in singleton pregnancies.

---

[i] Suzuki 2015 did not report a definition for IUFD. Yerlikaya 2016 defined it as "death of the fetus during pregnancy before delivery after the 20th week of gestation".

CONFIDENTIAL

Pre-eclampsia

Two studies explored this outcome. Ebbing 2015 found a statistically significant association between pre-eclampsia and VCI in singleton pregnancies (OR 1.61, 95% CI 1.49 to 1.75, p<0.0001). They reported pre-eclampsia in 639/10,768 (5.6%) VCI and 25,792/727,036 (3.6%) non-VCI pregnancies.[54] In a case-control study, Yerlikaya 2016 reported pre-eclampsia in 5/108 (4.6%) VCI vs 7/108 (6.5%) non-VCI infants which was not statistically significant (OR 0.70, 95% CI 0.22 to 2.28, p=0.768);[44] however, this was a small study so had limited power to find a significant difference.

Conclusion: Results from the two studies are not consistent, with Ebbing 2015 reporting a weak association and Yerlikaya 2016 reporting no significant difference between VCI and non-VCI pregnancies. However, given that Ebbing 2015 was a substantially larger study than Yerlikaya 2016, the results may be more reliable, so there may be a weak association between VCI and pre-eclampsia.[44, 54] A meta-analysis would be required to explore this further.

Pre-term delivery and emergency CS

Three studies reported on the association between VCI and pre-term delivery in singleton pregnancies.[1, 21, 44] The largest study found that 1,332/10,075 (11.7%) VCI and 42,529/727,036 (5.9%) non-VCI pregnancies delivered pre-term (OR 2.13, 95% CI 2.01 to 2.25, p<0.0001).[1] Suzuki 2015 also found an increased risk of VCI for pre-term delivery with 37/168 (22.0%) VCI foetuses delivered pre-term vs 1,349/16,797 (8.0%) of non-VCI foetuses (adjusted OR 1.74, 95% CI 1.1 to 2.8, p=0.03).[21] Similarly, Yerlikaya 2016 found that pre-term delivery was more common in VCI cases (35/108, 32.4%) than non-VCI controls (8/108, 6.5%, p<0.001).[21] By contrast, a crude regression coefficient for a shorter gestational age at birth was not significantly different between MCDA twin pairs with and without VCI in the single study exploring this issue.[53]

Four studies examined the proportion of VCI pregnancies delivered by emergency CS.[1, 21, 35, 43] Between 14.2% (1,346/9,500)[1] and 17.3% (29/168)[21] of VCI pregnancies were delivered by emergency CS, compared with 8.0% (48,830/613,978)[1] and 8.7% (n=26,216)[43] of non-VCI pregnancies (OR 1.80, 95% CI 1.69 to 1.91).[1]

Conclusion: There is consistent but limited evidence that there is a moderate association between VCI and pre-term delivery and emergency CS in singleton pregnancies. VCI may not be a risk factor for pre-term delivery in MCDA twins, though the evidence is based on a single small study. No conclusions can be drawn regarding the risk for pre-term delivery in non-MCDA twin pregnancies or for emergency CS in any twin pregnancies.

2.3.1.3 Evidence summary

The most frequent perinatal outcomes associated with VCI are low birth weight, pre-term delivery, need for emergency CS and admission to neonatal intensive care units. There is also a concern that VCI could be a risk for stillbirths in all pregnancies.

The UK NSC's criterion asks whether VCI is 'an important health problem in terms of its frequency and severity'. No studies estimating the incidence of VCI in a UK population were found. Studies in non UK populations suggest that VCI incidence is likely to be around 1% to 2%. As such a number of women could be at a low to moderate risk of a range of adverse perinatal outcomes. However, some women with VCI would be identified as being at risk of adverse perinatal outcomes through other risk management programmes due to having other, concomitant risk factors. A proportion of women with VCI would only be found if screening of the general population for VCI was implemented but the proportion is not clear from the identified evidence.

CONFIDENTIAL

Analysis of evidence relevant to criterion 1 – Epidemiology of VCI

Quantity: Overall, a moderate number of studies were identified that provide evidence on the epidemiology of VCI. This included some large, sufficiently statistically powered investigations that allow a quantitative examination of the incidence, risk factors and perinatal outcomes of VCI. However, some studies included only small numbers of women so had very small numbers of VCI cases.

Quality: In all studies identified, the VCI diagnosis was made during placental examination after delivery and in most studies cord insertion was specifically investigated, thus decreasing the likelihood that VCI incidence would have been underestimated. Apart from low cord insertion, abnormal placental forms and placenta praevia, the majority of risk factors and outcomes are conditions that are scored objectively and can thus be expected to be reported reliably.

Despite the reasonably high quality of the studies themselves, the total number and design of studies, and thus the overall quality of the evidence, is moderate as the majority of studies were either case-control or retrospective in design. Prospective, UK-based studies would provide the opportunity to substantiate the current findings of this review.

Applicability: Although no studies from the UK were identified, multiple studies reporting VCI incidence in populations analogous to the UK were found. Nevertheless, as the studies included in the review were from non-UK populations, the applicability to the UK population may be limited which could be a concern.

Consistency: The majority of risk factors and outcomes were consistently reported between large studies. However, on some important outcomes such as IUFD, neonatal mortality and pre-eclampsia data from the identified studies was not in agreement on the association with VCI. A meta-analysis may help explore these areas more thoroughly. Considerable variability of VCI incidence was reported among twin pregnancies, though this variability is likely to be due to different types of twin pregnancies having a different risk of developing VCI and the smaller sample sizes of studies investigating these.

Conclusion

Considering the limitations described above, it is unlikely that more precise estimates of the epidemiology of VCI in the UK will become available without further primary research. However, from the available evidence it is possible to draw a number of overall conclusions:

1. VCI is relatively uncommon among singleton pregnancies in the general population, with an incidence of approximately 1.0% to 2.4%
2. VCI is common in twin pregnancies in the general population, with incidence ranging between 5.9% and 40%, and highest in MCDA twin pregnancies
3. Incidence of VCI is increased among individuals with a risk factor, most notably in twin pregnancies, IVF pregnancies or nulliparous women
4. VCI is associated with a range of perinatal outcomes, most notably pre-term delivery and emergency CS in singleton pregnancies and perinatal mortality in MCDA twins. Associations were generally weak or moderate, but statistically significant
5. VCI is significantly associated with VP, which, if undiagnosed, is strongly associated with perinatal mortality. However, without large, prospective studies it is difficult to estimate the

number of VCI cases in women with no other VP risk factors, and the additional number of VP cases that could be identified within this group of women.

Summary: Criterion 1 **not met** for VCI

2.3.2 Criterion 4 – Screening tests for velamentous cord insertion

Criterion 4 of the UK NSC Screening Criteria states that: 'There should be a simple, safe, precise and validated screening test.'

The previous review did not formally address the accuracy of screening tests for VCI.[11] The evidence relating to this criterion was addressed through one question, discussed in section 2.3.2.1. Full details of all included studies are presented in Appendix 4, Table 20, and quality assessments are presented in Appendix 4, Table 25.

2.3.2.1 Question 8 – How effective is second-trimester transabdominal ultrasonography for velamentous cord insertion?

2.3.2.1.1 Description of the evidence

Seven publications including six unique cohorts of women reported the accuracy of TAS for diagnosing VCI.[38, 58-63] Hasegawa 2006[59] reported on a cohort overlapping with Hasegawa 2005.[60] Because the cohort in Hasegawa 2006 is larger and more recent,[59] Hasegawa 2005 is not considered further in this evidence synthesis.[60]

The quality and applicability of each identified study that used TAS as part of a routine screening protocol is shown in Table 4 below.

Table 4. Summary of QUADAS-2 assessments for VCI screening studies

Study reference	Di Salvo 1998[58]	Hasegawa 2006[59]	Nomiyama 1998[38]	Pretorius 1996[61]	Sepulveda 2006[62]	Sepulveda 2003[63]
PARTICIPANT SELECTION						
**Risk of bias**	Unclear	High	Low	Low	Low	High
**Concern about applicability**	Low	Low	Low	Low	Low	Low
INDEX TESTS						
**Risk of bias**	High	High	Low	High	High	High
**Concern about applicability**	Low	Low	Low	Low	High	High
REFERENCE STANDARD						
**Risk of bias**	High	Unclear	High	Low	Unclear	Unclear
**Concern about applicability**	Low	Low	Low	Low	Low	Low
PARTICIPANT FLOW						
**Risk of bias**	High	High	High	High	High	High

Participant selection

Three studies prospectively enrolled consecutive samples of patients and were at low risk of bias.[38, 61, 62] In Di Salvo 1998 it was unclear whether a consecutive sample of participants was enrolled.[58] Hasegawa 2006 was retrospective, leading to high risk of bias,[59] and Sepulveda 2003 excluded pregnancies with inadequate amniotic fluid volume, who may have been harder to diagnose.[63]

Index test

All six studies used TAS as part of a routine screening protocol.[38, 58, 59, 61-63] However, the screening algorithm was not consistent between the studies. Only one study, Nomiyama 1998, reported using the same screening methods for all patients and can therefore be judged to be at

CONFIDENTIAL

low risk of bias in this domain.[38] In the other five studies, not all women received the same index test. One used colour Doppler TAS routinely,[63] three studies used colour Doppler imaging at the discretion of the sonologist,[58, 59, 62] and in one study the ultrasound facilities had colour Doppler capabilities but it is not clear whether colour Doppler was used routinely.[61] Two studies additionally used TVS if TAS did not provide sufficient information to determine the cord insertion site, although it was unclear what proportion of women required TVS for this reason.[62, 63]

Ultrasound techniques

Although all six studies used TAS, each study used a different overall test strategy:

- Di Salvo 1998 and Hasegawa 2006 both used grey-scale TAS, with colour Doppler imaging used at the discretion of the sonologist,[58] or in cases of suspected VCI[59]
- Nomiyama 1998 used colour Doppler TAS, with the sonographer instructed to take additional time to image the placental cord insertion and indicate whether it was normal, velamentous or not visualised. The sonographer looked for the cord insertion site for two minutes before stating that it was not seen. At third-trimester repeat testing, if the cord insertion site could not be visualised using TAS, colour Doppler TVS was used[38]
- Pretorius 1996 assessed cord insertion using TAS with colour Doppler capabilities, but it is not clear how often the colour Doppler capabilities were used[61]
- Sepulveda 2006 initially used TAS, with TVS and colour Doppler used if needed[62]
- Sepulveda 2003 used colour Doppler TAS routinely, with colour Doppler TVS used if insufficient image quality was obtained from TAS[63]

Ultrasound timing

The timing of screening varied between the six studies:

- In Di Salvo 1998, GA at time of study sonogram ranged from 13 to 39 weeks (mean 27 weeks)[58]
- In Hasegawa 2006, abnormal cord insertion (CI) was evaluated between 18 and 20 weeks of gestation[59]
- In Nomiyama 1998, cord insertion was evaluated between 18 and 20 weeks of gestation; the mothers of fetuses with VCI were requested to return for repeat sonograms at 30 and 36 weeks of gestation to evaluate growth and to determine whether the cord insertion had changed[38]
- Pretorius 1996 screened fetuses at greater than 15 weeks of development[61]
- Sepulveda 2006 performed screening at 11 to 14 weeks (so some tests were first trimester and some were second trimester), with confirmation for all positive and negative cases later in the second trimester. There were no different diagnoses on the second trimester scans[62]
- The median GA at screening was 23 weeks in Sepulveda 2003, with a range of 16 to 40, and 75% of women screened between 16 and 30 weeks[63]

Reference standard

The target condition as defined by the reference standard in each study was VCI, determined by postnatal examination, although not all women receiving the index test subsequently received postnatal examination in each study. Only one study reported that the postnatal diagnosis was performed by a pathologist blinded to the screening result,[61] so the remaining five studies had

CONFIDENTIAL

high or unclear risk of bias in this domain.[38, 58, 59, 62, 63] One prospective study reported that the location of placental cord insertion was obtained from medical records.

Participant flow

All studies were at high risk of bias in this domain due to the long interval between the index test and the reference standard. Four studies performed postnatal examinations for all participants.[38, 59, 62, 63] In Di Salvo 1998, postnatal examination of the placenta was performed only for participants with perinatal complications or in multiple gestation pregnancies.[58] Pretorius 1996 attempted to systematically review placentas, but many specimens were not sent for pathologic study by the obstetricians.[61]

Results

Measures of test accuracy that were reported in the four studies, or could be calculated from data presented in each publication, are shown in Table 5.

Table 5. Test accuracy measures in VCI screening studies

Measure	Di Salvo 1998[58]	Hasegawa 2006[59]	Nomiyama 1998[38]	Pretorius 1996[61]	Sepulveda 2006[62]	Sepulveda 2003[63]
**Sensitivity**	1/4 *(25%)*	25/40 (62.5%)	5/5 (100%)	*2/6 (33.3%)*	5/5 *(100%)*	*7/7 (100%)*
**Specificity**	*50/50 (100%)*	3,406/3,406 (100%)	580/581 (99.8%)	*122/122 (100%)*	*528/528 (100%)*	*824/825 (99.8%)*
**PPV**	1/1 *(100%)*	25/25 (100%)	5/6 (83%)	2/2 *(100%)*	5/5 *(100%)*	*7/8 (87.5%)*
**NPV**	*50/53 (94.3%)*	3,406/3,421 (99.6%)	580/580 (100%)	*122/126 (96.8%)*	*528/528 (100%)*	*824/824 (100%)*
**Accuracy**	*51/54 (94.4%)*	*3,431/3,446 (99.6%)*	*585/586 (99.8%)*	*124/128 (96.9%)*	533/533 *(100%)*	*831/832 (99.9%)*

Abbreviations: NPV, negative predictive value; PPV, positive predictive value
Values in *italics* were calculated by a reviewer

Sensitivity varied from 1/4 (25%)[58] to 100% in three studies,[38, 62, 63] with the largest and highest-quality study reporting a sensitivity of 25/40 (62.5%).[59] Of the three studies reporting 100% sensitivity, two used TVS when TAS was insufficient, and the sensitivity is only reported for the composite testing pathway;[62, 63] it is therefore not possible to ascertain the sensitivity of TAS alone in these two studies.

PPV was 100% in four studies,[58, 59, 61, 62] and the lowest reported PPV was 5/6 (83%).[38] Only two studies reported a specificity below 100% (580/581 [99.8%][38], 824/825 [99.8%][63]). Although the false positive rate appears to be very low, the wide variation in reported sensitivity is a concern and suggests a great deal of uncertainty in the ability of TAS to reliably detect VCI. Results from larger prospective studies would be required to confirm the diagnostic accuracy.

In addition to the above, there are two related issues with interpreting results from these studies. Firstly, some studies reported that although researchers were reliably able to detect an abnormal cord insertion, the accuracy of diagnosing the specific type of abnormality (marginal cord insertion [MCI] vs VCI) was lower. Secondly, ultrasound scans were typically carried out several weeks before delivery. It is therefore possible, for example, that some cases of MCI that were correctly diagnosed during pregnancy may have progressed to VCI before delivery. This could account for the lower sensitivity seen in these studies. A further quality issue is that not all studies performed routine postnatal examinations to identify false negatives, which could lead to an overestimate of sensitivity.

CONFIDENTIAL

2.3.2.2 Evidence summary

The six studies reporting the performance of second-trimester TAS for diagnosing VCI showed consistently high specificity (>99.8% in all studies) and PPV (100% in four studies, and >83% in all studies). There was considerable variation in sensitivity between studies, with the largest and highest-quality study reporting a low sensitivity of 62.5%. It is not clear whether the variation in sensitivity is due to genuine false negative results from screening, or the progression of MCI to VCI during pregnancy in some cases.

Analysis of evidence relevant to criterion 4 – Screening tests for velamentous cord insertion

Quantity: Although a reasonable number of studies were identified, the total number of cases of VCI was low, which limits the robustness of any conclusions that can be drawn.

Quality: The quality of included studies was low because the index tests were inconsistent and not uniformly applied. Additionally, not all studies performed routine postnatal examinations to identify false negatives. Further quality concerns related to distinguishing between MCI and VCI, and the interval between screening and delivery. Some cases of MCI correctly diagnosed (and therefore recorded as screen-negative for VCI) during pregnancy could have progressed to VCI before delivery.

Applicability: All of the studies used second-trimester TAS, but some also performed screening in the first or third trimester, or used TVS as a confirmatory test in TAS-positive women.

Consistency: Although the false positive rate was consistently low, sensitivity varied considerably between the studies, which is a concern.

Conclusion

Overall, in studies identifying limited numbers of cases, screening for VCI using TAS appears to have good overall accuracy. However, this good accuracy is driven by high specificity. Therefore, well designed prospective studies in larger cohorts of women would be required to explore the sensitivity further.

Summary: Criterion 4 **not met** for VCI

2.3.3 Criteria 9 and 10 – Management pathways for velamentous cord insertion

Criteria 9 and 10 of the UK NSC Screening Criteria state that:

- 'There should be an effective intervention for patients identified through screening, with evidence that intervention at a pre-symptomatic phase leads to better outcomes for the screened individual compared with usual care. Evidence relating to wider benefits of screening, for example those relating to family members, should be taken into account where available. However, where there is no prospect of benefit for the individual screened then the screening programme shouldn't be further considered.'
- 'There should be agreed evidence based policies covering which individuals should be offered interventions and the appropriate intervention to be offered.'

The evidence relating to these criteria was addressed through one question, discussed in section 2.3.3.1

2.3.3.1 Question 9 - What is the most effective management pathway for women with screen-detected velamentous cord insertion?

2.3.3.1.1 Description of the evidence

No randomised control trials (RCTs) or cohort studies were identified that formally evaluated management pathways for VCI, which is unsurprising given the relatively low risks of adverse perinatal outcomes associated with VCI in the absence of VP or any other complications. This could be a concern if additional cases of VCI without VP were identified as a by-product of systematic screening for VP.

2.3.3.1.2 Evidence summary

No studies were identified that specifically addressed this question. However, the evidence presented in section 2.2.1 of this report about the substantially increased risk of VP among pregnancies with VCI suggests that testing for VP would be an appropriate course of action after a diagnosis of VCI.

Analysis of evidence relevant to criteria 9 and 10 – Management pathways for velamentous cord insertion
**Quantity:** Not applicable.<br>**Quality:** Not applicable.<br>**Applicability:** Not applicable.<br>**Consistency:** Not applicable.
Summary: Criterion 9 **not met**; criterion 10 **not met** for VCI

CONFIDENTIAL

3 Review summary

3.1 Conclusions and implications for policy

Do findings indicate whether screening should be recommended?

The previous review found that there was insufficient evidence for the implementation of universal screening of VP.

This review did not identify any evidence supporting a change in the previous recommendation. Overall, this review recommends that universal screening for VP should not be implemented because:

- There is not enough information about the incidence of VP in the UK
- VP can be found by ultrasound testing, but there is insufficient knowledge about the accuracy of the test
- CS would normally be recommended for cases of VP identified prenatally. However, there is no high-quality evidence on the optimum management pathway for cases of VP identified prenatally, such as inpatient vs outpatient treatment, or the timing of hospital admission or planned delivery

It has been suggested that ultrasound detection of VCI should be included in VP screening algorithms. However, this review found that:

- Most cases of VCI will not have VP. The reported incidence of VP among pregnancies with VCI is reported to be between 1% and 10%
- The accuracy of ultrasound for detecting VCI is unclear
- There are no established management pathways for VCI

3.1.1 Summary of findings

3.1.1.1 Epidemiology

Overall, the incidence of VP is likely to be between 0.02% and 0.04% in the general population, although UK-based evidence is lacking. The highest-quality evidence suggests that over 80% of VP cases can be found in high-risk groups. The previous UK NSC review included VCI, low-lying placenta in early pregnancy, succenturiate lobes, bilobed or multilobed placentas, multiple pregnancies, and pregnancies arising from IVF as risk factors for VP.[11] Unlike the previous review, this review did not find evidence of a significantly increased risk of VP in multiple pregnancies compared to singleton pregnancies. Evidence from multiple studies, including a meta-analysis, shows that for multiple pregnancies without additional risk factors there is no evidence of an increased risk of VP.

This review found that VCI is relatively common, occurring in 0.4% to 2.4% of singleton pregnancies and 5.9% to 40% of multiple pregnancies. The proportion of VCI cases with each risk factor was generally low. From the published literature it is unclear to what extent the risk factors overlap, so it was not possible to determine the proportion of VCI cases that occur in low-risk pregnancies.

3.1.1.2 Screening tests

This review identified a limited number of studies using second-trimester TAS as the primary screening test for VP. However, none reported key performance values, such as sensitivity and specificity, for TAS alone to screen for VP. Some studies routinely combined it with TVS, and others used TVS in cases of uncertainty or to confirm a suspected diagnosis of VP; as such, women with negative TAS results did not always receive a confirmatory test. Test accuracy characteristics were only reported for the composite testing pathways. Thus, the sensitivity of the primary screening test that would be used in a screening programme is uncertain. There were also no studies that confirmed VP diagnoses for all participants (both screen-positive and screen-negative) at birth.

The direction of the reported test values suggest that TAS-based screening may perform well. However, the limited volume and quality of the evidence means that this would require confirmation in well-designed prospective studies.

3.1.1.3 Management pathways

There were no studies comparing VP management pathways within the scope of this review. Furthermore, there is no evidence for using any particular management strategies in the general cohort of women with screen detected VCI. This would be a concern if additional cases of VCI without VP were identified as a by-product of a screening programme.

3.1.2 Are there gaps in the evidence highlighted by the review?

3.1.2.1 Epidemiology

For VP the current evidence base allows only tentative conclusions to be drawn about the epidemiology of VP with regard to overall incidence, incidence associated with risk factors, proportion of cases resolving by late pregnancy, and risk of adverse perinatal outcomes.

There were no studies reporting on VCI incidence in the UK, although studies on comparable cohorts were identified. As with VP, the current evidence base allows only tentative conclusions to be drawn about the epidemiology of VCI including overall incidence, incidence associated with several risk factors, and the risk of adverse perinatal outcomes. The value of including VCI in a VP screening algorithm was not explored in the studies.

3.1.2.2 Screening tests

This review did not identify any prospective cohort studies performing TAS routinely in all women to screen for VP and then confirming the presence or absence of VP at delivery in all women in the cohort. The quality of the reported estimates of diagnostic accuracy is therefore questionable.

In particular, there is no robust evidence for the sensitivity of TAS screening. However, the absence of a gold-standard reference test for diagnosing VP postnatally would limit any attempt to determine the diagnostic accuracy more precisely.

In relation to studies reporting on the performance of ultrasound detection of VCI, similar issues of volume and quality as those in the VP studies were encountered. These are unlikely to be resolved without well designed, large scale, prospective studies.

CONFIDENTIAL

3.1.2.3 Management pathways

Although it appears that perinatal outcomes in cases of VP diagnosed prenatally are substantially better than outcomes in undiagnosed cases,[29] this review found substantial evidence gaps around the most effective management pathways after diagnosis. In particular, this review did not identify any evidence on the optimal timing of elective CS, optimal timing of third-trimester follow-up scans, or third-trimester interventions such as the use of corticosteroids to promote lung development in infants delivered pre-term among VP pregnancies. However, the practical difficulties of conducting interventional studies on the management of VP, and the problems of investigating management strategies in a rigorous manner in observational studies, mean that the generation of evidence in this area will be challenging.

This review did not identify any studies investigating management pathways for VCI. This absence of studies could be indicative of a clinical consensus that no specific management is needed in this context. However, management of the low to moderate risk associations that would be identified as a consequence of screening for VP would require consideration as part of any attempt to evaluate the currently proposed screening algorithms.

3.1.3 Is further work warranted?

Future updates to this evidence review should prioritise the identification of UK-based epidemiology data, prospective studies reporting the accuracy of TAS as the primary screening test for the detection of VP, and developments relating to the effective clinical management of VP and VCI. However, in the absence of large UK-based prospective studies, it is unlikely that the evidence gaps identified in the current review will be filled.

3.2 Limitations

3.2.1 Limitations of the available evidence

Evidence gaps are detailed in Section 3.1.2 above. As well as these gaps, limitations relating to the quality and applicability of some identified evidence are discussed above.

3.2.2 Limitations of the review methodology

This rapid review was conducted in line with the UK NSC requirements for evidence summaries, as described at https://www.gov.uk/government/publications/uk-nsc-evidence-review-process/appendix-f-requirements-for-uk-nsc-evidence-summaries. The methodology was similar to a systematic review. It included searches of multiple databases including MEDLINE, Embase and three databases from the Cochrane Library. Database search terms were very broad, including terms for the disease area only, and were not restricted by study design, interventions and comparators, or publication date.

However, as this was a rapid review, some methodological compromises were made. Limitations of the review are discussed in detail below.

3.2.2.1 Included publication types

This review only included peer-reviewed journal publications, and excluded any literature that was not peer-reviewed such as congress presentations and government reports. This may have led to the exclusion of relevant evidence that has only been published in non-peer-reviewed

CONFIDENTIAL

formats. However, this is an accepted methodological adjustment for a rapid review, and is unlikely to miss any pivotal studies, which would likely be published in peer-reviewed journals.

3.2.2.2 Included study designs

Interventional and observational study designs such as RCTs, non-randomised trials, cohort studies, database studies and case-control studies were eligible for inclusion, as well as systematic reviews and meta-analyses of relevant study types. However, case studies were excluded.

This is standard accepted methodology for both systematic reviews and rapid reviews. However, for the specific conditions considered in this review, there is a high volume of case studies giving clinical insight about management strategies, and a lack of more robust study designs. Including these could have provided further information about how to manage VP and VCI after diagnosis. However, case studies are prone to bias and cannot be used to draw general conclusions about the effectiveness of strategies. It would be preferable to use published guidelines for management strategies, which take into account the breadth of clinical experience in this area rather than using individual case studies to inform management strategies.

Additionally, only studies published in English were included. Given that this review was focusing on evidence relevant to the UK setting, this limitation should not have led to the exclusion of any pivotal studies.

3.2.2.3 Date limits

Searches were run without date limits, and studies were initially considered for inclusion regardless of when they were conducted or published. However, given that a high volume of studies reporting epidemiology outcomes were identified, it was necessary to reduce the number of studies selected for extraction. Studies completed after 2000 and 2006 were ultimately included for VP and VCI respectively. Although some evidence from older studies has therefore been excluded from the evidence synthesis, there is evidence that rates of VP have changed over time.[14] The underlying risk factors for VP and VCI, particularly IVF, have also changed over time.[12, 13] The most recent estimates are therefore likely to provide the most relevant estimates of current epidemiology.

3.2.2.4 Review methodology

Articles were reviewed by a single reviewer in the first instance. A second reviewer examined all included articles, 10% of excluded articles, and any articles where there was uncertainty about inclusion. Although a fully systematic review would require all articles to be reviewed by both reviewers, this pragmatic strategy should have ensured that any articles where the eligibility was unclear were reviewed twice.

3.2.2.5 Articles not freely available

Searches for full-text articles were carried out at Cambridge University Library. Some articles were not freely available at this library. One article (Francois 2003[33]) was included in the evidence synthesis on the basis of the abstract alone, but for the remainder of the articles it was judged that they would not contain any additional pivotal data from relevant populations that would affect the conclusions of this review.

CONFIDENTIAL

4 Appendices

Appendix 1 – Search strategy

Electronic databases

The search strategy included searches of the databases shown in Table 6.

Table 6. Summary of electronic database searches and dates

Database	Platform	Searched on date	Date range of search
MEDLINE, MEDLINE In-Process, MEDLINE Daily, Epub Ahead of Print	Ovid SP	5th July 2016	1946 to Present
Embase	Ovid SP	5th July 2016	1974 to 1st July 2016
The Cochrane Library, including:<br>• Cochrane Database of Systematic Reviews (CDSR)<br>• Cochrane Central Register of Controlled Trials (CENTRAL)<br>• Database of Abstracts of Reviews of Effects (DARE)	Wiley Online	5th July 2016	CDSR: Issue 7 of 12, July 2016<br>CENTRAL: Issue 6 of 12, June 2016<br>DARE: Issue 2 of 4, April 2015 (no longer updated)

Search terms

Search terms included combinations of free text and subject headings (Medical Subject Headings [MeSH] for MEDLINE, and Emtree terms for Embase) relating to VP and VCI. Search terms for the databases searched through Ovid SP are shown in Table 7, and search terms for the Cochrane Library databases are shown in Table 8.

Table 7. Search strategy for MEDLINE, MEDLINE In-Process, MEDLINE Daily, Epub Ahead of Print and Embase (searched simultaneously via Ovid SP)

Term group	#	Search terms	Results (05/07/2016)
**VP and VCI**	1	Exp Vasa Previa/	308
	2	(vasa previa or vasa praevia).tw	541
	3	((velamentous or marginal) adj3 insertion).tw.	560
	4	Or/1-3	1018
**Date limit**	5	Remove duplicates from 4	652

Table 8. Search strategy for the Cochrane Library Databases (Searched via the Wiley Online platform)

Term group	#	Search terms	Results (05/07/2016)
**VP and VCI**	#1	[mh "vasa previa"]	1
	#2	"vasa previa" or "vasa praevia"	7
	#3	(velamentous or marginal) near /3 insertion	5
	#4	#1 or #2 or #3	12
**Data and database limit**	#5	#4<br>In Cochrane Reviews (Reviews only), Other Reviews and Trials	11

Results were imported into EndNote and de-duplicated.

CONFIDENTIAL

CONFIDENTIAL

Appendix 2 – Study selection

Review process

The following review process was followed:

- Each abstract was reviewed against the inclusion/exclusion criteria by one reviewer. Where the applicability of the inclusion criteria was unclear, the article was included at this stage in order to ensure that all potentially relevant studies were captured. A second independent reviewer provided input in cases of uncertainty, and validated 20% of the first reviewer's screening decisions. Any disagreements were resolved by discussion until a consensus was met.
- Full-text articles required for the full-text review stage were acquired.
- Each full-text article was reviewed against the inclusion/exclusion criteria by one reviewer, who determined whether the article was relevant to one or more of the review questions. A second independent reviewer provided input in cases of uncertainty, and validated 20% of the first reviewer's screening decisions. Any disagreements were resolved by discussion until a consensus was met.

Eligibility criteria

Eligibility criteria for each question are presented in Table 9 to Table 11 below. At the full-text review stage, publications were labelled as "relevant" or "not relevant" to each of the questions of interest. Questions were considered separately for [Condition 1] and [Condition 2]; if a publication reported outcomes relevant to multiple questions and/or both conditions then it was included in all relevant sections.

For all topics, systematic reviews and meta-analyses were considered for inclusion in this review. If the scope of a systematic review or meta-analysis was very closely aligned to one of the questions in this review, it was included in this review in its own right. However, if the scope was not closely aligned to one of the questions in this review but some of the included articles were of interest, the reference list of the systematic review or meta-analysis was hand searched. Any primary research articles that were identified as being relevant to this review were then included.

Table 9. Eligibility criteria for publications relating to epidemiology

Domain	Inclusion criteria		Exclusion criteria
**Population**	Pregnant women with a diagnosis of VP or VCI		Studies that did not include pregnant women with a diagnosis of VP or VCI, or that considered this population cohort but did not present outcomes for them separately to outcomes for irrelevant cohorts.
**Intervention**	Any or none		-
**Comparator**	Any or none		-
**Outcomes**	VP<br>• Prevalence or incidence<br>• Percentage of cases identified in the second trimester that	VCI<br>• Prevalence or incidence<br>• Risk of adverse perinatal outcomes, including but not restricted to:	Outcomes not deemed of relevance to this topic, although a broad approach was taken with regard to "relevance".

Domain	Inclusion criteria		Exclusion criteria
	resolve by late pregnancy<br>• Risk of adverse perinatal outcomes, including but not restricted to:<br>• Abnormal intrapartum fetal heart rate patterns<br>• Admission to neonatal intensive care unit<br>• Fetal growth restriction<br>• Low Apgar scores at 1 and 5 minutes<br>• Low birth weight<br>• Neonatal and fetal deaths<br>• Placental abruption<br>• Pre-term delivery (including emergency CS)	• Admission to neonatal intensive care unit<br>• Fetal growth restriction<br>• Low Apgar scores at 1 and 5 minutes<br>• Low birth weight<br>• Neonatal and fetal deaths<br>• Placental abruption<br>• Pre-eclampsia<br>• Pre-term delivery (including emergency CS)	
**Study design and publication type**	Peer-reviewed evidence derived from the following types of study:<br>• Systematic reviews and meta-analyses<br>• Observational studies<br>• Cross-sectional studies<br><br>Case studies were tagged during abstract review but were not included for full-text review in the first instance. It was planned that case studies would be re-visited at a later stage of the review if any evidence gaps were identified for which they may have provided additional relevant evidence		Other study designs/publication types.<br><br>Alternatively, RCTs or interventional non-RCTs that were unlikely to report relevant outcomes.<br><br>Conference abstracts or other publication types that have not been peer-reviewed.
**Language**	English language		Non-English language
**Date**	No date limit pre-specified, but only studies completed in or after 2000 (for VP) or 2006 (for VCI) were included in the evidence synthesis		No date limit pre-specified, but studies completed before 2000 (for VP) or 2006 (for VCI) were excluded from the evidence synthesis

Table 10. Eligibility criteria for publications relating to performance of screening methods

Domain	Inclusion criteria	Exclusion criteria
**Population**	Pregnant women at risk of VP or VCI	Studies that did not include pregnant women with a diagnosis of VP or VCI, or that considered this population cohort but did not present outcomes for them separately to outcomes for irrelevant cohorts
**Intervention**	• TAS ± colour Doppler<br>• Transvaginal ultrasound ± colour Doppler<br>• Other detection methods	-
**Comparator**	As listed in “Interventions”	-

CONFIDENTIAL

Domain	Inclusion criteria	Exclusion criteria
**Outcomes**	Outcomes relating to clinical performance measures:<br>• Sensitivity<br>• Specificity<br>• False positive rate<br>• False negative rate<br>• Positive predictive value<br>• Negative predictive value	Outcomes not relating to clinical performance measures for VP or VCI detection methods.
**Study design and publication type**	Peer-reviewed evidence derived from the following types of study:<br>• Systematic reviews and meta-analyses<br>• Randomised controlled trials (RCTs)<br>• Interventional non-RCTs<br>• Prospective cohort studies<br>• Retrospective cohort studies<br>• Case control studies<br>• Cross-sectional studies<br><br>Case studies were tagged during abstract review but were not included for full-text review in the first instance. It was planned that case studies would be re-visited at a later stage of the review if any evidence gaps were identified for which they may have provided additional relevant evidence	Other study designs/publication types.<br><br>Conference abstracts or other publication types that have not been peer-reviewed.
**Language**	English language	Non-English language
**Date**	No date limit	-

Table 11. Eligibility criteria for publications relating to the effectiveness of management pathways

Domain	Inclusion criteria	Exclusion criteria
**Population**	Pregnant women with screen-detected VP, or VCI in the absence of VP, who have been the subject of a management strategy.	Studies that did not include pregnant women with a diagnosis of VP, or VCI in the absence of VP, who have been the subject of a management strategy. Alternatively, studies that considered one or both of these population cohorts but did not present outcomes for them separately to outcomes for irrelevant cohorts
**Intervention**	Any	-
**Comparator**	Any or none	-
**Outcomes**	Clinical outcomes relating to the effectiveness of management pathways for VP or VCI in the absence of VP, including but not limited to:<br><br><u>VP</u><br>• Abnormal intrapartum fetal heart rate patterns<br>• Admission to neonatal intensive care unit<br>• Fetal growth restriction<br>• Low Apgar scores at 1 and 5 minutes<br>• Low birth weight<br>• Neonatal and fetal deaths<br>• Placental abruption<br>• Pre-term delivery (including emergency	Outcomes not relating to the clinical effectiveness of management pathways for VP or VCI, although a broad approach was taken with regard to "relevance"

CONFIDENTIAL

Domain	Inclusion criteria	Exclusion criteria
	caesarean section)<br><br><u>VCI</u><br>• Admission to neonatal intensive care unit<br>• Fetal growth restriction<br>• Low Apgar scores at 1 and 5 minutes<br>• Low birth weight<br>• Neonatal and fetal deaths<br>• Placental abruption<br>• Pre-eclampsia<br>• Pre-term delivery (including emergency caesarean section)	
**Study design and publication type**	Peer-reviewed evidence derived from the following types of study:<br>• Systematic reviews and (network) meta-analyses<br>• Randomised controlled trials (RCTs)<br>• Interventional non-RCTs<br>• Prospective cohort studies<br>• Retrospective cohort studies<br>• Case control studies<br>• Cross-sectional studies<br><br>Case studies were tagged during abstract review but were not included for full-text review in the first instance. It was planned that case studies would be re-visited at a later stage of the review if any evidence gaps were identified for which they may have provided additional relevant evidence	Other study designs/publication types<br><br>Conference abstracts or other publication types that have not been peer-reviewed
**Language**	English language	Non-English language
**Date**	No date limit	-

Appraisal for quality and risk of bias

The following tools were pre-specified for assessing the quality and risk of bias of each study included in the review:

- **Epidemiology studies**: JBI Critical Appraisal Checklist for Studies Reporting Prevalence Data[64]
- **Prognostic studies**: Centre for Evidence Based Medicine Prognostic Studies Critical Appraisal Worksheet[65]
- **Diagnostic accuracy studies**: Quality Assessment of Diagnostic Accuracy Studies (QUADAS-2) tool[66]
- **RCTs**: Cochrane Collaboration's "Risk of Bias" Tool[67]
- **Interventional non-RCTs**: Downs and Black checklist[68]
- **Cohort studies**: Critical Appraisal Skills Programme (CASP) Cohort Study Checklist[69]
- **Case control studies**: Critical Appraisal Skills Programme (CASP) Case Control Checklist[70]

CONFIDENTIAL

Appendix 3 – Included and excluded studies

PRISMA flowchart

Figure 2 summarises the volume of publications included and excluded at each stage of the review. 75 publications were ultimately judged to be relevant to one or more review questions and were considered for extraction. After excluding some articles reporting only epidemiology data from before 2000 (for VP) or 2006 (for VCI), 56 articles were included in the evidence synthesis. Publications that were included or excluded after the review of full-text articles are detailed below.

Figure 2. Summary of publications included and excluded at each stage of the review

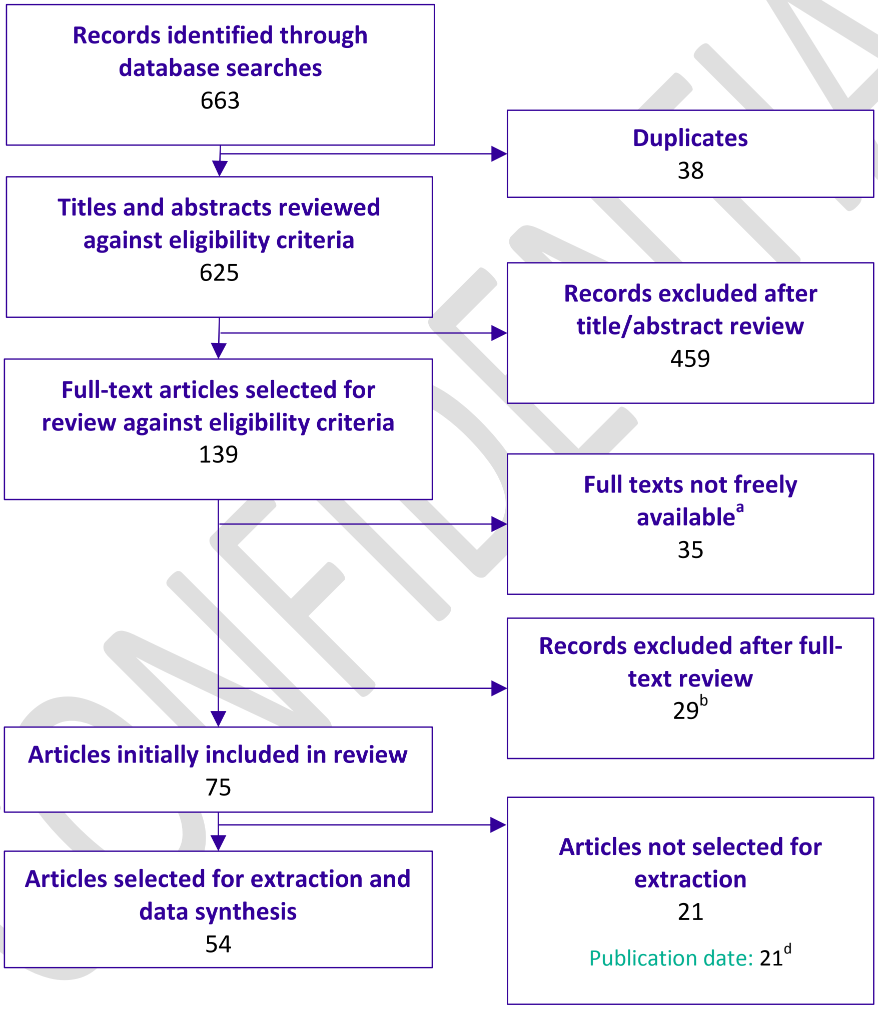

[a] Searches for full-text articles were carried out at Cambridge University Library. Some articles were not freely available at this library. One article (Francois 2003[33]) was included on the basis of the abstract alone, but for the remainder of the articles it was judged that they would not contain any additional pivotal data from relevant populations that would affect the conclusions of this review. [b] Eight articles were hand searched, but not included in their own right. [c] This number does not include one study (Schachter 2002[23]) from which epidemiology data was extracted for VP but not for VCI (due to different cut-off dates). [d] Two would also have been excluded for country if they were not already excluded for date.

CONFIDENTIAL

Publications included after review of full-text articles

The 56 publications relating to VP and VCI included after review of full-texts are summarised in Table 12 and Table 13 respectively.

Studies were prioritised for extraction and data synthesis. It was planned *a priori* that the following approach would be taken to prioritise studies for extraction:

- Systematic reviews and meta-analyses would be considered the highest quality of evidence if any were found. Following this, study designs would be prioritised for each question in the order listed in Table 9 to Table 11 respectively
- Studies relating to epidemiology would be prioritised if they considered a UK population, followed by studies from Western populations analogous to the UK

In addition, the following criteria were applied after assessing the overall volume of evidence identified in the review:

- For VP, epidemiology studies that were completed before 2000, regardless of publication date, were not extracted
- For VCI, epidemiology studies that were completed over 10 years before this review was conducted (studies that were completed in 2005 or earlier, regardless of publication date) were not extracted

Publications not selected for extraction and data synthesis are detailed in Table 14 below.

CONFIDENTIAL

Table 12. Summary of publications on VP included after review of full-text articles, and the question(s) each publication was relevant to VP

Study	Study design	Country	Years of study	Criterion 1 – Epidemiology	Criterion 4 – Screening	Criteria 9 and 10 – Management pathways
Ruiter 2016[7]	SLR	NA	NA	Q1	-	-
Baulies 2007[16]	Retrospective	Spain	2000 to 2005	Q1, Q3	-	(a)
Baumfeld 2016,[35] Evron 2015,[34] Rosenberg 2011[32]	Retrospective	Israel	1988 to 2012	Q1, Q3	-	-
Bronsteen 2013,[17] Lee 2000[37]	Retrospective	USA	1990 to 2010	Q1, Q2, Q3	Q4	(a)
Catanzarite 2001[2]	Prospective	USA	1991 to 1998	Q1	Q4	(a)
Donegan 2014[15]	Retrospective	UK	2010 to 2012	Q1	-	-
Francois 2003[33]	Case-control	USA	1991 to 2001	Q1	-	-
Hasegawa 2015,[18] Hasegawa 2010[24]	Retrospective	Japan	2005 to 2013	Q1, Q3	-	(b)
Hasegawa 2006[19]	Prospective	Japan	2003 to 2005	Q1	-	-
Heller 2014[25]	Retrospective	USA	2007 to 2011	Q1, Q3	-	-
Huerta-Enochian 2001[71]	Retrospective	USA	1995 to 2000	Q1	-	-
Kanda 2011[20]	Retrospective	Japan	2002 to 2007	Q1, Q3	Q4	(a)
Kapoor 2014[31]	Retrospective	Australia	2009 to 2011	Q1	-	-
Nomiyama 1998[38]	Prospective	Japan	1993 to 1996	Q1	Q4	-
Oyelese 2004[29]	Retrospective	UK, USA, Israel	1991 to 2003	Q1, Q3	-	(b)
Rebarber 2014[6]	Retrospective	USA	2005 to 2012	Q1, Q2, Q3	-	(a)
Robinson 2012[22]	Prospective	Australia	2007 to 2008	Q1	-	-
Schachter 2002[23]	Retrospective	Israel	1987 to 2001	Q1, Q3	-	-
Smorgick 2010[14]	Retrospective	Israel	1998 to 2007	Q1, Q3	-	-
Suzuki 2015,[21] Suzuki 2010,[28] Suzuki 2008[30]	Retrospective	Japan	2000 to 2011	Q1, Q3	-	-
Swank 2016[26]	Retrospective	USA	2000 to 2012	Q1, Q2, Q3	-	-

[a] Management pathways reported descriptively but not analytically
[b] Studies of interest, but not eligible for inclusion in this review
Studies in orange were completed before 2000 so epidemiology data has not been extracted, but other relevant data from the study has been extracted

Table 13. Summary of publications on VCI included after review of full-text articles, and the question(s) each publication was relevant to VCI

Study	Study design	Country	Years of study	Criterion 1 – Epidemiology	Criterion 4 – Screening	Criteria 9 and 10 – Management pathways
Baumfeld 2016[35]	Retrospective	Israel	1988 to 2012	Q6, Q7	-	-
Chu 2013[56]	Retrospective	USA	2010 to 2011	Q7	-	-
Costa-Castro 2013,[45] Lopriore 2007,[50] Lopriore 2012[49]	Retrospective	Portugal, the Netherlands	2002 to 2012	Q6, Q7	-	-
Costa-Castro 2016[53]	Retrospective	Portugal, the Netherlands	2005 to 2015	Q6, Q7	-	-
De Paepe 2010a,[51] De Paepe 2010b[52]	Prospective	USA	2001 to 2008	Q6	-	-
De Paepe 2011[4]	Prospective	USA	2009 to 2011	Q6	-	-
Di Salvo 1998[58]	Prospective	USA	1992 to 1995	Q6	Q8	-
Ebbing 2013,[1] Ebbing 2015[54]	Retrospective	Norway	1999 to 2011	Q6, Q7	-	-
Hack 2008[46]	Prospective	The Netherlands	1998 to 2007	Q6, Q7	-	-
Hack 2009[47]	Prospective	The Netherlands	1998 to 2008	Q6, Q7	-	-
Hasegawa 2005,[60] Hasegawa 2006[59]	Prospective, retrospective	Japan	2002 to 2004	Q6, Q7	Q8	-
Hasegawa 2009a,[41] Hasegawa 2009b[55]	Retrospective cohort	Japan	2005 to 2006	Q6, Q7	-	-
Hasegawa 2011[40]	Retrospective cohort	Japan	2006 to 2009	Q6	-	-

CONFIDENTIAL

Study	Study design	Country	Years of study	Criterion 1 – Epidemiology	Criterion 4 – Screening	Criteria 9 and 10 – Management pathways
Kent 2011[48]	Prospective	Ireland	2007 to 2009	Q6, Q7	-	-
Lepais 2014[72]	Retrospective	France	2005 to 2009	Q6	-	-
McNamara 2014[42]	Retrospective	Canada	1978 to 2007	Q6, Q7	-	-
Nomiyama 1998[38]	Prospective	Japan	1993 to 1996	Q6	Q8	-
Pinar 2014[57]	Case-control	USA	2006 to 2008	Q6	-	-
Pretorius 1996[61]	Prospective	USA	1992 to 1993	Q6	Q8	-
Raisanen 2012[43]	Retrospective	Finland	2000 to 2011	Q6, Q7	-	-
Sepulveda 2006[62]	Prospective	Chile	NR	Q6	Q8	-
Sepulveda 2003[63]	Prospective	Chile	2001 to 2002	Q6	Q8	-
Suzuki 2015[21]	Prospective	Japan	2002 to 2011	Q6, Q7	-	-
Walker 2012[73]	Retrospective	Canada	2000 to 2010	Q6	-	-
Yerlikaya 2016[44]	Case-control	Austria	2003 to 2013	Q6, Q7	-	-

Table 14. Summary of publications included after review of full-text articles, but not selected for extraction

Study	Study design	Country	Years of study	Vasa praevia			Velamentous cord insertion			Reason for exclusion from extractions
				Criterion 1 – Epidemiology	Criterion 4 – Screening	Criteria 9 and 10 – Management pathways	Criterion 1 – Epidemiology	Criterion 4 – Screening	Criteria 9 and 10 – Management pathways	
Ananth 2005[74]	Retrospective	USA	1979 to 2001	-	-	-	Q6, Q7	-	-	Date
Bjoro 1983[75, 76]	Retrospective	Norway	1969 to 1981	-	-	-	Q6, Q7	-	-	Date
De Laat 2006[77]	Cross-sectional	The Netherlands	2002 to 2003	-	-	-	Q6	-	-	Date

Study	Study design	Country	Years of study	Vasa praevia: Criterion 1 – Epidemiology	Vasa praevia: Criterion 4 – Screening	Vasa praevia: Criteria 9 and 10 – Management pathways	Velamentous cord insertion: Criterion 1 – Epidemiology	Velamentous cord insertion: Criterion 4 – Screening	Velamentous cord insertion: Criteria 9 and 10 – Management pathways	Reason for exclusion from extractions
Delbaere 2007[78]	Retrospective	Belgium	1985 to 2004	-	-	-	Q6	-	-	Date
Englert 1987[79]	Prospective	Belgium, Austria	1984 to 1987	-	-	-	Q6	-	-	Date
Feldman 2002[80]	Retrospective	USA	1995 to 1999	-	-	-	Q6, Q7	-	-	Date
Hasegawa 2006[19]	Prospective	Japan	2003 to 2005	-	-	-	Q6	-	-	Date
Paavonen 1984[81]	Retrospective	Finland	1980 to 1983	Q1	-	-	Q6	-	-	Date
Nakajima 2011[82]	Prospective	Japan	1993 to 1996	-	-	-	Q6	-	-	Date
Otsubo 1999[83]	Prospective	Japan	Mar to Sep 1997	-	-	-	Q6	-	-	Date
Fries 1993[84]	Retrospective	USA	1984 to 1992	-	-	-	Q6, Q7	-	-	Date
Heinonen 1996[85]	Retrospective	Finland	1991 to 1993	-	-	-	Q6	-	-	Date
Heinonen 1996[86]	Retrospective	Finland	1989 to 1993	-	-	-	Q6, Q7	-	-	Date
Lauslahti 1979[87]	Prospective	Finland	1973	-	-	-	Q6	-	-	Date
Nordenvall 1988[88]	Prospective	Sweden	Feb to Mar 1983	-	-	-	Q6	-	-	Date
Quek 1972[89]	NR	Singapore	NR, published 1972	Q1	-	-	-	-	-	Date, Country
Salafia 2010,[90] Yampolsky 2009[91]	Prospective	USA	2002 to 2004	-	-	-	Q6	-	-	Date
Sato 2006[92]	Retrospective	USA	1990 to 2000	-	-	-	Q6	-	-	Date
Schachter 2002[23]	Retrospective	Israel	1987 to 2001	Q1[a]	-	-	Q6	-	-	Date
Tang 1990[93]	Retrospective	China	1985 to 1988	-	-	-	Q6	-	-	Date, Country

[a] Schachter 2002 was extracted for VP epidemiology because the date cut-off was different for VP and VCI

CONFIDENTIAL

Publications excluded after review of full-text articles

Of the 139 publications included after the review of titles and abstracts, 21 were ultimately judged not to be relevant to this review and a further 8 were excluded but their reference lists were hand searched. These publications, along with reasons for exclusion, are listed in Table 15.

Table 15. Publications excluded after review of full-text articles

Reference	Reason for exclusion
Antoniou EE, Derom C, Thiery E, Fowler T, Southwood TR, Zeegers MP. The Influence of genetic and environmental factors on the etiology of the human umbilical cord: The east Flanders prospective twin survey. Biology of Reproduction. 2011;85(1):137-43.	VCI data not reported separately
Bajoria R. Chorionic plate vascular anatomy determines the efficacy of amnioreduction therapy for twin-twin transfusion syndrome. Human Reproduction. 1998;13(6):1709-13	No relevant outcomes
Borges KJ, Hassan N, Hussain R, Akhtar MT. Effects of variation in umbilical artery resistive index on placental morphology and birth weight in pregnancy induced hypertension. Journal of Ayub Medical College, Abbottabad: JAMC. 2013;25(3-4):23-6.	Investigating MCI
Das SR, Kar P, Sahoo S, Panda SK, Choudhury S, Nayak SR, et al. Morphological study of placenta in pregnancy with hypertension in Western Odisha. International Journal of Pharmaceutical Sciences Review and Research. 2015;33(1):74-8	Investigating MCI
De Paepe ME, DeKoninck P, Friedman RM. Vascular distribution patterns in monochorionic twin placentas. Placenta. 2005;26(6):471-5	VCI data not reported separately
Groen RS, Trelles M, Caluwaerts S, Papillon-Smith J, Noor S, Qudsia B, et al. A cross-sectional study of indications for cesarean deliveries in Medecins Sans Frontieres facilities across 17 countries. International Journal of Gynecology and Obstetrics. 2015;129(3):231-5	VP data not reported separately
Hasegawa J, Higashi M, Takahashi S, Mimura T, Nakamura M, Matsuoka R, et al. Can ultrasonography of the placenta previa predict antenatal bleeding? Journal of clinical ultrasound: JCU. 2011;39(8):458-62.	VCI data not reported separately
He M, Curran P, Raker C, Martin S, Larson L, Bourjeily G. Placental findings associated with maternal obesity at early pregnancy. Pathology Research and Practice. 2016;212(4):282-7.	Not relevant to VP or VCI
Jones KP, Wheater AW, Musgrave W. Simple test for bleeding from vasa praevia. [Erratum appears in Lancet 1988 Feb 6;1(8580):314]. Lancet. 1987;2(8573):1430-1.	Screening method not specific to VP or VCI
Kawakita T, Reddy UM, Landy HJ, Iqbal SN, Huang CC, Grantz KL. Indications for primary cesarean delivery relative to body mass index. Am J Obstet Gynecol. 2016; 19:19.	VP data not reported separately
Komuro H, Amagai T, Hori T, Hirai M, Matoba K, Watanabe M, et al. Placental vascular compromise in jejunoileal atresia. Journal of Pediatric Surgery. 2004;39(11):1701-5.	No relevant outcomes
Kramer MS, Dahhou M, Vallerand D, Liston R, Joseph KS. Risk Factors for Postpartum Hemorrhage: Can We Explain the Recent Temporal Increase? Journal of Obstetrics and Gynaecology Canada. 2011;33(8):810-9	Not relevant to VP or VCI
Lindqvist PG, Gren P. An easy-to-use method for detecting fetal hemoglobin-A test to identify bleeding from vasa previa. European Journal of Obstetrics Gynecology and Reproductive Biology. 2007;131(2):151-3.	Screening method not specific to VP or VCI
Liu CC, Pretorius DH, Scioscia AL, Hull AD. Sonographic prenatal diagnosis of marginal placental cord insertion: Clinical importance. Journal of Ultrasound in Medicine. 2002;21(6):627-32	Investigating MCI
Munden A, Butschek R, Tom WL, Marshall JS, Poeltler DM, Krohne SE, et al. Prospective study of infantile haemangiomas: Incidence, clinical characteristics and association with placental anomalies. British Journal of Dermatology. 2014;170(4):907-13.	Focus of study is haemangiomas, not VP/VCI

CONFIDENTIAL

Reference	Reason for exclusion
Nordenvall M, Ullberg U, Laurin J, Lingman G, Sandstedt B, Ulmsten U. Placental morphology in relation to umbilical artery blood velocity waveforms. European Journal of Obstetrics Gynecology and Reproductive Biology. 1991;40(3):179-90.	Not relevant to VP or VCI
Odunsi K, Bullough CHW, Henzel J, Polanska A. Evaluation of chemical tests for fetal bleeding from vasa previa. International Journal of Gynecology and Obstetrics. 1996;55(3):207-12.	Screening method not specific to VP
Proctor LK, Fitzgerald B, Whittle WL, Mokhtari N, Lee E, MacHin G, et al. Umbilical cord diameter percentile curves and their correlation to birth weight and placental pathology. Placenta. 2013;34(1):62-6	Not relevant to VP or VCI
Redline RW. Clinical and pathological umbilical cord abnormalities in fetal thrombotic vasculopathy. Human Pathology. 2004;35(12):1494-8.	Not relevant to VP or VCI
Suzuki S, Hiraizumi Y, Miyake H. Influence of umbilical cord abnormalities (velamentous/marginal cord insertion and nuchal cord) on the perinatal outcomes of the second twin after vaginal delivery of the first twin. Journal of Perinatal Medicine. 2011;39(6):745-8.	VCI data not reported separately
Uyanwah Akpom P, Fox H. The clinical significance of marginal and velamentous insertion of the cord. British Journal of Obstetrics and Gynaecology. 1977;84(12):941-3.	VCI data not reported separately
Hand-searched publications	
Cipriano LE, Barth Jr W, Zaric GS. The cost-effectiveness of targeted or universal screening for vasa praevia at 18-20 weeks of gestation in Ontario. BJOG: An International Journal of Obstetrics and Gynaecology 2010;117:1108-1118.	Article included for hand searches
Gagnon R, Morin L, Bly S, et al. Guidelines for the Management of Vasa Previa. Journal of Obstetrics and Gynaecology Canada 2009;31:748-753.	Article included for hand searches
Lijoi AF, Brady J. Vasa previa diagnosis and management. The Journal of the American Board of Family Practice / American Board of Family Practice 2003;16:543-548.	Article included for hand searches
Robinson BK, Grobman WA. Effectiveness of timing strategies for delivery of individuals with vasa previa. Obstetrics and Gynecology 2011;117:542-549.	Article included for hand searches
Ruiter L, Kok N, Limpens J, et al. Systematic review of accuracy of ultrasound in the diagnosis of vasa previa. Ultrasound in Obstetrics & Gynecology 2015;45:516-22	Article included for hand searches
Sinkey RG, Odibo AO, Dashe JS. #37: Diagnosis and management of vasa previa. American Journal of Obstetrics and Gynecology 2015;213:615-619.	Article included for hand searches
Vahanian SA, Lavery JA, Ananth CV, et al. Placental implantation abnormalities and risk of preterm delivery: A systematic review and metaanalysis. American Journal of Obstetrics and Gynecology 2015;Part S. 213:S78-S90.	Article included for hand searches
Vintzileos AM, Ananth CV, Smulian JC. Using ultrasound in the clinical management of placental implantation abnormalities. American Journal of Obstetrics and Gynecology 2015;Part S. 213:S70-S77.	Article included for hand searches

CONFIDENTIAL

Appendix 4 – Study-level synthesis of results

CONFIDENTIAL

Data extraction

Studies relating to the epidemiology, screening tests and management of VP are described in Table 16, Table 17 and Table 18 respectively. Studies relating to the epidemiology and screening tests for VCI are described in Table 19 and Table 20 respectively. There were no studies reporting on the management of VCI. Where a study reports data relevant to multiple topics or both conditions, the appropriate details have been included in each relevant table.

Studies relating to vasa praevia

Criterion 1 – Epidemiology of vasa praevia

Twenty-three publications reported data on the epidemiology of VP. Three publications reported on women from the same tertiary teaching hospital in Israel during overlapping time periods,[32, 34, 35] two publications reported on the same cohort from the USA during overlapping time periods,[17, 37] two publications reported on the same cohort from Japan during overlapping time periods,[18, 24] and three publications reported on another cohort from Japan during overlapping time periods.[21, 28, 30] There were therefore 17 unique cohorts of women reported. Where multiple publications reported similar outcomes for overlapping cohorts of women, relevant data has been extracted into Table 16 from each publication, but only the publication which followed up the cohort at the most recent time point has been used in the question-level synthesis in Section 2.2.1.1.

Table 16. Studies relevant to criterion 1 – Epidemiology of VP

Study reference, review questions	Study design	Population characteristics	Prevalence and incidence outcomes	Risk or incidence of perinatal outcomes associated with VP
**Baulies 2007**[16]<br><br>Relevant to Q1 and Q3	Design<br>Retrospective<br><br>Objective<br>Evaluate the role of ultrasound in prenatal diagnosis of VP and assess the	Data collection<br>Review of all term pregnancies in their hospital. The protocol for monitoring low-risk pregnancies in the hospital includes the assessment of cord insertion and the transvaginal measurement of cervical length at 20 to 22 weeks. If there is a suspicion of VP, a transvaginal ultrasound is repeated at 32 weeks.<br><br>VP was suspected when a tubular structure was	Overall incidence<br>• 9 of 12,063 cases of VP diagnosed prenatally, giving a reported incidence of 7.4/10,000 deliveries, 1/1,351 births, or 0.07%<br><br>Incidence associated with risk factors<br>• Incidence of VP in pregnancies resulting from IVF was 48/10,000 deliveries, 1/208 births (0.48%)	*Values for low Apgar score, low birthweight and pre-term delivery were calculated from Table 2, which reports outcomes for each pregnancy and/or infant individually*<br><br>Low Apgar score<br>All infants had Apgar scores 9, 10 at 1 minute and 5 minutes *(0%)*<br><br>Low birthweight<br>• Among 11 infants (9 VP pregnancies with 2

<table>
<tr><th>Study reference, review questions</th><th>Study design</th><th>Population characteristics</th><th>Prevalence and incidence outcomes</th><th>Risk or incidence of perinatal outcomes associated with VP</th></tr>
<tr>
<td></td>
<td>risk of VP associated with different causal factors

<u>Dates</u>
January 2000 to March 2005

<u>Country</u>
Spain

<u>Setting</u>
General hospital (Institut Universitari Dexeus, Barcelona), which manages a high percentage of IVF pregnancies (6.8%)</td>
<td>visualised over the cervix and internal cervical os; colour and pulsed Doppler were used to confirm the diagnosis.

<u>Sample size and demographics</u>
12,063 deliveries, of which:
• 824 (6.83%) were the result of IVF
• 466 (3.86%) were multiple pregnancies
• 336 (2.8%) women presented a second-trimester placenta praevia
• 25 (0.2%) had bilobate or succenturiate placenta with anatomopathological (AP) confirmation.

Mean age of VP mothers was 34 years (range 28 to 40)</td>
<td>• Incidence of VP in non-IVF pregnancies was 4.4/10,000 deliveries, 1/2,272 births (0.044%)

VP incidence in pregnancies with risk factors
<table>
<tr><th>Risk factor</th><th>VP, n (%) n=9</th><th>No VP, n (%) n=12,054</th><th>P value</th></tr>
<tr><td>IVF pregnancy</td><td>4 (44.4)</td><td>820 (6.8)</td><td><0.001</td></tr>
<tr><td>Multiple pregnancy</td><td>2 (22.2)</td><td>464 (3.8)</td><td><0.01</td></tr>
<tr><td>Bilobate or succenturiate placenta with AP confirmation</td><td>1 (11.1)[a]</td><td>24 (0.2)</td><td><0.001</td></tr>
<tr><td>Second-trimester placenta praevia</td><td>4 (44.4)</td><td>332 (2.8)</td><td><0.001</td></tr>
</table>
[a] Second-trimester ultrasound led to a diagnosis of 3 cases of bilobate placenta with VP. However, for the case-control study, data was only collected from the cases of bilobate or succenturiate placenta with subsequent AP confirmation, thus providing data for one case.

Calculated incidence by risk factors:
• Multiple pregnancy 2/466 (0.04%)
• Confirmed bilobate or succenturiate placenta 1/25 (4.0%)
• Second-trimester placenta praevia 4/336 (1.2%)

The absence of any placental anomaly, except for VCI, was only observed in one case. 77.77%</td>
<td>sets of twins), mean (SD) birthweight was 2675 (672) g
• 4/11 (36%) had low birthweight (<2500 g)

<u>Neonatal and fetal deaths</u>
None of the 62 deaths prior to and during delivery for the period in question were due to VP (0%)

<u>Pre-term delivery</u>
In 4/9 (44%) pregnancies, gestational age at delivery was pre-term (<37 weeks), including 3 emergency CS and one elective CS
Emergency CS was required in 4/9 (44%) VP pregnancies overall, including:
• 2/4 (50%) cases with VCI
• 1/2 (50%) cases with MCI
• 1/3 (33%) cases with normal cord insertion

Elective CS was carried out in the remaining 5/9 (55%) cases.

Gestational age at diagnosis, reason for ultrasound, clinical signs, pulmonary maturity, gestational age at delivery, sex and pH also reported</td>
</tr>
</table>

CONFIDENTIAL

<table>
<tr><th>Study reference, review questions</th><th>Study design</th><th>Population characteristics</th><th>Prevalence and incidence outcomes</th><th>Risk or incidence of perinatal outcomes associated with VP</th></tr>
<tr><td></td><td></td><td></td><td>of cases were diagnosed in asymptomatic women.

Multivariate regression was performed to identify independent risk factors for VP. Multiple pregnancy was not an independent risk factor. Odds ratios for the three independent risk factors are shown in the table below.

ORs calculated through multivariate regression
<table>
<tr><th>Risk factor</th><th>OR</th><th>95% CI</th></tr>
<tr><td>IVF</td><td>7.75</td><td>1.99 to 30.10</td></tr>
<tr><td>Bilobate or succenturiate placenta</td><td>22.11</td><td>1.92 to 253.84</td></tr>
<tr><td>Second-trimester placenta praevia</td><td>22.86</td><td>5.57 to 93.78</td></tr>
</table>
Correlation coefficients were also reported but not extracted.</td><td></td></tr>
<tr><td>Baumfeld 2016,[35] Evron 2015,[34] Rosenberg 2011[32]

(reporting on women from the same cohort)

Relevant to Q1 and Q3</td><td><u>Design</u>
Retrospective cohort

<u>Objective</u>
Each publication had a different objective: to investigate the fetal heart rate (FHR) patterns in pregnancies complicated with VP and VCI; to evaluate whether</td><td>Baumfeld 2016[35]

<u>Patient recruitment methodology</u>
All VP and VCI cases diagnosed at birth

<u>Sample size</u>
37 women with VP. Their subsequent pregnancies uncomplicated by VP served as controls, so this is effectively a case-control study

<u>Baseline characteristics</u>
In the VP group there were 36 singleton pregnancies and 1 multiple pregnancy, and in the control group there were 29 singleton pregnancies and 1 multiple pregnancy</td><td>Baumfeld 2016[35]

<u>Overall incidence</u>
NR

<u>Incidence associated with risk factors</u>
Among the VP pregnancies, 1/37 (2.7%) were multiple pregnancies

VP incidence in multiple and singleton pregnancies
<table>
<tr><th>Pregnancy type</th><th>VP, n (%) n=37</th><th>No VP, n (%) n=30</th><th>OR (95% CI)</th></tr>
<tr><td>Multiple</td><td>1 (2.7)</td><td>1 (3.3)</td><td rowspan="2">0.81 (0.05 to 13.44),</td></tr>
<tr><td>Singleton</td><td>36</td><td>29</td></tr>
</table></td><td><u>Abnormal intrapartum fetal heart rate patterns</u>
Values reported in Table 1 do not appear to include all pregnancies but the reason is unclear. Fetal monitor categories were as follows:
1st – baseline rate 110 to 160 bpm, moderate FHR variability (amplitude 6 to 25 bpm), no late or variable decelerations
3rd – absent baseline FHR variability and any of the following: recurrent late decelerations, recurrent variable decelerations, bradycardia; or, a sinusoidal pattern
2nd – all FHR patterns not classified as 1st or 3rd category
Fetal heart rate patterns in VP and non-VP pregnancies</td></tr>
</table>

CONFIDENTIAL

<table>
<tr><th>Study reference, review questions</th><th>Study design</th><th>Population characteristics</th><th>Prevalence and incidence outcomes</th><th>Risk or incidence of perinatal outcomes associated with VP</th></tr>
<tr>
<td></td>
<td>vanishing twin syndrome (VTS) is associated with adverse perinatal outcome; and to investigate risk factors and pregnancy outcome of women with placenta praevia

<u>Dates</u>
1988 to 2012 (Baumfeld 2016 and Evron 2015); 1988 to 2009 (Rosenberg 2011)

<u>Data sources</u>
Computerised perinatal database, which included information on maternal comorbidities, perinatal assessment, maternal and fetal complications. The obstetrical information is</td>
<td></td>
<td><table><tr><td></td><td>(97.3)</td><td>(96.7)</td><td>p=0.88</td></tr></table>
Note: N for no VP group was reported as 37 because subsequent pregnancies from the same women were used as controls, but from the data reported in Table 1 there appear to only be 30 pregnancies in the control group</td>
<td><table>
<tr><th></th><th>VP, n=33</th><th>No VP, n=29</th><th>P value</th></tr>
<tr><td colspan="4">Monitor category, n (%)</td></tr>
<tr><td>1st</td><td>12 (36.4)</td><td>14 (48.3)</td><td rowspan="3">0.34</td></tr>
<tr><td>2nd</td><td>21 (63.6)</td><td>15 (51.7)</td></tr>
<tr><td>3rd</td><td>0 (0.0)</td><td>0 (0.0)</td></tr>
<tr><td colspan="4">Baseline fetal heart rate, n (%)</td></tr>
<tr><td>Normal</td><td>24 (72.7)</td><td>27 (93.1)</td><td rowspan="4">0.04</td></tr>
<tr><td>Tachycardia</td><td>6 (18.2)</td><td>1 (3.4)</td></tr>
<tr><td>Bradycardia</td><td>3 (9.1)</td><td>1 (3.4)</td></tr>
<tr><td>Abnormal baseline</td><td>9 (27.3)</td><td>2 (6.9)</td></tr>
<tr><td colspan="4">Variability, n (%)</td></tr>
<tr><td>Absent</td><td>0</td><td>0</td><td rowspan="3">NR</td></tr>
<tr><td>Minimal</td><td>13 (39.4)</td><td>10 (34.5)</td></tr>
<tr><td>Moderate</td><td>20 (60.6)</td><td>19 (65.5)</td></tr>
<tr><td>Abnormal (absent and minimal)</td><td>13 (39.4)</td><td>10 (34.5)</td><td rowspan="2">0.69</td></tr>
<tr><td>Normal (moderate and marked)</td><td>20 (60.6)</td><td>19 (65.5)</td></tr>
<tr><td colspan="4">Accelerations, n (%)</td></tr>
<tr><td>Absent</td><td>16 (48.5)</td><td>7 (24.1)</td><td rowspan="2">0.05</td></tr>
<tr><td>Present</td><td>17 (51.5)</td><td>22 (75.9)</td></tr>
<tr><td colspan="4">Decelerations, n (%)</td></tr>
<tr><td>Absent</td><td>13 (39.4)</td><td>20 (69.0)</td><td rowspan="3">NR</td></tr>
<tr><td>Early</td><td>4 (12.1)</td><td>3 (10.3)</td></tr>
<tr><td>Late</td><td>0 (0)</td><td>1 (3.4)</td></tr>
</table></td>
</tr>
</table>

CONFIDENTIAL

<table>
<tr><th>Study reference, review questions</th><th>Study design</th><th>Population characteristics</th><th>Prevalence and incidence outcomes</th><th>Risk or incidence of perinatal outcomes associated with VP</th></tr>
<tr><td></td><td>entered immediately after birth by an obstetrician, and is routinely checked for inaccuracies. Only four skilled medical secretaries examine the information before entering it into the database.

<u>Country</u>
Israel

<u>Setting</u>
Soroka University Medical Center, a 1007 bed tertiary teaching hospital</td><td></td><td></td><td><table>
<tr><td>Variable</td><td>16 (48.5)</td><td>5 (17.2)</td><td></td></tr>
<tr><td>Present</td><td>20 (60.6)</td><td>9 (31.0)</td><td>0.02</td></tr>
<tr><td colspan="4">Sinusoidal pattern, n (%)</td></tr>
<tr><td>All</td><td>0 (0)</td><td>0 (0)</td><td>NR</td></tr>
</table>

<u>Low Apgar scores at 1 and 5 minutes</u>
Low scores were ≤7, normal scores were ≥8

Apgar scores at 1 minute in VP and non-VP pregnancies
<table>
<tr><th></th><th>Low, n (%)</th><th>Normal, n (%)</th><th>OR (95% CI)</th></tr>
<tr><td>VP, N=37</td><td>13 (36.1)</td><td>23 (63.9)</td><td rowspan="2">7.91 (1.62 to 38.71), p=0.01</td></tr>
<tr><td>No VP, N=30</td><td>2 (6.7)</td><td>28 (93.3)</td></tr>
</table>

Apgar scores at 5 minutes in VP and non-VP pregnancies
<table>
<tr><th></th><th>Low, n (%)</th><th>Normal, n (%)</th><th>OR (95% CI)</th></tr>
<tr><td>VP, N=37</td><td>8 (22.2)</td><td>28 (77.8)</td><td rowspan="2">Not calculated</td></tr>
<tr><td>No VP, N=30</td><td>0 (0)</td><td>30 (100)</td></tr>
</table>

<u>Neonatal and fetal deaths</u>
Total neonatal and fetal deaths included 3 stillbirths and 1 intrapartum death, all in the VP group

Fetal demise in VP and non-VP pregnancies
<table>
<tr><th></th><th>Death,</th><th>No</th><th>OR (95% CI)</th></tr>
</table></td></tr>
</table>

CONFIDENTIAL

Study reference, review questions	Study design	Population characteristics	Prevalence and incidence outcomes	Risk or incidence of perinatal outcomes associated with VP

	n (%)	death, n (%)	
VP, N=37	4 (10.8)	33 (89.2)	*Not calculated*
No VP, N=30	0 (0)	30 (100)	

<u>Pre-term delivery, including emergency CS</u>
Deliveries reported in Table 1 do not appear to include all pregnancies but the reason is unclear

Delivery route in VP and non-VP pregnancies

	Emergency CS, n (%)	Elective CS, n (%)	Vaginal, n (%)
VP, N=29	19 (65.5)	4 (13.8)	6 (20.7)
No VP, N=25	7 (28.0)	3 (12.0)	15 (60.0)

- *Calculated OR for emergency CS in VP vs non-VP pregnancies: 4.89 (95% CI 1.53 to 15.61, p=0.01)*
- *Calculated OR for elective CS in VP vs non-VP pregnancies: 1.17 (95% CI 0.24 to 5.83, p=0.85)*
- *Calculated OR for total CS in VP vs non-VP pregnancies: 5.75 (95% CI 1.73 to 19.15, p=0.004)*

CONFIDENTIAL

<table>
<tr><th>Study reference, review questions</th><th>Study design</th><th>Population characteristics</th><th>Prevalence and incidence outcomes</th><th>Risk or incidence of perinatal outcomes associated with VP</th></tr>
<tr><td></td><td></td><td>
Evron 2015[34]

<u>Patient recruitment methodology</u>
All women (except higher order pregnancies than twins and women lacking prenatal care) were included

<u>Sample size</u>
278 cases with vanishing twin syndrome (VTS), 252,994 singletons, 1801 first born dichorionic twins

<u>Baseline characteristics</u>
Baseline characteristics were reported separately for VTS, singleton and twin pregnancies

Baseline characteristics in VTS, singleton and twin pregnancies
<table>
<tr><th></th><th>VTS, N= 278</th><th>Singleton, N= 252,994</th><th>Twins, N= 1801</th></tr>
<tr><td>Maternal age (yr), mean (SD)</td><td>31.62 (5.737)</td><td>28.68 (5.817)</td><td>30.11 (5.555)</td></tr>
<tr><td colspan="4">Ethnicity (%)</td></tr>
<tr><td>Bedouins</td><td>37.4</td><td>47.2</td><td>46</td></tr>
<tr><td>Jews</td><td>62.6</td><td>52.8</td><td>54</td></tr>
<tr><td colspan="4">Parity (%)</td></tr>
<tr><td>1</td><td>36.3</td><td>24.6</td><td>32.4</td></tr>
<tr><td>2 to 4</td><td>45.7</td><td>51.6</td><td>45.5</td></tr>
<tr><td>5+</td><td>18</td><td>23.8</td><td>22.1</td></tr>
<tr><td colspan="4">Fertility treatment (%)</td></tr>
<tr><td>IVF</td><td>16.5</td><td>0.6</td><td>12.9</td></tr>
<tr><td>Ovulation</td><td>18</td><td>1.4</td><td>22.8</td></tr>
</table>
</td><td>
<u>Overall incidence</u>
NR

<u>Incidence associated with risk factors</u>

1.1% in VTS group
0.1% in singletons
0.4% in first-born dichorionic twins

</td><td>NR</td></tr>
</table>

CONFIDENTIAL

Study reference, review questions	Study design	Population characteristics	Prevalence and incidence outcomes	Risk or incidence of perinatal outcomes associated with VP
		<table><tr><td>induction</td><td></td><td></td><td></td></tr><tr><td>None</td><td>65.5</td><td>98.1</td><td>64.3</td></tr></table>		
		**Rosenberg 2011**[32]<br><br>Patient recruitment methodology<br>All singleton pregnancies. Pregnancies with multiple fetuses and pregnancies without adequate prenatal surveillance were excluded from the study<br><br>Sample size<br>184,705 with no placenta praevia and 771 with placenta praevia.<br><br>Placenta praevia included all singleton pregnancies with placental attachment totally or mostly in the lower uterine segment which covered completely or partially the internal os in the second and third trimester, as diagnosed by ultrasound.<br><br>Baseline characteristics<br><br>**Baseline characteristics in pregnancies with and without placenta praevia**<br><table><tr><th></th><th>Placenta praevia, n= 771</th><th>No placenta praevia, n= 184,705</th></tr><tr><td colspan="3">Maternal age (years)</td></tr></table>	Overall incidence<br>NR<br><br>Incidence associated with risk factors<br>• 4/771 (0.5%) in placenta praevia cases<br>• 0.1% in 184,705 non-placenta praevia cases<br><br>Reported OR 4.8 (95% CI 1.8 to 13.0, p<0.001) for placenta praevia vs non-placenta praevia cases	NR

CONFIDENTIAL

<table>
<tr><th>Study reference, review questions</th><th>Study design</th><th>Population characteristics</th><th>Prevalence and incidence outcomes</th><th>Risk or incidence of perinatal outcomes associated with VP</th></tr>
<tr><td></td><td></td><td>
<table>
<tr><td><20</td><td>1</td><td>4.1</td></tr>
<tr><td>20 to 29</td><td>32.5</td><td>55.8</td></tr>
<tr><td>30 to 34</td><td>29.0</td><td>23.6</td></tr>
<tr><td>35+</td><td>37.4</td><td>16.5</td></tr>
<tr><td colspan="3">Ethnicity (%)</td></tr>
<tr><td>Bedouins</td><td>35.3</td><td>46.1</td></tr>
<tr><td>Jews</td><td>64.7</td><td>53.9</td></tr>
<tr><td colspan="3">GA (weeks)</td></tr>
<tr><td><34</td><td>23.5</td><td>2.1</td></tr>
<tr><td>34 to 36</td><td>28.4</td><td>5.6</td></tr>
<tr><td>37 to 41</td><td>47.1</td><td>87.6</td></tr>
<tr><td>42+</td><td>1.0</td><td>4.7</td></tr>
<tr><td colspan="3">Other (%)</td></tr>
<tr><td>Infertility treatment</td><td>6.4</td><td>1.9</td></tr>
<tr><td>Prior CS</td><td>26.3</td><td>11.6</td></tr>
</table>
p<0.001 for all extracted characteristics
% who were smokers, who had habitual abortions and who had hypertensive disorders were reported but not extracted</td><td></td><td></td></tr>
<tr><td>Bronsteen 2013,[17] Lee 2000[37]

(reporting on women from the same cohort)

Relevant to Q1, Q2 and Q3</td><td><u>Design</u>
Retrospective

<u>Objective</u>
To review experience with diagnosis, clinical associations, and outcomes of vasa praevia in a single institution

<u>Dates</u>
January 1990 to</td><td><u>Patient recruitment methodology</u>
All cases where ultrasound examinations were performed at 15 weeks of gestation and later in which VP was reported, defined as a fetal vessel within the membranes of the lower uterus. Women with cases of VP diagnosed in the unit but who delivered elsewhere were excluded

<u>Sample size</u>
182,554 pregnancies with at least one ultrasound examination

Outcomes reported for 60 cases of VP, and incidence of placental or cord abnormalities for 58 cases</td><td><u>Overall incidence</u>
81 cases of VP were diagnosed through either prenatal ultrasound or placental evaluation after delivery
5 cases diagnosed by ultrasound had subsequent examinations that did not support the VP diagnosis
2 cases were excluded due to lack of ultrasound information
Therefore, 74 cases were confirmed, giving an incidence of 1 in 2,467 pregnancies (0.04%)

(Note: overall incidence was also reported in Lee 2000 as 18 cases of VP in 93,874 pregnancies [0.019%], but data from the larger and more recent cohort from Bronsteen 2013 are used in</td><td>Outcomes are reported for 60 cases of VP, including 7 sets of twins, so a total of 67 fetuses

<u>Admission to neonatal intensive care unit</u>
6/67 (9.0%) infants had hypovolemic shock at birth requiring intubation and neonatal intensive care unit admission

<u>Low Apgar scores at 1 and 5 minutes</u>
Among the smaller cohort from Lee 2000, no infants had low Apgar scores

<u>Low birth weight</u>
Among the smaller cohort from Lee 2000, mean (SD) birth weight was 2660 (660) g

<u>Neonatal and fetal deaths</u></td></tr>
</table>

CONFIDENTIAL

<table>
<tr><th>Study reference, review questions</th><th>Study design</th><th>Population characteristics</th><th>Prevalence and incidence outcomes</th><th>Risk or incidence of perinatal outcomes associated with VP</th></tr>
<tr>
<td></td>
<td>June 2010 (Lee 2000 reports cases from January 1991 to December 1998)

<u>Data sources</u>
Electronic files for all ultrasound scans and hospital discharge records

<u>Country</u>
USA

<u>Setting</u>
William Beaumont Hospital</td>
<td><u>Baseline characteristics</u>
None reported</td>
<td>this review)

<u>Resolution of VP</u>
5/79 (6.3%) cases diagnosed by ultrasound had subsequent ultrasound examinations and delivery information that did not support the VP diagnosis. This could represent true resolution of VP, or misdiagnosis.

(Note: Lee 2000 reports that 3/18 (16.7%) suspected cases had normal late third-trimester scans, but data from the larger and more recent cohort from Bronsteen 2013 are used in this review)

<u>Incidence associated with risk factors</u>
Among cases where delivery and placental pathology reports allowed for evaluation of the cord insertion site, 43/48 (89.6%) VP cases had VCI or MCI
Among cases who had an ultrasound in the study unit, the placenta was low-lying or overlying the cervix in 36/58 (62.1%) of VP cases
Overall, at least one of these risk factors (abnormal cord insertion or abnormal placental location) was present in 55/58 (94.8%) of VP cases</td>
<td>There were 2 neonatal deaths and 1 antenatal death, for a mortality rate of 3/67 (4.5%)

<u>Pre-term delivery, including emergency CS</u>
Pre-term delivery rate was reported on graph. All twin pregnancies and approximately 70% of singleton pregnancies were delivered before 37 weeks.

Emergency CS was required in:
7/60 cases (11.7%) overall
3/4 (75%) undiagnosed cases
4/56 (7%) prenatally diagnosed VP cases
Calculated OR for emergency CS in undiagnosed vs prenatally diagnosed VP cases: 39.0 (95% CI 3.26 to 466.27, p=0.004)

<u>Delivery by vessel location</u>
The definition of VP in this study included cases with vessels over the cervix as well as cases with low-lying vessels.

Delivery type by twin vs singleton pregnancy, and vessel over cervix vs low-lying vessel
<table>
<tr><th>Delivery Type</th><th>n (%)</th></tr>
<tr><td colspan="2">Twins with vessel over cervix (n=6)</td></tr>
<tr><td>Elective</td><td>3 (50)</td></tr>
<tr><td>Emergent</td><td>1 (16.7)</td></tr>
<tr><td>Urgent due to bleeding</td><td>2 (33.3)</td></tr>
<tr><td>Urgent due to labour or premature rupture of membranes</td><td>0 (0)</td></tr>
<tr><td colspan="2">Twins with low-lying vessel (n=1)</td></tr>
<tr><td>Elective</td><td>0 (0)</td></tr>
<tr><td>Emergent</td><td>1 (100)</td></tr>
<tr><td>Urgent due to bleeding</td><td>0 (0)</td></tr>
<tr><td>Urgent due to labour or</td><td>0 (0)</td></tr>
</table></td>
</tr>
</table>

CONFIDENTIAL

Study reference, review questions	Study design	Population characteristics	Prevalence and incidence outcomes	Risk or incidence of perinatal outcomes associated with VP
				premature rupture of membranes | **Singletons with vessel over cervix (n=36)**<br>Elective | 21 (58.3)<br>Emergent | 1 (2.8)<br>Urgent due to bleeding | 7 (19.4)<br>Urgent due to labour or premature rupture of membranes | 7 (19.4)<br>**Singletons with low-lying vessel (n=13)**<br>Elective | 7 (53.8)<br>Emergent | 1 (7.7)<br>Urgent due to bleeding | 1 (7.7)<br>Urgent due to labour or premature rupture of membranes | 4 (30.8)<br>Emergent delivery defined as delivery because of bleeding with abnormal fetal heart rate tracings. Urgent delivery defined as an unscheduled delivery for an acute problem (bleeding, labour, rupture of membranes) without concerning fetal heart rate tracings
**Donegan 2014**[15]<br><br>Relevant to Q1	<u>Design</u><br>Retrospective cohort<br><br><u>Objective</u><br>To examine the safety of pertussis vaccination in pregnancy<br><br><u>Dates</u><br>October 2012 to March 2013	<u>Patient recruitment methodology</u><br>Pregnant women were identified from the database through Read codes. A validated algorithm was used to identify pregnancy outcomes<br><br><u>Sample size</u><br>20,074 pregnant women with pertussis vaccine. 17,560 (87%) vaccinated pregnant women had ≥28 days' follow-up data after their vaccination record<br><br><u>Baseline characteristics</u><br>Median age 30 years (IQR 26 to 34) among the whole cohort and among the cohort with follow-	<u>Overall incidence</u><br>No cases of VP were reported (0%)	NA – no cases of VP

CONFIDENTIAL

Study reference, review questions	Study design	Population characteristics	Prevalence and incidence outcomes	Risk or incidence of perinatal outcomes associated with VP
	<u>Data sources</u><br>Data was taken from the Clinical Practice Research Datalink, which collates demographic, clinical, prescribing, test and referral data extracted from over 650 primary care general practice databases for more than 12.5 million women throughout the UK.<br><br>Adverse events were identified by using records of clinical diagnoses during pregnancy, with additional data from the matched child record identified through the mother-child linkage compiled by	up data		

CONFIDENTIAL

<table>
<tr><th>Study reference, review questions</th><th>Study design</th><th>Population characteristics</th><th>Prevalence and incidence outcomes</th><th>Risk or incidence of perinatal outcomes associated with VP</th></tr>
<tr><td></td><td>CPRD research staff. VP was a secondary event of interest in this study

Country
UK

Setting
The UK Clinical Practice Research Datalink</td><td></td><td></td><td></td></tr>
<tr><td>Francois 2003[33]

Full-text not available, data extracted from abstract

Relevant to Q1</td><td>Design
Retrospective case-control

Objective
To evaluate whether VP at delivery is associated with a history of second-trimester placenta praevia.

Dates
January 1991 to May 2001

Data sources
Cases were</td><td>Patient recruitment methodology
Each case was matched in a 1:4 ratio with controls based upon normal placentation at delivery, ultrasound documentation of mid-trimester placental location, maternal parity and gestational age at delivery

Sample size
13 cases of VP and 52 controls were identified

Baseline characteristics
NR in abstract</td><td>Overall incidence
As this was a case-control study, overall incidence was not reported

Incidence associated with risk factors

VP incidence by risk factors
<table>
<tr><th>Risk factor</th><th>VP, n (%) n=13</th><th>No VP, n (%) n=52</th><th>OR (95% CI)</th></tr>
<tr><td>Placenta praevia</td><td>9 (69.2)</td><td>2 (3.8)</td><td rowspan="2">56.3 (8.9 to 354.1)</td></tr>
<tr><td>No placenta praevia</td><td>4 (30.1)</td><td>50 (96.2)</td></tr>
<tr><td>Multiple pregnancy[a]</td><td>1 (7.7)</td><td>3 (23.1)</td><td rowspan="2">1.36 (0.13 to 14.27)</td></tr>
<tr><td>Singleton pregnancy[a]</td><td>12 (92.3)</td><td>49 (94.2)</td></tr>
</table>
[a] Taken from Ruiter 2016[7]</td><td>NR in abstract</td></tr>
</table>

CONFIDENTIAL

Study reference, review questions	Study design	Population characteristics	Prevalence and incidence outcomes	Risk or incidence of perinatal outcomes associated with VP
	identified by ICD-9 codes and confirmed by chart review. Placenta praevia was documented by mid-trimester ultrasonography, and vasa praevia was assessed at delivery<br><br><u>Country</u><br>USA<br><br><u>Setting</u><br>Good Samaritan Regional Medical Center			
**Hasegawa 2015,**[18] **Hasegawa 2010**[24]<br><br>(reporting on women from the same cohort)<br><br>Relevant to Q1, Q3	<u>Design</u><br>Retrospective<br><br><u>Objective</u><br>Hasegawa 2015: to review cases of vasa praevia in which the women were managed at the university hospital in order to clarify the clinical course	**Hasegawa 2015**<br><br><u>Patient recruitment methodology</u><br>All cases of VP managed at the hospital between January 2005 and October 2013 were identified<br><br><u>Sample size</u><br>21 women with VP were identified<br><br><u>Baseline characteristics</u><br>Median maternal age 31 (range 23 to 37) among women admitted to the hospital, and 36.5 (range 30 to 42) among women managed as outpatients	<u>Overall incidence</u><br>21/8176 (0.3%) of deliveries had VP	15 women with VP required admission to the hospital due to a low-lying placenta with bleeding, fetal growth restriction, uterine contractions, shortened uterine cervix, and/or abnormal heart rate tracing. 6 women were managed as outpatients until CS.<br><br><u>Admission to neonatal intensive care unit</u><br>• 11/15 (73%) of women admitted to the hospital<br>• 2/6 (33%) of women managed as outpatients<br>• *13/21 (61.9%) overall*<br><br><u>Fetal growth restriction</u><br>'Light for date' was reported:<br>• 5/15 (33%) of women admitted to the

CONFIDENTIAL

Study reference, review questions	Study design	Population characteristics	Prevalence and incidence outcomes	Risk or incidence of perinatal outcomes associated with VP
	and neonatal outcomes of the hospital's management strategy<br><br>Hasegawa 2010: to identify ultrasound findings indicative of prenatal VP<br><br><u>Dates</u><br>January 2005 to October 2013 (Hasegawa 2015), 2006 to 2009 (Hasegawa 2010)<br><br><u>Data sources</u><br>Medical records<br><br><u>Country</u><br>Japan<br><br><u>Setting</u><br>Showa University School of Medicine			hospital<br>• 0/6 (0%) of women managed as outpatients<br>• *5/21 (23.8%) overall*<br><br><u>Low Apgar scores at 1 and 5 minutes</u><br>Apgar scores at 1 minute:<br>• Median 8 (range 1 to 9) among 15 women admitted to the hospital<br>• Median 8 (range 8 to 9) among 6 women managed as outpatients<br><br>Apgar scores at 5 minutes:<br>• Median 9 (range 6 to 10) among 15 women admitted to the hospital<br>• Median 9 (range 8 to 10) among 6 women managed as outpatients<br><br><u>Low birth weight</u><br>• Median 2017 g (range 1211 to 3359) among 15 women admitted to the hospital<br>• Median 2450 g (range 2181 to 2875) among 6 women managed as outpatients<br><br><u>Pre-term delivery, including emergency CS</u><br>Gestational age at delivery:<br>• Median 35 weeks 4 days (range 27 to 36 weeks) among 15 women admitted to the hospital<br>• Median 36 weeks 2 days (range 35 to 36 weeks) among 6 women managed as outpatients<br><br>Emergency CS:<br>• 4/15 (27%) of women admitted to the hospital (2 for abnormal heart rate tracing, 2 for premature membrane rupture)<br>• 1/6 (17%) of women managed as outpatients (for pre-eclampsia)

CONFIDENTIAL

<table>
<tr><th>Study reference, review questions</th><th>Study design</th><th>Population characteristics</th><th>Prevalence and incidence outcomes</th><th>Risk or incidence of perinatal outcomes associated with VP</th></tr>
<tr><td rowspan="2"></td><td rowspan="2"></td><td></td><td></td><td>
5/21 (23.8%) overall
Rescheduled CS ahead of initially planned operation:
3/15 (20%) of women admitted to the hospital (2 for uterine contractions, 1 for fetal growth arrest)
0/6 (0%) of women managed as outpatients
3/21 (14.3%) overall
</td></tr>
<tr><td>
Hasegawa 2010

<u>Patient recruitment methodology</u>
Consecutive women who delivered at Showa University Hospital between 2006 and 2009 were included

<u>Sample size</u>
4,532 pregnancies (4,375 singletons, 154 twins, 3 triplets), giving 4,692 placentas

<u>Baseline characteristics</u>
Mean (SD) maternal age:
34.0 (4.0) years among VP cases
32.5 (4.7) years among non-VP cases
</td><td>
<u>Overall incidence</u>
10/4,532 cases, giving an incidence of 0.221%
6/10 (60%) cases were type I, of which 1/6 (17%) had low-lying placenta
4/10 (40%) cases were type II, of which 3/4 (75%) had low-lying placenta
<u>Incidence associated with risk factors</u>

VP incidence by risk factors
<table>
<tr><th>Risk factor</th><th>VP, n (%) n= 10</th><th>No VP, n (%) n= 4,682</th><th>OR (95% CI)</th></tr>
<tr><td>Multiple pregnancy</td><td>0</td><td>157</td><td rowspan="2">1.32 (0.08 to 22.62)[a]</td></tr>
<tr><td>Singletons</td><td>10</td><td>4,365</td></tr>
<tr><td>VCI</td><td>9 (90)</td><td>75 (1.6)</td><td>552.8 (69.2 to 4419)</td></tr>
<tr><td>Placenta praevia or low-lying placenta</td><td>4 (40)</td><td>109 (2.3)</td><td>28.0 (7.8 to 100.5)</td></tr>
<tr><td>Abnormal placental forms[b]</td><td>4 (40)</td><td>90 (1.9)</td><td>34.0 (9.4 to 122.6)</td></tr>
</table>
</td><td>
<u>Fetal growth restriction</u>
50% of VP cases and 9.8% of non-VP cases
Crude OR: 9.2 (95% CI 2.7 to 32.1)
<u>Low Apgar scores at 1 and 5 minutes</u>
Apgar scores at 1 minute:
Median 8 (range 1 to 8) among 10 VP cases
Median 9 (range 0 to 10) among 4682 non-VP cases
Apgar scores at 5 minutes:
Median 9 (range 6 to 10) among 10 VP cases
Median 9 (range 0 to 10) among 4682 non-VP cases
<u>Low birth weight</u>
Mean (SD) birth weight:
1942 (481) g among 10 VP cases
2837 (584) g among 4682 non-VP cases
<u>Neonatal and fetal deaths</u>
There were no cases of intrauterine fetal death associated with VP

<u>Pre-term delivery, including emergency CS</u>
From Table 1, 7/10 (70%) cases were delivered
</td></tr>
</table>

CONFIDENTIAL

<table>
<tr><th>Study reference, review questions</th><th>Study design</th><th>Population characteristics</th><th>Prevalence and incidence outcomes</th><th>Risk or incidence of perinatal outcomes associated with VP</th></tr>
<tr><td></td><td></td><td></td><td><table><tr><td>Low cord insertion</td><td>9 (90)</td><td>17 (0.4)</td><td>2469.7 (296 to 20,579)</td></tr></table>[a] Reported in Ruiter 2016 [7]
[b] Multilobed, succenturiate and accessory
Note: calculated numbers of women with no VP are based on the reported percentage and have been checked against the OR to find the precise value

Calculated incidence for each risk factor:
• Multiple pregnancy 0/157 (0%)
• VCI 9/84 (10.7%)
• Placenta praevia 4/113 (3.5%)
• Abnormal placental forms 4/94 (4.3%)
• Low cord insertion 9/26 (34.6%)

After multivariate logistic regression analysis, adjusted OR for VCI was 65.1 (95% CI 5.8 to 733, p=0.001) and adjusted OR for low cord insertion was 344.7 (95% CI 31 to 3838, p<0.001)</td><td>pre-term (gestational age <37 weeks), and the remaining 3/10 deliveries were at 37 weeks</td></tr>
<tr><td>Hasegawa 2006[19]

Relevant to Q1</td><td><u>Design</u>
Prospective

<u>Objective</u>
Assess the feasibility of detecting the cord insertion site during the late first trimester, and to investigate the possible association between perinatal</td><td><u>Patient recruitment methodology</u>
Consecutive enrolment of pregnant women scheduled to deliver at the hospital

<u>Sample size</u>
318

<u>Baseline characteristics</u>
Baseline characteristics for women with low cord insertion and normal cord insertion are shown below.

Baseline characteristics by sonographic diagnosis of cord insertion at 9 to 11 weeks<table><tr><th>Characteristic</th><th>Low cord insertion,</th><th>Normal cord insertion,</th></tr></table></td><td><u>Overall incidence</u>
1/318 (0.31%)

<u>Incidence associated with risk factors</u>
• 1/35 (2.9%) in women with low cord insertion
• 0/283 (0%) in women with normal cord insertion
OR reported in Ruiter 2016 [7]: 24.65 (95% CI 0.98 to 617.03)

Univariate relative risk for VP in cases of low cord insertion diagnosed at 9 to 11 weeks: 9.32 (95% CI 6.28 to 12.81)</td><td>NR</td></tr>
</table>

CONFIDENTIAL

<table>
<tr><th>Study reference, review questions</th><th>Study design</th><th>Population characteristics</th><th>Prevalence and incidence outcomes</th><th>Risk or incidence of perinatal outcomes associated with VP</th></tr>
<tr><td></td><td>complications and a cord insertion in the lower third of the uterus in the first trimester

<u>Dates</u>
June 2003 to January 2005

<u>Data sources</u>
Transabdominal ultrasound and postnatal placenta examination

<u>Country</u>
Japan

<u>Setting</u>
Showa University School of Medicine</td><td><table>
<tr><th></th><th>n=35</th><th>n=283</th></tr>
<tr><td>Age (years), mean (SD)</td><td>31.7 (3.8)</td><td>31.7 (4.5)</td></tr>
<tr><td>Parity, mean (SD)</td><td>0.7 (0.7)</td><td>0.6 (0.7)</td></tr>
<tr><td>Prior CS, n (%)</td><td>1 (2.9)</td><td>9 (3.2)</td></tr>
<tr><td>Prior dilatation and curettage, mean (SD)</td><td>0.6 (0.7)</td><td>0.4 (0.9)</td></tr>
<tr><td>Gestational age at delivery (weeks), mean (SD)</td><td>39.1 (1.9)</td><td>38.6 (1.2)</td></tr>
<tr><td>IVF/IUI, n (%)</td><td>3 (8.6)</td><td>11 (3.9)</td></tr>
</table>IUI, intrauterine insemination
None of the characteristics were significantly different between the groups</td><td></td><td></td></tr>
<tr><td>Heller 2014[25]

Relevant to Q1, Q3</td><td><u>Design</u>
Retrospective

<u>Objective</u>
To determine how often a low-lying placenta, defined as a</td><td><u>Patient recruitment methodology</u>
Computer search

<u>Sample size</u>
1,416 pregnancies had a diagnosis of a low-lying placenta between 16 and 24 weeks over the date range specified in the study. Of those, 176 women had no further follow-up sonographic examinations or obstetric care at the institution,</td><td><u>Overall incidence</u>
NR

<u>Incidence associated with risk factors</u>
VP was diagnosed in 3/20 (15%) women with low-lying placentas whose placentas did not clear by delivery

3/3 (100%) cases had VCI identified through</td><td><u>Emergency CS</u>
• Required in 1/3 (33%) VP cases
• Required in 1/17 (5.9%) non-VP cases
Calculated OR: 8.00 (95% CI 0.35 to 184.38, p=0.19)</td></tr>
</table>

CONFIDENTIAL

Study reference, review questions	Study design	Population characteristics	Prevalence and incidence outcomes	Risk or incidence of perinatal outcomes associated with VP
	placenta ending within 2 cm of the internal cervical os but not covering it, diagnosed sonographically in the second trimester resolves before delivery<br><br><u>Dates</u><br>July 2007 to September 2011<br><br><u>Data sources</u><br>Ultrasound database. The last sonographic examination (to identify low-lying placenta and vasa praevia, among other things) was performed an average of 5.9 days before delivery.<br><br><u>Country</u><br>USA<br><br><u>Setting</u><br>Women's	leaving a study cohort of 1240 placentas.<br><br><u>Baseline characteristics</u><br>1220/1240 (98.4%) of low-lying placentas eventually cleared the internal cervical os by 2 cm or greater.<br>• Mean GA at clearance was 26.0 weeks<br><br>Only reported for 20 women whose placentas did not clear by delivery.<br>• 1/20 (5%) pregnancies was twin<br>• 19/20 (95%) pregnancies were singleton<br><br>Mean GA at diagnosis, distance from placenta to internal os, GA at final ultrasound, if diagnosis was transvaginal, placental pathologic finding and placental locations for each of the 20 non-resolved by birth low-lying placentas were also reported.	placental pathologic examination.	

CONFIDENTIAL

Study reference, review questions	Study design	Population characteristics	Prevalence and incidence outcomes	Risk or incidence of perinatal outcomes associated with VP
	Hospital, Harvard Medical School			
**Huerta-Enochian 2001**[71]<br><br>Relevant to Q1	Design<br>Retrospective<br><br>Objective<br>Investigate whether adverse outcomes associated with elevated maternal serum α-fetoprotein levels may be prevented by intensive antenatal monitoring<br><br>Dates<br>November 1995 to June 2000<br><br>Data Sources<br>Charts<br><br>Country<br>USA<br><br>Setting<br>Communities of the southern Willamette Valley	Patient recruitment methodology<br>Chart review; included women who had undergone second-trimester triple-screen testing as part of routine obstetric care between November 1995 and June 2000. Women with maternal serum alpha fetoprotein levels >2.0 multiples of the median were included.<br><br>Sample size<br>The study enrolled 136 women. 23 women were excluded because of multiple gestations, structural or chromosomal abnormalities, or fetal death or for lack of available follow-up. 113 pregnancies were therefore included in analyses.<br><br>Baseline characteristics<br>Mean age was 24.6 years	Overall incidence<br>NR<br><br>Incidence associated with risk factors<br>1/113 *(0.88%)* pregnancies with maternal serum alpha fetoprotein levels >2.0 multiples of the median had VP	NR

CONFIDENTIAL

Study reference, review questions	Study design	Population characteristics	Prevalence and incidence outcomes	Risk or incidence of perinatal outcomes associated with VP
**Kanda 2011**[20]<br><br>Relevant to Q1, Q3	<u>Design</u><br>Retrospective<br><br><u>Objective</u><br>To evaluate the methods of screening and prenatal diagnosis of vasa praevia<br><br><u>Dates</u><br>January 2002 to December 2007<br><br><u>Data Sources</u><br>Medical records. Cord insertion was visualised using transabdominal ultrasound and the internal os was visualised using grey-scale transvaginal ultrasound. A diagnosis of VP was confirmed by transvaginal colour Doppler imaging<br><br><u>Country</u><br>Japan<br><br><u>Setting</u>	<u>Patient recruitment methodology</u><br>Appears that all VP cases identified through the medical record review were included<br><br><u>Sample size</u><br>5,131 deliveries<br><br><u>Baseline characteristics</u><br>• 473 multifetal pregnancies<br><br>For the VP group (all mean [SD]):<br>• Maternal age was 31.5 (8.4) years<br>• Maternal parity 0.7 (0.89)<br>• GA at diagnosis 26.6 (4.1) weeks<br>• GA at delivery 33.9 (3.2) weeks<br>Reason for referral, method of VP detection (either TAUS and grey-scale TVUS or colour Doppler TVUS), indication for delivery, pH of UA, gender also reported for each VP case	<u>Overall incidence</u><br>10/5,131 *(0.19%)* deliveries<br><br><u>Incidence associated with risk factors</u><br>10/10 *(100%)* of cases had one or more known risk factors<br>• 9/10 *(90%)* cases had low lying placenta<br>• 8/10 *(80%)* cases had VCI<br>• 10/10 *(100%)* had cord insertion to the lower uterine segment<br>• 4/10 *(40%)* pregnancies resulted from assisted reproductive technologies including IVF, artificial insemination with husband and intra cytoplasmic sperm injection	Outcomes for each case are reported separately in Table 1<br><br><u>Abnormal intrapartum fetal heart rate patterns</u><br>• 1/10 (*10%*) cases had an “ominous fetal heart rate pattern” as the indication for delivery<br>• 4/10 (*40%*) cases had non-reassuring fetal status (such as occasional late decelerations and variable decelerations) as the indication for delivery<br><br><u>Low Apgar scores at 1 and 5 minutes</u><br>Apgar scores at 1 minute:<br>• 6/10 *(60%)* with low score (<8)<br><br>Apgar scores at 5 minutes:<br>• 2/10 *(20%)* with low score (<8)<br><br><u>Low birth weight</u><br>• Mean (SD) neonatal weight was 2071 (551.3) g<br>• 7/10 *(70%)* cases had neonatal weight <2500 g<br><br><u>Pre-term delivery, including emergency CS</u><br>• 5/10 *(50%)* cases had pre-term labour, defined as progressive dilation of the cervix with uterine contractions before 36 weeks of gestation<br>• 9/10 *(90%)* cases were delivered before 37 weeks<br>• Mean (SD) GA at delivery 33.9 (3.2) weeks

CONFIDENTIAL

Study reference, review questions	Study design	Population characteristics	Prevalence and incidence outcomes	Risk or incidence of perinatal outcomes associated with VP
	Kagoshima City Hospital (a tertiary medical centre)			
**Kapoor 2014**[31]<br><br>Relevant to Q1	<u>Design</u><br>Retrospective cohort<br><br><u>Objective</u><br>To determine the rate of placenta praevia, vasa praevia and cord prolapse in the third trimester in women with low-lying placenta<br><br><u>Dates</u><br>May 2009 to June 2011<br><br><u>Data Sources</u><br>Electronic clinical database 'Viewpoint' and chart review<br><br><u>Country</u><br>Australia<br><br><u>Setting</u>	<u>Patient recruitment methodology</u><br>All identified publicly managed pregnancies with a low-lying placenta within 30 mm of the internal cervical os at the routine second trimester ultrasound were included<br><br>Cases reported with the placenta overlapping or covering the internal cervical os, and women transferred for management at the study hospital for antepartum haemorrhage from other facilities were excluded<br><br><u>Sample size</u><br>240 women had a follow-up ultrasound scan for placental localisation, of whom 181 women met the study criteria<br><br><u>Baseline characteristics</u><br>• Age (IQR) 27 to 34 years<br>• Mean (SD) GA at diagnosis of low-lying placenta 19.2 (1.0) weeks<br>• Transvaginal ultrasound for assessment of placental localisation at their centre performed at mean (SD) 34.1 (2.3) weeks<br>• Placenta praevia 15/181 (8.3%) cases<br>• Multiparity 102/181 *(56.4%)*<br>• Previous CS 31/181 *(17.1%)*	<u>Overall incidence</u><br>NR<br><br><u>Incidence associated with risk factors</u><br>• 2/181 (1.1%) cases of VP among women with low-lying placenta at second trimester ultrasound<br><br>Placental to os distance by second trimester scan:<br>• 0 to <10 mm – 2/42 (4.8%) cases<br>• 10 to 19 mm – 0/67 cases<br>• 20 to 30 mm – 0/37 cases<br>• No distance recorded – 0/35 cases	NR

CONFIDENTIAL

<table>
<tr><th>Study reference, review questions</th><th>Study design</th><th>Population characteristics</th><th>Prevalence and incidence outcomes</th><th>Risk or incidence of perinatal outcomes associated with VP</th></tr>
<tr><td></td><td>Mater Mothers Hospital (tertiary level maternity hospital)</td><td></td><td></td><td></td></tr>
<tr><td>Oyelese 2004[29]

Relevant to Q1, Q3</td><td><u>Design</u>
Retrospective chart review

<u>Objective</u>
To evaluate outcomes and predictors of neonatal survival in pregnancies complicated by VP and to compare outcomes in prenatally diagnosed cases of VP with those not diagnosed prenatally

<u>Dates</u>
1991 to 2003

<u>Data sources</u>
Chart review and perinatal ultrasound database

<u>Country</u></td><td><u>Patient recruitment methodology</u>
Cases were identified from two sources:
Women registering with the Vasa Previa Foundation website
Discharge diagnoses from 5 large hospital obstetric services in the UK, USA and Israel
<u>Sample size</u>
155 women with VP (only cases confirmed by pathology reported)

<u>Baseline characteristics</u>
Percentages are calculated as proportions of non-missing data
7/155 (4.5%) cases from the UK
Mean (SD) age 31.6 (4.9) years
Mean (SD) GA at delivery 36.9 (2.8) weeks
7/155 (4.5%) were twin pregnancies
12/154 (7.8%) had previous CS
60/155 (38.7%) had previous vaginal delivery
41/155 (26.5%) had previous spontaneous or therapeutic abortion
15/155 (9.7%) were a result of IVF</td><td><u>Overall incidence</u>
NA – all cases had VP

<u>Incidence associated with risk factors</u>
50/152 (32.9%) had bilobed placenta
95/153 (62.1%) had second-trimester low-lying placenta, of which 31/153 (20.3%) remained low-lying at delivery
56/155 (36.1%) had third-trimester bleeding, but a test was only performed to determine whether the blood was of maternal or fetal origin in 3 cases</td><td><u>Low Apgar scores at 1 and 5 minutes</u>
Apgar scores at among 100 survivors, median (range):
1 minute: 8 (0 to 10)
5 minutes: 9 (0 to 10)
<u>Neonatal and fetal deaths</u>
Overall perinatal mortality was 55/155 (35.5%)
30/155 (19.4%) stillbirths
25/125 (20%) neonatal deaths among live births
Survival associated with risk factors<table><tr><th>n/N (%) unless specified</th><th>Did not survive, n=55</th><th>Survive d, n=100</th><th>P</th></tr><tr><td>Maternal age, mean (SD)</td><td>30.6 (5.0)</td><td>32.1 (4.8)</td><td>0.08</td></tr><tr><td>Previous CS</td><td>4/54 (7.4%)</td><td>8/100 (8%)</td><td>0.90</td></tr><tr><td>Previous vaginal delivery</td><td>17/55 (30.9%)</td><td>43/100 (43%)</td><td>0.14</td></tr><tr><td>IVF</td><td>4/55 (7.3%)</td><td>11/100 (11%)</td><td>0.58</td></tr><tr><td>Bilobed placenta</td><td>11/54 (20.4%)</td><td>39/98 (39.8%)</td><td>0.01</td></tr><tr><td>Second-trimester</td><td>26/44 (48.1%)</td><td>69/98 (70.4%</td><td>0.001</td></tr></table></td></tr>
</table>

CONFIDENTIAL

<table>
<tr><th>Study reference, review questions</th><th>Study design</th><th>Population characteristics</th><th>Prevalence and incidence outcomes</th><th>Risk or incidence of perinatal outcomes associated with VP</th></tr>
<tr><td></td><td>UK, USA, Israel

<u>Setting</u>
Multicentre (5 hospitals and VP foundation referred cases)</td><td></td><td></td><td><table><tr><td>low-lying placenta</td><td></td><td>)</td><td></td></tr><tr><td>Low-lying placenta at delivery</td><td>2/55 (3.6%)</td><td>29/98 (29.6%)</td><td>0.001</td></tr></table>Survival associated with management (steroids, GA at delivery, mode of delivery) also reported, and extracted in Table 18.

<u>Pre-term delivery, including emergency CS</u>
80/154 (51.9%) cases were delivered by emergency CS

<u>Other outcomes</u>
• Transfusion was required in 26/100 (26%) survivor cases
• 74/151 (49.0%) had antepartum haemorrhage</td></tr>
<tr><td>Rebarber 2014[6]

Relevant to Q1, Q2, Q3</td><td><u>Design</u>
Retrospective cohort

<u>Objective</u>
Estimate the prevalence and persistence rate of vasa praevia in at-risk pregnancies using a standardised screening protocol

<u>Dates</u></td><td><u>Patient recruitment methodology</u>
Cases identified in charts by ICD-9, code analysis of billing system and separate content search of two ultrasound reporting systems with ‘vasa previa’ as keywords

Screening for VP using transvaginal sonography was performed routinely in the following clinical situations: resolved placenta praevia, history of VP in a prior pregnancy, VCI in the lower uterine segment, succenturiate placenta with implantation in the lower uterine segment, and twin gestations.

Once a VP was suspected with greyscale or colour flow imaging, confirmation was performed with pulsed wave Doppler imaging.</td><td><u>Overall incidence</u>
• Incidence from scans performed between 15 and 22 weeks GA was 31/27,573 (0.11%)
• 5 cases resolved before delivery, so the final incidence was as 26/27,573 (0.94/1000 births, 0.094%)

Of 32 diagnoses from sonography, 2 were lost to follow-up and 1 was misdiagnosed

<u>Resolution of VP</u>
29 cases identified sonographically were available for analysis (5 twin gestations, 19 singletons)
• 5/29 (17.2%) VP cases resolved across gestation
• All 5 cases that resolved were from cases diagnosed at second trimester (<26 weeks,</td><td><u>Low birth weight</u>
• Median (range) birth weight was 2525 (1320 to 3005) g

<u>Neonatal and fetal deaths</u>
• 24/24 survived, so perinatal mortality was 0%

<u>Pre-term delivery, including emergency CS</u>
• Median (range) gestation length was 35 (27.7 to 36.7) weeks, so 100% of deliveries were before 37 weeks
• 19/24 (79%) had elective delivery at 34 to 36 weeks
• 4/24 (17%) were urgently delivered for preterm labour and 1/24 (4.1%) was emergently delivered for vaginal bleeding</td></tr>
</table>

CONFIDENTIAL

Study reference, review questions	Study design	Population characteristics	Prevalence and incidence outcomes	Risk or incidence of perinatal outcomes associated with VP
	June 2005 to June 2012<br><br>Data Sources<br>Chart review. Imaging was conducted by registered diagnostic medical sonographers and maternal-fetal medicine specialists. The ultrasound unit has standardised policies and protocols for the evaluation of VP. Placental pathologic reports were reviewed in all cases to identify the incidence of VCI.<br><br>Country<br>USA<br><br>Setting<br>Carnegie Imaging for Women ultrasound unit	Sample size:<br>Of 27,573 women referred to the unit for fetal anatomic surveys over the study period, there were 32 cases of prenatally sonographically identified VP<br><br>Baseline characteristics:<br>• Age, years: median (IQR): 35 (24 to 58)<br>• White 85%<br>• Asian 9%<br>• Hispanic 6%<br><br>Among confirmed cases:<br>• 25/29 cases had previous resolved placenta praevia<br>• 2/29 cases had previous VCI<br>• 7/29 sets of twin pregnancies, 5 dichorionic diamniotic and 2 monochorionic diamniotic	5/21 [23.8%] cases), and none from the 8 cases diagnosed at 3 trimester (0/8)<br><br>Median GA at diagnosis for 5 resolved cases was 20 weeks 4 days (range 16 weeks 4 days to 25 weeks 2 days), and median GA at resolution was 25 weeks (range 22 weeks 5 days to 30 weeks)<br><br>Incidence associated with risk factors<br>Among all 29 pregnancies with a prenatal diagnosis of VP:<br>• *12/29* (41%) cases involved assisted reproductive technologies<br>• 21/29 (72%) had VCI<br>• 5/29 (17%) showed either a succenturiate lobe or a bilobed placenta<br><br>Among persistent VP cases:<br>• 20/24 (83%) had placenta praevia at some point in pregnancy	
**Robinson 2012**[22]	Design	Patient recruitment methodology	Overall incidence	NA

CONFIDENTIAL

Study reference, review questions	Study design	Population characteristics	Prevalence and incidence outcomes	Risk or incidence of perinatal outcomes associated with VP
Relevant to Q1	Prospective cohort<br><br><u>Objective</u><br>To determine if a low-lying placenta not overlapping the cervical os in the second trimester increases the risk of obstetric complications and whether there is a cut-off point at which that increase occurs<br><br><u>Dates</u><br>October 2007 to October 2008<br><br><u>Data Sources</u><br>Data were collected after birth from computerised perinatal records complemented with case note review for all women in the low-lying group	Eligible women had a singleton pregnancy, a routine morphology ultrasound at one of the two perinatal imaging departments and a placenta that did not overlap the cervical os<br><br><u>Sample size:</u><br>Of 4,593 singleton morphology ultrasound examinations performed at both centres during the study period, 484 women showed a placental edge within 0 to 30 mm from the cervical os. The control group included 797 women, slightly less than expected owing to recruiting practicalities<br><br><u>Baseline characteristics:</u><br>Baseline characteristics were reported for the group with low-lying placenta and the control group. As no cases of VP were reported, baseline data has not been extracted.<br><br>Reported characteristics included age (maternal and gestational), ethnicity, % nulliparous, smoking status, pre-existent hypertension or diabetes, previous CS<br><br>Median GA at the mid-trimester ultrasound was 19.6 weeks (IQR 19.1 to 20.0).	No VP cases found in the cohort *(0%)*	

CONFIDENTIAL

Study reference, review questions	Study design	Population characteristics	Prevalence and incidence outcomes	Risk or incidence of perinatal outcomes associated with VP
	Country<br>Australia<br><br>Setting<br>Two tertiary hospitals			
**Schachter 2002**[23]<br><br>Relevant to Q1, Q3	Design<br>Retrospective cohort<br><br>Dates<br>January 1987 to October 2001<br><br>Data Sources<br>Labour and delivery records, verified against charts<br><br>Country<br>Israel<br><br>Setting<br>NR	Patient recruitment methodology<br>All deliveries included<br><br>Sample size<br>72,818 deliveries<br><br>Baseline characteristics<br>1,173/72,818 (1.61%) resulted from IVF	Overall incidence<br>• 12/72,818 (0.017%)<br>• *1.65*/10,000 deliveries<br>• 1/6,068 deliveries<br><br>Incidence associated with risk factors<br>• 4/12 (33.3%) cases were pregnant through IVF<br>• Frequency of VP after IVF was *4*/1173, 34/10,000 deliveries *(0.34%)*, or 1/293 IVF deliveries.<br><br>OR for VP after IVF compared with non-IVF: 27.4 (95% CI 7.4 to 91.7, p<0.001)<br><br>(Note: this differs from the OR reported by Ruiter 2016[7] of 30.64 [95% CI 9.21 to 101.89])	Pre-term delivery, including emergency CS<br>Among the cases of VP associated with IVF, 3/4 *(75%)* delivered prematurely but 4/4 *(100%)* survived
**Smorgick 2010**[14]<br><br>Relevant to Q1, Q3	Design<br>Retrospective<br><br>Objective<br>To assess the prenatal detection of vasa praevia	Patient recruitment methodology<br>The department's computerised database of medical records was searched for diagnoses of 'vasa previa' and 'bleeding vasa previa'<br><br>The sonographic examination report and hard copy of sonographic images of the prenatally diagnosed cases were reviewed	Overall incidence<br>• 19/110,684 *(0.017%)*<br>• 1.7/10,000 deliveries<br><br>Incidence was 0.7 per 10,000 deliveries from 1988 to 1997, and 2.6 per 10,000 deliveries from 1998 to 2007	Outcomes were reported separately for each decade (1988 to 1997, 1998 to 2007)<br><br>Low Apgar scores at 1 minutes<br>Very low Apgar scores (<5) were reported at 1 minute only:<br>• 2/4 (50%) from 1988 to 1997<br>• 5/15 (33%) from 1998 to 2007<br>• *7/19 (36.8%) overall*

CONFIDENTIAL

Study reference, review questions	Study design	Population characteristics	Prevalence and incidence outcomes	Risk or incidence of perinatal outcomes associated with VP
	and its subsequent impact on neonatal outcomes<br><br><u>Dates</u><br>January 1988 to December 2007<br><br><u>Data sources</u><br>Computerised database of medical records. Sonographic diagnoses of VP were made with transvaginal sonography combined with colour/pulsed Doppler. Final diagnosis of each VP case was based on post-partum examination of the placenta and membranes<br><br><u>Country</u><br>Israel<br><br><u>Setting</u><br>Assaf Harofe Medical Center	<u>Sample size</u><br>110,684 deliveries at the medical centre during the 20-year study period<br><br><u>Baseline characteristics</u><br>Reported for 19 VP cases only:<br>• Median (range) maternal age 33 (22 to 46) years<br>• Median (range) parity 1 (0 to 6)<br>• 9/19 (47.3%) women had a previous<br>• dilatation and curettage because of a spontaneous or induced abortion<br>• 2/19 (5.3%) had previous CS<br>• 11/19 (57.9%) had spontaneous conception<br>• 1/19 (5.3%) controlled ovulation induction<br><br>Proportion of cases diagnosed prenatally:<br>• 1/4 (25%) from 1988 to 1997<br>• 9/15 (60%) from 1998 to 2007<br>• *10/19 (52.6%) overall*	<u>Incidence associated with risk factors</u><br>• 10/19 (52.7%) had VCI<br>• 5/19 (26.3%) had low-lying placenta<br>• 3/19 (15.8%) had succenturiate placental lobe<br>• 3/19 (15.8%) started as twin pregnancies<br>• 7/19 (36.8%) had IVF	<u>Low birth weight</u><br>Mean birthweight 2515 (range 1200 to 3600) g<br><br><u>Neonatal and fetal deaths</u><br>• 1/4 (25%) from 1988 to 1997<br>• 0/15 (0%) from 1998 to 2007<br>• *1/19 (5.3%) overall*<br><br><u>Pre-term delivery, including emergency CS</u><br>Mean GA at delivery was 36 (range 32 to 40) weeks<br><br>17/19 *(89.5%)* of cases were delivered by CS<br>• 7/9 (77.8%) cases with no prenatal diagnosis<br>• 10/10 (100%) cases with prenatal diagnosis, of which 5 were planned<br>• *12/19 (63.2%) had emergency CS overall*

CONFIDENTIAL

<table>
<tr><th>Study reference, review questions</th><th>Study design</th><th>Population characteristics</th><th>Prevalence and incidence outcomes</th><th>Risk or incidence of perinatal outcomes associated with VP</th></tr>
<tr><td rowspan="2">Suzuki 2015,[21] Suzuki 2010,[28] Suzuki 2008[30]

Relevant to Q1</td><td rowspan="2"><u>Design</u>
Retrospective

<u>Objective</u>
Suzuki 2015: To examine the clinical significance of pregnancies complicated by velamentous cord insertion (VCI) associated with other umbilical cord or placental abnormalities

Suzuki 2010: To examine the incidence of abnormally shaped placentae such as circumvallate placenta and succenturiate lobes of placenta and their complications in twin compared with singleton pregnancies

Suzuki 2008: To</td><td>Suzuki 2015[21]

<u>Patient recruitment methodology</u>
Women included the total population of women who gave birth to singleton babies at the study hospital between 2002 and 2011

<u>Sample size</u>
168 deliveries complicated by VCI and 16,797 unaffected controls

<u>Baseline characteristics</u>
<table><tr><th>All n (%)</th><th>No VCI, n= 16,797</th><th>VCI, n= 168</th><th>P value</th></tr><tr><td>Maternal age ≥ 35 years</td><td>5,755 (34.3)</td><td>63 (37.5)</td><td>0.37</td></tr><tr><td>Nulliparity</td><td>8,655 (51.5)</td><td>114 (6.8)</td><td><0.01</td></tr><tr><td>IVF use</td><td>900 (5.4)</td><td>36 (21.4)</td><td><0.01</td></tr><tr><td>Maternal smoking</td><td>357 (2.1)</td><td>9 (5.4)</td><td><0.01</td></tr><tr><td>Hypertensive disorders</td><td>702 (4.2)</td><td>18 (10.7)</td><td><0.01</td></tr></table></td><td>Suzuki 2015[21]

<u>Overall incidence</u>
3/16,965 (0.018%)

<u>Incidence associated with risk factors</u>
• 0/16,797 (0%) cases without VCI
• 3/168 (1.79%) cases with VCI
• OR NR, p<0.01</td><td>NR (only p-values reported)</td></tr>
<tr><td>Suzuki 2010[28]

<u>Patient recruitment methodology</u>
Women included the total population of women who gave birth at ≥22 weeks gestation at the study hospital between 2000 and 2007

<u>Sample size</u>
592 (174 monochorionic and 418 dichorionic) twin and 11,311 singleton pregnancies

<u>Baseline characteristics</u></td><td>Suzuki 2010[28]

<u>Overall incidence</u>
3/11,903 (0.025%)

<u>Incidence associated with risk factors</u>
Among singletons, VP was reported in:
• 1/83 (1.2%) with succenturiate placental lobes
• 2/11,228 (0.089%) without succenturiate placental lobes
• OR 68.4 (95% CI 6.2 to 760, p<0.01)</td><td>NR</td></tr>
</table>

CONFIDENTIAL

<table>
<tr><th>Study reference, review questions</th><th>Study design</th><th>Population characteristics</th><th>Prevalence and incidence outcomes</th><th>Risk or incidence of perinatal outcomes associated with VP</th></tr>
<tr><td rowspan="2"></td><td rowspan="2">examine the clinical significance of women complicated by succenturiate lobes of placenta in comparison with women with normal placentas

<u>Dates</u>
Suzuki 2015: 2002 to 2011

Suzuki 2010: 2000 to 2007

Suzuki 2008: 2002 to 2005

<u>Data sources</u>
Patient charts

<u>Country</u>
Japan

<u>Setting</u>
Japanese Red Cross Katsushika Maternity Hospital</td><td>
<table>
<tr><th>All n (%)</th><th>Singleton, n=11,311</th><th>Twin, n=592</th><th>P value</th></tr>
<tr><td>Maternal age ≥35 years</td><td>2,871 (25)</td><td>438 (74)</td><td><0.01</td></tr>
<tr><td>Nulliparity</td><td>5,484 (48)</td><td>377 (64)</td><td><0.01</td></tr>
<tr><td>Infertility therapy</td><td>158 (1.4)</td><td>94 (16)</td><td><0.01</td></tr>
<tr><td>Using IVF</td><td>100 (0.88)</td><td>75 (13)</td><td><0.01</td></tr>
</table>
</td><td>Among twins, VP was reported in:
• 0/9 (0%) with succenturiate placental lobes
• 0/583 (0%) without succenturiate placental lobes
• OR NR

VP was reported in:
• 3/11,311 (0.027%) singleton pregnancies
• 0/592 (0%) twin pregnancies</td><td></td></tr>
<tr><td>Suzuki 2008

<u>Patient recruitment methodology</u>
Women included the total population of women who gave birth to singleton babies at the study hospital between 2002 and 2005, excluding deliveries complicated by circumvallate placenta, circummarginate placenta or bilobed placenta

<u>Sample size</u>
47 women with succenturiate placenta and 7666 women without succenturiate placenta

<u>Baseline characteristics</u>
<table>
<tr><th>All n (%)</th><th>Succenturiate placenta, n= 47</th><th>Control n= 7666</th><th>P value</th></tr>
<tr><td>Maternal age <20 years</td><td>0</td><td>120 (1.6)</td><td>0.38</td></tr>
<tr><td>Maternal age ≥35 years</td><td>16 (34)</td><td>859 (11)</td><td><0.01</td></tr>
<tr><td>Multiparous</td><td>26 (55)</td><td>3,992 (52)</td><td>0.66</td></tr>
</table>
</td><td>Suzuki 2008

<u>Overall incidence</u>
3/7,713 (0.039%)

<u>Incidence associated with risk factors</u>
• 1/47 (2.1%) in women with succenturiate lobes
• 2/7,666 (0.026%) in women without succenturiate lobes.
• OR 83.30 (95% CI 7.42 to 934.90)</td><td>NR</td></tr>
</table>

CONFIDENTIAL

<table>
<tr><th>Study reference, review questions</th><th>Study design</th><th>Population characteristics</th><th>Prevalence and incidence outcomes</th><th>Risk or incidence of perinatal outcomes associated with VP</th></tr>
<tr><td></td><td></td><td><table><tr><td>History of infertility</td><td>6 (13)</td><td>158 (2.0)</td><td><0.01</td></tr><tr><td>History of IVF use</td><td>4 (8.5)</td><td>101 (1.3)</td><td><0.01</td></tr></table></td><td></td><td></td></tr>
<tr><td>Swank 2016[26]

Relevant to Q1, Q2, Q3</td><td><u>Design</u>
Retrospective

<u>Objective</u>
To investigate the diagnostic and management strategies for this potentially catastrophic entity and to describe further maternal and placental risk factors that may aid in the establishment of a screening protocol for vasa praevia

<u>Dates</u>
January 2000 to December 2012

<u>Data Sources</u>
Databases of ultrasound</td><td><u>Patient recruitment methodology</u>
Data from ultrasound scans and hospital records of women who delivered from January 1 2000 to December 31 2012 was reviewed to identify all VP cases with a diagnosis made antenatally or at the time of delivery

<u>Sample size</u>
68 pregnancies were identified that included the diagnosis of ‘vasa previa’ or ‘possible vasa previa’ either in the ultrasound record or in the hospital record at the time of delivery

<u>Baseline characteristics</u>
For the 47 cases with VP diagnosed antenatally and confirmed at delivery:
20/47 (42.6%) had maternal age ≥35
8/44 (18.2%) had an IVF pregnancy
5/47 (10.6%) had a multiple gestation
6/47 (12.8%) had no risk factors for VP (cases with risk factor missing data were not counted as “no risk factors”) (this likely inly included maternal age, IVF and multiple gestation as risk factors)</td><td><u>Resolution of VP cases</u>
4/68 cases (5.9%) resolved on repeat ultrasound at 21 (1 case), 32 (2 cases) and 33 (1 case) weeks of gestation
Additionally, 15/64 cases could not be verified at delivery

<u>Incidence associated with risk factors</u>
6/47 (12.8%) had no risk factors for VP or had missing risk factor data, so 41/47 (87.2%) had confirmed risk factors

Among the 49 total cases confirmed at delivery:
24/49 (49.0%) had low-lying placenta
12/49 (24.5%) had MCI
20/49 (40.8%) had VCI
17/49 (34.7%) had bilobed or succenturiate placenta</td><td><u>Pre-term delivery, including emergency CS</u>
Among 49 total cases confirmed at delivery, 8 cases (16.3%) required emergency CS.

Mean (SD) GA at delivery was:
34.69 (1.762) weeks for 47 cases diagnosed prenatally and confirmed after delivery
35.86 (0.404) weeks for 2 cases not diagnosed prenatally but confirmed at delivery</td></tr>
</table>

CONFIDENTIAL

CONFIDENTIAL

Study reference, review questions	Study design	Population characteristics	Prevalence and incidence outcomes	Risk or incidence of perinatal outcomes associated with VP
	scans, hospital and operative records of women<br><br>Country<br>USA<br><br>Setting<br>Multicentre; maternal fetal medicine practices and 1 university practice			

Note: Values in *italics* were calculated by the reviewer.
Abbreviations: 95% CI, 95% confidence interval; CS, caesarean section; IQR, interquartile range; IVF, in vitro fertilisation; MCI, marginal cord insertion; NA, not applicable; NR, not reported; OR, odds ratio; SD, standard deviation; VCI, velamentous cord insertion; VP, vasa praevia; VTS, vanishing twin syndrome

Criterion 4 – Screening tests for VP

Nine publications reported data on the effectiveness of screening in detection of VP. Two publications reported on the same cohort from the USA during overlapping time periods.[17, 37] There were therefore 8 unique cohorts of women reported. Where the two publications reported similar outcomes for overlapping cohorts of women, relevant data has been extracted into Table 17 from each publication and all unique outcomes of relevance to the question have been used in the question-level synthesis in Section 2.2.1.1.2.

Table 17. Studies relevant to criterion 4 – screening tests for VP

Study reference	Study design	Population characteristics	Screening methods	Screening accuracy
**Bronsteen 2013**[17]<br><br>Relevant to Q4	Design<br>Retrospective observational review<br><br>Objective<br>To review experience with	Patient recruitment methodology<br>Women who delivered at William Beaumont Hospital, Royal Oak, Michigan from January 1st 1990 to June 30th 2010	Screening<br>Ultrasound examinations were performed 15 weeks of gestation and later by board-certified sonologists and were reviewed by physicians certified by either Maternal-Fetal Medicine or Radiology boards	Sensitivity<br>Overall:<br>Reported as 56/58 (96.6%)<br><br>After initial ultrasound examination after 14 weeks of gestation:

<table>
<tr><th>Study reference</th><th>Study design</th><th>Population characteristics</th><th>Screening methods</th><th>Screening accuracy</th></tr>
<tr>
<td></td>
<td>diagnosis, clinical associations, and outcomes of VP in a single institution

<u>Dates</u>
January 1st 1990 to June 30th 2010

<u>Country</u>
USA

<u>Setting</u>
William Beaumont Hospital, Royal Oak, Michigan</td>
<td>VP cases diagnosed in the unit but delivered elsewhere were excluded

Cases were identified from the electronic files for all ultrasound scans performed in the Division of Fetal imaging and hospital discharge records

<u>Sample size</u>
182,554 pregnancies that had at least one ultrasound examination in the unit over the course of the study period

<u>Baseline characteristics</u>
NR</td>
<td>Protocol included a two-dimensional evaluation of the lower uterus and cervix

TVS and colour Doppler evaluation were not part of the routine protocol but were used when needed to assist in the pregnancy evaluation

If VP was not diagnosed on an earlier ultrasonogram, or VP resolved on a subsequent ultrasonogram, video recordings of the ultrasonogram were retrospectively reviewed

VP was diagnosed when a fetal vessel or vessels were noted in the lower uterus. The location of the membranous fetal vessel was classified as overlying the cervix or low-lying when present in the lower uterus but not directly over the cervix. When low-lying, the distance from the membranous vessel to the internal os was measured

<u>Reference Standard</u>
Outcome after delivery. Presumably placental examination, because the discussion reports that “because placental examination is not universal in our institution, we almost certainly have underascertained cases of vasa praevia”</td>
<td>Calculated as 26/58 (44.8%)
• Among the 32 cases not identified at initial examination, 8 were missed and 24 had placenta praevia, which precluded a diagnosis of VP

After the first ultrasound examination without a placenta covering the cervix: Reported as 45/58 (77.6%)

<u>Specificity</u>
Not possible to calculate

<u>PPV</u>
56/61 (91.8%) overall

<u>NPV</u>
Not possible to calculate

For initial examination only:
<table>
<tr><th></th><th>VP +</th><th>VP -</th><th>Total</th></tr>
<tr><td>Screen +</td><td>26</td><td>NR</td><td>NR</td></tr>
<tr><td>Screen -</td><td>32</td><td>NR</td><td>NR</td></tr>
<tr><td>Total</td><td>58</td><td>NR</td><td>NR</td></tr>
</table></td>
</tr>
<tr>
<td>Catanzarite 2001[2]

Relevant to Q4</td>
<td><u>Design</u>
Prospective observational review

<u>Objective</u>
To assess the specificity of sonographic diagnosis of vasa praevia and pregnancy outcome in sonographically diagnosed cases

<u>Dates</u>
January 1991 to December 1998</td>
<td><u>Patient recruitment methodology</u>
Pregnant women who were scanned and subsequently delivered at Sharp Perinatal Center

<u>Sample size</u>
33,208 pregnant women

<u>Baseline characteristics</u>
NR</td>
<td><u>Screening</u>
Ultrasound examinations were performed using Ultramark 9, 9-HDI and 9-ESP systems (Advanced Technology Laboratories, Bothell, WA, USA) and Acuson XP-128 and Sequoia systems (Acuson Corporation, Mountain View, CA, USA)

Sonograms were performed transabdominally according to a protocol including imaging of the uterus and cervix, adnexa, placental location, and a comprehensive anatomical survey. If the cervix could not be imaged due to technical factors, then TVS was performed at the discretion of the sonographer</td>
<td>Of 11 suspected cases of VP without placenta praevia, the diagnosis was confirmed at delivery in 10

Three additional patients were followed for VP coexistent with marginal placenta praevia, with sonographic findings confirmed by clinical and/or pathologic examination

The authors received feedback from two referring obstetricians regarding possible missed cases of VP. Placental examination showed VCI in both. In one of the cases, retrospective review of the videotape of the greyscale examination showed linear echoes</td>
</tr>
</table>

CONFIDENTIAL

<table>
<tr><th>Study reference</th><th>Study design</th><th>Population characteristics</th><th>Screening methods</th><th>Screening accuracy</th></tr>
<tr>
<td></td>
<td><u>Country</u>
USA

<u>Setting</u>
Sharp Perinatal Center</td>
<td></td>
<td>Screening techniques changed over time. Initially, colour Doppler was used only when fetal vessels over the cervix were suspected, based on grey-scale imaging. From 1993 onwards, routine imaging of cord insertion was attempted. Since 1995, colour Doppler and/or colour power angiography imaging was used over the cervix in women with low-lying placentae with accessory lobes. During 1998, routine colour Doppler or colour power angiography was added to ‘sweep’ across the lower uterine segment, specifically to detect velamentous vessels over the cervix

The diagnosis of suspected VP was made if the placenta was clear of the internal os of the cervix and fetal vessels were identified over the cervix. If the diagnosis was suspected prior to 26 weeks’ gestation, it was confirmed by TVS or by a repeat sonogram after 26 weeks, at the discretion of the attending perinatologist

<u>Reference standard</u>
Confirmation of the ultrasound findings were based on the obstetrician’s operative findings and placental pathological examination</td>
<td>over the cervix consistent with VP

<u>Sensitivity</u>
13/15 (86.7%)

<u>Specificity</u>
33,192/33,193 (99.997%)

<u>PPV</u>
13/14 (92.9%)

<u>NPV</u>
33,192/33,194 (99.994%)

<u>Accuracy</u>
33,205/33,208 (99.99%)

<table>
<tr><th></th><th>VP +</th><th>VP-</th><th>Total</th></tr>
<tr><td>Screen +</td><td>13</td><td>1</td><td>14</td></tr>
<tr><td>Screen -</td><td>2</td><td>33,192</td><td>33,194</td></tr>
<tr><td>Total</td><td>15</td><td>33,193</td><td>33,208</td></tr>
</table>

Note that specificity was reported as 91%, but this was actually referring to PPV, and only among cases of VP in the absence of placenta praevia</td>
</tr>
<tr>
<td>Kanda 2011[20]

Relevant to Q4</td>
<td><u>Design</u>
Retrospective record review

<u>Objective</u>
To evaluate the methods of screening and prenatal diagnosis of VP

<u>Dates</u>
January 2002 to December</td>
<td><u>Patient recruitment methodology</u>
Medical records of women with VP at the study centre were examined

<u>Sample size</u>
5131 deliveries

<u>Baseline characteristics</u>
NR</td>
<td><u>Screening</u>
Cord insertion site was visualised using TAS and the internal os was observed using grey-scale TVS as part of second-trimester screening between 20 and 25 weeks of gestation. If VP was suspected, the diagnosis was confirmed with colour Doppler TAS or TVS

For women attending the hospital in the third trimester (GA 26 or more weeks), screening was performed as for the other women</td>
<td>Nine cases diagnosed prenatally
• All four cases that underwent second-trimester screening were accurately diagnosed
• Of 6 women referred to the hospital after 26 weeks:
  ○ 3 had VP detected at routine ultrasonography
  ○ 2 had VP detected at repeat detailed examination using colour Doppler TVS after the presence of non-reassuring FHR patterns</td>
</tr>
</table>

CONFIDENTIAL

<table>
<thead>
<tr><th>Study reference</th><th>Study design</th><th>Population characteristics</th><th>Screening methods</th><th>Screening accuracy</th></tr>
</thead>
<tbody>
<tr>
<td></td>
<td>2007

<u>Country</u>
Japan

<u>Setting</u>
Kagoshima City Hospital, a tertiary medical centre</td>
<td></td>
<td><u>Reference standard</u>
Unclear</td>
<td>
1 was not diagnosed antenatally
Looking at routine ultrasound only:

<u>Sensitivity</u>
7/10 (70%)

<u>PPV</u>
7/7 (100%)

<table>
<tr><th></th><th>VP +</th><th>VP-</th><th>Total</th></tr>
<tr><td>Screen +</td><td>7</td><td>0</td><td>7</td></tr>
<tr><td>Screen -</td><td>3</td><td>NR</td><td>NR</td></tr>
<tr><td>Total</td><td>10</td><td>NR</td><td>5131</td></tr>
</table>

Looking at all prenatal ultrasound:

<u>Sensitivity</u>
9/10 (90%)

<u>PPV</u>
9/9 (100%)

<table>
<tr><th></th><th>VP +</th><th>VP-</th><th>Total</th></tr>
<tr><td>Screen +</td><td>9</td><td>0</td><td>9</td></tr>
<tr><td>Screen -</td><td>1</td><td>NR</td><td>NR</td></tr>
<tr><td>Total</td><td>10</td><td>NR</td><td>5131</td></tr>
</table>
</td>
</tr>
<tr>
<td>Nomiyama 1998[38]

Relevant to Q4</td>
<td><u>Design</u>
Prospective cohort

<u>Objective</u>
To determine whether cord insertion can be consistently visualised and whether VCI and VP can be consistently identified with colour</td>
<td><u>Patient recruitment methodology</u>
All fetuses scanned at 18 to 20 weeks' GA by one sonographer during routine examination

<u>Sample size</u>
587 fetuses (571 pregnancies)

<u>Baseline characteristics</u></td>
<td><u>Screening</u>
Routine colour Doppler TAS was used to assess cord insertion. When the insertion was velamentous, the sonographer was instructed to indicate whether or not it was VP (defined as VCI when the umbilical vessels traverse the fetal membranes in the lower uterine segment in front of the presenting part)

Cases of VCI had repeat sonograms at 30 and 36 weeks. At this stage, if cord insertion could not be visualised by TAS,</td>
<td><u>Sensitivity</u>
1/1 (100%)

<u>Specificity</u>
585/586 (99.8%)

<u>PPV</u>
1/2 (50%)</td>
</tr>
</tbody>
</table>

CONFIDENTIAL

Study reference	Study design	Population characteristics	Screening methods	Screening accuracy
	Doppler imaging during routine sonography in the mid-trimester<br><br><u>Dates</u><br>January 1993 to December 1996<br><br><u>Country</u><br>Japan<br><br><u>Setting</u><br>Saga Prefectural Hospital Kouseikan	555 singletons and 16 sets of twins	colour Doppler TVS was used<br><br><u>Reference standard</u><br>One case was confirmed at the time of CS. The other case could not be identified on repeat ultrasound examinations at 30 and 36 weeks	<u>NPV</u><br>*585/585 (100%)*<br><br><u>Accuracy</u><br>*586/587 (99.8%)*

	VP +	VP-	Total
Screen +	1	1	2
Screen -	0	*585*	*585*
Total	1	*586*	587

Note: Values in *italics* were calculated by the reviewer.

Abbreviations: 95% CI, 95% confidence interval; FHR, fetal heart rate; GA, gestational age; MCI, marginal cord insertion; NPV, negative predictive value; NR, not reported; PPV, positive predictive value; VP, vasa praevia

Criteria 9 and 10 – Management pathways for VP

Three studies that report on outcomes depending on differential management of VP were identified. One study reported on outcomes depending on whether women were treated as in or outpatients and two studies reported on outcomes depending on whether women were diagnosed with VP prenatally. These outcomes are reported in Table 18 and have been used for the question-level synthesis discussed in Section 2.2.3.1.

Table 18. Studies relevant to criteria 9 and 10 – Management pathways for VP

Study reference	Study characteristics	Population characteristics	Management pathways considered	Outcomes related to each management pathway
**Bronsteen 2013**[17]	<u>Design</u><br>Retrospective<br><br><u>Objective</u><br>To review experience	<u>Patient recruitment methodology</u><br>All VP cases identified from ultrasound records or hospital discharge files		<u>Bronsteen 2013</u><br>3/4 cases not diagnosed prenatally were delivered emergently. The fourth woman was delivered with recurrent antepartum bleeding and normal fetal heart rate tracings. Rate of emergency deliveries in the prenatally diagnosed group was 7% compared to 75% in

CONFIDENTIAL

<table>
<tr><th>Study reference</th><th>Study characteristics</th><th>Population characteristics</th><th>Management pathways considered</th><th>Outcomes related to each management pathway</th></tr>
<tr><td></td><td>with diagnosis, clinical associations, and outcomes of vasa praevia in a single institution.

<u>Dates</u>
January 1990 to June 2010

<u>Data sources</u>
Electronic files for all ultrasound scans and hospital discharge records

<u>Country</u>
USA

<u>Setting</u>
William Beaumont Hospital</td><td><u>Sample size</u>
182,554 pregnancies with at least one ultrasound examination
Outcomes reported for 60 cases of VP, and incidence of placental or cord abnormalities for 58 cases

<u>Baseline characteristics</u>
None reported</td><td></td><td>undiagnosed cases.</td></tr>
<tr><td>Hasegawa 2015[18]</td><td><u>Design</u>
Retrospective

<u>Objective</u>
Unclear

<u>Dates</u>
January 2005 to October 2013

<u>Data sources</u>
Unclear</td><td><table>
<tr><th>Median (range)</th><th>Inpatient, n = 15</th><th>Outpatient, n = 6</th></tr>
<tr><td>Age</td><td>31 (23 to 37)</td><td>36.5 (30 to 42)</td></tr>
<tr><td>Gravida</td><td>1 (0 to 5)</td><td>1 (0 to 3)</td></tr>
<tr><td>Parity</td><td>0 (0 to 1)</td><td>0 (0 to 1)</td></tr>
</table></td><td><u>Management pathway</u>
Treatment as inpatient or outpatient

VP women not routinely admitted, in their institution, although precise outpatient management, including confirming the presence of uterine contractions and measuring the cervical length and fetal heart rate, are provided for</td><td><table>
<tr><th>Median (range) or n (%)</th><th>Inpatient, n = 15</th><th>Outpatient, n = 6</th></tr>
<tr><td>GA at delivery</td><td>35 w 4 d (27 to 36)</td><td>36 w 2 d (35 to 36)</td></tr>
<tr><td>Emergency CS</td><td>4 (27%)</td><td>1 (17%)</td></tr>
<tr><td>Indications for emergency CS</td><td>Abnormal heart rate tracing (2)
Preterm PROM (2)</td><td>Preeclampsia (1)</td></tr>
<tr><td>Rescheduled CS ahead of initially planned operation</td><td>3 (20)</td><td>0 (0)</td></tr>
</table></td></tr>
</table>

CONFIDENTIAL

CONFIDENTIAL

<table>
<tr><th>Study reference</th><th>Study characteristics</th><th>Population characteristics</th><th>Management pathways considered</th><th>Outcomes related to each management pathway</th></tr>
<tr>
<td></td>
<td><u>Country</u>
Japan

<u>Setting</u>
Showa University School of Medicine</td>
<td></td>
<td>pregnant females with vasa praevia.

After 30 weeks' of gestation, these examinations were performed weekly. In addition, fetal growth and amniotic fluid volume are evaluated every 2 weeks throughout the patient's pregnancy. Only when abnormal findings were detected on these examinations, the women with VL were hospitalised. Elective CS was planned at 35 to 36 weeks of gestation.

15 women with VP required admission to the hospital due to a low-lying placenta with bleeding, fetal growth restriction, uterine contractions, shortened uterine cervix, and/or abnormal heart rate tracing. 6 women were managed as outpatients.</td>
<td>
<table>
<tr><td>Indications for rescheduled CS</td><td>Uterine contractions: 2
Fetal growth arrest: 1</td><td>NA</td></tr>
<tr><td>Neonatal birth weight (g)</td><td>2017 (1211 to 3359)</td><td>2450 (2181 to 2875)</td></tr>
<tr><td>Light for date</td><td>5 (33)</td><td>0 (0)</td></tr>
<tr><td>Apgar score at 1 minute</td><td>8 (1 to 9)</td><td>8 (8 to 9)</td></tr>
<tr><td>Apgar score at 5 minutes</td><td>9 (6 to 10)</td><td>9 (8 to 10)</td></tr>
<tr><td>Admission to neonatal intensive care unit</td><td>11 (73%)</td><td>2 (33%)</td></tr>
<tr><td>Indications for admission</td><td>Preterm birth and/or low birth weight: 11</td><td>Ventricular septal defect: 1
Apnoea: 1</td></tr>
</table>
</td>
</tr>
<tr>
<td>Oyelese 2004[29]</td>
<td><u>Design</u>
Retrospective chart review

<u>Objective</u>
To evaluate</td>
<td><u>Patient recruitment methodology</u>
Cases identified in charts and referred by VP Foundation (with medical records retrieved from their physicians)</td>
<td>• 61/155 diagnosed prenatally
• 94/155 did not have prenatal diagnosis

<u>Steroids</u>
Steroids given to 52/154</td>
<td>Outcomes by prenatal diagnosis
<table>
<tr><th>Median (range) or n (%)</th><th>Prenatal diagnosis (n=61)</th><th>No prenatal diagnosis (n=94)</th></tr>
<tr><td>Survival</td><td>59/61 (96.7%)</td><td>41/94 (43.6%)</td></tr>
<tr><td>Stillbirth</td><td>1/61</td><td>29/94</td></tr>
</table>
</td>
</tr>
</table>

<table>
<tr><th>Study reference</th><th>Study characteristics</th><th>Population characteristics</th><th>Management pathways considered</th><th>Outcomes related to each management pathway</th></tr>
<tr>
<td></td>
<td>outcomes and predictors of neonatal survival in pregnancies complicated by VP and to compare outcomes in prenatally diagnosed cases of VP with those not diagnosed prenatally.

<u>Dates</u>
1991 to 2003

<u>Data Sources</u>
Charts and perinatal ultrasound database

<u>Country</u>
UK, USA, Israel

<u>Setting</u>
Multicentre (5 hospitals and VP foundation referred cases)</td>
<td><u>Sample size</u>
155 women with VP (only cases verified by pathology were included)

<u>Baseline characteristics</u>
• Mean (SD) age 31.6 (4.9) years
• Mean (SD) GA at delivery 36.9 (2.8) weeks
• Twin pregnancy 7/155 (4.5%)
• Previous CS 12/154 (7.8%)
• Previous vaginal delivery 60/155 (38.7%)
• Previous spontaneous or therapeutic abortion 41/155 (26.5%)
• IVF 15/155 (9.7%)
• Smoking 12/155 (7.7%)</td>
<td>(33.8%) cases
Of these:
• 49/61 (80.3%) prenatally diagnosed
• 3/93 (3.2%) not prenatally diagnosed (P<0.001)
• 50/52 (96.2%) survived
• 2/52 (3.8%) died (P<0.001)

<u>GA at delivery</u>
• 34.9 (SD 2.5) weeks in prenatally diagnosed cases
• 38.2 (SD 2.1) weeks in non-prenatally diagnosed cases
• P<.001

<u>GA by survival</u>
Mean (SD)
• 37.6 (3) weeks in those that died
• 36.5 (2.6) weeks in surviving infants
• P=0.02</td>
<td><table>
<tr><td></td><td>(1.6%)</td><td>(30.1%)</td></tr>
<tr><td>Apgar score at 1 minute[a]</td><td>8 (7 to 10)</td><td>1 (0 to 10)</td></tr>
<tr><td>Apgar score at 5 minutes[a]</td><td>9 (7 to 10)</td><td>4 (0 to 10)</td></tr>
<tr><td>Neonatal death[a]</td><td>1/60 (1.67)</td><td>24/65 (36.9)</td></tr>
<tr><td>Transfusion[b]</td><td>2/59 (3.4)</td><td>24/41 (58.5)</td></tr>
</table>
P<0.001 for all outcomes
[a] based on live births, n=125
[b] based on survivors, n=100

<u>By multivariate regression, significant predictors of survival were</u>
• Prenatal diagnosis OR 102.9 (95% CI 16.2 to 638.3; P<0.001)
• GA at delivery OR 0.77 (95% CI 0.64 to 0.93; P=0.01)

<u>Survival by presence of prenatal diagnosis and delivery</u>
46/154 had elective CS, including:
• 42/61 (68.9%) who had prenatal diagnosis
• 4/93 (4.3%) who had no prenatal diagnosis
45/46 survived (97.8%)

80/154 (51.9) had emergency CS, including:
• 16/61 (27.9%) who had prenatal diagnosis
• 63/93 (67.7%) who had no prenatal diagnosis
47/81 survived (58.8%)
34/81 died (42.0%)
(Note that there is a discrepancy between the number reported as having emergency CS [80] and the numbers reported as surviving and dying [47 + 34 = 81])

28/154 (18.2%) had a vaginal delivery, including:
• 2/61 (3.3%) who had prenatal diagnosis
• 26/93 (28.0%) who had no prenatal diagnosis
8/28 survived (28.6%)
20/28 died (71.4%)</td>
</tr>
</table>

CONFIDENTIAL

Study reference	Study characteristics	Population characteristics	Management pathways considered	Outcomes related to each management pathway

Studies relating to VCI

Criterion 1 – Epidemiology of VCI

Twenty-four publications reported data on the epidemiology of VCI. Three publications reported on women from the same medical centres in Portugal and the Netherlands during overlapping time periods,[45, 49, 50] two publications reported on the same cohort from the USA during overlapping time periods,[51, 52] two publication reported on the same cohort of women during overlapping time periods in Norway,[1, 54] and two publications reported on the same cohort from Japan during overlapping time periods.[41, 55] There were therefore 19 unique cohorts of women reported. Where multiple publications reported similar outcomes for overlapping cohorts of women, relevant data has been extracted into from each publication (Table 19), but only the publication which followed up the cohort at the most recent time point has been used in the question-level synthesis in Section 2.3.1.

Table 19. Studies relevant to criterion 1 – Epidemiology of VCI

<table>
<tr><th>Study reference</th><th>Study design</th><th>Population characteristics</th><th>Prevalence and incidence outcomes</th><th>Risk or incidence of perinatal outcomes associated with VCI</th></tr>
<tr>
<td>Baumfeld 2016[35]

Relevant to Q6 and Q7</td>
<td><u>Design</u>
Retrospective cohort

<u>Objective</u>
Each publication had a different objective: to investigate the fetal heart rate (FHR) patterns in pregnancies complicated with VP and VCI; to evaluate whether vanishing twin</td>
<td><u>Patient recruitment methodology</u>
Women with VCI from the deliveries in the time period indicated

<u>Sample size</u>
184 women with VCI and their subsequent pregnancies

<u>Baseline characteristics</u></td>
<td><table>
<tr><th>Variables</th><th>VCI n=184</th><th>No VCI n=184</th><th>P value</th></tr>
<tr><td>Singleton (%)</td><td>153 (80.1)</td><td>183 (99.5)</td><td><.001</td></tr>
<tr><td>Multiple (%)</td><td>38 (19.9)</td><td>1 (0.5)</td><td></td></tr>
</table></td>
<td>Perinatal Outcomes between VCI and Non-VCI cases

Fetal monitor categories were as follows:
• 1st – baseline rate 110 to 160 bpm, moderate FHR variability (amplitude 6 to 25 bpm), no late or variable decelerations
• 3rd – absent baseline FHR variability and any of the following: recurrent late decelerations, recurrent variable decelerations, bradycardia; or, a sinusoidal pattern
• 2nd – all FHR patterns not classified as 1st or 3rd category

Note that although the study claims N=184 for each category, the numbers reported as percentage suggest there were 153 infants in the VCI and 154 in the non-VCI category.</td>
</tr>
</table>

CONFIDENTIAL

Study reference	Study design	Population characteristics	Prevalence and incidence outcomes	Risk or incidence of perinatal outcomes associated with VCI
	syndrome (VTS) is associated with adverse perinatal outcome; and to investigate risk factors and pregnancy outcome of women with placenta praevia<br><br><u>Dates</u><br>1988 to 2012 (Baumfeld 2016 and Evron 2015); 1988 to 2009 (Rosenberg 2011)<br><br><u>Data sources</u><br>Computerised perinatal database, which included information on maternal comorbidities, perinatal assessment, maternal and fetal complications. The obstetrical information is entered immediately after birth by an obstetrician, and is routinely checked for inaccuracies. Only			**Fetal heart rate patterns in VCI and non-VCI pregnancies** (table below)

Fetal heart rate patterns in VCI and non-VCI pregnancies

	VCI n=153	No VCI n=158	P value
Monitor, n (%)			
1st category	54 (35.3)	70 (44.3)	
2nd category	98 (64.1)	87 (55.1)	
3rd category	1 (0.7)	1 (0.6)	
Baseline fetal heart rate, n (%)			
Normal	141 (92.8)	154 (98.1)	
Tachycardia	7 (4.6)	2 (1.3)	
Bradycardia	4 (2.6)	1 (0.6)	
Abnormal baseline	11 (7.2)	3 (1.9)	0.02
Variability, n (%)			
Absent	1 (0.7)	0	
Minimal	48 (31.6)	35 (22.3)	
Moderate	102 (67.1)	121 (77.1)	
Abnormal (absent and minimal)	49 (32.2)	35 (22.3)	0.05
Normal (moderate and marked)	103 (67.8)	122 (77.7)	
Accelerations, n (%)			
Absent	55 (36.2)	47 (29.9)	0.24
Present	97 (63.8)	110 (70.1)	
Decelerations, n (%)			
Absent	65 (42.8)	72 (45.9)	0.02
Early	7 (4.6)	21	

CONFIDENTIAL

<table>
<tr><th>Study reference</th><th>Study design</th><th>Population characteristics</th><th>Prevalence and incidence outcomes</th><th>Risk or incidence of perinatal outcomes associated with VCI</th></tr>
<tr><td></td><td>four skilled medical secretaries examine the information before entering it into the database

<u>Country</u>
Israel

<u>Setting</u>
Soroka University Medical Center, a 1007 bed tertiary teaching hospital</td><td></td><td></td><td>
<table>
<tr><td></td><td></td><td>(13.4)</td><td></td></tr>
<tr><td>Late</td><td>8 (5.3)</td><td>3 (1.9)</td><td></td></tr>
<tr><td>Variable</td><td>71 (46.7)</td><td>61 (38.9)</td><td></td></tr>
<tr><td>Present</td><td>85 (57.0)</td><td>85 (54.1)</td><td>0.65</td></tr>
<tr><td colspan="4">Sinusoidal pattern, n (%)</td></tr>
<tr><td>All</td><td>1 (0.7)</td><td>1 (0.6)</td><td>1.0</td></tr>
</table>

<u>Low Apgar scores at 1 min and 5 min</u>
Low Apgar scores are <7, normal scores were ≥8

Apgar scores at 1 minute in VCI and non-VCI pregnancies
<table>
<tr><th></th><th>Low n (%)</th><th>Normal n (%)</th><th>OR (95% CI)</th></tr>
<tr><td>VCI n=194</td><td>29 (14.9)</td><td>165 (85.1)</td><td rowspan="2">2.62 (1.27 to 5.42), p=0.0094</td></tr>
<tr><td>No VCI n=175</td><td>11 (6.3)</td><td>164 (93.7)</td></tr>
</table>

Apgar scores at 5 min in VCI and non-VCI pregnancies
<table>
<tr><th></th><th>Low, n (%)</th><th>Normal , n (%)</th><th>OR (95% CI)</th></tr>
<tr><td>VCI n=194</td><td>10 (5.2)</td><td>184 (94.8)</td><td rowspan="2">2.44 (1.17 to 5.09, p=0.018)</td></tr>
<tr><td>No VCI n=175</td><td>5 (2.9)</td><td>170 (97.1)</td></tr>
</table>

<u>Perinatal Mortality</u>
Includes stillbirths, intrapartum and postpartum deaths
<table>
<tr><th></th><th>Death, n (%)</th><th>No death, n (%)</th><th>OR (95% CI)</th></tr>
<tr><td>VCI n=184</td><td>11 (5.6)</td><td>173 (94.4)</td><td>3.84 (1.05 to 13.99,</td></tr>
</table>
</td></tr>
</table>

CONFIDENTIAL

<table>
<tr><th>Study reference</th><th>Study design</th><th>Population characteristics</th><th>Prevalence and incidence outcomes</th><th>Risk or incidence of perinatal outcomes associated with VCI</th></tr>
<tr><td></td><td></td><td></td><td></td><td>
<table>
<tr><td>No VCI n=184</td><td>3 (1.5)</td><td>181 (98.5)</td><td>p=0.04)</td></tr>
</table>
<u>Pre-term delivery, including emergency CS</u>
Deliveries reported in Table 1 do not appear to include all pregnancies but the reason is unclear

Delivery route in VCI and non-VCI pregnancies
<table>
<tr><th></th><th>Emergency CS, n (%)</th><th>Elective CS, n (%)</th><th>Vaginal, n (%)</th></tr>
<tr><td>VCI, n=184</td><td>60 (31.7)</td><td>12 (6.3)</td><td>117 (61.9)</td></tr>
<tr><td>No VCI, n=184</td><td>27 (17.1)</td><td>14 (8.9)</td><td>117 (74.1)</td></tr>
</table>
Delivery Route in VCI and Non-VCI Pregnancies

Calculated OR for emergency CS in VCI vs non-VCI pregnancies: 2.26 (95% CI 1.35 to 3.78, p=0.002)
Calculated OR for elective CS in VCI vs non-VCI pregnancies: 0.70 (95% CI 0.31 to 1.55, p=0.38)

Calculated OR for total CS in VCI vs non-VCI pregnancies: 1.76 (95% CI 1.11 to 2.79, p=0.017)
</td></tr>
<tr><td>Chu 2013[56]

Relevant to Q7</td>
<td><u>Design</u>
Retrospective case-control

<u>Objective</u>
To determine whether this correlation between cord insertion type and vascularization of the co-twin also extends to the deeper chorionic villus tree</td>
<td><u>Patient recruitment methodology</u>
Diamniotic monochorionic twins with VCI/paracentral (PC) cords compared with PC/PC cords. TTTS and preeclampsia cases were excluded.

<u>Sample size</u>
12 VCI/PC cases and 10 PC/PC cases

<u>Baseline characteristics</u>

Maternal and gestation age in VCI/PC and PC/PC cases
<table>
<tr><th></th><th>VCI/PC (n=12)</th><th>PC/PC (n=10)</th></tr>
</table>
</td>
<td>No incidence reported</td>
<td><u>Birth weight</u>
Mean birthweight, g (SD) was reported for twins with PC/PC insertions and separately for each insertion in PC/VCI twin pairs:

PC/PC cases: 2419 (511)
VCI/PC cases:

infants with VCI: 1895 (455) (p<0.02 vs PC/PC)
infants with PC: 2236 (440)

Inter-twin birthweight discordance and placental weight also reported</td></tr>
</table>

CONFIDENTIAL

<table>
<tr><th>Study reference</th><th>Study design</th><th>Population characteristics</th><th>Prevalence and incidence outcomes</th><th>Risk or incidence of perinatal outcomes associated with VCI</th></tr>
<tr>
<td></td>
<td><u>Dates</u>
Mid-2010 to mid-2011

<u>Data sources</u>
Pathology records and charts

<u>Country</u>
USA

<u>Setting</u>
Women and Infants Hospital</td>
<td><table>
<tr><td>maternal age, years (SD)</td><td>28.6 (6.9)</td><td>27.9 (6.8)</td></tr>
<tr><td>gestational age, weeks (SD)</td><td>33.7 (3.7)</td><td>34.3 (2.6)</td></tr>
</table>
No significant differences were reported between the groups for these parameters.</td>
<td></td>
<td></td>
</tr>
<tr>
<td>Costa-Castro 2013,[45] Lopriore 2007,[50] Lopriore 2012[49]

Relevant to Q6 and Q7</td>
<td><u>Design</u>
Retrospective cohort (Costa-Castro 2013), Retrospective case-control (Lopriore 2012)

<u>Objective</u>
To study the association between VCI and different outcomes in monochorionic twins with and without twin-twin transfusion syndrome (TTTS)

<u>Dates</u>
June 2002 to September 2012 (Costa-Castro 2013), April 2002</td>
<td><u>Patient recruitment methodology</u>
All consecutive placentas of monochorionic diamniotic twins examined in the time period at these institutions were included. Cases (n=67) with monochorionic twin pregnancies with twin anemia-polycythemia sequence, twin reversed arterial perfusion, monoamniotic twins and higher multiple, pregnancies or placentas with intrauterine fetal demise where maceration prevented cord insertion examination were excluded.

<u>Sample size</u>
630 placentas

<u>Baseline characteristics</u>
Mean gestational age was:
30 weeks (range: 15 to 38 weeks) in the TTTS group
33 weeks (range: 16 to 38 weeks) in the non-TTTS group</td>
<td><u>Incidence of VCI</u>

Incidence of VCI between TTTS and non-TTTS twins
<table>
<tr><th></th><th>TTTS (n=304)</th><th>Non-TTTS (n=326)</th><th>P value</th></tr>
<tr><td>One fetus with VCI</td><td>112 (36.8%)</td><td>117 (35.9%)</td><td>0.886</td></tr>
<tr><td>Both foetuses with VCI</td><td>12 (3.9%)</td><td>11 (3.4%)</td><td></td></tr>
<tr><td>Overall</td><td>136/608 (22.4%)</td><td>139/652 (21.3%)</td><td>0.65</td></tr>
</table>
Overall incidence in the sample: 275/1260 (18.1%)</td>
<td><u>Small for gestational age (SGA)</u>

OR were reported by cases with and without TTTS as well as for overall analysed population.

SGA crude OR (95% CI) for SGA in one or both twins having VCI vs no twin with VCI
<table>
<tr><th></th><th>TTTS (n=304)</th><th>Non-TTTS (n=326)</th><th>P value</th></tr>
<tr><td>SGA for liveborn twins</td><td>1.68 (0.99 to 2.86)</td><td>1.63 (1.01 to 2.68)</td><td>0.951</td></tr>
<tr><td>SGA for all twins</td><td>1.61 (0.98 to 2.68)</td><td>1.80 (1.12 to 2.90)</td><td>0.754</td></tr>
</table>

OR and OR adjusted for institution for SGA in twins with VCI vs no twins without VCI
<table>
<tr><th></th><th>OR (95% CI)</th><th>OR (95% CI) adjusted for institution</th></tr>
<tr><td>VCI in one twin</td><td>1.29 (1.01 to 1.34)</td><td>1.45 (1.13 to 1.87)</td></tr>
<tr><td>VCI in both</td><td>1.18 (0.63 to</td><td>1.41 (0.75 to</td></tr>
</table></td>
</tr>
</table>

CONFIDENTIAL

<table>
<thead>
<tr><th>Study reference</th><th>Study design</th><th>Population characteristics</th><th>Prevalence and incidence outcomes</th><th>Risk or incidence of perinatal outcomes associated with VCI</th></tr>
</thead>
<tbody>
<tr>
<td></td>
<td>to June 2011 (Lopriore 2012), June 2002 to April 2006 (Lopriore 2007)

<u>Data sources</u>
Histopathological examinations of placentas. TTTS was diagnosed at ultrasound stage

<u>Country</u>
Portugal, the Netherlands

<u>Setting</u>
University Medical Center of Porto (Portugal) and Leiden (The Netherlands). Both institutes are tertiary medical centres for perinatal medicine</td>
<td></td>
<td></td>
<td>
<table>
<tr><td>twins</td><td>2.15)</td><td>2.58)</td></tr>
</table>

<u>Intrauterine fetal demise (IUFD)</u>

Crude OR for IUFD in at least one twin having VCI vs no twins with VCI (p=0.032)

1.08 (95% CI: 0.57 to 2.02) in TTTS
3.40 (95% CI: 1.51 to 8.18) in non-TTTS

<table>
<tr><th></th><th>IUFD</th><th>No IUFD</th><th>OR (95% CI)</th></tr>
<tr><td colspan="4">Non-TTTS</td></tr>
<tr><td>VCI</td><td>18 (14.1)</td><td>110 (85.9)</td><td rowspan="2">3.4 (1.48 to 7.83), p=0.004</td></tr>
<tr><td>No VCI</td><td>9 (4.6)</td><td>187 (95.4)</td></tr>
<tr><td colspan="4">TTTS</td></tr>
<tr><td>VCI</td><td>20 (16.1)</td><td>104 (83.9)</td><td rowspan="2">1.08 (0.58 to 2.03), p=0.805</td></tr>
<tr><td>No VCI</td><td>27 (15.1)</td><td>152 (84.9)</td></tr>
<tr><td colspan="4">Overall</td></tr>
<tr><td>VCI n=252</td><td>38 (15.1)</td><td>214</td><td rowspan="2">1.67 (1.03 to 2.72, p=0.039)</td></tr>
<tr><td>No VCI n=375</td><td>36 (9.6)</td><td>339</td></tr>
</table>
IUFD OR and OR adjusted for centre

<u>Neonatal mortality</u>
Crude OR for neonatal mortality in at least one twin having VCI vs no twins with VCI (p=0.564)

1.05 (95% CI: 0.43 to 2.44) in TTTS
1.70 (95% CI: 0.39 to 7.33) in non-TTTS

OR and OR adjusted for centre for neonatal mortality in twins with VCI vs twins without VCI
<table>
<tr><th></th><th>OR (95% CI)</th><th>OR (95% CI) adjusted for</th></tr>
</table>
</td>
</tr>
</tbody>
</table>

CONFIDENTIAL

Study reference	Study design	Population characteristics	Prevalence and incidence outcomes	Risk or incidence of perinatal outcomes associated with VCI

Risk or incidence of perinatal outcomes associated with VCI (continued):

		centre
VCI in one twin	1.26 (0.65 to 2.43)	1.21 (0.62 to 2.32)
VCI in both twins	0.79 (0.04 to 3.93)	0.74 (0.04 to 3.68)

Gestational age

Crude regression coefficient for shorter gestational age in at least one twin having VCI vs no twins with VCI (p=0.014)

- -0.57 (-1.69 to 0.55) in TTTS
- -2.30 (-3.15 to -1.46) in non-TTTS

Gestational age in twins with and without VCI in TTTS and non-TTTS twins, weeks (SD)

	TTTS	Non-TTTS
VCI	31.0 (4.7)	34.4 (2.8)
No VCI	30.4 (5.6)	32.1 (4.9)

Severe birth weight discordance and Quintero stage also reported.

No additional outcomes were reported in Lopriore 2007

Population characteristics:

Patient recruitment methodology

All consecutive birth weight discordant placentas were included. The control pregnancies were the next MC twin pregnancy delivered at a matched gestational age (±1 week gestation) that was not associated with BWD (inter-twin BWD was defined as a difference in birth weight >25% and was calculated using the following formula: (birth weight larger twin-birth weight smaller twin)/birth weight larger twin x 100%). MC placentas from twins delivered at other centres and sent to our institution for detailed investigation were also included in this study.

Cases with TTTS or twin-anemiapolycythemia sequence (TAPS), triplets (or higher order), fetal

Prevalence and incidence outcomes:

VCI incidence in BWD twins

Incidence and risk of BWD in VCI and non-VCI cases

	BWD (n=47)	No BWD (n=47)	OR (95% CI)
VCI	49	33	*2.01 (1.12 to 3.62), p=0.019*
No VCI	45	61	

CONFIDENTIAL

<table>
<tr><th>Study reference</th><th>Study design</th><th>Population characteristics</th><th>Prevalence and incidence outcomes</th><th>Risk or incidence of perinatal outcomes associated with VCI</th></tr>
<tr><td></td><td></td><td>demise and/or damaged placentas were excluded.

<u>Sample size</u>
47 birth weight discordant twins and 47 controls

<u>Baseline characteristics</u>
None reported</td><td></td><td></td></tr>
<tr><td>Costa-Castro 2016[53]</td><td><u>Design</u>
Retrospective cohort

<u>Objective</u>
To estimate the prevalence of velamentous cord insertion (VCI) in dichorionic (DC) and monochorionic (MC) twins with and without twin-twin transfusion syndrome (TTTS), and to study the associated outcomes

<u>Dates</u>
January 2005 to September 2015

<u>Data sources</u>
Histopathological examinations of placentas. TTTS was diagnosed at</td><td><u>Patient recruitment methodology</u>
All consecutive placentas of MC and DC twins examined in the time period at these institutions were included. Cases (n=53) where data could not be recorded completely were excluded

<u>Sample size</u>
1,498 placentas

<u>Baseline characteristics</u>
None reported</td><td><u>Incidence of VCI</u>

Incidence of VCI between DC twins, MC twins with TTTS and MC twins without TTTS
<table>
<tr><th></th><th>DC (n=550)</th><th>MC Non-TTTS (n=513)</th><th>MC TTTS (n=435)</th></tr>
<tr><td>VCI in one fetus, n (%)</td><td>42 (7.6)</td><td>178 (34.7)</td><td>157 (36.1)</td></tr>
<tr><td>VCI in both fetuses, n (%)</td><td>4 (0.7)</td><td>12 (2.3)</td><td>17 (3.9)</td></tr>
<tr><td>Overall (%)</td><td>46 (8.4)</td><td>190 (37.0)</td><td>174 (40.0)</td></tr>
</table>
P<0.001

Overall incidence in the sample: 410/1498 (27.4%)
Overall MCDA twins: 364/948 (38.4%) per pregnancy and 393/1896 (20.7%) per insertion</td><td>Raw numbers for SGA, IUFD and neonatal mortality were not reported specifically for VCI cases. However, crude odds rations were calculated for interaction between the outcome and VCI

<u>Small for gestational age (SGA)</u>

Crude OR for SGA between twins with and without VCI was reported for N=1490 twins (overall interaction p=0.266)
• 0.96 (0.52 to 1.83) in DC twins (n=550)
• 1.75 (1.19 to 2.59) in MC non-TTTS twins (n=524)
• 1.66 (1.10 to 2.53) in MC TTTS twins (n=416)

Crude OR for SGA cases calculated without IUFD cases between twins with and without VCI (overall interaction p=0.329)
• 0.94 (0.50 to 1.80) in DC twins (n=550)
• 1.66 (1.12 to 2.50) in MC non-TTTS twins (n=524)
• 1.50 (0.96 to 2.34) in MC TTTS twins (n=416)

<u>Intrauterine fetal demise (IUFD)</u>

Crude OR for IUFD between twins with and without VCI was reported for N=1539 twins (overall interaction p value = 0.291).
• 2.24 (0.34 to 8.84) in DC twins (n=551)
• 2.71 (1.38 to 5.47) in MC non-TTTS twins (n=533)
• 1.39 (0.83 to 2.31) in MC TTTS twins (n=455)</td></tr>
</table>

CONFIDENTIAL

Study reference	Study design	Population characteristics	Prevalence and incidence outcomes	Risk or incidence of perinatal outcomes associated with VCI
	ultrasound stage<br><br>Country<br>Portugal, the Netherlands<br><br>Setting<br>Sao Joao Hospital Center (Portugal) and the Leiden University Medical Center (The Netherlands). Both institutes are tertiary medical centres for perinatal medicine.			Neonatal mortality<br>Crude OR for neonatal mortality between twins with and without VCI was calculated for N=1398 (overall interaction p value = 0.486)<br>• 0 (0 to 3.71) in DC twins (n=534)<br>• 2.17 (0.77 to 6.29) in MC non-TTS twins (n=495)<br>• 1.03 (0.47 to 2.19) MC TTTS twins (n=366)<br><br>Gestational age<br>Crude regression coefficient for shorter gestational age in at least one twin having VCI vs no twins with VCI (p=0.148)<br>• -1.91 (-3.29 to -0.53) in DC twins<br>• -1.93 (-2.77 to -1.10) in MC non-TTTS twins<br>• -0.85 (-1.78 to 0.07) in MC TTTS twins<br>•<br>Crude OR for severe birth weight discordance also reported.
**De Paepe 2010a,[51] De Paepe 2010b[52]**<br><br>Relevant to Q6	Design<br>Prospective cohort<br><br>Dates<br>2001 to 2009 (De Paepe 2010a)<br>2001 to 2008 (De Paepe 2010b)<br><br>Objective<br>The aim of this study was to characterize the placental markers of selective birth weight	Patient recruitment methodology<br>A prospective cohort of 319 consecutive diamniotic/monochorionic twin placentas was examined at Women and Infants Hospital between 2001 and 2009. TTTS, triplet or quadruplet placentas (14), monochorionic-monoamniotic placentas (12), placentas of gestations complicated by twin reversed arterial perfusion (TRAP) sequence (2), placentas with marked disruption of the chorionic plate (7) and cases with unknown birth weights (12) were excluded<br><br>Sample size<br>216 placentas (36 birth weight (BW)-discordant and 180 BW-concordant)<br><br>Baseline characteristics<br>The gestational age at delivery was BW-discordant twins 33.4 (SD 3.2, range 25 to	VCI incidence (De Paepe 2010a)<br>16/72 (22%) in BW-discordant twin placentas<br>29/360 (8%) in BW-concordant twin placentas<br>P<0.001<br><br>*Overall incidence was 45/432 (10.4%) per insertion*	No VCI-specific outcomes were reported

CONFIDENTIAL

Study reference	Study design	Population characteristics	Prevalence and incidence outcomes	Risk or incidence of perinatal outcomes associated with VCI
	discordance, with special emphasis on the choriovascular architecture<br><br>Data sources<br>Studied the placental characteristics and angioarchitecture of the placentas. Reviewed accompanying charts<br><br>Country<br>USA<br><br>Setting<br>Women and Infants Hospital	38) weeks<br>BW-concordant twins 34.9 (SD 3.4, range 23 to 40) weeks<br>P<0.02 for gestational age at delivery between BW-discordant and BW-concordant twins		
		Patient recruitment methodology<br>A cohort of 284 consecutive monochorionic placentas was included. Triplet and quadruplet placentas (11), monochorionic–monoamniotic placentas (11), placentas of gestations complicated by twin reversed arterial perfusion (TRAP) sequence (2) and placentas with remote (>48 h prior to delivery) fetal demise of one twin (7) and laser-coagulated TTTS placentas (29) were excluded.<br><br>Sample size<br>224 placentas, 24 TTTS and 200 non-TTTS<br><br>Baseline characteristics<br>The gestational age at delivery was<br>TTTS twins 27.3 (SD 3.8, range 20 to 33) weeks<br>Non-TTTS twins 34.5 (SD 3.8, range 21 to 42) weeks<br>P<0.01 for gestational age at delivery between TTTS and non-TTTS twins	VCI incidence<br>14/42 (33%) in TTTS placentas<br>41/394 (10%) in non-TTTS placentas<br>P<0.0001 for VCI incidence between TTTS and non-TTTS twins<br><br>*Overall incidence 55/436 (12.6%) per insertion*	No VCI-specific outcomes were reported
**De Paepe 2011**[4]<br><br>Relevant to Q6	Design<br>Prospective cohort<br><br>Dates<br>2009 to early 2011	Patient recruitment methodology<br>A prospective cohort of 138 monochorionic placentas was examined at Women and Infants Hospital between 2009 and early 2011. Those excluded were placentas from higher multiple births (5), monochorionic-monoamniotic placentas (5), placentas with remote (>48 h prior to delivery) fetal demise of one twin (3) and placentas with marked disruption of the	VCI incidence<br>Per twin pregnancy:<br>29/103 *(28.2%)* VCI cases in one twin only<br>2/103 *(1.9%)* in both twins<br><br>*33/206 (16.0%) of cord insertions*	The only VCI-specific outcomes reported were the median number of perforating chorionic arteries (PCAs), the surface area of each individual vascular territory and the PCA density

CONFIDENTIAL

<table>
<tr><th>Study reference</th><th>Study design</th><th>Population characteristics</th><th>Prevalence and incidence outcomes</th><th>Risk or incidence of perinatal outcomes associated with VCI</th></tr>
<tr><td></td><td><u>Objective</u>
To study the association between type of cord insertion and choriovascular distribution of both twin territories in diamniotic-monochorionic twin placentas.

<u>Data sources</u>
Examination of the placentas and dividing membranes.

<u>Country</u>
USA

<u>Setting</u>
Women and Infants Hospital</td><td>chorionic plate (4) were excluded from this study. In addition, placentas from pregnancies with TTTS (18). A total of 35/138 (25%) cases were excluded.

<u>Sample size</u>
103 diamniotic-monochorionic placentas (206 twin territories)

<u>Baseline characteristics</u>
None reported</td><td></td><td></td></tr>
<tr><td>Ebbing 2013,[1] Ebbing 2015[54]</td><td><u>Design</u>
Retrospective cohort

<u>Objective</u>
To assess whether anomalous cord insertion is associated with risk of</td><td><u>Patient recruitment methodology</u>
All singleton births in Norway with gestational age >16 and <45 weeks. Statistical analysis was only performed on vaginal births.

<u>Sample size</u>
738,443 singleton pregnancies

<u>Baseline characteristics</u>
None reported</td><td><u>VCI incidence</u>

VCI incidence by maternal age, parity and overall<table>
<tr><th>Risk factor</th><th>VCI, n</th><th>No VCI, n</th></tr>
<tr><td colspan="3">Maternal age</td></tr>
<tr><td><19</td><td>253</td><td>16,942</td></tr>
<tr><td>20 to 24</td><td>1,582</td><td>108,044</td></tr>
</table></td><td><u>Pre-eclampsia</u>

Risk of pre-eclampsia in VCI vs non-VCI cases<table>
<tr><th></th><th>No pre-eclampsia</th><th>Pre-eclampsia</th><th>OR (95% CI)</th></tr>
<tr><td>VCI n=11,407</td><td>10,768</td><td>639</td><td rowspan="2">1.61 (1.49 to 1.75),</td></tr>
<tr><td>No VCI</td><td>701,24</td><td>25,792</td></tr>
</table></td></tr>
</table>

CONFIDENTIAL

Study reference	Study design	Population characteristics	Prevalence and incidence outcomes	Risk or incidence of perinatal outcomes associated with VCI

Study design (continued)

complications in the third stage of labour

Dates
1999 to 2011 (Ebbing 2015), 1999 to 2009 (Ebbing 2013)

Data sources
The Medical Birth Register of Norway (registration is compulsory). Placental examination was performed by the attending midwife or physician. The information regarding cord insertion has been specified since 1999 using tick boxes labelled as 'normal,' 'marginal,' 'velamentous' and 'vessel anomalies'

Country
Norway

Setting
Norwegian Medical Birth Register

Prevalence and incidence outcomes (continued)

25 to 29	3,452	*237,106*
30 to 34	3,758	*240,194*
35 to 39	1,975	*106,137*
>40	387	*18,613*
Overall	11,407 (1.5%)	
Parity		
0	5,155	324,568
1	3,875	278,048
2	1,701	126,797
3	463	2,111
≥4	213	16,960

"No VCI" numbers calculated by subtracting "VCI" from "Total"

Risk or incidence of perinatal outcomes associated with VCI (continued)

n=727,036	*4*		*p<0.0001*

No VCI cases calculated by subtracting the VCI numbers from "All" in Table 1

Preterm birth
Preterm birth was defined as birth occurring before gestational week 37

Risk of preterm birth in VCI vs non-VCI singletons

	No pre-term birth	Pre-term birth	OR (95% CI)
VCI	10,075	1332	*2.13 (2.01 to 2.25), p<0.0001*
No VCI	*684,507*	*42,529*	

No VCI cases calculated by subtracting the VCI numbers from "All" in Table 1

Population characteristics

Ebbing 2013

Sample size
11,263 pairs of twins; 623,478 singletons

Baseline characteristics
None reported

Prevalence and incidence outcomes

Ebbing 2013

VCI incidence in twins
5.9%, OR 4.0, 95% CI 3.7 to 4.3

VCI incidence in singletons by risk factors that was not included in Ebbing 2015

Risk of VCI between depending on pregnancy originating from assisted reproductive technologies

	No assisted reproductive technologies	Assisted reproductive technologies	OR (95% CI)
VCI	9127	373	2.16 (1.94 to 2.41)
No VCI	*604,143*	*9,835*	

Calculated OR: 2.51 (2.26 to 2.79), p<0.0001

Incidence of VCI by fetal gender, smoking, previous CS, vaginal bleeding in pregnancy, presence of rheumatoid arthritis, asthma, chronic hypertension,

Risk or incidence of perinatal outcomes associated with VCI

Ebbing 2013 – risk of additional outcomes not reported by Ebbing 2015. All reported ORs were adjusted for maternal age and parity.

Number of cases unaffected by outcome was calculated by subtracting the number of affected cases from the number of total cases

Admission to neonatal care unit (NICU)

Risk of admission to NICU in VCI vs non-VCI singletons

	No NICU admission	NICU admission, n (%)	OR (95% CI)
VCI	8,141	1,359 (14.3)	1.83 (1.72 to 1.94)
No VCI	*563,786*	50,192 (8.18)	

Calculated OR: 1.88, 95% CI 1.77 to 1.99, p<0.0001

Low Apgar Score

CONFIDENTIAL

<table>
<tr><th>Study reference</th><th>Study design</th><th>Population characteristics</th><th>Prevalence and incidence outcomes</th><th>Risk or incidence of perinatal outcomes associated with VCI</th></tr>
<tr><td></td><td></td><td></td><td>diabetes, epilepsy in the mother or the mother taking folate or multivitamin supplementation also reported</td><td>
5 min Apgar scores <7 were considered low.

Risk of low 5 min Apgar scores for VCI vs non-VCI singletons
<table>
<tr><th></th><th>Apgar ≥7</th><th>Apgar <7</th><th>OR (95% CI)</th></tr>
<tr><td>VCI</td><td>9,210</td><td>290</td><td rowspan="2">1.87 (1.66 to 2.10)</td></tr>
<tr><td>No VCI</td><td>604,086</td><td>9,892</td></tr>
</table>

<u>Small for gestational age (SGA)</u>
SGA was defined as birth weight below the 10th centile and no further details given.

Risk of SGA for VCI vs non-VCI singletons
<table>
<tr><th></th><th>Not SGA, n</th><th>SGA, n (%)</th><th>OR (95% CI)</th></tr>
<tr><td>VCI</td><td>6,438</td><td>1,050 (14.0)</td><td rowspan="2">1.88 (1.76 to 2.01)</td></tr>
<tr><td>No VCI</td><td>430,714</td><td>36,768 (7.9)</td></tr>
</table>

<u>Perinatal death</u>

Risk of perinatal death in VCI vs non-VCI singletons
<table>
<tr><th></th><th>No death, n</th><th>Death, n (%)</th><th>OR (95% CI)</th></tr>
<tr><td>VCI</td><td>9,344</td><td>156 (1.6)</td><td rowspan="2">2.14 (1.83 to 2.52)</td></tr>
<tr><td>No VCI</td><td>609,249</td><td>4,729 (0.7)</td></tr>
</table>

<u>Placental abruption</u>

Risk of placental abruption in VCI and non-VCI singletons
<table>
<tr><th></th><th>No abruptio</th><th>Abruptio n</th><th>OR (95% CI)</th></tr>
</table>
</td></tr>
</table>

CONFIDENTIAL

<table>
<thead>
<tr><th>Study reference</th><th>Study design</th><th>Population characteristics</th><th>Prevalence and incidence outcomes</th><th>Risk or incidence of perinatal outcomes associated with VCI</th></tr>
</thead>
<tbody>
<tr><td></td><td></td><td></td><td></td><td>
<table>
<tr><th></th><th>n</th><th></th><th></th></tr>
<tr><td>VCI</td><td>9,402</td><td>98</td><td rowspan="2">2.60 (2.12 to 3.18)</td></tr>
<tr><td>No VCI</td><td>611,535</td><td>2,443</td></tr>
</table>

<u>Placenta praevia</u>

Risk of placenta praevia in VCI vs non-VCI singletons
<table>
<tr><th></th><th>No placenta praevia</th><th>Placenta praevia</th><th>OR (95% CI)</th></tr>
<tr><td>VCI</td><td>9,398</td><td>102</td><td rowspan="2">3.71 (3.03 to 4.55)</td></tr>
<tr><td>No VCI</td><td>639,663</td><td>1713</td></tr>
</table>

<u>Emergency CS</u>
OR for emergency CS risk were adjusted for maternal age and parity

Risk of emergency CS in VCI vs non-VCI cases
<table>
<tr><th></th><th>No emergency CS</th><th>Emergency CS</th><th>OR (95% CI)</th></tr>
<tr><td>VCI</td><td>8,154</td><td>1,346</td><td rowspan="2">1.80 (1.69 to 1.91)</td></tr>
<tr><td>No VCI</td><td>565,148</td><td>48,830</td></tr>
</table>

Other risks (risk of VCI given VCI in previous pregnancy) and outcomes (elective CS, forceps delivery, vacuum delivery, operative vaginal delivery, beech presentation, transverse lie, serious malformations, placental weight <10th centile, birthweight to placental weight ratio <10th centile) are also reported for VCI vs non-VCI cases
</td></tr>
<tr><td>Hack 2008[46]

Relevant to</td><td><u>Design</u>
Prospective cohort</td><td><u>Patient recruitment methodology</u>
All consecutive placentas of MC diamniotic twins examined in the time period at this institution</td><td><u>VCI incidence</u>
30/150 (20%) of pregnancies.</td><td><u>Birthweight</u></td></tr>
</tbody>
</table>

CONFIDENTIAL

<table>
<tr><th>Study reference</th><th>Study design</th><th>Population characteristics</th><th>Prevalence and incidence outcomes</th><th>Risk or incidence of perinatal outcomes associated with VCI</th></tr>
<tr>
<td>Q6 and Q7</td>
<td><u>Dates</u>
January 1998 to January 2007

<u>Objective</u>
To study placental characteristics in relation to perinatal outcome in 150 pairs of monochorionic diamniotic (MCDA) twins

<u>Data sources</u>
Histopathological examination of the placentas and dividing membranes

<u>Country</u>
The Netherlands

<u>Setting</u>
University Medical Center, Utrecht</td>
<td>were included. Cases (n=3) where placentas could not be examined due to being fragmented were excluded. Each umbilical cord was labelled at delivery to identify the twin from whom it originated.

<u>Sample size</u>
150 pairs of monochorionic diamniotic twins

<u>Baseline characteristics</u>
Gestational age at delivery median (range) was 34 (14 to 40) weeks.</td>
<td></td>
<td>Mean (SD) birthweight was
• 1727 (920) g in VCI cases, and
• 2000 (916) g in non-VCI cases,
which was significantly different (p=0.04)

<u>Perinatal mortality</u>

Risk of perinatal mortality in VCI and non-VCI cases
<table>
<tr><th>VCI</th><th>Mortality, n (%)</th><th>OR (95% CI)</th></tr>
<tr><td>VCI (n= 60)</td><td>18 (30.0)</td><td rowspan="2">3.65 (1.83 to 7.28), p<0.001</td></tr>
<tr><td>No VCI (n=238)</td><td>25 (10.5)</td></tr>
</table>

Risk of birthweight discordance and TTTS in VCI and cases also reported.</td>
</tr>
<tr>
<td>Hack 2009[47]

Relevant to Q6 and Q7</td>
<td><u>Design</u>
Prospective cohort

<u>Dates</u>
January 1998 and May 2008</td>
<td><u>Patient recruitment methodology</u>
All consecutive placentas of MC twins examined in the time period at this institution were included. Each umbilical cord was labelled at delivery to identify the twin from whom it originated. Excluded five acardiac twin pregnancies, 2 pregnancies with conjoined twins, 4 higher order multiples and one pregnancy with an early termination on social</td>
<td><u>VCI incidence</u>

Overall VCI incidence was 3/84 (3.6%)

(In one placenta both umbilical cord insertions could not be evaluated.)</td>
<td><u>Perinatal mortality</u>
perinatal mortality was defined as intra-uterine death or early or late neonatal death of one or both infants

Risk of perinatal mortality between VCI and non-VCI cases
<table>
<tr><th></th><th>Mortality, n (%)</th><th>OR (95% CI)</th></tr>
</table></td>
</tr>
</table>

CONFIDENTIAL

<table>
<tr><th>Study reference</th><th>Study design</th><th>Population characteristics</th><th>Prevalence and incidence outcomes</th><th>Risk or incidence of perinatal outcomes associated with VCI</th></tr>
<tr>
<td></td>
<td><u>Objective</u>
To study placental characteristics in relation to perinatal outcome in 55 pairs of monochorionic monoamniotic (MA) twins

<u>Data sources</u>
Scanning and examination of placentas and intertwin membranes

<u>Country</u>
The Netherlands

<u>Setting</u>
4 different Dutch tertiary care centres</td>
<td>indication (mental disability), leaving 43 MA placentas for analysis.

<u>Sample size</u>
43 pairs of monochorionic twins

<u>Baseline characteristics</u>
• Median gestational age at delivery 33 weeks (range: 16–38) weeks
• Birthweight was 1702 (SD 577) g
• Birthweight of six twin pairs was severely discordant (≥20%)</td>
<td></td>
<td><table><tr><td>VCI (n=3)</td><td>1 (33.3)</td><td rowspan="2">1.75 (0.15 to 20.42), p=0.542</td></tr><tr><td>No VCI (n=81)</td><td>18 (22.2)</td></tr></table>
Incidence of VCI and risk of birthweight discordance also reported.</td>
</tr>
<tr>
<td>Hasegawa 2009a[41], Hasegawa 2009b[55]

Relevant to Q6 and Q7</td>
<td><u>Design</u>
Retrospective cohort

<u>Objective</u>
To evaluate whether various umbilical cord abnormalities, including velamentous, marginal cord</td>
<td><u>Patient recruitment methodology</u>
Consecutive vertex singleton pregnancies with and without cord abnormalities delivered between 22 and 41 weeks of gestation

<u>Sample size</u>
1,286 singletons

<u>Baseline characteristics</u>
Baseline characteristics for VCI cases:
• Maternal age, mean (SD) years: 33.1 (3.4)
• Parity, mean (SD) 0.2 (0.6)</td>
<td>Note that although 1286 pregnancies were recorded, baseline characteristics and VCI incidence are reported for 801 “analysed cases”

<u>VCI incidence</u>
13/801 (1.6%)</td>
<td><u>Low Apgar scores</u>
Low Apgar scores at 1 and 5 min were defined as ≤7.

• 2/13 (15.4%) had ≤7 Apgar scores at 1 min. OR 3.8 (95% CI 0.8 to 18.3)
• 1/13 (7.7%) had ≤7 Apgar scores at 5 min. OR 20.2 (95% CI 1.7 to 238.0). “OR was calculated between control and each cord abnormalities”

<u>Emergency CS</u>
• 1/13 (7.7%) VCI cases were delivered by emergency CS.</td>
</tr>
</table>

CONFIDENTIAL

<table>
<tr><th>Study reference</th><th>Study design</th><th>Population characteristics</th><th>Prevalence and incidence outcomes</th><th>Risk or incidence of perinatal outcomes associated with VCI</th></tr>
<tr>
<td></td>
<td>insertion (VCI, MCI), hypercoiled cord (HCC) and nuchal cord (NC), affect the appearance of atypical variable deceleration (VD) during labour Hasegawa 2009b objective was to examine differences in intrapartum fetal heart rate (FHR) patterns among cases with various cord abnormalities

<u>Dates</u>
June 2005 to December 2006

<u>Data sources</u>
Medical records where obstetricians provide detailed notes on their post-delivery examination of placentas

<u>Country</u>
Japan

<u>Setting</u>
Showa University</td>
<td>Baseline characteristics in controls cases (characteristics for cases with marginal, hypercoiled and nuchal cord were reported separately):
• Maternal age, mean (SD) years: 32.8 (5.2)
• Parity, mean (SD) 0.7 (0.7)

The authors report that the groups were similar, but it is unclear if this was statistically tested.</td>
<td></td>
<td>• OR (95% CI) for delivery by emergency CS in VCI vs non-VCI cases was 2.4 (0.3 to 20.1)

<u>Fetal heart rate (FHR) patterns</u>
In intrapartum FHR observation, non-reassuring fetal status (NRFS) was diagnosed when severe variable decelerations, prolonged decelerations, recurrent late decelerations, diminishing of baseline variability, or fetal bradycardia occurred. The National Institute of Child Health and Human Development guidelines were applied for FHR interpretation

Control cases do not include 301 cases with cord abnormalities other than VCI, which were reported separately

Frequencies of atypical and pure variable decelerations (VD) in the first stage of labour, per uterine contraction
<table>
<tr><th>All values % (SD)</th><th>Controls (n=487)</th><th>VCI (n=13)</th></tr>
<tr><td>Total VD</td><td>7.6 (12.3)</td><td>24.5 (19.5)*</td></tr>
<tr><td>Atypical VD</td><td>4.3 (9.3)</td><td>20.8 (18.3)*</td></tr>
<tr><td>Pure VD</td><td>3.3 (7.2)</td><td>3.7 (5.5)</td></tr>
<tr><td>Loss of variability during VD</td><td>0.01 (0.3)</td><td>0.6 (2.1)</td></tr>
<tr><td>VD with persistent secondary acceleration</td><td>0.1 (0.9)</td><td>0 (0)</td></tr>
<tr><td>Slow return of VD to baseline</td><td>0.4 (1.9)</td><td>1.2 (2.4)</td></tr>
<tr><td>VD without acceleration</td><td>3.0 (7.1)</td><td>18.7 (19.0)*</td></tr>
<tr><td>Biphasic VD</td><td>0.1 (1.0)</td><td>0.3 (1.1)</td></tr>
</table>
* P<0.01 by ANOVA in control and each cord abnormality, with Bonferroni post-hoc test

Frequencies of atypical and pure variable</td>
</tr>
</table>

CONFIDENTIAL

<table>
<thead>
<tr><th>Study reference</th><th>Study design</th><th>Population characteristics</th><th>Prevalence and incidence outcomes</th><th>Risk or incidence of perinatal outcomes associated with VCI</th></tr>
</thead>
<tbody>
<tr>
<td rowspan="2"></td>
<td rowspan="2">Hospital</td>
<td></td>
<td></td>
<td>decelerations (VD) in the second stage of labour, per uterine contraction
<table>
<tr><th>All values % (SD)</th><th>Controls (n=471)</th><th>VCI (n=12)</th></tr>
<tr><td>Total VD</td><td>37.8 (31.7)</td><td>55.2 (25.9)</td></tr>
<tr><td>Atypical VD</td><td>21.8 (25.6)</td><td>34.6 (19.8)</td></tr>
<tr><td>Pure VD</td><td>16.0 (22.8)</td><td>20.6 (28.3)</td></tr>
<tr><td>Loss of variability during VD</td><td>0.1 (1.7)</td><td>2.4 (8.2)*</td></tr>
<tr><td>VD with persistent secondary acceleration</td><td>2.2 (7.5)</td><td>1.7 (5.8)</td></tr>
<tr><td>Slow return of VD to baseline</td><td>3.5 (10.2)</td><td>5.0 (9.5)</td></tr>
<tr><td>VD without acceleration</td><td>14.7 (21.7)</td><td>23.2 (18.0)</td></tr>
<tr><td>Biphasic VD</td><td>1.3 (7.2)</td><td>2.4 (8.2)</td></tr>
</table>
* P<0.01 by ANOVA in control and each cord abnormality, with Bonferroni post-hoc test</td>
</tr>
<tr>
<td><u>Patient recruitment methodology</u>
Consecutive vertex singleton pregnancies with and without cord abnormalities delivered between 37 and 41 weeks of gestation

<u>Sample size</u>
1,229 singletons

<u>Baseline characteristics</u>
Baseline characteristics for VCI cases:
Maternal age, mean (SD) years: 33.0 (3.5)
Parity, median: 0</td>
<td></td>
<td><u>Fetal heart rate (FHR) patterns</u>
Additional FHR parameters were measured and reported by Hasegawa 2009b

<table>
<tr><th>All values % (SD)</th><th>Controls (n=466)</th><th>VCI (n=12)</th></tr>
<tr><td colspan="3">First stage of labour</td></tr>
<tr><td>Early deceleration</td><td>1.4 (5.1)</td><td>1.7 (2.8)</td></tr>
<tr><td>Plate deceleration</td><td>0.7 (3.5)</td><td>1.2 (3.5)</td></tr>
<tr><td>Prolonged deceleration</td><td>0.9 (3.3)</td><td>1.8 (3.1)</td></tr>
<tr><td colspan="3">Second stage of labour</td></tr>
</table></td>
</tr>
</tbody>
</table>

CONFIDENTIAL

<table>
<tr><th>Study reference</th><th>Study design</th><th>Population characteristics</th><th>Prevalence and incidence outcomes</th><th>Risk or incidence of perinatal outcomes associated with VCI</th></tr>
<tr><td></td><td></td><td>Baseline characteristics in controls cases (characteristics for cases with marginal, hypercoiled and nuchal cord were reported separately):
• Maternal age, mean (SD) years: 32.7 (5.2)
• Parity, median: 1

The authors report that the groups were similar, but it is unclear if this was statistically tested.</td><td></td><td><table><tr><th></th><th>Controls (n=466)</th><th>VCI (n=11)</th></tr><tr><td>Early deceleration</td><td>1.0 (4.3)</td><td>1.7 (3.1)</td></tr><tr><td>Plate deceleration</td><td>0.7 (3.3)</td><td>2.9 (9.0)</td></tr><tr><td>Prolonged deceleration</td><td>12.6 (24.5)</td><td>12.0 (16.8)</td></tr></table></td></tr>
<tr><td>Hasegawa 2011[40]

Relevant to Q6</td><td><u>Design</u>
Retrospective cohort

<u>Objective</u>
To assess the usefulness for predicting vasa previa by detecting a cord insertion site in the lower third of the uterus between 9 and 13 weeks' gestation

<u>Dates</u>
2006 to 2009

<u>Data sources</u>
Medical and ultrasonographic records. Ultrasound diagnoses were made with colour Doppler transabdominal</td><td><u>Patient recruitment methodology</u>
Women who underwent ultrasonographic examination at 9 to 13 weeks of gestation and delivered at the authors' hospital.

<u>Sample size</u>
1,231 singleton, 37 twin, 2 triplet pregnancies; 1,311 cord insertion sites in 1,270 mothers were evaluated.

<u>Baseline characteristics</u>
There were no significant differences in baseline demographics between groups with different cord insertion sites.

Baseline demographics in low and normal cord insertion pregnancies<table><tr><th></th><th>Low cord insertion (n=139)</th><th>Normal cord insertion (n=1131)</th></tr><tr><td>Maternal age, years (SD)</td><td>33.4 (4.7)</td><td>32.8 (4.6)</td></tr><tr><td>Gravida, median (range)</td><td>1 (0 to 5)</td><td>1 (0 to 8)</td></tr></table></td><td>Groups of low or normal cord insertion were based on the diagnosis at 9 to 13 weeks gestation, where cords located in the upper and middle thirds of the distance between the os and the fundus were classified as normal and cord located in the lower third were classified as low.

<u>VCI incidence</u>
There were:
• 10/139 (7.2%) VCI cases in low cord insertion group
• 11/1,172 (0.9%) VCI cases in normal cord insertion group
• The relative risk ratio (95% CI) for VCI (calculated between normal and low cord insertion) was 8.1 (3.4 to 19.6), p<0.001</td><td>No VCI-specific outcomes reported</td></tr>
</table>

CONFIDENTIAL

Study reference	Study design	Population characteristics	Prevalence and incidence outcomes	Risk or incidence of perinatal outcomes associated with VCI
	and transvaginal ultrasound and confirmed after delivery by pathological examination<br><br>Country<br>Japan<br><br>Setting<br>Showa University Hospital	Parity. Median (range) / 0 (0 to 3) / 0 (0 to 4)		
**Kent 2011**[48] (ESPRiT)<br><br>Relevant to Q6 and Q7	Design<br>Prospective cohort<br><br>Objective<br>To evaluate the impact of noncentral placental cord insertion on birthweight discordance in twins.<br><br>Dates<br>May 2007 to October 2009<br><br>Data sources<br>Data recorded at time of ultrasonography, delivery and outcome occurrence	Patient recruitment methodology<br>Twin pregnancies with 2 viable fetuses that were identified from 11 to 22 weeks' gestation were eligible for inclusion. Monoamniotic twins, twins with chromosomal abnormalities in either twin or cases where 1 or both fetuses had an intrauterine fetal demise before 24 weeks of gestation were excluded<br><br>Sample size<br>816 twin pregnancies with cord insertion site confirmed at delivery<br><br>Baseline characteristics<br>None reported	VCI incidence<br>• 31/330 (9.4%) in monochorionic twins<br>• 46/1,302 (3.5%) in dichorionic twins<br><br>Overall incidence<br>77/1,632 (4.7%)<br><br>Incidence stratified by TTTS in monochorionic twins<br>• 2/13 TTTS cases had VCI<br>• 29/152 non-TTTS cases had VCI	Percentage of birthweight concordant as well as lighter and heavier birthweight-discordant twins in VCI cases reported on graph<br><br>Small for gestational age (SGA)<br>• 4/31 (12.9%) of monochorionic twins with VCI were SGA (OR 4.0, 95%CI 1.1 to 14.3, p=0.02)<br>• 5/45 (11.1%) of dichorionic twins with VCI were SGA (OR 1.7, 95% CI 0.6 to 4.4, p=0.26)<br>• Overall OR 2.02, 95%CI 0.9 to 4.2, p=0.05

CONFIDENTIAL

Study reference	Study design	Population characteristics	Prevalence and incidence outcomes	Risk or incidence of perinatal outcomes associated with VCI
	(prospective study)<br><br><u>Country</u><br>Ireland<br><br><u>Setting</u><br>8 tertiary obstetric units			
**Lepais 2014**[72]<br><br>Relevant to Q6	<u>Design</u><br>Retrospective case-control<br><br><u>Objective</u><br>To test the hypothesis that placental fetal thrombotic vasculopathy (FTV) is associated with obstetric complications and predisposes the child to unfavourable outcomes<br><br><u>Dates</u><br>2005 to 2009<br><br><u>Data sources</u><br>Database of the hospital's pathology department and hospitals clinical and laboratory database	<u>Patient recruitment methodology</u><br>Placentas with fetal thrombotic vasculopathy for which follow-up to 3 years was available.<br><br><u>Sample size</u><br>54 cases with fetal thrombotic vasculopathy and 100 controls<br><br><u>Baseline characteristics</u><br>46 singletons and 4 dichorionic-diamniotic twins	<u>VCI incidence</u><br>• 4/54 (7.40%) in fetal thrombotic vasculopathy<br>• 1/100 (1%) in control cases	<u>OR (95% CI) for fetal thrombotic vasculopathy in VCI and non-VCI cases</u><br>8.154 (0.876 to 75.933), p=0.0653

CONFIDENTIAL

<table>
<tr><th>Study reference</th><th>Study design</th><th>Population characteristics</th><th>Prevalence and incidence outcomes</th><th>Risk or incidence of perinatal outcomes associated with VCI</th></tr>
<tr><td></td><td><u>Country</u>
France

<u>Setting</u>
Croix-Rousse Hospital</td><td></td><td></td><td></td></tr>
<tr><td>McNamara 2014[42]

Relevant to Q6</td><td><u>Design</u>
Retrospective cohort

<u>Objective</u>
To identify maternal, placental, and umbilical cord determinants of placental weight, before and after accounting for birthweight

<u>Dates</u>
1978 to 2007

<u>Data sources</u>
McGill Obstetric and Neonatal Database that contains the obstetrical and neonatal medical records of all Births at the hospital

<u>Country</u>
Canada</td><td><u>Patient recruitment methodology</u>
The study population was drawn from singleton births with no congenital anomalies

<u>Sample size</u>
87,600 non-anomalous singleton births

<u>Baseline demographics</u>
<table>
<tr><th>Placental weight z-score</th><th><−1</th><th>−1 to +1</th><th>>+1</th></tr>
<tr><td></td><td>n (%)</td><td>n (%)</td><td>n (%)</td></tr>
<tr><td>Total</td><td>13,348 (15.2)</td><td>60,733 (69.3)</td><td>13,519 (15.4)</td></tr>
<tr><td colspan="4">Maternal age (years)</td></tr>
<tr><td><25</td><td>2,278 (16.9)</td><td>9,311 (69.2)</td><td>1,874 (13.9)</td></tr>
<tr><td>25–29</td><td>4,636 (16.2)</td><td>19,842 (69.1)</td><td>4,241 (14.7)</td></tr>
<tr><td>30–34</td><td>4,190 (14.4)</td><td>20,372 (69.9)</td><td>4,564 (15.7)</td></tr>
<tr><td>35–39</td><td>1,799 (13.8)</td><td>8,926 (68.47)</td><td>2,311 (17.7)</td></tr>
<tr><td>≥40</td><td>355 (13.9)</td><td>1,759 (68.9)</td><td>441 (17.3)</td></tr>
<tr><td colspan="4">Parity</td></tr>
<tr><td>0</td><td>7,362 (17.9)</td><td>28,259 (68.8)</td><td>5,432 (13.2)</td></tr>
<tr><td>1</td><td>4,105 (13.2)</td><td>21,847 (70.0)</td><td>5,243 (16.8)</td></tr>
<tr><td>2</td><td>1,357</td><td>7,566</td><td>1,957</td></tr>
</table></td><td><u>VCI incidence</u>
VCI incidence was reported stratified by placental weight z-score (a sex- and gestational age-specific score calculated using a day-specific internal reference based on the weights of liveborn infants with ultrasound confirmed estimates of gestational age)

• 691/60,733 placentas with a z-score -1 to 1 (used as reference to calculate the relative risk)
• 199/13,348 placentas with z-score <-1, with a relative risk (95% CI) of 1.25 (1.11 to 1.42) to reference placentas
• 154/13,519 placentas with a z-score >1, with a relative risk (95% CI) 0.96 (0.83 to 1.11) to reference placentas

Overall incidence was 1044/87,600 (1.19%)</td><td>No VCI-specific outcomes reported</td></tr>
</table>

CONFIDENTIAL

<table>
<tr><th>Study reference</th><th>Study design</th><th>Population characteristics</th><th>Prevalence and incidence outcomes</th><th>Risk or incidence of perinatal outcomes associated with VCI</th></tr>
<tr><td></td><td><u>Setting</u>
Royal Victoria Hospital, a McGill University teaching hospital</td><td><table>
<tr><td></td><td>(12.5)</td><td>(69.5)</td><td>(18.0)</td></tr>
<tr><td>3+</td><td>524
(11.7)</td><td>3,061
(68.5)</td><td>887
(19.8)</td></tr>
<tr><td colspan="4">Pre-pregnancy BMI (kg/m^2)</td></tr>
<tr><td><18.5</td><td>962
(23.6)</td><td>2,714
(66.6)</td><td>399
(9.8)</td></tr>
<tr><td>18.5 to 24.9</td><td>4,883
(16.2)</td><td>21,076
(69.7)</td><td>4,276
(14.1)</td></tr>
<tr><td>25 to 29.9</td><td>780
(11.3)</td><td>4,698
(67.9)</td><td>1,442
(20.8)</td></tr>
<tr><td>≥30</td><td>283
(9.0)</td><td>2,016
(64.0)</td><td>852
(27.0)</td></tr>
<tr><td>Missing</td><td>6,440
(14.9)</td><td>30,229
(69.9)</td><td>6,550
(15.2)</td></tr>
<tr><td>Pre-existing hypertension</td><td>282
(25.3)</td><td>677
(60.1)</td><td>158
(14.2)</td></tr>
<tr><td>Pre-eclampsia</td><td>584
(19.1)</td><td>1,978
(64.7)</td><td>498
(16.3)</td></tr>
<tr><td>Gestational diabetes</td><td>337
(10.3)</td><td>2,149
(65.4)</td><td>800
(24.4)</td></tr>
<tr><td>Pre-existing diabetes</td><td>41
(8.8)</td><td>291
(62.2)</td><td>136
(29.1)</td></tr>
<tr><td>Smoking during pregnancy</td><td>2,307
(16.7)</td><td>9,311
(68.1)</td><td>2,063
(16.9)</td></tr>
</table></td><td></td><td></td></tr>
<tr><td>Pinar 2014[57]

Relevant to Q6</td><td><u>Design</u>
Prospective case-control

<u>Objective</u>
To compare placental lesions for stillbirth cases and live birth controls in a population-based study</td><td><u>Patient recruitment methodology</u>
Eligible women were residents of five catchment areas that were defined by state and county boundaries and included the state of Rhode Island and portions of Massachusetts, Georgia, Texas, and Utah. Women were enrolled at delivery. Women delivering live births prior to 32 weeks of gestation were oversampled to ensure adequate numbers for stratified analyses: all live births delivered at 20 to 23 weeks were selected for potential enrolment, and live births between 24 to 31 weeks were selected at random using selection probabilities</td><td><u>VCI incidence</u>
VIC incidence was 5.0% in stillbirths and 1.1% in live births (OR 4.50, 95% CI 2.18 to 9.27), p<0.001

Incidence of VCI stratified by gestational age at delivery between stillbirths and livebirths<table>
<tr><th>GA</th><th>Stillbirths (%)</th><th>Live births (%)</th><th>OR (95% CI)</th></tr>
<tr><td><24 weeks</td><td>7.0</td><td>0.0</td><td><0.019*</td></tr>
<tr><td>24 to 31</td><td>2.5</td><td>6.0</td><td>0.40 (0.09</td></tr>
</table></td><td>No VCI-specific outcomes reported</td></tr>
</table>

CONFIDENTIAL

Study reference	Study design	Population characteristics	Prevalence and incidence outcomes	Risk or incidence of perinatal outcomes associated with VCI

Dates
March 2006 to September 2008

Data sources
Maternal interview, medical records, placental pathology examination, and maternal and fetal biospecimens, and for stillbirths a postmortem examination. The placental examination protocol included initial digital imaging under specified lighting, macroscopic examination, collection of frozen and ambient temperature samples of the cord, membranes and the placental disc, and microscopic examination of sections collected according to a specific sampling protocol. The examiners were

that were pre-specified by week of gestation to provide numbers similar to those for stillbirths at each gestational week. African American women delivering live births at or beyond 32 weeks of gestation were also oversampled.

Sample size
518 stillbirths and 1,200 livebirths

Baseline demographics

Baseline characteristics provided for weighted sample size (indicated by n_w)

	Stillbirth (n_w=518)	Live birth (n_w=966)
Maternal age at delivery, years (%)		
<20	74 (14.3)	100 (10.3)
20 to 34	358 (69.2)	737 (76.3)
35 to 39	62 (11.9)	106 (11.0)
40+	24 (4.6)	23 (2.4)
Maternal race/ethnicity, n (%)		
Non-Hispanic white	177 (34.2)	435 (45.0)
Non-Hispanic black	115 (22.2)	112 (11.6)
Hispanic	195 (37.8)	351 (36.4)
Other	30 (5.8)	68 (7.0)

weeks			to 1.82)
32 to 36 weeks	7.6	1.9	4.32 (0.51 to 36.31)
≥37 weeks	2.1	1.0	2.06 (0.44 to 9.68)

* an upper bound p-value provided as VCI data was absent for one of the groups

CONFIDENTIAL

<table>
<tr><th>Study reference</th><th>Study design</th><th>Population characteristics</th><th>Prevalence and incidence outcomes</th><th>Risk or incidence of perinatal outcomes associated with VCI</th></tr>
<tr><td></td><td>not blinded to stillbirth/live birth status.

Country
USA

Setting
59 hospitals in 5 US states</td><td></td><td></td><td></td></tr>
<tr><td>Raisanen 2012[43]

Relevant to Q6 and Q7</td>
<td>Design
Retrospective cohort

Objective
To identify risk factors associated with VCI and to evaluate the association between adverse pregnancy outcomes and VCI in singleton pregnancies

Dates
2000 to 2011

Data sources
Kuopio University Hospital register. Further information was gathered from self-administered paper-based questionnaires</td>
<td>Patient recruitment methodology
All women who gave birth to singleton pregnancies were included in the study. Diagnosis of VCI was clinical; weight, length and insertion of the umbilical cord were examined routinely by midwives after every delivery, either vaginal or caesarean.

Sample size
26,849 pregnancies

Baseline demographics
Baseline demographics for women with and without VCI
<table>
<tr><th></th><th>VCI (n=633)</th><th>No VCI (n=26, 216)</th><th>p value</th></tr>
<tr><td colspan="4">Maternal age, years (SD) or %</td></tr>
<tr><td>Mean maternal age</td><td>29.3 (5.7)</td><td>29.1 (5.6)</td><td>0.26</td></tr>
<tr><td><18</td><td>0.3</td><td>0.6</td><td rowspan="3">0.34</td></tr>
<tr><td>18 to 34</td><td>80.4</td><td>81.7</td></tr>
<tr><td>>35</td><td>19.3</td><td>17.6</td></tr>
<tr><td colspan="4">Pregravid BMI, %</td></tr>
<tr><td>≤24.9</td><td>62.1</td><td>66.4</td><td rowspan="3">0.008</td></tr>
<tr><td>25.0 to 29.9</td><td>21.4</td><td>21.2</td></tr>
<tr><td>30.0 to 34.9</td><td>10.1</td><td>8.4</td></tr>
</table></td>
<td>VCI incidence
The incidence of VCI was 633/26,849 (2.4%)

Incidence of VCI stratified by parity and fertility treatment
<table>
<tr><th></th><th>VCI (n= 633)</th><th>No VCI (n= 26,216)</th><th>p value</th></tr>
<tr><td colspan="4">Parity, %</td></tr>
<tr><td>Nulliparous</td><td>47.2</td><td>40.4</td><td rowspan="2">0.001</td></tr>
<tr><td>Multiparous</td><td>52.8</td><td>59.6</td></tr>
<tr><td colspan="4">Fertility, %</td></tr>
<tr><td>Infertility</td><td>7.7</td><td>3.9</td><td>≤0.001</td></tr>
<tr><td>Assisted reproductive technologies</td><td>3.5</td><td>2.5</td><td>0.17</td></tr>
<tr><td colspan="4">Other, %</td></tr>
<tr><td>Placental abruption</td><td>0.9</td><td>0.6</td><td>0.34</td></tr>
<tr><td>Pre-eclampsia</td><td>6.6</td><td>4.2</td><td>0.003</td></tr>
<tr><td>Placenta praevia</td><td>1.7</td><td>0.7</td><td>0.004</td></tr>
</table>
Assisted reproductive technologies included IVF, ICSI, frozen embryo replacement and egg donation.

Factors affecting the risk of VCI calculated by multivariate logistic regression for n=23,493 pregnancies</td>
<td>Perinatal outcomes were reported for 25,317 infants and reported as % of VCI or non-VCI cases with OR and adjusted OR (adjusted for parity, gestational age, prior termination, pregravid BMI, IUD before pregnancy, infertility, gestational diabetes mellitus, preeclampsia, placenta previa, sex, caesarean, smoking during pregnancy (>5/day), birth weight, mean umbilical cord length and umbilical cord insertion).

Admission to a neonatal unit

Incidence and risk of admission to a neonatal unit between VCI and non-VCI infants
<table>
<tr><th></th><th>%</th><th>OR (95% CI)</th><th>Adj. OR (95% CI)</th></tr>
<tr><td>VCI</td><td>17.2</td><td rowspan="2">2.12 (1.71 to 2.61), p≤0.001</td><td rowspan="2">1.38 (1.06 to 1.80), p≤0.05</td></tr>
<tr><td>No VCI</td><td>9.0</td></tr>
</table>
Low Apgar scores
Low Apgar scores were defined as 0 to 6.

Incidence and risk of low 1 min Apgar scores between VCI and non-VCI infants
<table>
<tr><th></th><th>%</th><th>OR (95% CI)</th><th>Adj. OR (95% CI)</th></tr>
<tr><td>VCI</td><td>7.7</td><td rowspan="2">1.50 (1.11 to 2.01), p≤0.01</td><td rowspan="2">0.97 (0.68 to 1.37)</td></tr>
<tr><td>No VCI</td><td>5.3</td></tr>
</table></td></tr>
</table>

CONFIDENTIAL

Study reference	Study design	Population characteristics	Prevalence and incidence outcomes	Risk or incidence of perinatal outcomes associated with VCI
	until 2008 and thereafter electronically via the internet. Information was complemented by midwife interviews during visits or at the time of delivery<br><br><u>Country</u><br>Finland<br><br><u>Setting</u><br>Kuopio University Hospital, a tertiary level perinatal centre for the area	(see below)	(see below)	(see below)

Population characteristics (continued)

≥35.0	6.4	4.0	

BMI, body mass index

Prevalence and incidence outcomes (continued)

	OR (95% CI)	p value
Nulliparous	1	0.004
Multiparous	0.78 (0.66 to 0.92)	
Prior termination	0.71 (0.53 to 0.94)	0.02
Pregravid BMI, %		
≤24.9	1	
25.0-29.9	1.12 (0.91 to 1.37)	0.30
30.0-34.9	1.32 (1.00 to 1.75)	0.06
≥35.0	1.83 (1.30 to 2.57)	0.001
Infertility	1.89 (1.38 to 2.59)	≤0.001
Placenta praevia	2.24 (1.17 to 4.27)	0.02
Smoking in pregnancy (>5 cigarettes/day)	1.34 (1.04 to 1.72)	0.02

OR adjusted for parity, prior termination, pregravid BMI, intrauterine device before pregnancy, infertility, assisted reproductive technologies, maternal smoking, gestational diabetes mellitus, placenta praevia, and pre-eclampsia.

Risk or incidence of perinatal outcomes associated with VCI (continued)

Incidence and risk of low 5 min Apgar scores between VCI and non-VCI infants

	%	OR (95% CI)	Adj. OR (95% CI)
VCI	4.9	1.93 (1.33 to 2.79), p≤0.001	0.89 (0.56 to 1.43)
No VCI	2.6		

<u>Low birth weight (LBW)</u>
LBW was defined as an infant weighing <2500g

Incidence and risk of LBW between VCI and non-VCI infants

	%	OR (95% CI)	Adj. OR (95% CI)
VCI	15.0	3.89 (3.10 to 4.88), p≤0.001	3.93 (2.75 to 5.62), p≤0.001
No VCI	4.3		

<u>Small for Gestational Age (SGA)</u>
An infant was considered to be SGA when the sex and gestational age –adjusted birth weight was below the normal tenth percentile

Incidence and risk of SGA between VCI and non-VCI infants

	%	OR (95% CI)	Adj. OR (95% CI)
VCI	14.9	1.55 (1.24 to 1.94), p≤0.001	1.39 (1.09 to 1.77), p≤0.01
No VCI	10.1		

<u>Fetal death</u>

Incidence and risk of fetal death between VCI and non-VCI infants

	%	OR (95% CI)	Adj. OR (95% CI)
VCI	0.5	1.71 (0.54 to	0.64 (0.14 to

CONFIDENTIA

Study reference	Study design	Population characteristics	Prevalence and incidence outcomes	Risk or incidence of perinatal outcomes associated with VCI
				(continued below)

Risk or incidence of perinatal outcomes associated with VCI (continued)

No VCI	0.3	5.43)	2.86)

Delivery by CS

Incidence of emergency CS in VCI and non-VCI cases

	VCI (n=633)	No VCI (n=26,216)	p value
Total CS, %	22.4	15.7	≤0.001
Elective CS, %	6.8	6.8	≤0.001
Emergency CS, %	15.3	8.7	

Study reference

Suzuki 2015[21]

Relevant to Q6 and Q7

Study design

Design
Retrospective

Objective
To examine the clinical significance of pregnancies complicated by velamentous cord insertion (VCI) associated with other umbilical cord or placental abnormalities

Dates
2002 to 2011

Data sources
Patient charts

Country
Japan

Population characteristics

Patient recruitment methodology
Women included the total population of women who gave birth to singleton babies at the study hospital between 2002 and 2011

Sample size
168 deliveries complicated by VCI and 16,797 unaffected controls

Baseline demographics

All n (%)	No VCI, n= 16,797	VCI, n= 168	OR (95% CI)
Maternal age ≥ 35 years	5,755 (34.3)	63 (37.5)	OR NR, p=0.37
Nulliparity	8,655 (51.5)	114 (67.8)	1.99 (1.4 to 2.8) p<0.01
IVF use	900 (5.4)	36 (21.4)	4.82 (3.3 to 7.0) p<0.01
Maternal smoking	357 (2.1)	9 (5.4)	2.61 (1.3 to 5.1) p<0.01
Hypertensive disorders	702 (4.2)	18 (10.7)	2.75 (1.7 to 4.5)

Prevalence and incidence outcomes

VCI incidence
168/16,797 (1.0%)

Using a multivariate analysis, VCI was associated with the following as risk factors:
- IVF use (adjusted OR 2.01; 95% CI: 1.2 to 3.3; p<0.01)
- maternal smoking (adjusted OR: 1.43; 95% CI: 1.0 to 2.3; p=0.03)

VCI incidence stratified by risk factors

All n (%)	No VCI, n= 16,797	VCI, n= 168	P value	Adj. OR (95% CI)
Succenturiate placenta	112 (0.7)	2 (1.2)	0.41	
Lobed placenta	21 (0.1)	3 (1.8)	0.01	4.70 (1.0 to 18)
Placenta praevia	164 (1.0)	5 (3.0)	0.03	3.25 (0.91 to 5.5)
Low-lying placenta	105 (0.6)	3 (1.8)	0.06	

Adj., adjusted

Risk or incidence of perinatal outcomes associated with VCI

Apart from SGA and preterm delivery, multivariate regression analysis also revealed that VCI is a risk factor for fetal asphyxia (adjusted OR: 1.47; 95% CI: 1.0 to 2.6; p=0.01).

Small for gestational age (SGA)
SGA infants were defined as those with sex- and aged-adjusted birth heights and weights below and over the 10th percentile according to the fetal growth curve for Japanese

Risk of SGA in VCI and non-VCI cases

	No VCI, n= 16,797	VCI, n= 168	Adj. OR (95% CI)
SGA, n (%)	936 (5.6)	22 (13.1)	1.68; 95% CI: 1.1 to 2.9; p=0.02
No SGA, n (%)	*15,861 (94.4)*	*146 (86.9)*	

Intrauterine fetal demise (IUFD)

Risk of IUFD in VCI and non-VCI cases

CONFIDENTIAL

Study reference	Study design	Population characteristics	Prevalence and incidence outcomes	Risk or incidence of perinatal outcomes associated with VCI
	<u>Setting</u><br>Japanese Red Cross Katsushika Maternity Hospital			

			p<0.01

	No VCI, n= 16,797	VCI, n= 168	OR (95% CI)
IUFD, n (%)	124 (0.7)	3 (1.8)	*2.44; 0.77 to 7.76; p=0.13*
No IUFD, n (%)	*16,673 (99.3)*	*165 (98.2)*	

<u>Placental abruption</u>

Risk of placental abruption in VCI and non-VCI cases

	No VCI, n= 16,797	VCI, n= 168	OR (95% CI)
Placental abruption, n (%)	101 (0.6)	5 (3.0)	5.07; 95% CI: 2.0 to 13; p<0.01
No placental abruption, n (%)	*16,696 (99.4)*	*163 (97.0)*	

<u>Pre-term delivery</u>

Risk of pre-term delivery in VCI and non-VCI cases

	No VCI, N= 16,797	VCI, N= 168	Adj. OR (95% CI)
Pre-term delivery, n (%)	1,349 (8.0)	37 (22.0)	1.74; 95% CI: 1.1 to 2.8; p=0.03
Term delivery, n (%)	*15,448 (92.0)*	*131 (78.0)*	

<u>Emergency CS</u>

Risk of emergency CS in VCI and non-VCI cases

CONFIDENTIAL

<table>
<tr><th>Study reference</th><th>Study design</th><th>Population characteristics</th><th>Prevalence and incidence outcomes</th><th>Risk or incidence of perinatal outcomes associated with VCI</th></tr>
<tr><td></td><td></td><td></td><td></td><td><table>
<tr><th></th><th>No VCI, n= 16,797</th><th>VCI, n= 168</th><th>OR (95% CI)</th></tr>
<tr><td>Emergency CS, n (%)</td><td>1,439 (8.6)</td><td>29 (17.3)</td><td rowspan="2">2.23; 95% CI: 1.5 to 3.3; p<0.01</td></tr>
<tr><td>No emergency CS, n (%)</td><td>15,358 (91.4)</td><td>139 (82.7)</td></tr>
</table>
Incidence of fetal and neonatal asphyxia, excessively long umbilical cord, single umbilical artery, circumvallate placenta, and placenta accreta in VCI and non-VCI cases also reported.
P-values for association between Vasa praevia, single umbilical artery, lobed placenta and placenta praevia in VCI pregnancies also reported.</td></tr>
<tr><td>Walker 2012[73]

Relevant to Q6</td><td><u>Design</u>
Retrospective case-control

<u>Objective</u>

<u>Dates</u>
2000 to 2010

<u>Data sources</u>
Placental pathology records

<u>Country</u>
Canada

<u>Setting</u>
Major Provincial perinatal centre</td><td><u>Patient recruitment methodology</u>
Inclusion criteria for cases of severe placental disease in this gestational age window were singleton structurally and chromosomally normal pregnancies (live birth or stillbirth) with either severe intrauterine growth restriction (IUGR) defined as absent or reversed umbilical artery flow by Doppler ultrasonography before delivery and newborn birth weight <10th percentile for gestational age and sex, pure severe pre-eclampsia as per the American College of Obstetricians and Gynecologists (ACOG) definition, or both. Multiple pregnancies were excluded

<u>Sample size</u>
262 cases affected by pre-eclampsia and/or IUGR and 40 controls

<u>Baseline demographics</u>

Baseline demographics reported for 262 cases and stratified by neonatal gender</td><td><u>VCI incidence</u>
13/140 (9.5%) VCI occurrences in male cases
2/122 (1.7%) VCI occurrences in females cases
0/40 cases in the control group
Relative Risk (95% CI) 5.66 (1.30 to 24.6)

Calculated incidence in the pre-eclampsia/IUGR group was 15/262 (5.7%)</td><td>No VCI-specific outcomes reported</td></tr>
</table>

CONFIDENTIAL

<table>
<tr><th>Study reference</th><th>Study design</th><th>Population characteristics</th><th>Prevalence and incidence outcomes</th><th>Risk or incidence of perinatal outcomes associated with VCI</th></tr>
<tr><td></td><td></td><td><table>
<tr><th></th><th>Males (n=140)</th><th>Females (n=122)</th></tr>
<tr><td>Maternal age, median (range)</td><td>31 (16 to 43)</td><td>32 (18 to 44)</td></tr>
<tr><td>Primigravida, n (%)</td><td>58 (41.4)</td><td>46 (37.8)</td></tr>
</table></td><td></td><td></td></tr>
<tr><td>Yerlikaya 2016[44]

Relevant to Q6 and Q7</td><td><u>Design</u>
Retrospective case-control

<u>Objective</u>
To evaluate obstetric outcomes and results of uterine and umbilical artery Doppler flowmetry associated with VCI

<u>Dates</u>
January 2003 to December 2013

<u>Data sources</u>
Charts, PIA Fetal Database software

<u>Country</u>
Austria

<u>Setting</u>
Department of Obstetrics and</td><td><u>Patient recruitment methodology</u>
All singleton cases with VCI reported during the time period were included. Majority of VCI cases were detected during the inspection of the placenta.

<u>Sample size</u>
108 VCI cases and 108 matched controls

<u>Baseline demographics</u>
Baseline demographics between VCI cases and matched controls<table>
<tr><th></th><th>VCI n=108</th><th>No VCI n=108</th><th>P value</th></tr>
<tr><td>Maternal age, years (SD)</td><td>31.0 (6.1)</td><td>31.4 (6.7)</td><td>0.165</td></tr>
<tr><td>BMI, kg/m2 (SD)</td><td>24.5 (5.7)</td><td>25.5 (6.3)</td><td>0.603</td></tr>
<tr><td>Gravidity, mean (SD)</td><td>2.7 (1.7)</td><td>2.8 (1.6)</td><td>0.601</td></tr>
<tr><td>Parity, mean (SD)</td><td>1.1 (1.2)</td><td>2.1 (1.1)</td><td><0.001</td></tr>
<tr><td>IVF, n (%)</td><td>8 (7.4)</td><td>3 (2.8)</td><td>0.214</td></tr>
<tr><td>Smoking during pregnanc</td><td>19 (17.6)</td><td>24 (22.2)</td><td>0.065</td></tr>
</table></td><td><u>VCI incidence</u>
108/27,818 (0.4%) amongst singleton pregnancies</td><td>Outcomes between VCI case and their matched controls<table>
<tr><th></th><th>VCI n=108</th><th>No VCI n=108</th><th>P value</th></tr>
<tr><td>Pre-eclampsia, n (%)</td><td>5 (4.6)</td><td>7 (6.5)</td><td>0.768</td></tr>
<tr><td>Growth retardation,[a] n (%)</td><td>6 (5.6)</td><td>5 (4.6)</td><td>1.000</td></tr>
<tr><td>Intrauterine fetal death, n (%)</td><td>7 (6.5)</td><td>0</td><td>0.014</td></tr>
<tr><td>Pre-term delivery, n (%)</td><td>35 (32.4)</td><td>8 (6.5)</td><td><0.001</td></tr>
<tr><td>CS</td><td>43 (39.8)</td><td>34 (31.5)</td><td>0.201</td></tr>
<tr><td>Apgar score 1 min, mean (SD)</td><td>7.6 (2.7)</td><td>8.7 (1.1)</td><td><0.001</td></tr>
<tr><td>Apgar score 5 min, mean (SD)</td><td>8.6 (2.9)</td><td>9.7 (1.1)</td><td><0.001</td></tr>
</table>[a] No detail as to how this parameter was defined are given.

Pregnancy induced hypertension, malformations, gestational age at delivery, early premature delivery, birth weight, pH and 10 min Apgar scores are also reported.</td></tr>
</table>

CONFIDENTIAL

Study reference	Study design	Population characteristics	Prevalence and incidence outcomes	Risk or incidence of perinatal outcomes associated with VCI
	Fetomaternal Medicine of the Medical University of Vienna	y, n (%) | | |		

Note: Values in *italics* were calculated by the reviewer.

Abbreviations: 95% CI, 95% confidence interval; BMI, body mass index; CS, caesarean section; DC, dichorionic; FHR, fetal heart rate; FTV, fetal thrombotic vasculopathy, IVF, *in vitro* fertilisation; IUFD, intra-uterine fetal demise; MCDA; monochorionic diamniotic; MCI, marginal cord insertion; MCMA, monochorionic monoamniotic; NA, not applicable; NR, not reported; OR, odds ratio; PC, paracentral; SD, standard deviation; SGA, small for gestational age, TTTS, twin to twin transfusion syndrome; VCI, velamentous cord insertion; VP, vasa praevia

CONFIDENTIAL

Criterion 4 – Screening tests for VCI

Six publications reported data on the effectiveness of screening in detection of VCI, each performed on a unique cohort of women. All relevant data has been extracted into Table 20 and used for the question-level synthesis in Section 2.3.2.1.

Table 20. Studies relevant to criterion 4 – Screening tests for VCI

Study reference	Study design	Population characteristics	Screening methods	Screening accuracy
**Di Salvo 1998**[58]<br><br>Relevant to Q8	Design<br>Prospective cohort study<br><br>Objective<br>To assess the ability of prenatal sonography to reveal abnormal insertions of the umbilical cord into the placenta.<br><br>Dates<br>1992 to 1995	Patient recruitment methodology<br>Prospective evaluation of the placental cord insertion site in 360 pregnant women scanned at the researchers' institution.<br><br>Sample size<br>Pathologic examination of the placenta was performed in 46 of the 360 women who had perinatal complications or multiple gestations. The 46 pregnancies included 38 singletons and eight twin pairs, for a **total of 54 placental cord insertions**.	Screening<br>Placental cord insertion site was identified with **grey-scale imaging** by visualising the entry of the umbilical vessels into the fetal surface of the placenta, noting contiguity of the cord sheath with the chorionic plate.<br><br>In some cases, **colour Doppler imaging was used, at the discretion of the sonologist**, to assist localisation.<br><br>The placental cord insertion was classified sonographically as normal if it was located greater than 1cm from the placental edge and as abnormal if it was located within 1cm from the edge.	The study as a whole was assessing all types of abnormal cord insertion, but screening test performance measures for VCI can be calculated.<br><br>Sensitivity<br>*Calculated value: 1/4 = 25%*<br><br>Specificity<br>*Calculated value: 50/50 = 100%*<br><br>PPV<br>*Calculated value: 1/1 = 100%*<br><br>NPV

<table>
<tr><th>Study reference</th><th>Study design</th><th>Population characteristics</th><th>Screening methods</th><th>Screening accuracy</th></tr>
<tr>
<td></td>
<td>Duration
36 months

Country
USA

Setting
Brigham and Women's Hospital, Harvard Medical School</td>
<td>Baseline characteristics
Gestational age at the time of the study sonogram ranged from 13 to 39 weeks (mean 27 weeks). The time elapsed between the date of the study sonogram and delivery ranged from 2 days to 21 weeks (mean 7.9 weeks).</td>
<td>All examinations were performed by one of six radiologists experienced in obstetric sonography using a 128-XP scanner (Acuson, Mountain View, CA) with a 3.5- to 5-MHz vector or curved transducer.

Reference Standard
Postnatal pathologic examination was performed and sonographic classification was compared with the final pathologic diagnosis.</td>
<td>Calculated value: 50/53 = 94.3%
<table>
<tr><th></th><th>VCI +</th><th>VCI -</th><th>Total</th></tr>
<tr><td>Screen +</td><td>1</td><td>0</td><td>1</td></tr>
<tr><td>Screen -</td><td>3*</td><td>50</td><td>53</td></tr>
<tr><td>Total</td><td>4</td><td>50</td><td>54</td></tr>
</table>
*Note: the 3 false negatives were originally diagnosed as MCI

When all types of abnormal cord insertion are taken into account, sonography had an overall sensitivity of 69% (11/16), a specificity of 100% (38/38), an accuracy of 91% (49/54), and a negative predictive value of 88% (5/43) for revealing an abnormal insertion site</td>
</tr>
<tr>
<td>Hasegawa 2006,[59] Hasegawa 2005[60]

(reporting on overlapping cohorts)

Relevant to Q8</td>
<td>Design
Retrospective cohort study

Objective
Hasegawa 2006: To evaluate the accuracy of sonographic identification of the site of umbilical cord insertion at 18 to 20 weeks of gestation, to compare the sensitivities for detection of a velamentous cord insertion (VCI) secondary to a cord insertion into the anterior, posterior or fundal wall, and to compare the intrapartum complications secondary to VCI into the upper, middle or lower third of</td>
<td>Hasegawa 2006

Patient recruitment methodology
Pregnant women who delivered between September 2002 and June 2004. Women who first visited the hospital after 20 weeks of gestation were excluded from the study.

Sample size
3,446 pregnant women

Baseline characteristics
<table>
<tr><th>Mean (SD)</th><th>VCI</th><th>MCI</th><th>Normal CI</th></tr>
<tr><td>Age (years)</td><td>31.0 (5.4)</td><td>30.0 (4.3)</td><td>31.5 (4.8)</td></tr>
<tr><td>GA at delivery (weeks)</td><td>38.0 (3.0)</td><td>38.9 (1.9)</td><td>38.3 (2.7)</td></tr>
</table></td>
<td>Hasegawa 2006

Screening
Abnormal cord insertion and the location of the cord insertion in the uterus was evaluated by ultrasound examination between 18 and 20 weeks of gestation.

The approximately 30-min ultrasound examination of the fetus, placenta and cord was part of a routine prenatal scan. The examiners were obstetricians and gynaecologists with 3 to 5 years' experience. They used Sonovista Colour FD (Mochida Inc., Tokyo) and Prosound SSD-5000 (Aloka Inc., Tokyo) equipment with a 3.5-MHz convex transducer transabdominally. The cord insertion site was imaged using grey-scale ultrasonography, and colour-flow imaging was applied in cases of suspected VCI

Reference standard
Postnatal examinations of the aberrant vessels were pathologically measured by obstetricians at delivery</td>
<td>Hasegawa 2006

Sensitivity
Reported as 25/40 (62.5%)

Specificity
Reported as 3,406/3,406 (100%)

PPV
Reported as 25/25 (100%)

NPV
Reported as 3,406/3,421 (99.6%)
<table>
<tr><th></th><th>VCI +</th><th>VCI -</th><th>Total</th></tr>
<tr><td>Screen +</td><td>25</td><td>0</td><td>25</td></tr>
<tr><td>Screen -</td><td>15</td><td>3,406</td><td>3,421</td></tr>
<tr><td>Total</td><td>40</td><td>3,421</td><td>3,446</td></tr>
</table>
Of the 15 false negative cases, two were diagnosed as MCI, nine were diagnosed as normal cord insertion, and four were diagnosed as unknown</td>
</tr>
</table>

<table>
<tr><th>Study reference</th><th>Study design</th><th>Population characteristics</th><th>Screening methods</th><th>Screening accuracy</th></tr>
<tr>
<td></td>
<td>the uterus

Hasegawa 2005: To examine intrapartum fetal heart rate (FHR) patterns in the presence of velamentous cord insertion (VCI)

<u>Dates</u>
Hasegawa 2006: September 2002 to June 2004

Hasegawa 2005: February 2002 to May 2003

<u>Country</u>
Japan

<u>Setting</u>
Showa University Hospital</td>
<td>Hasegawa 2005

<u>Patient recruitment methodology</u>
The placentas of 1,460 consecutive women with singleton pregnancies

<u>Sample size</u>
1,460 women with singleton pregnancies, of whom 29 had VCI

<u>Baseline characteristics</u>
Only reported for the VCI group:
• Mean (SD) age 31.1 (5.2) years
• Mean (SD) GA at birth 38.2 (3.2) weeks (range 37 to 41 weeks)</td>
<td>Hasegawa 2005

<u>Screening</u>
The attachment site of the placenta and location of the cord insertion in the placenta were determined by antenatal ultrasound screening, and antenatal diagnosis of VCI was made in some cases

<u>Reference standard</u>
Postnatal examinations of the placentas and umbilical cord were performed and ultrasonographic classification was compared with the final pathologic diagnosis.</td>
<td>Hasegawa 2005

<u>Sensitivity</u>
Reported as detection rate of 17/29 (58.6%)

<u>Specificity</u>
Calculated value: 1,429/1,431 (99.9%)

<u>PPV</u>
Reported as 17.19 (89.5%)

<u>NPV</u>
Calculated value: 1,429/1,441 (99.2%)

<table>
<tr><th></th><th>VCI +</th><th>VCI -</th><th>Total</th></tr>
<tr><td>Screen +</td><td>17</td><td>2</td><td>19</td></tr>
<tr><td>Screen -</td><td>12</td><td>1,429</td><td>1,441</td></tr>
<tr><td>Total</td><td>29</td><td>1,431</td><td>1,460</td></tr>
</table></td>
</tr>
<tr>
<td>Nomiyama 1998[38]

Relevant to Q8</td>
<td><u>Design</u>
Prospective study

<u>Objective</u>
To determine whether cord insertion can be consistently visualised and whether VCI and VP can be consistently identified with colour Doppler imaging during routine sonography in the mid-trimester

<u>Dates</u></td>
<td><u>Patient recruitment methodology</u>
Women undergoing routine ultrasonographic examination

<u>Sample size</u>
The study group included 555 singleton and 16 sets of twin pregnancies (three diamniotic monochorionic and 13 diamniotic dichorionic). Cord insertion was visualised in 586/587 (99.8%) fetuses</td>
<td><u>Screening</u>
A total of 587 fetuses at 18 to 20 weeks' gestation were scanned using transabdominal colour Doppler imaging for placental cord insertion by one sonographer during routine ultrasonographic examination. The sonographer was instructed to take additional time to image the placental cord insertion and indicate whether it was normal, velamentous or not visualised. The sonographer looked for the cord insertion site for 2 minutes before stating that it was not seen

The equipment used included Toshiba SSA-160A and SSA-260A (Tokyo, Japan)

The mothers of fetuses with VCI were requested to</td>
<td><u>Sensitivity</u>
Reported as 5/5 (100%)

<u>Specificity</u>
Reported as 580/581 (99.8%)

<u>PPV</u>
Reported as 5/6 (83%)

<u>NPV</u>
Reported as 580/580 (100%)

<table>
<tr><th></th><th>VCI +</th><th>VCI -</th><th>Total</th></tr>
<tr><td>Screen +</td><td>5</td><td>1</td><td>6</td></tr>
</table></td>
</tr>
</table>

CONFIDENTIAL

<table>
<tr><th>Study reference</th><th>Study design</th><th>Population characteristics</th><th>Screening methods</th><th>Screening accuracy</th></tr>
<tr>
<td></td>
<td>January 1993 to December 1996

<u>Country</u>
Japan

<u>Setting</u>
Saga Prefectual Hospital Kouseikan</td>
<td></td>
<td>return for repeat sonograms at 30 and 36 weeks of gestation to evaluate growth and to determine whether the cord insertion had changed. At this stage, if the cord insertion site could not be visualised by the transabdominal approach, transvaginal colour Doppler imaging was used

<u>Reference standard</u>
Outcome information regarding the location of placental cord insertion was obtained from medical records</td>
<td><table>
<tr><td>Screen -</td><td>0</td><td>580</td><td>580</td></tr>
<tr><td>Total</td><td>5</td><td>581</td><td>586</td></tr>
</table></td>
</tr>
<tr>
<td>Pretorius 1996[61]

Relevant to Q8</td>
<td><u>Design</u>
Prospective study

<u>Objective</u>
To relate sonographic identification of placental cord insertion with placental pathology and to evaluate the possibility that a marginal cord insertion may evolve into a velamentous cord insertion, and to determine the frequency and factors affecting sonographic visualisation of cord insertion

<u>Dates</u>
October 1992 to December 1993

<u>Country</u>
USA

<u>Setting</u>
Sharp Perinatal Centre,</td>
<td><u>Patient recruitment methodology</u>
Fetuses were scanned prospectively during routine perinatal ultrasonographic examination

<u>Sample size</u>
917 fetuses at greater than 15 (and up to 40) weeks of development were scanned

Placental pathologic reports were available for 139 pregnancies: 128 singleton, 10 twin, and one triplet pregnancies

Outcomes relate to 128 singleton pregnancies only

<u>Baseline characteristics</u>
NR</td>
<td><u>Screening</u>
917 fetuses at greater than 15 weeks of development were scanned (1,091 scans) prospectively for placental cord insertion by three sonographers during routine perinatal ultrasonographic examination. Sonographers were instructed to take additional time and to image the placental cord insertion and indicate cord insertion as normal, marginal, velamentous or not visualised

The mothers of fetuses with MCI and VCI were requested to return for repeat sonograms near 32 weeks' GA to evaluate growth and determine whether the cord insertion had changed. The last sonogram obtained during gestation in which the cord insertion was identified was used to compare to outcome data

Equipment used included Ultramark 9 and High Density Imaging (Advanced Technology Laboratories, Bothell, WA), both with colour Doppler capabilities

<u>Reference standard</u>
Available placental samples were examined by a single pathologist, who was blinded to ultrasonographic results, for cord insertion location (measuring the shortest distance between the cord insertion and the placental edge)</td>
<td>The study as a whole was assessing normal, marginal and velamentous cord insertions, but screening test performance measures for VCI can be calculated from Table 2 in the publication

<u>Sensitivity</u>
Calculated value: 2/6 (33.3%)

<u>Specificity</u>
Calculated value: 122/122 (100%)

<u>PPV</u>
Calculated value: 2/2 (100%)

<u>NPV</u>
Calculated value: 122/126 (96.8%)

<table>
<tr><th></th><th>VCI +</th><th>VCI -</th><th>Total</th></tr>
<tr><td>Screen +</td><td>2</td><td>0</td><td>2</td></tr>
<tr><td>Screen -</td><td>4*</td><td>122</td><td>126</td></tr>
<tr><td>Total</td><td>6</td><td>122</td><td>128</td></tr>
</table>
*Note: includes three where the screening result was 'marginal' and one where the screening result was 'not seen'

Note: When both marginal and velamentous cord insertion are taken into account, the sonographic identification for 128 singleton</td>
</tr>
</table>

CONFIDENTIAL

<table>
<thead>
<tr><th>Study reference</th><th>Study design</th><th>Population characteristics</th><th>Screening methods</th><th>Screening accuracy</th></tr>
</thead>
<tbody>
<tr>
<td></td>
<td>Mary Birch Hospital for Women</td>
<td></td>
<td></td>
<td>pregnancies has a sensitivity of 42%, specificity of 95%, PPV of 67%, and NPV of 12%</td>
</tr>
<tr>
<td>Sepulveda 2006[62]

Relevant to Q8</td>
<td><u>Design</u>
Prospective study

<u>Objective</u>
The purpose of this study was to determine the feasibility of prenatal sonography for detecting velamentous insertion of the umbilical cord in singleton pregnancies at the 11 to 14 week scan

<u>Dates</u>
NR, duration was one year

<u>Country</u>
Chile

<u>Setting</u>
Fetal Medical Centre, Department of Obstetrics and Gynaecology, Santiago</td>
<td><u>Patient recruitment methodology</u>
Consecutive singleton pregnancies over a 1-year period

<u>Sample size</u>
533 consecutive singleton pregnancies

<u>Baseline characteristics</u>
Screening was carried out at a median (range) GA of 12 (11 to 14) weeks</td>
<td><u>Screening</u>
Routine sonographic screening at 11 to 14 weeks

For the purpose of this study, all scans were performed by a fetal medicine specialist using high-resolution sonography equipment (Accuvix XQ [Medison Co, Ltd, Seoul, Korea] and Voluson 730 [GE healthcare, Milwaukee, WI]). The scan was initially performed transabdominally, but when an abnormal umbilical cord insertion was suspected, a transvaginal sonographic examination was offered to obtain a better view and confirm the diagnosis

Insertion site was confirmed with colour flow imaging if needed

The umbilical cord insertion site was again sought in the second trimester as part of the detailed sonographic examination of the fetal anatomy

<u>Reference standard</u>
Placental examination after delivery</td>
<td><u>Sensitivity</u>
Calculated value: 5/5 (100%)

<u>Specificity</u>
Calculated value: 528/528 (100%)

<u>PPV</u>
Calculated value: 5/5 (100%)

<u>NPV</u>
Calculated value: 528/528 (100%)

<table>
<tr><th></th><th>VCI +</th><th>VCI -</th><th>Total</th></tr>
<tr><td>Screen +</td><td>5</td><td>0</td><td>5</td></tr>
<tr><td>Screen -</td><td>0</td><td>528</td><td>528</td></tr>
<tr><td>Total</td><td>5</td><td>528</td><td>533</td></tr>
</table>
Note: although these values are from the first screening, there were no differences on the second scans</td>
</tr>
<tr>
<td>Sepulveda 2003[63]

Relevant to Q8</td>
<td><u>Design</u>
Prospective, cross-sectional study

<u>Objective</u>
To determine the feasibility of identifying VCI during routine obstetric ultrasound</td>
<td><u>Patient recruitment methodology</u>
Women attending the centre for routine obstetric ultrasound were prospectively enrolled into this study. Only live singleton pregnancies of at least 16 weeks' gestation, with adequate amniotic fluid volume and scanned by one of the authors were included</td>
<td><u>Screening</u>
Colour Doppler ultrasound was routinely performed to identify the placental cord insertion site. The role of three-dimensional (3D) ultrasound in evaluating the placental cord insertion site was also studied in a subset of 50 pregnancies from this population

All examinations were performed using a Voluson 730 real-time 4D ultrasound system (General Electric Medical Systems, Milwaukee, WI, USA)</td>
<td><u>Sensitivity</u>
Calculated value: 7/7 (100%)

<u>Specificity</u>
Calculated value: 824/825 (99.8%)

<u>PPV</u>
Calculated value: 7/8 (87.5%)</td>
</tr>
</tbody>
</table>

CONFIDENTIAL

<table>
<tr><th>Study reference</th><th>Study design</th><th>Population characteristics</th><th>Screening methods</th><th>Screening accuracy</th></tr>
<tr>
<td></td>
<td><u>Dates</u>
November 2001 to July 2002

<u>Country</u>
Chile

<u>Setting</u>
Fetal Medical Centre, Department of Obstetrics and Gynaecology, Santiago</td>
<td><u>Sample size</u>
832 women

<u>Baseline characteristics</u>
Median GA at the time of evaluation was 23 (range 16 to 40) weeks
• 235 (29%) had GA 16 to 20 weeks
• 251 (30%) had GA 21 to 24 weeks
• 142 (17%) had GA 25 to 30 weeks
• 139 (17%) had GA 31 to 34 weeks
• 65 (8%) had GA 35 to 40 weeks</td>
<td>equipped with colour Doppler, power Doppler and three-dimensional (3D) imaging capabilities

If insufficient transabdominal image quality was obtained, transvaginal ultrasound was performed to search the lower uterine segment with colour flow imaging and rule out vasa praevia

<u>Reference standard</u>
The placenta was routinely examined after delivery by the attending obstetrician or midwife caring for the woman during labour. The diagnosis of VCI was made macroscopically if the cord inserted into the membranes away from the placental edge</td>
<td><u>NPV</u>
Calculated value: 824/824 (100%)
<table><tr><th></th><th>VCI +</th><th>VCI -</th><th>Total</th></tr><tr><td>Screen +</td><td>7</td><td>1</td><td>8</td></tr><tr><td>Screen -</td><td>0</td><td>824</td><td>824</td></tr><tr><td>Total</td><td>7</td><td>825</td><td>832</td></tr></table>Confident identification of the placental cord insertion site was achieved in 825/832 (99%) cases

The one false positive case was confirmed as marginal insertion at delivery</td>
</tr>
</table>

Note: Values in *italics* were calculated by the reviewer.
Abbreviations: 95% CI, 95% confidence interval; FHR, fetal heart rate; GA, gestational age; MCI, marginal cord insertion; NPV, negative predictive value; PPV, positive predictive value; VCI, velamentous cord insertion

Criteria 9 and 10 – Management pathways for VCI

No studies were identified that analysed outcomes relating to different management pathways for VCI.

CONFIDENTIAL

Appraisal for quality and risk of bias

Quality assessments of included studies are reported below. Where one publication reported data relevant to multiple questions (for example, studies that reported epidemiology data as well as performance of screening methods), a separate quality assessment has been performed for each question.

Studies relating to VP

Criterion 1 – Epidemiology of VP

Table 21. Quality assessment of epidemiological and prognostic studies for VP

Study reference	Baulies 2007[16]	Baumfeld 2016[35]	Evron 2015[34]	Rosenberg 2011[32]	Bronsteen 2013[17]	Lee 2000[37]	Donegan 2014[15]	Francois 2003[33]	Hasegawa 2015[18]	Hasegawa 2010[24]	Hasegawa 2006[19]	Heller 2014[25]	Huerta-Enochian 2001[71]	Kanda 2011[20]	Kapoor 2014[31]	Oyelese 2014[29]	Rebarber 2014[6]	Robinson 2012[22]	Schachter 2002[23]	Smorgick 2010[14]	Suzuki 2015[21]	Suzuki 2010[28]	Suzuki 2008[30]	Swank 2016[26]
PATIENT SELECTION																								
Was the sample representative of the target population?	U	Y	Y	N	Y	Y	N	Y	NA	Y	Y	N	N	Y	N	Y	U	N	Y	Y	N	Y	N	Y
Were study participants recruited in an appropriate way?	Y	Y	Y	Y	Y	Y	Y	Y	U	Y	Y	Y	Y	Y	Y	Y	Y	Y	Y	Y	Y	Y	U	Y
Was the sample size adequate?	Y	Y	Y	U	Y	Y	Y	Y	NA	Y	N	N	N	Y	Y	Y	Y	N	Y	Y	Y	Y	Y	Y
Were the study subjects and the setting described in detail?	N	N	Y	Y	N	N	N	U	N	N	Y	N	N	N	N	Y	U	Y	N	N	Y	Y	Y	Y
STUDY ANALYSIS																								
Was the data analysis conducted with sufficient coverage of the identified sample?	Y	Y	Y	Y	Y	Y	Y	Y	Y	Y	Y	U	Y	Y	U	Y	Y	N	Y	Y	Y	Y	U	Y
Were objective, standard criteria used for the measurement of the condition?	Y	U	U	U	U	Y	U	U	U	Y	U	U	U	U	Y	Y	Y	U	U	Y	U	U	U	Y
Was the condition measured reliably?	Y	U	Y	Y	Y	N	U	U	U	Y	Y	U	U	U	U	Y	Y	Y	U	U	U	U	U	Y
Was there appropriate statistical analysis?	Y	Y	Y	NA	Y	NA	NA	Y	NA	U	Y	NA	NA	NA	Y	Y	NA	NA	Y	Y	Y	Y	Y	Y
Are all important confounding factors/ subgroups/differences identified and	Y	N	Y	N	N	N	NA	Y	N	N	N	N	N	N	N	NA	N	NA	N	Y	N	N	N	Y

Study reference	Baulies 2007[16]	Baumfeld 2016[35]	Evron 2015[34]	Rosenberg 2011[32]	Bronsteen 2013[17]	Lee 2000[37]	Donegan 2014[15]	Francois 2003[33]	Hasegawa 2015[18]	Hasegawa 2010[24]	Hasegawa 2006[19]	Heller 2014[25]	Huerta-Enochian 2001[71]	Kanda 2011[20]	Kapoor 2014[31]	Oyelese 2014[29]	Rebarber 2014[6]	Robinson 2012[22]	Schachter 2002[23]	Smorgick 2010[14]	Suzuki 2015[21]	Suzuki 2010[28]	Suzuki 2008[30]	Swank 2016[26]
accounted for?																								
Were subpopulations identified using objective criteria?	U	NA	NA	Y	NA	NA	NA	U	Y	U	Y	NA	NA	NA	NA	Y	NA	NA	Y	U	U	Y	Y	Y
PROGNOSTIC STUDIES QA																								
Was the defined representative sample of patients assembled at a common (usually early) point in the course of their disease)?	N	Y	Y	Y	N	Y	U	Y	U	U	Y	Y	U	N	Y	U	N	Y	U	U	Y	Y	Y	N
Was patient follow-up sufficiently long and complete?	Y	Y	Y	Y	Y	Y	Y	Y	Y	Y	Y	Y	Y	Y	Y	Y	Y	Y	Y	Y	Y	Y	Y	Y
Were outcome criteria either objective or applied in a 'blind' fashion?	U	U	NA	NA	Y	U	NA	U	U	U	U	NA	NA	U	NA	Y	U	NA	Y	U	NA	NA	NA	Y
If subgroups with different prognoses are identified, did adjustment for important prognostic factors take place?	Y	N	NA	NA	N	N	NA	N	N	Y	N	NA	NA	N	NA	Y	NA	NA	N	N	NA	NA	NA	N

Abbreviations: N, no; NA, not applicable; U, unclear; Y, yes

Criterion 4 – Screening tests for VP

Table 22. Quality assessment of diagnostic test studies for VP

Study reference	Bronsteen 2013[17]	Catanzarite 2001[2]	Kanda 2011[20]	Nomiyama 1998[38]
PATIENT SELECTION				
Was a consecutive or random sample of patients enrolled?	Y	Y	Y	Y
Was a case-control design avoided?	Y	Y	Y	Y
Did the study avoid inappropriate exclusions?	Y	Y	Y	Y
**Could the selection of patients have introduced bias? (Risk: Low, Unclear, High)**	High	Low	High	Low
**Is there concern that the included patients do not match the review question? (Concern: Low, Unclear, High)**	Low	Low	Low	Low
INDEX TESTS				

Study reference	Bronsteen 2013[17]	Catanzarite 2001[2]	Kanda 2011[20]	Nomiyama 1998[38]
Were the index test results interpreted without knowledge of the reference standard?	Y	Y	Y	Y
If a threshold was used, was it pre-specified?	Y	Y	U	Y
**Could the conduct or interpretation of the index test have introduced bias? (Risk: Low, Unclear, High)**	High	High	High	Low
**Is there concern that the index test, its conduct, or interpretation differ from the review question? (Concern: Low, Unclear, High)**	High	High	High	High
REFERENCE STANDARD				
Is the reference standard likely to correctly classify the test condition?	U	U	U	Y
Were the reference standard results interpreted without knowledge of the results of the index test?	N	N	N	N
**Could the reference standard, its conduct, or its interpretation have introduced bias? (Risk: Low, Unclear, High)**	High	High	High	High
**Is there concern that the target condition as defined by the reference standard does not match the review question? (Concern: Low, Unclear, High)**	Low	Low	Low	Low
PATIENT FLOW				
Was there an appropriate interval between the index test(s) and the reference standard?	N	N	N	N
Did all patients receive a reference standard?	N	U	U	N
Did patients receive the same reference standard?	Y	U	U	N
Were all patients included in the analysis?	Y	Y	N	Y
**Could the patient flow have introduced bias? (Risk: Low, Unclear, High)**	High	High	High	High

Criteria 9 and 10 – Management pathways for VP

Table 23. Quality assessment of cohort studies for VP

Study reference	Hasegawa 2015[18]	Oyelese 2004[29]
Are the results of the study valid?		
Did the study address a clearly focused issue?	Y	Y
Was the cohort recruited in an acceptable way?	Y	N
Was the exposure accurately measured to minimise bias?	**Y**	**Y**
Was the outcome accurately measured to minimise bias?	**Y**	**Y**
Have the authors identified all important confounding factors?	N	N
Have they taken account of the confounding factors in the design and/or analysis?	N	N
Was the follow-up of subjects complete enough?	**Y**	**Y**
Was the follow-up of subjects long enough?	**Y**	**Y**

Note: The CASP Cohort Study Checklist also includes domains for “What are the results” and “Will the results help locally” but these have not been formally assessed using the checklist

Studies relating to VCI

Criterion 1 – Epidemiology of VCI

Table 24. Quality assessment of epidemiological and prognostic studies for VCI

Study reference	Baumfeld 2016[35]	Chu 2013[56]	Costa-Castro 2013[45], Lopriore 2007[50]	Lopriore 2012[49]	Costa-Castro 2016[53]	De Paepe 2010a[51] De Paepe 2010b[52]	De Paepe 2011[4]	Ebbing 2015[54], Ebbing 2013[1]	Hack 2008[46]	Hack 2009[47]	Hasegawa 2009a[41], Hasegawa 2009b[55]	Hasegawa 2011[40]	Kent 2011[48]	Lepais 2014[72]	McNamara 2014[42]	Pinar 2014[57]	Raisanen 2012[43]	Suzuki 2015[21]	Walker 2012[73]	Yerlikaya 2016[44]
PATIENT SELECTION																				
Was the sample representative of the target population?	N	N	N	N	N	N	N	Y	N	N	N	Y	N	N	N	Y	N	N	N	N
Were study participants recruited in an appropriate way?	Y	U	Y	Y	Y	Y	Y	Y	Y	Y	Y	Y	Y	Y	Y	Y	Y	Y	Y	Y
Was the sample size adequate?	NA	NA	Y	Y	Y	Y	Y	Y	Y	Y	N	Y	Y	N	Y	Y	Y	Y	Y	Y
Were the study subjects and the setting described in detail?	N	N	N	N	N	N	N	Y	N	N	N	N	N	N	N	N	Y	Y	N	Y
STUDY ANALYSIS																				
Was the data analysis conducted with sufficient coverage of the identified sample?	Y	Y	Y	Y	Y	Y	N	Y	Y	N	N	Y	Y	Y	Y	N	Y	Y	Y	Y
Were objective, standard criteria used for the measurement of the condition?	U	Y	U	Y	Y	Y	Y	U	Y	Y	N	Y	Y	U	U	U	Y	U	U	N
Was the condition measured reliably?	U	Y	U	Y	Y	Y	Y	Y	Y	Y	Y	Y	Y	N	U	Y	Y	N	Y	U
Was there appropriate statistical analysis?	Y	Y	Y	Y	Y	Y	Y	Y	Y	Y	Y	Y	Y	Y	Y	U	Y	Y	Y	Y
Are all important confounding factors/ subgroups/differences identified and accounted for?	N	N	N	N	N	N	N	N	N	N	N	N	N	N	N	N	Y	Y	N	N
Were subpopulations identified using objective criteria?	U	NA	U	NA	NA	NA	NA	Y	NA	NA	NA	NA	NA	NA	NA	NA	Y	U	NA	NA
PROGNOSTIC STUDIES QA																				

Study reference	Baumfeld 2016[35]	Chu 2013[56]	Costa-Castro 2013[45], Lopriore 2007[50]	Lopriore 2012[49]	Costa-Castro 2016[53]	De Paepe 2010a[51] De Paepe 2010b[52]	De Paepe 2011[4]	Ebbing 2015[54], Ebbing 2013[1]	Hack 2008[46]	Hack 2009[47]	Hasegawa 2009a[41], Hasegawa 2009b[55]	Hasegawa 2011[40]	Kent 2011[48]	Lepais 2014[72]	McNamara 2014[42]	Pinar 2014[57]	Raisanen 2012[43]	Suzuki 2015[21]	Walker 2012[73]	Yerlikaya 2016[44]
Was the defined representative sample of patients assembled at a common (usually early) point in the course of their disease)?	Y	Y	Y	Y	Y	Y	Y	Y	Y	Y	Y	Y	Y	Y	Y	Y	Y	Y	Y	N
Was patient follow-up sufficiently long and complete?	Y	Y	Y	Y	Y	NA	NA	Y	Y	NA	Y	Y	Y	Y	Y	Y	Y	Y	Y	Y
Were outcome criteria either objective or applied in a 'blind' fashion?	U	Y	Y	NA	Y	U	Y	U	Y	Y	U	NA	Y	U	NA	NA	U	U	NA	U
If subgroups with different prognoses are identified, did adjustment for important prognostic factors take place?	N	N	Y	NA	Y	NA	NA	Y	U	N	Y	NA	N	Y	NA	NA	Y	Y	NA	N

Criterion 4 – Screening tests for VCI

Table 25. Quality assessment of diagnostic test studies for VCI

**Study reference**	**Di Salvo 1998[58]**	**Hasegawa 2006[59], Hasegawa 2005[60]**	**Nomiyama 1998[38]**	**Pretorius 1996[61]**	**Sepulveda 2006[62]**	**Sepulveda 2003[63]**
PATIENT SELECTION						
Was a consecutive or random sample of patients enrolled?	U	Y	Y	Y	Y	Y
Was a case-control design avoided?	Y	Y	Y	Y	Y	Y
Did the study avoid inappropriate exclusions?	Y	Y	Y	Y	Y	N
**Could the selection of patients have introduced bias? (Risk: Low, Unclear, High)**	**Unclear**	**High**	**Low**	**Low**	**Low**	**High**

Study reference	Di Salvo 1998[58]	Hasegawa 2006[59], Hasegawa 2005[60]	Nomiyama 1998[38]	Pretorius 1996[61]	Sepulveda 2006[62]	Sepulveda 2003[63]
**Is there concern that the included patients do not match the review question? (Concern: Low, Unclear, High)**	**Low**	**Low**	**Low**	**Low**	**Low**	**Low**
INDEX TESTS						
Were the index test results interpreted without knowledge of the reference standard?	Y	Y	Y	Y	Y	Y
If a threshold was used, was it pre-specified?	Y	Y	Y	Y	Y	Y
**Could the conduct or interpretation of the index test have introduced bias? (Risk: Low, Unclear, High)**	**High**	**High**	**Low**	**High**	**High**	**High**
**Is there concern that the index test, its conduct, or interpretation differ from the review question? (Concern: Low, Unclear, High)**	**Low**	**Low**	**Low**	**Low**	**High**	**High**
REFERENCE STANDARD						
Is the reference standard likely to correctly classify the test condition?	Y	Y	Y	Y	Y	Y
Were the reference standard results interpreted without knowledge of the results of the index test?	N	U	N	Y	U	U
**Could the reference standard, its conduct, or its interpretation have introduced bias? (Risk: Low, Unclear, High)**	**High**	**Unclear**	**High**	**Low**	**Unclear**	**Unclear**
**Is there concern that the target condition as defined by the reference standard does not match the review question? (Concern: Low, Unclear, High)**	**Low**	**Low**	**Low**	**Low**	**Low**	**Low**
PATIENT FLOW						
Was there an appropriate interval between the index test(s) and the reference standard?	N	N	N	N	N	N
Did all patients receive a reference standard?	N	Y	Y	N	Y	Y
Did patients receive the same reference standard?	Y	Y	Y	Y	Y	Y
Were all patients included in the analysis?	N	Y	Y	N	Y	Y
**Could the patient flow have introduced bias? (Risk: Low, Unclear, High)**	**High**	**High**	**High**	**High**	**High**	**High**

Criteria 9 and 10 – Management pathways for VCI

No relevant studies were identified, so no quality assessment was performed.

Appendix 5 – UK NSC reporting checklist for evidence summaries

All items on the UK NSC Reporting Checklist for Evidence Summaries have been addressed in this report. A summary of the checklist, along with the page or pages where each item can be found in this report, is presented in Table 26.

Table 26. UK NSC reporting checklist for evidence summaries

	Section	Item	Page no.
1.	TITLE AND SUMMARIES		
1.1	Title sheet	Identify the review as a UK NSC evidence summary.	Title page
1.2	Plain English summary	Plain English description of the executive summary.	1
1.3	Executive summary	Structured overview of the whole report. To include: the purpose/aim of the review; background; previous recommendations; findings and gaps in the evidence; recommendations on the screening that can or cannot be made on the basis of the review.	1
2.	INTRODUCTION AND APPROACH		
2.1	Background and objectives	Background – Current policy context and rationale for the current review – for example, reference to details of previous reviews, basis for current recommendation, recommendations made, gaps identified, drivers for new reviews<br>Objectives – What are the questions the current evidence summary intends to answer? – statement of the key questions for the current evidence summary, criteria they address, and number of studies included per question, description of the overall results of the literature search.<br>Method – briefly outline the rapid review methods used.	8
2.2	Eligibility for inclusion in the review	State all criteria for inclusion and exclusion of studies to the review clearly (PICO, dates, language, study type, publication type, publication status etc.) To be decided *a priori*.	47
2.3	Appraisal for quality/risk of bias tool	Details of tool/checklist used to assess quality, for example QUADAS 2, CASP, SIGN, AMSTAR.	133
3.	SEARCH STRATEGY AND STUDY SELECTION (FOR EACH KEY QUESTION)		
3.1	Databases/ sources searched	Give details of all databases searched (including platform/interface and coverage dates) and date of final search.	46
3.2	Search strategy and results	Present the full search strategy for at least one database (usually a version of Medline), including limits and search filters if used.<br>Provide details of the total number of (results from each database searched), number of duplicates removed, and the final number of unique records to consider for inclusion.	46
3.3	Study selection	State the process for selecting studies – inclusion and exclusion criteria, number of studies screened by title/abstract and full text, number of reviewers, any cross checking carried out.	47
4.	STUDY LEVEL REPORTING OF RESULTS (FOR EACH KEY QUESTION)		
4.1	Study level reporting, results and risk of bias assessment	For each study, produce a table that includes the full citation and a summary of the data relevant to the question (for example, study size, PICO, follow-up period, outcomes reported, statistical analyses etc.).<br>Provide a simple summary of key measures, effect estimates and confidence intervals for each study where available.<br>For each study, present the results of any assessment of quality/risk of bias.	Study level reporting: 59<br>Quality assessment: 134

CONFIDENTIAL

	Section	Item	Page no.
4.2	Additional analyses	Describe additional analyses (for example, sensitivity, specificity, PPV, etc.) carried out by the reviewer.	Study level analyses within data extraction tables: 59
5.	QUESTION LEVEL SYNTHESIS		
5.1	Description of the evidence	For each question, give numbers of studies screened, assessed for eligibility, and included in the review, with summary reasons for exclusion.	12
5.2	Combining and presenting the findings	Provide a balanced discussion of the body of evidence which avoids over reliance on one study or set of studies. Consideration of four components should inform the reviewer's judgement on whether the criterion is 'met', 'not met' or 'uncertain': quantity; quality; applicability and consistency.	12
5.3	Summary of findings	Provide a description of the evidence reviewed and included for each question, with reference to their eligibility for inclusion. Summarise the main findings including the quality/risk of bias issues for each question. Have the criteria addressed been 'met', 'not met' or 'uncertain'?	12
6.	REVIEW SUMMARY		
6.1	Conclusions and implications for policy	Do findings indicate whether screening should be recommended? Is further work warranted? Are there gaps in the evidence highlighted by the review?	42
6.2	Limitations	Discuss limitations of the available evidence and of the review methodology if relevant.	44

CONFIDENTIAL

Appendix 6 – Glossary of key terms

Key terms relating to epidemiology and screening are defined below, and screening test performance measures are summarised in Figure 3.

Accuracy – the overall proportion of individuals with a correct diagnosis, whether positive or negative, out of the total study population

False negative – individuals with a disease who incorrectly receive a negative screening result

False positive – individuals without a disease who incorrectly receive a positive screening result

Incidence – the number of instances of disease during a given period in a specified population

Negative predictive value (NPV) – the proportion of individuals that screen negative that do not really have the disease

Positive predictive value (PPV) – the proportion of those that screen positive that do really have the disease

Prevalence – the number of instances of disease in the population at a single point in time

Reference standard – a test that is known to give highly accurate results for identifying the disease, which can be compared to a new screening method

Sensitivity – the proportion of individuals with the disease who will correctly receive a positive screen result, that is, the ability of the test to correctly identify individuals with the disease

Specificity – the proportion of individuals without the disease who will correctly receive a negative screen result, that is, the ability of the test to correctly exclude individuals without the disease

True negative – individuals without a disease who correctly receive a negative screening result

True positive – individuals with a disease who correctly receive a positive screening result

Figure 3. Summary of screening test performance measures

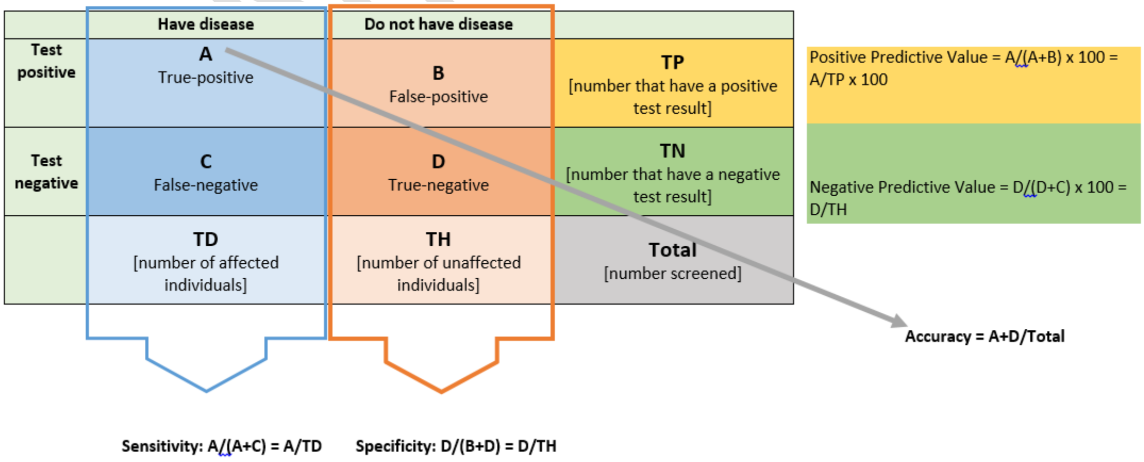

CONFIDENTIAL

5 References

1. Ebbing C, Kiserud T, Johnsen SL, et al. Prevalence, risk factors and outcomes of velamentous and marginal cord insertions: a population-based study of 634,741 pregnancies. PLoS ONE [Electronic Resource] 2013;8:e70380.
2. Catanzarite V, Maida C, Thomas W, et al. Prenatal sonographic diagnosis of vasa previa: Ultrasound findings and obstetric outcome in ten cases. Ultrasound in Obstetrics and Gynecology 2001;18:109-115.
3. National Institute for Health and Care Excellence (NICE). Antenatal care: Routine care for the healthy pregnant woman. [https://www.nice.org.uk/guidance/CG62]. 2008.
4. De Paepe ME, Shapiro S, Hanley LC, et al. Correlation between cord insertion type and superficial choriovasculature in diamniotic-monochorionic twin placentas. Placenta 2011;32:901-905.
5. Clark JFJ. Fetal heart monitoring in detecting velamentous insertion of the umbilical cord. Journal of the National Medical Association 1984;76:292-294.
6. Rebarber A, Dolin C, Fox NS, et al. Natural history of vasa previa across gestation using a screening protocol. Journal of ultrasound in medicine : official journal of the American Institute of Ultrasound in Medicine 2014;33:141-147.
7. Ruiter L, Kok N, Limpens J, et al. Incidence of and risk indicators for vasa praevia: a systematic review. BJOG: An International Journal of Obstetrics & Gynaecology 2016;123:1278-87.
8. Ioannou C, Wayne C. Diagnosis and management of vasa previa: a questionnaire survey. Ultrasound Obstet Gynecol 2010;35:205-9.
9. Derbala Y, Grochal F, Jeanty P. Vasa previa. J Prenat Med 2007;1:2-13.
10. Sinkey RG, Odibo AO, Dashe JS. #37: Diagnosis and management of vasa previa. American Journal of Obstetrics and Gynecology 2015;213:615-619.
11. UK NSC, Wood P. Vasa praevia and placenta praevia screening in pregnancy: UK National Screening Committee, 2013.
12. Human Fertilisation & Embryology Authority. Fertility Treatment in 2011. Trends and Figures. Available from: http://www.hfea.gov.uk/docs/HFEA_Fertility_Trends_and_Figures_2011_-_Annual_Register_Report.pdf. Last Accessed 5 August 2016.
13. Human Fertilisation & Embryology Authority. A long term analysis of the HFEA Register data (1991-2006). Available from: http://www.hfea.gov.uk/2586.html. Last accessed 5 August 2016.
14. Smorgick N, Tovbin Y, Ushakov F, et al. Is neonatal risk from vasa previa preventable? The 20-year experience from a single medical center. Journal of clinical ultrasound : JCU 2010;38:118-122.
15. Donegan K, King B, Bryan P. Safety of pertussis vaccination in pregnant women in UK: observational study. BMJ 2014;349:g4219.
16. Baulies S, Maiz N, Munoz A, et al. Prenatal ultrasound diagnosis of vasa praevia and analysis of risk factors. Prenatal Diagnosis 2007;27:595-599.
17. Bronsteen R, Whitten A, Balasubramanian M, et al. Vasa previa: clinical presentations, outcomes, and implications for management. Obstetrics and gynecology 2013;122:352-357.
18. Hasegawa J, Arakaki T, Ichizuka K, et al. Management of vasa previa during pregnancy. Journal of Perinatal Medicine 2015;43:783-784.

CONFIDENTIAL

19. Hasegawa J, Matsuoka R, Ichizuka K, et al. Cord insertion into the lower third of the uterus in the first trimester is associated with placental and umbilical cord abnormalities. Ultrasound in Obstetrics and Gynecology 2006;28:183-186.
20. Kanda E, Matsuda Y, Kamitomo M, et al. Prenatal diagnosis and management of vasa previa: a 6-year review. The journal of obstetrics and gynaecology research 2011;37:1391-1396.
21. Suzuki S, Kato M. Clinical Significance of Pregnancies Complicated by Velamentous Umbilical Cord Insertion Associated With Other Umbilical Cord/Placental Abnormalities. Journal of Clinical Medicine Research 2015;7:853-6.
22. Robinson AJ, Muller PR, Allan R, et al. Precise mid-trimester placenta localisation: Does it predict adverse outcomes? Australian and New Zealand Journal of Obstetrics and Gynaecology 2012;52:156-160.
23. Schachter M, Tovbin Y, Arieli S, et al. In vitro fertilization is a risk factor for vasa previa. Fertility and Sterility 2002;78:642-643.
24. Hasegawa J, Farina A, Nakamura M, et al. Analysis of the ultrasonographic findings predictive of vasa previa. Prenatal Diagnosis 2010;30:1121-1125.
25. Heller HT, Mullen KM, Gordon RW, et al. Outcomes of pregnancies with a low-lying placenta diagnosed on second-trimester sonography. Journal of ultrasound in medicine : official journal of the American Institute of Ultrasound in Medicine 2014;33:691-696.
26. Swank ML, Garite TJ, Maurel K, et al. Vasa previa: diagnosis and management. American Journal of Obstetrics & Gynecology 2016;2:2.
27. Eddleman KA, Lockwood CJ, Berkowitz GS, et al. Clinical significance and sonographic diagnosis of velamentous umbilical cord insertion. American Journal of Perinatology 1992;9:123-126.
28. Suzuki S, Igarashi M, Inde Y, et al. Abnormally shaped placentae in twin pregnancy. Archives of Gynecology and Obstetrics 2010;281:65-69.
29. Oyelese Y, Catanzarite V, Prefumo F, et al. Vasa previa: The impact of prenatal diagnosis on outcomes. Obstetrics and Gynecology 2004;103:937-942.
30. Suzuki S, Igarashi M. Clinical significance of pregnancies with succenturiate lobes of placenta. Archives of Gynecology and Obstetrics 2008;277:299-301.
31. Kapoor S, Thomas JT, Petersen SG, et al. Is the third trimester repeat ultrasound scan for placental localisation needed if the placenta is low lying but clear of the os at the mid-trimester morphology scan? Australian and New Zealand Journal of Obstetrics and Gynaecology 2014;54:428-432.
32. Rosenberg T, Pariente G, Sergienko R, et al. Critical analysis of risk factors and outcome of placenta previa. Archives of Gynecology and Obstetrics 2011;284:47-51.
33. Francois K, Mayer S, Harris C, et al. Association of Vasa Previa at Delivery with a History of Second-Trimester Placenta Previa. Journal of Reproductive Medicine for the Obstetrician and Gynecologist 2003;48:771-774.
34. Evron E, Sheiner E, Friger M, et al. Vanishing twin syndrome: Is it associated with adverse perinatal outcome? Fertility and Sterility 2015;103:1209-1214.
35. Baumfeld Y, Gutvirtz G, Shoham I, et al. Fetal heart rate patterns of pregnancies with vasa previa and velamentous cord insertion. Archives of Gynecology and Obstetrics 2016;293:361-367.
36. O'Connor M, Nair M, Kurinczuk JJ, et al. UKOSS Annual Report 2016. Oxford: National Perinatal

Epidemiology Unit. 2016.

CONFIDENTIAL

37. Lee W, Lee VL, Kirk JS, et al. Vasa previa: Prenatal diagnosis, natural evolution, and clinical outcome. Obstetrics and Gynecology 2000;95:572-576.
38. Nomiyama M, Toyota V, Kawano H. Antenatal diagnosis of velamentous umbilical cord insertion and vasa previa with color Doppler imaging. Ultrasound in Obstetrics and Gynecology 1998;12:426-429.
39. Ruiter L, Kok N, Limpens J, et al. Systematic review of accuracy of ultrasound in the diagnosis of vasa previa. Ultrasound in Obstetrics & Gynecology 2015;45:516-22.
40. Hasegawa J, Nakamura M, Sekizawa A, et al. Prediction of risk for vasa previa at 9-13 weeks' gestation. The journal of obstetrics and gynaecology research 2011;37:1346-1351.
41. Hasegawa J, Matsuoka R, Ichizuka K, et al. Atypical variable deceleration in the first stage of labor is a characteristic fetal heart-rate pattern for velamentous cord insertion and hypercoiled cord. Journal of Obstetrics and Gynaecology Research 2009a;35:35-39.
42. McNamara H, Hutcheon JA, Platt RW, et al. Risk factors for high and low placental weight. Paediatric and Perinatal Epidemiology 2014;28:97-105.
43. Raisanen S, Georgiadis L, Harju M, et al. Risk factors and adverse pregnancy outcomes among births affected by velamentous umbilical cord insertion: A retrospective population-based register study. European Journal of Obstetrics Gynecology and Reproductive Biology 2012;165:231-234.
44. Yerlikaya G, Pils S, Springer S, et al. Velamentous cord insertion as a risk factor for obstetric outcome: a retrospective case-control study. Archives of Gynecology and Obstetrics 2016;293:975-981.
45. Costa-Castro T, De Villiers S, Montenegro N, et al. Velamentous cord insertion in monochorionic twins with or without twin-twin transfusion syndrome: Does it matter? Placenta 2013;34:1053-1058.
46. Hack KEA, Nikkels PGJ, Koopman-Esseboom C, et al. Placental Characteristics of Monochorionic Diamniotic Twin Pregnancies in Relation to Perinatal Outcome. Placenta 2008;29:976-981.
47. Hack KEA, van Gemert MJC, Lopriore E, et al. Placental Characteristics of Monoamniotic Twin Pregnancies in Relation to Perinatal Outcome. Placenta 2009;30:62-65.
48. Kent EM, Breathnach FM, Gillan JE, et al. Placental cord insertion and birthweight discordance in twin pregnancies: Results of the national prospective ESPRiT Study. American Journal of Obstetrics and Gynecology 2011;205:376.e1-376.e7.
49. Lopriore E, Pasman SA, Klumper FJ, et al. Placental characteristics in growth-discordant monochorionic twins: A matched case-control study. Placenta 2012;33:171-174.
50. Lopriore E, Sueters M, Middeldorp JM, et al. Velamentous cord insertion and unequal placental territories in monochorionic twins with and without twin-to-twin-transfusion syndrome. American Journal of Obstetrics and Gynecology 2007;196:159.e1-159.e5.
51. De Paepe ME, Shapiro S, Greco D, et al. Placental markers of twin-to-twin transfusion syndrome in diamniotic-monochorionic twins: A morphometric analysis of deep artery-to-vein anastomoses. Placenta 2010a;31:269-276.
52. De Paepe ME, Shapiro S, Young L, et al. Placental characteristics of selective birth weight discordance in diamniotic-monochorionic twin gestations. Placenta 2010b;31:380-386.
53. Costa-Castro T, Zhao DP, Lipa M, et al. Velamentous cord insertion in dichorionic and monochorionic twin pregnancies - Does it make a difference? Placenta 2016;42:87-92.
54. Ebbing C, Kiserud T, Johnsen SL, et al. Third stage of labor risks in velamentous and marginal cord insertion: A population-based study. Acta Obstetricia et Gynecologica Scandinavica 2015;94:878-883.

CONFIDENTIAL

55. Hasegawa J, Matsuoka R, Ichizuka K, et al. Do fetal heart rate deceleration patterns during labor differ between various umbilical cord abnormalities? Journal of Perinatal Medicine 2009b;37:276-280.
56. Chu S, Mao Q, Shapiro S, et al. Correlation between cord insertion type and chorionic villus vascularization of the co-twin in diamniotic-monochorionic twin pregnancies. Early Human Development 2013;89:243-247.
57. Pinar H, Goldenberg RL, Koch MA, et al. Placental findings in singleton stillbirths. Obstetrics and gynecology 2014;123:325-336.
58. Di Salvo DN, Benson CB, Laing FC, et al. Sonographic evaluation of the placental cord insertion site. American Journal of Roentgenology 1998;170:1295-1298.
59. Hasegawa J, Matsuoka R, Ichizuka K, et al. Velamentous cord insertion into the lower third of the uterus is associated with intrapartum fetal heart rate abnormalities. Ultrasound in Obstetrics and Gynecology 2006;27:425-429.
60. Hasegawa J, Matsuoka R, Ichizuka K, et al. Velamentous cord insertion and atypical variable decelerations with no accelerations. International Journal of Gynecology and Obstetrics 2005;90:26-30.
61. Pretorius DH, Chau C, Poeltler DM, et al. Placental cord insertion visualization with prenatal ultrasonography. Journal of Ultrasound in Medicine 1996;15:585-593.
62. Sepulveda W. Velamentous insertion of the umbilical cord: A first-trimester sonographic screening study. Journal of Ultrasound in Medicine 2006;25:963-968.
63. Sepulveda W, Rojas I, Robert JA, et al. Prenatal detection of velamentous insertion of the umbilical cord: A prospective color Doppler ultrasound study. Ultrasound in Obstetrics and Gynecology 2003;21:564-569.
64. Munn Z, Moola S, Riitano D, et al. The development of a critical appraisal tool for use in systematic reviews addressing questions of prevalence. Int J Health Policy Manag 2014;3:123-8.
65. Centre for Evidence-Based Medicine (CEBM). Critical Appraisal of Prognostic Studies. Available from: http://www.cebm.net/critical-appraisal/. Last accessed 3 August 2016.
66. Whiting PF, Rutjes AW, Westwood ME, et al. QUADAS-2: a revised tool for the quality assessment of diagnostic accuracy studies. Ann Intern Med 2011;155:529-36.
67. Higgins JPT, Altman DG, Gøtzsche PC, et al. The Cochrane Collaboration's tool for assessing risk of bias in randomised trials. BMJ 2011;343.
68. Downs SH, Black N. The feasibility of creating a checklist for the assessment of the methodological quality both of randomised and non-randomised studies of health care interventions. Journal of Epidemiology and Community Health 1998;52:377-384.
69. Critical Appraisal Skills Programme (CASP). Cohort Study Checklist 31.05.13. Available from: http://www.casp-uk.net/#!casp-tools-checklists/c18f8. Last accessed 3 August 2016.
70. Critical Appraisal Skills Programme (CASP). Case Control Study Checklist 31.05.13. Available from: http://www.casp-uk.net/#!casp-tools-checklists/c18f8. Last accessed 3 August 2016.
71. Huerta-Enochian G, Katz V, Erfurth S. The association of abnormal alpha-fetoprotein and adverse pregnancy outcome: Does increased fetal surveillance affect pregnancy outcome? American Journal of Obstetrics and Gynecology 2001;184:1549-1555.
72. Lepais L, Gaillot-Durand L, Boutitie F, et al. Fetal thrombotic vasculopathy is associated with thromboembolic events and adverse perinatal outcome but not with neurologic complications: A retrospective cohort study of 54 cases with a 3-year follow-up of children. Placenta 2014;35:611-617.

CONFIDENTIAL

73. Walker MG, Fitzgerald B, Keating S, et al. Sex-specific basis of severe placental dysfunction leading to extreme preterm delivery. Placenta 2012;33:568-571.
74. Ananth CV, Oyelese Y, Yeo L, et al. Placental abruption in the United States, 1979 through 2001: Temporal trends and potential determinants. American Journal of Obstetrics and Gynecology 2005;192:191-198.
75. Bjoro Jr K. Vascular anomalies of the umbilical cord: I. Obstetric implications. Early Human Development 1983;8:119-127.
76. Bjoro Jr K. Vascular anomalies of the umbilical cord. II. Perinatal and pediatric implications. Early Human Development 1983;8:279-287.
77. De Laat MWM, Franx A, Bots ML, et al. Umbilical coiling index in normal and complicated pregnancies. Obstetrics and Gynecology 2006;107:1049-1055.
78. Delbaere I, Goetgeluk S, Derom C, et al. Umbilical cord anomalies are more frequent in twins after assisted reproduction. Human Reproduction 2007;22:2763-2767.
79. Englert Y, Imbert MC, Van Rosendael E. Morphological anomalies in the placentae of IVF pregnancies: Preliminary report of a multicentric study. Human Reproduction 1987;2:155-157.
80. Feldman DM, Borgida AF, Trymbulak WP, et al. Clinical implications of velamentous cord insertion in triplet gestations. American Journal of Obstetrics and Gynecology 2002;186:809-811.
81. Paavonen J, Jouttunpaa K, Kangasluoma P. Velamentous insertion of the umbilical cord and vasa previa. International Journal of Gynecology and Obstetrics 1984;22:207-211.
82. Nakajima Y, Masaoka N, Yamamoto T. Obstetrical risk factors for focal intestinal perforation in very low birth weight infants. Journal of Perinatal Medicine 2011;39:179-184.
83. Otsubo Y, Yoneyama Y, Suzuki S, et al. Sonographic evaluation of umbilical cord insertion with umbilical coiling index. Journal of Clinical Ultrasound 1999;27:341-344.
84. Fries MH, Goldstein RB, Kilpatrick SJ, et al. The role of velamentous cord insertion in the etiology of twin-twin transfusion syndrome. Obstetrics and Gynecology 1993;81:569-574.
85. Heinonen S, Ryynanen M, Kirkinen P, et al. Velamentous umbilical cord insertion may be suspected from maternal serum alpha-fetoprotein and hCG. British Journal of Obstetrics and Gynaecology 1996;103:209-213.
86. Heinonen S, Ryynanen M, Kirkinen P, et al. Perinatal diagnostic evaluation of velamentous umbilical cord insertion: Clinical, Doppler, and ultrasonic findings. Obstetrics and Gynecology 1996;87:112-117.
87. Lauslahti K, Ikonen S. Placenta as an indicator of fetal postnatal prognosis. Acta Obstetricia et Gynecologica Scandinavica 1979;58:163-167.
88. Nordenvall M, Sandstedt B, Ulmsten U. Relationship between placental shpae, cord insertion, lobes and gestational outcome. Acta Obstetricia et Gynecologica Scandinavica 1988;67:611-616.
89. Quek SP, Tan KL. Vasa Praevia. The Australian & New Zealand journal of obstetrics & gynaecology 1972;12:206-209.
90. Salafia CM, Yampolsky M, Misra DP, et al. Placental surface shape, function, and effects of maternal and fetal vascular pathology. Placenta 2010;31:958-962.
91. Yampolsky M, Salafia CM, Shlakhter O, et al. Centrality of the Umbilical Cord Insertion in a Human Placenta Influences the Placental Efficiency. Placenta 2009;30:1058-1064.
92. Sato Y, Benirschke K. Increased prevalence of fetal thrombi in monochorionic-twin placentas. Pediatrics 2006;117:e113-e117.

CONFIDENTIAL

93. Tang MY. Pathologic analysis of preterm delivery placentas. Chinese medical journal 1990;103:856-861.

CONFIDENTIAL
