# Supplementary material for: Velamentous cord insertion: results from a rapid review of incidence, risk factors, adverse outcomes and screening
Source: Syst Rev. 2020 Jun 23;9:147. doi: 10.1186/s13643-020-01355-0 (PMC7313176; doi:10.1186/s13643-020-01355-0)
Supplement: Supplementary file 3 — Additional file 3. Supplementary data [file 13643_2020_1355_MOESM3_ESM.docx]

SUPPLEMENTARY DATA

Supplementary Table 1 – Summary of electronic database searches

**A) Databases, search platforms, and dates of searches**

| Database | Platform | Searched on date | Updated on date |
| --- | --- | --- | --- |
| MEDLINE, MEDLINE In-Process, MEDLINE Daily, Epub Ahead of Print | Ovid SP | 5^th^ July 2016 | 11 October 2019 |
| Embase | Ovid SP | 5^th^ July 2016 | 11 October 2019 |
| The Cochrane Library, including:  Cochrane Database of Systematic Reviews (CDSR)  Cochrane Central Register of Controlled Trials (CENTRAL)  Database of Abstracts of Reviews of Effects (DARE) | Wiley Online | 5^th^ July 2016 | 11 October 2019 |

**B) Search strategy for MEDLINE, MEDLINE In-Process, MEDLINE Daily, Epub Ahead of Print and Embase (searched simultaneously via Ovid SP) for the original and update searches**

| Original searches | | |
| --- | --- | --- |
| # | Search terms | Results (05/07/2016) |
| 1 | Exp Vasa Previa/ | 308 |
| 2 | (vasa previa or vasa praevia).tw | 541 |
| 3 | ((velamentous or marginal) adj3 insertion).tw. | 560 |
| 4 | Or/1-3 | 1018 |
| 5 | Remove duplicates from 4 | 652 |
| **Update searches** | | |
| **#** | **Search terms** | **Results (11/10/2019)** |
| 1 | Exp Vasa Previa/ | 495 |
| 2 | (vasa previa or vasa praevia).tw | 702 |
| 3 | ((velamentous or marginal) adj3 insertion).tw. | 695 |
| 4 | Or/1-3 | 1291 |
| 5 | Limit 4 to yr=2016-current | 360 |
| 6 | Remove duplicates from 5 | 238 |

**C) Search strategy for the Cochrane Library Databases (Searched via the Wiley Online platform) for the original and update searches**

| **Original searches** | | |
| --- | --- | --- |
| **#** | **Search terms** | **Results (05/07/2016)** |
| #1 | [mh “vasa previa”] | 1 |
| #2 | “vasa previa” or “vasa praevia” | 7 |
| #3 | (velamentous or marginal) near/3 insertion | 5 |
| #4 | #1 or #2 or #3 | 12 |
| #5 | #4  In Cochrane Reviews (Reviews only), Other Reviews and Trials | 11 |
| **Update searches** | | |
| **#** | **Search terms** | **Results (11/10/2019)** |
| #1 | [mh “vasa previa”] | 0 |
| #2 | “vasa previa” or “vasa praevia” | 11 |
| #3 | (velamentous or marginal) near/3 insertion | 8 |
| #4 | #1 or #2 or #3 | 19 |
| #5 | #4 with Cochrane Library publication date Between Jul 2016 and Oct 2019, in Cochrane Reviews | 0 |
| #6 | #4 with Publication Year from 2016 to 2019, in Trials | 7 |

Supplementary Table 2 ─ Eligibility criteria for publications relating to epidemiology

| Domain | Inclusion criteria* | | Exclusion criteria |
| --- | --- | --- | --- |
| **Population** | Pregnant women with a diagnosis of VP or VCI | | Studies that did not include pregnant women with a diagnosis of VP or VCI, or that considered this population cohort but did not present outcomes for them separately to outcomes for irrelevant cohorts. |
| **Intervention** | Any or none | | - |
| **Comparator** | Any or none | | - |
| **Outcomes** | VP  • Prevalence or incidence  • Percentage of cases identified in the second trimester that resolve by late pregnancy  • Risk of adverse perinatal outcomes, including but not restricted to:  • Abnormal intrapartum fetal heart rate patterns  • Admission to neonatal intensive care unit  • Fetal growth restriction  • Low Apgar scores at 1 and 5 minutes  • Low birthweight  • Neonatal and fetal deaths  • Placental abruption  • Pre-term delivery (including emergency CS) | VCI  • Prevalence or incidence  • Risk of adverse perinatal outcomes, including but not restricted to:  • Admission to neonatal intensive care unit  • Fetal growth restriction  • Low Apgar scores at 1 and 5 minutes  • Low birthweight  • Neonatal and fetal deaths  • Placental abruption  • Pre-eclampsia  • Pre-term delivery (including emergency CS) | Outcomes not deemed of relevance to this topic, although a broad approach was taken with regard to “relevance”. |
| **Study design and publication type** | Peer-reviewed evidence derived from the following types of study:  • Systematic reviews and meta-analyses  • Observational studies  • Cross-sectional studies  Case studies were tagged during abstract review but were not included for full-text review in the first instance. It was planned that case studies would be re-visited at a later stage of the review if any evidence gaps were identified for which they may have provided additional relevant evidence | | Other study designs/publication types.  Alternatively, RCTs or interventional non-RCTs that were unlikely to report relevant outcomes.  Conference abstracts or other publication types that have not been peer-reviewed. |
| **Language** | English language | | Non-English language |
| **Date** | No date limit pre-specified, but only studies completed in or after 2000 (for VP) or 2006 (for VCI) were included in the evidence synthesis | | No date limit pre-specified, but studies completed before 2000 (for VP) or 2006 (for VCI) were excluded from the evidence synthesis |

*Only the VCI part of the population and outcome eligibility criteria were applied during the update of the review. CS: cesaerian section; RCT: randomised controlled trial; VCI: velamentous cord insertion; VP: vasa praevia.

Supplementary Table 3 ─ Eligibility criteria for publications relating to performance of screening methods

| Domain | Inclusion criteria* | Exclusion criteria |
| --- | --- | --- |
| **Population** | Pregnant women at risk of VP or VCI | Studies that did not include pregnant women with a diagnosis of VP or VCI, or that considered this population cohort but did not present outcomes for them separately to outcomes for irrelevant cohorts |
| **Intervention** | • TAS ± colour Doppler  • Transvaginal ultrasound ± colour Doppler  • Other detection methods | - |
| **Comparator** | As listed in “Interventions” | - |
| **Outcomes** | Outcomes relating to clinical performance measures:  • Sensitivity  • Specificity  • False positive rate  • False negative rate  • Positive predictive value  • Negative predictive value | Outcomes not relating to clinical performance measures for VP or VCI detection methods. |
| **Study design and publication type** | Peer-reviewed evidence derived from the following types of study:  • Systematic reviews and meta-analyses  • RCTs  • Interventional non-RCTs  • Prospective cohort studies  • Retrospective cohort studies  • Case-control studies  • Cross-sectional studies  Case studies were tagged during abstract review but were not included for full-text review in the first instance. It was planned that case studies would be re-visited at a later stage of the review if any evidence gaps were identified for which they may have provided additional relevant evidence | Other study designs/publication types.  Conference abstracts or other publication types that have not been peer-reviewed. |
| **Language** | English language | Non-English language |
| **Date** | No date limit | - |

*Only the VCI part of the population eligibility criteria were applied during the update of the review. RCT: randomised controlled trial; TAS: transabdominal sonography; VCI: velamentous cord insertion; VP: vasa praevia.

Supplementary Table 4 ─ Eligibility criteria for publications relating to the effectiveness of management pathways

| Domain | Inclusion criteria* | Exclusion criteria |
| --- | --- | --- |
| **Population** | Pregnant women with screen-detected VP, or VCI in the absence of VP, who have been the subject of a management strategy. | Studies that did not include pregnant women with a diagnosis of VP, or VCI in the absence of VP, who have been the subject of a management strategy. Alternatively, studies that considered one or both of these population cohorts but did not present outcomes for them separately to outcomes for irrelevant cohorts |
| **Intervention** | Any | - |
| **Comparator** | Any or none | - |
| **Outcomes** | Clinical outcomes relating to the effectiveness of management pathways for VP or VCI in the absence of VP, including but not limited to:  VP  • Abnormal intrapartum fetal heart rate patterns  • Admission to neonatal intensive care unit  • Fetal growth restriction  • Low Apgar scores at 1 and 5 minutes  • Low birthweight  • Neonatal and fetal deaths  • Placental abruption  • Pre-term delivery (including emergency CS)  VCI  • Admission to neonatal intensive care unit  • Fetal growth restriction  • Low Apgar scores at 1 and 5 minutes  • Low birthweight  • Neonatal and fetal deaths  • Placental abruption  • Pre-eclampsia  • Pre-term delivery (including emergency CS) | Outcomes not relating to the clinical effectiveness of management pathways for VP or VCI, although a broad approach was taken with regard to “relevance” |
| **Study design and publication type** | Peer-reviewed evidence derived from the following types of study:  • Systematic reviews and (network) meta-analyses  • RCTs  • Interventional non-RCTs  • Prospective cohort studies  • Retrospective cohort studies  • Case-control studies  • Cross-sectional studies  Case studies were tagged during abstract review but were not included for full-text review in the first instance. It was planned that case studies would be re-visited at a later stage of the review if any evidence gaps were identified for which they may have provided additional relevant evidence | Other study designs/publication types  Conference abstracts or other publication types that have not been peer-reviewed |
| **Language** | English language | Non-English language |
| **Date** | No date limit | - |

*Only the VCI part of the population and outcome eligibility criteria were applied during the update of the review. CS: caesarean section; RCT: randomised controlled trial; VCI: velamentous cord insertion; VP: vasa praevia.

Supplementary Table 5 – Studies excluded at full text review stage during the update.

| Reference | Reason excluded |
| --- | --- |
| Atallah A, Bolze PA, Buenerd A, et al. Macroscopic description of placental vascular anastomoses after dye injection for the comprehension of monochorionic pregnancy complications. Gynecologie Obstetrique Fertilite et Senologie 2017;45:269-275. | Full text in French |
| Curtin WM, Hill JM, Millington KA, et al. Accuracy of fetal anatomy survey in the diagnosis of velamentous cord insertion: A case-control study. International Journal of Women's Health 2019;11:169-176. | Unclear if transabdominal or transvaginal ultrasonography used |
| de los Reyes S, Henderson J, Eke AC. A systematic review and meta-analysis of velamentous cord insertion among singleton pregnancies and the risk of preterm delivery. International Journal of Gynecology and Obstetrics 2018;142:9-14. | SLR including studies pre-2006 |
| Ismail KI, Hannigan A, O'Donoghue K, et al. Abnormal placental cord insertion and adverse pregnancy outcomes: A systematic review and meta-analysis. Systematic Reviews 2017;6 (1) (no pagination). | SLR including studies pre-2006 |
| Sullivan EA, Javid N, Duncombe G, et al. Vasa Previa Diagnosis, Clinical Practice, and Outcomes in Australia. Obstetrics and Gynecology 2017;130:591-598. | Full text not freely unavailable |
| Takita H, Hasegawa J, Nakamura M, et al. Causes of intrauterine fetal death are changing in recent years. Journal of Perinatal Medicine 2018;46:97-101. | Full text not freely unavailable |
| Wan Masliza WD, Bajuri MY, Hassan MR, et al. Sonographically abnormal placenta: an association with an increased risk poor pregnancy outcomes. La Clinica terapeutica 2017;168:e283-e289. | No relevant results reported for VCI |

Supplementary Table 6 – Summary of publications with relevant VP data, and the question(s) they were used to address.

| Study | Study design | Country | Years of study | Criterion 1 – Epidemiology | Criterion 4 – Screening | Criteria 9 and 10 – Management pathways |
| --- | --- | --- | --- | --- | --- | --- |
| Ruiter 2016 (1) | SLR | NA | NA | Epidemiology | - | - |
| Baulies 2007 (2) | Retrospective | Spain | 2000 to 2005 | Epidemiology, adverse outcomes | - | [b] |
| Baumfeld 2016 (3), Evron 2015 (4), Rosenberg 2011 (5) | Retrospective | Israel | 1988 to 2012 | Epidemiology, adverse outcomes | - | - |
|  |  |  |  |  |  |  |
| Catanzarite 2001 (8) | Prospective | USA | 1991 to 1998 | Epidemiology [a] | Test accuracy | [b] |
| Donegan 2014 (9) | Retrospective | UK | 2010 to 2012 | Epidemiology | - | - |
| Francois 2003 (10) | Case-control | USA | 1991 to 2001 | Epidemiology | - | - |
| Hasegawa 2015 (11), Hasegawa 2010 (12) | Retrospective | Japan | 2005 to 2013 | Epidemiology, adverse outcomes | - | [c] |
| Hasegawa 2006 (13) | Prospective | Japan | 2003 to 2005 | Epidemiology | - | - |
|  |  |  |  |  |  |  |
| Huerta-Enochian 2001 (15) | Retrospective | USA | 1995 to 2000 | Epidemiology | - | - |
|  |  |  |  |  |  |  |
| Kapoor 2014 (17) | Retrospective | Australia | 2009 to 2011 | Epidemiology | - | - |
| Nomiyama 1998 (18) | Prospective | Japan | 1993 to 1996 | Epidemiology [a] | Test accuracy | - |
| Oyelese 2004 (19) | Retrospective | UK, USA, Israel | 1991 to 2003 | Epidemiology, adverse outcomes | - | [c] |
|  |  |  |  |  |  |  |
| Robinson 2012 (21) | Prospective | Australia | 2007 to 2008 | Epidemiology | - | - |
| Schachter 2002 (22) | Retrospective | Israel | 1987 to 2001 | Epidemiology, adverse outcomes | - | - |
|  |  |  |  |  |  |  |
| Suzuki 2015 (24), Suzuki 2010 (25), Suzuki 2008 (26) | Retrospective | Japan | 2000 to 2011 | Epidemiology, adverse outcomes | - | - |
| Swank 2016 (27) | Retrospective | USA | 2000 to 2012 | Epidemiology, natural history, adverse outcomes | - | - |

[a] Studies were completed before 2000 so epidemiology data have not been extracted, but other relevant data from the study have been extracted [b] Management pathways reported descriptively but not analytically; [c] Studies of interest, but not eligible for inclusion in this review.

Supplementary Table 7 – Summary of quality assessments of epidemiological and prognostic studies for VCI

| Study reference | Baumfeld 2016(3) | Bronsteen 2013 (6)  Lee 2000 (7) | Brouilliet 2014(28) | Bukowski 2017(29) | Chu 2013(30) | Cirstoiu 2016(31) | Costa-Castro 2013(32), Lopriore 2007(33) | Couck 2018(34) | Lopriore 2012(35) | Costa-Castro 2016(36) | De Paepe 2010a(37)  De Paepe 2010b(38) | De Paepe 2011(39) | Ebbing 2015(40), Ebbing 2013(41), Ebbing 2017(42) | Hack 2008(43) | Hack 2009(44) | Hasegawa 2009a(45), Hasegawa 2009b(46) |
| --- | --- | --- | --- | --- | --- | --- | --- | --- | --- | --- | --- | --- | --- | --- | --- | --- |
| PATIENT SELECTION |  |  |  |  |  |  |  |  |  |  |  |  |  |  |  |  |
| Was the sample representative of the target population? | N | Y | N | N | N | U | N | N | N | N | N | N | Y | N | N | N |
| Were study participants recruited in an appropriate way? | Y | Y | Y | Y | U | Y | Y | Y | Y | Y | Y | Y | Y | Y | Y | Y |
| Was the sample size adequate? | NA | Y | Y | Y | NA | Y | Y | Y | Y | Y | Y | Y | Y | Y | Y | N |
| Were the study subjects and the setting described in detail? | N | N | Y | N | N | N | N | Y | N | N | N | N | Y | N | N | N |
| STUDY ANALYSIS |  |  |  |  |  |  |  |  |  |  |  |  |  |  |  |  |
| Was the data analysis conducted with sufficient coverage of the identified sample? | Y | Y | Y | N | Y | U | Y | Y | Y | Y | Y | N | Y | Y | N | N |
| Were objective, standard criteria used for the measurement of the condition? | U | U | Y | Y | Y | Y | U | Y | Y | Y | Y | Y | U | Y | Y | N |
| Was the condition measured reliably? | U | Y | Y | N | Y | U | U | N | Y | Y | Y | Y | Y | Y | Y | Y |
| Was there appropriate statistical analysis? | Y | Y | NA | Y | Y | NA | Y | NA | Y | Y | Y | Y | Y | Y | Y | Y |
| Are all important confounding factors/ subgroups/differences identified and accounted for? | N | N | N | U | N | U | N | N | N | N | N | N | N | N | N | N |
| Were subpopulations identified using objective criteria? | U | NA | NA | NA | NA | NA | U | NA | NA | NA | NA | NA | Y | NA | NA | NA |
| PROGNOSTIC STUDIES QA |  |  |  |  |  |  |  |  |  |  |  |  |  |  |  |  |
| Was the defined representative sample of patients assembled at a common (usually early) point in the course of their disease)? | Y | N | Y | U | Y | U | Y | Y | Y | Y | Y | Y | Y | Y | Y | Y |
| Was patient follow-up sufficiently long and complete? | Y | Y | Y | Y | Y | Y | Y | Y | Y | Y | NA | NA | Y | Y | NA | Y |
| Were outcome criteria either objective or applied in a ‘blind’ fashion? | U | Y | Y | Y | Y | Y | Y | Y | NA | Y | U | Y | U | Y | Y | U |
| If subgroups with different prognoses are identified, did adjustment for important prognostic factors take place? | N | N | NA | Y | N | N | Y | NA | NA | Y | NA | NA | Y | U | N | Y |

| **Study reference** | **Hasegawa 2011(47)** | **Heller 2014 (14)** | **Ismail 2017(48)** | **Kalafat 2018(49)** | **Kanda 2011 (16)** | **Kent 2011(50)** | **Lepais 2014(51)** | **McNamara 2014(52)** | **Melcer 2017(53)** | **Pinar 2014(54)** | **Raisanen 2012(55)** | **Rebarber 2014 (20)** | **Smorgick 2010 (23)** | **Suzuki 2015(24)** | **Swank 2016 (27)** | **Walker 2012(56)** | **Waszak 2016(57)** | **Yanaihara 2018(58)** | **Yerlikaya 2016(59)** |
| --- | --- | --- | --- | --- | --- | --- | --- | --- | --- | --- | --- | --- | --- | --- | --- | --- | --- | --- | --- |
| PATIENT SELECTION |  |  |  |  |  |  |  |  |  |  |  |  |  |  |  |  |  |  |  |
| Was the sample representative of the target population? | Y | N | Y | N | Y | N | N | N | N | Y | N | U | Y | N | Y | N | N | N | N |
| Were study participants recruited in an appropriate way? | Y | Y | Y | Y | Y | Y | Y | Y | Y | Y | Y | Y | Y | Y | Y | Y | U | U | Y |
| Was the sample size adequate? | Y | N | Y | Y | Y | Y | N | Y | Y | Y | Y | Y | Y | Y | Y | Y | N | Y | Y |
| Were the study subjects and the setting described in detail? | N | N | Y | Y | N | N | N | N | Y | N | Y | U | N | Y | Y | N | N | N | Y |
| STUDY ANALYSIS |  |  |  |  |  |  |  |  |  |  |  |  |  |  |  |  |  |  |  |
| Was the data analysis conducted with sufficient coverage of the identified sample? | Y | U | U | Y | Y | Y | Y | Y | Y | N | Y | Y | Y | Y | Y | Y | U | U | Y |
| Were objective, standard criteria used for the measurement of the condition? | Y | U | Y | Y | U | Y | U | U | Y | U | Y | Y | Y | U | Y | U | Y | U | N |
| Was the condition measured reliably? | Y | U | Y | U | U | Y | N | U | Y | Y | Y | Y | U | N | Y | Y | U | U | U |
| Was there appropriate statistical analysis? | Y | NA | Y | NA | NA | Y | Y | Y | NA | U | Y | Y | Y | Y | Y | Y | Y | Y | Y |
| Are all important confounding factors/ subgroups/differences identified and accounted for? | N | N | U | Y | N | N | N | N | U | N | Y | NA | Y | Y | Y | N | U | Y | N |
| Were subpopulations identified using objective criteria? | NA | NA | NA | NA | NA | NA | NA | NA | NA | NA | Y | Y | U | U | Y | NA | NA | U | NA |
| QA OF PROGNOSTIC STUDIES |  |  |  |  |  |  |  |  |  |  |  |  |  |  |  |  |  |  |  |
| Was the defined representative sample of patients assembled at a common (usually early) point in the course of their disease)? | Y | Y | Y | Y | N | Y | Y | Y | Y | Y | Y | N | U | Y | N | Y | U | Y | N |
| Was patient follow-up sufficiently long and complete? | Y | Y | Y | Y | Y | Y | Y | Y | Y | Y | Y | Y | Y | Y | Y | Y | Y | Y | Y |
| Were outcome criteria either objective or applied in a ‘blind’ fashion? | NA | NA | Y | Y | U | Y | U | NA | U | NA | U | U | U | U | Y | NA | U | U | U |
| If subgroups with different prognoses are identified, did adjustment for important prognostic factors take place? | NA | NA | N | NA | N | N | Y | NA | NA | NA | Y | NA | N | Y | N | NA | N | NA | N |

REFERENCES

1. Ruiter L, Kok N, Limpens J, Derks JB, de Graaf IM, Mol B, et al. Incidence of and risk indicators for vasa praevia: a systematic review. BJOG: An International Journal of Obstetrics & Gynaecology. 2016;123(8):1278-87.

2. Baulies S, Maiz N, Munoz A, Torrents M, Echevarria M, Serra B. Prenatal ultrasound diagnosis of vasa praevia and analysis of risk factors. Prenatal Diagnosis. 2007;27(7):595-9.

3. Baumfeld Y, Gutvirtz G, Shoham I, Sheiner E. Fetal heart rate patterns of pregnancies with vasa previa and velamentous cord insertion. Archives of Gynecology and Obstetrics. 2016;293(2):361-7.

4. Evron E, Sheiner E, Friger M, Sergienko R, Harlev A. Vanishing twin syndrome: Is it associated with adverse perinatal outcome? Fertility and Sterility. 2015;103(5):1209-14.

5. Rosenberg T, Pariente G, Sergienko R, Wiznitzer A, Sheiner E. Critical analysis of risk factors and outcome of placenta previa. Archives of Gynecology and Obstetrics. 2011;284(1):47-51.

6. Bronsteen R, Whitten A, Balasubramanian M, Lee W, Lorenz R, Redman M, et al. Vasa previa: clinical presentations, outcomes, and implications for management. Obstetrics and gynecology. 2013;122(2 Pt 1):352-7.

7. Lee W, Lee VL, Kirk JS, Sloan CT, Smith RS, Comstock CH. Vasa previa: Prenatal diagnosis, natural evolution, and clinical outcome. Obstetrics and Gynecology. 2000;95(4):572-6.

8. Catanzarite V, Maida C, Thomas W, Mendoza A, Stanco L, Piacquadio KM. Prenatal sonographic diagnosis of vasa previa: Ultrasound findings and obstetric outcome in ten cases. Ultrasound in Obstetrics and Gynecology. 2001;18(2):109-15.

9. Donegan K, King B, Bryan P. Safety of pertussis vaccination in pregnant women in UK: observational study. Bmj. 2014;349:g4219.

10. Francois K, Mayer S, Harris C, Perlow JH. Association of Vasa Previa at Delivery with a History of Second-Trimester Placenta Previa. Journal of Reproductive Medicine for the Obstetrician and Gynecologist. 2003;48(10):771-4.

11. Hasegawa J, Arakaki T, Ichizuka K, Sekizawa A. Management of vasa previa during pregnancy. Journal of Perinatal Medicine. 2015;43(6):783-4.

12. Hasegawa J, Farina A, Nakamura M, Matsuoka R, Ichizuka K, Sekizawa A, et al. Analysis of the ultrasonographic findings predictive of vasa previa. Prenatal Diagnosis. 2010;30(12-13):1121-5.

13. Hasegawa J, Matsuoka R, Ichizuka K, Otsuki K, Sekizawa A, Farina A, et al. Cord insertion into the lower third of the uterus in the first trimester is associated with placental and umbilical cord abnormalities. Ultrasound in Obstetrics and Gynecology. 2006;28(2):183-6.

14. Heller HT, Mullen KM, Gordon RW, Reiss RE, Benson CB. Outcomes of pregnancies with a low-lying placenta diagnosed on second-trimester sonography. Journal of ultrasound in medicine : official journal of the American Institute of Ultrasound in Medicine. 2014;33(4):691-6.

15. Huerta-Enochian G, Katz V, Erfurth S. The association of abnormal alpha-fetoprotein and adverse pregnancy outcome: Does increased fetal surveillance affect pregnancy outcome? American Journal of Obstetrics and Gynecology. 2001;184(7):1549-55.

16. Kanda E, Matsuda Y, Kamitomo M, Maeda T, Mihara K, Hatae M. Prenatal diagnosis and management of vasa previa: a 6-year review. The journal of obstetrics and gynaecology research. 2011;37(10):1391-6.

17. Kapoor S, Thomas JT, Petersen SG, Gardener GJ. Is the third trimester repeat ultrasound scan for placental localisation needed if the placenta is low lying but clear of the os at the mid-trimester morphology scan? Australian and New Zealand Journal of Obstetrics and Gynaecology. 2014;54(5):428-32.

18. Nomiyama M, Toyota V, Kawano H. Antenatal diagnosis of velamentous umbilical cord insertion and vasa previa with color Doppler imaging. Ultrasound in Obstetrics and Gynecology. 1998;12(6):426-9.

19. Oyelese Y, Catanzarite V, Prefumo F, Lashley S, Schachter M, Tovbin Y, et al. Vasa previa: The impact of prenatal diagnosis on outcomes. Obstetrics and Gynecology. 2004;103(5 I):937-42.

20. Rebarber A, Dolin C, Fox NS, Klauser CK, Saltzman DH, Roman AS. Natural history of vasa previa across gestation using a screening protocol. Journal of ultrasound in medicine : official journal of the American Institute of Ultrasound in Medicine. 2014;33(1):141-7.

21. Robinson AJ, Muller PR, Allan R, Ross R, Baghurst PA, Keirse MJNC. Precise mid-trimester placenta localisation: Does it predict adverse outcomes? Australian and New Zealand Journal of Obstetrics and Gynaecology. 2012;52(2):156-60.

22. Schachter M, Tovbin Y, Arieli S, Friedler S, Ron-El R, Sherman D. In vitro fertilization is a risk factor for vasa previa. Fertility and Sterility. 2002;78(3):642-3.

23. Smorgick N, Tovbin Y, Ushakov F, Vaknin Z, Barzilay B, Herman A, et al. Is neonatal risk from vasa previa preventable? The 20-year experience from a single medical center. Journal of clinical ultrasound : JCU. 2010;38(3):118-22.

24. Suzuki S, Kato M. Clinical Significance of Pregnancies Complicated by Velamentous Umbilical Cord Insertion Associated With Other Umbilical Cord/Placental Abnormalities. J Clin Med Res. 2015;7(11):853-6.

25. Suzuki S, Igarashi M, Inde Y, Miyake H. Abnormally shaped placentae in twin pregnancy. Archives of Gynecology and Obstetrics. 2010;281(1):65-9.

26. Suzuki S, Igarashi M. Clinical significance of pregnancies with succenturiate lobes of placenta. Archives of Gynecology and Obstetrics. 2008;277(4):299-301.

27. Swank ML, Garite TJ, Maurel K, Das A, Perlow JH, Combs CA, et al. Vasa previa: diagnosis and management. Am J Obstet Gynecol. 2016;2:2.

28. Brouillet S, Dufour A, #xEF, Prot F, Feige J-J, Equy V, et al. Influence of the Umbilical Cord Insertion Site on the Optimal Individual Birth Weight Achievement. BioMed Research International. 2014;2014:8.

29. Bukowski R, Hansen NI, Pinar H, Willinger M, Reddy UM, Parker CB, et al. Altered fetal growth, placental abnormalities, and stillbirth. PLoS ONE [Electronic Resource]. 2017;12(8):e0182874.

30. Chu S, Mao Q, Shapiro S, Luks SL, De Paepe ME. Correlation between cord insertion type and chorionic villus vascularization of the co-twin in diamniotic-monochorionic twin pregnancies. Early Human Development. 2013;89(4):243-7.

31. Cirstoiu MM, Turcan N, BrAtil AE, Munteanu O, Bodean O, Voicu D, et al. Velamentous cord insertion - An important obstetrical risk factor. Ginecoeu. 2016;12(3):129-34.

32. Costa-Castro T, De Villiers S, Montenegro N, Severo M, Oepkes D, Matias A, et al. Velamentous cord insertion in monochorionic twins with or without twin-twin transfusion syndrome: Does it matter? Placenta. 2013;34(11):1053-8.

33. Lopriore E, Sueters M, Middeldorp JM, Oepkes D, Walther FJ, Vandenbussche FPHA. Velamentous cord insertion and unequal placental territories in monochorionic twins with and without twin-to-twin-transfusion syndrome. American Journal of Obstetrics and Gynecology. 2007;196(2):159.e1-.e5.

34. Couck I, Mourad Tawfic N, Deprest J, De Catte L, Devlieger R, Lewi L. Does site of cord insertion increase risk of adverse outcome, twin-to-twin transfusion syndrome and discordant growth in monochorionic twin pregnancy? Ultrasound in obstetrics & gynecology : the official journal of the International Society of Ultrasound in Obstetrics and Gynecology. 2018;52(3):385-9.

35. Lopriore E, Pasman SA, Klumper FJ, Middeldorp JM, Walther FJ, Oepkes D. Placental characteristics in growth-discordant monochorionic twins: A matched case-control study. Placenta. 2012;33(3):171-4.

36. Costa-Castro T, Zhao DP, Lipa M, Haak MC, Oepkes D, Severo M, et al. Velamentous cord insertion in dichorionic and monochorionic twin pregnancies - Does it make a difference? Placenta. 2016;42:87-92.

37. De Paepe ME, Shapiro S, Greco D, Luks VL, Abellar RG, Luks CH, et al. Placental markers of twin-to-twin transfusion syndrome in diamniotic-monochorionic twins: A morphometric analysis of deep artery-to-vein anastomoses. Placenta. 2010a;31(4):269-76.

38. De Paepe ME, Shapiro S, Young L, Luks FI. Placental characteristics of selective birth weight discordance in diamniotic-monochorionic twin gestations. Placenta. 2010b;31(5):380-6.

39. De Paepe ME, Shapiro S, Hanley LC, Chu S, Luks FI. Correlation between cord insertion type and superficial choriovasculature in diamniotic-monochorionic twin placentas. Placenta. 2011;32(11):901-5.

40. Ebbing C, Kiserud T, Johnsen SL, Albrechtsen S, Rasmussen S. Third stage of labor risks in velamentous and marginal cord insertion: A population-based study. Acta Obstetricia et Gynecologica Scandinavica. 2015;94(8):878-83.

41. Ebbing C, Kiserud T, Johnsen SL, Albrechtsen S, Rasmussen S. Prevalence, risk factors and outcomes of velamentous and marginal cord insertions: a population-based study of 634,741 pregnancies. PLoS ONE. 2013;8(7):e70380.

42. Ebbing C, Johnsen SL, Albrechtsen S, Sunde ID, Vekseth C, Rasmussen S. Velamentous or marginal cord insertion and the risk of spontaneous preterm birth, prelabor rupture of the membranes, and anomalous cord length, a population-based study. Acta Obstetricia et Gynecologica Scandinavica. 2017;96(1):78-85.

43. Hack KEA, Nikkels PGJ, Koopman-Esseboom C, Derks JB, Elias SG, van Gemert MJC, et al. Placental Characteristics of Monochorionic Diamniotic Twin Pregnancies in Relation to Perinatal Outcome. Placenta. 2008;29(11):976-81.

44. Hack KEA, van Gemert MJC, Lopriore E, Schaap AHP, Eggink AJ, Elias SG, et al. Placental Characteristics of Monoamniotic Twin Pregnancies in Relation to Perinatal Outcome. Placenta. 2009;30(1):62-5.

45. Hasegawa J, Matsuoka R, Ichizuka K, Kotani M, Nakamura M, Mikoshiba T, et al. Atypical variable deceleration in the first stage of labor is a characteristic fetal heart-rate pattern for velamentous cord insertion and hypercoiled cord. Journal of Obstetrics and Gynaecology Research. 2009a;35(1):35-9.

46. Hasegawa J, Matsuoka R, Ichizuka K, Nakamura M, Sekizawa A, Okai T. Do fetal heart rate deceleration patterns during labor differ between various umbilical cord abnormalities? Journal of Perinatal Medicine. 2009b;37(3):276-80.

47. Hasegawa J, Nakamura M, Sekizawa A, Matsuoka R, Ichizuka K, Okai T. Prediction of risk for vasa previa at 9-13 weeks' gestation. The journal of obstetrics and gynaecology research. 2011;37(10):1346-51.

48. Ismail KI, Hannigan A, Kelehan P, O'Donoghue K, Cotter A. Abnormal Placental Cord Insertion and Adverse Pregnancy Outcomes: Results from a Prospective Cohort Study. American Journal of Perinatology. 2017;34(11):1152-9.

49. Kalafat E, Thilaganathan B, Papageorghiou A, Bhide A, Khalil A. Significance of placental cord insertion site in twin pregnancy. Ultrasound in obstetrics & gynecology : the official journal of the International Society of Ultrasound in Obstetrics and Gynecology. 2018;52(3):378-84.

50. Kent EM, Breathnach FM, Gillan JE, McAuliffe FM, Geary MP, Daly S, et al. Placental cord insertion and birthweight discordance in twin pregnancies: Results of the national prospective ESPRiT Study. American Journal of Obstetrics and Gynecology. 2011;205(4):376.e1-.e7.

51. Lepais L, Gaillot-Durand L, Boutitie F, Lebreton F, Buffin R, Huissoud C, et al. Fetal thrombotic vasculopathy is associated with thromboembolic events and adverse perinatal outcome but not with neurologic complications: A retrospective cohort study of 54 cases with a 3-year follow-up of children. Placenta. 2014;35(8):611-7.

52. McNamara H, Hutcheon JA, Platt RW, Benjamin A, Kramer MS. Risk factors for high and low placental weight. Paediatric and Perinatal Epidemiology. 2014;28(2):97-105.

53. Melcer Y, Maymon R, Pekar-Zlotin M, Levinsohn-Tavor O, Tovbin J, Jauniaux E. Evaluation of the impact of vasa previa on feto-placental hormonal synthesis and fetal growth. European Journal of Obstetrics Gynecology and Reproductive Biology. 2017;215:193-6.

54. Pinar H, Goldenberg RL, Koch MA, Heim-Hall J, Hawkins HK, Shehata B, et al. Placental findings in singleton stillbirths. Obstetrics and gynecology. 2014;123(2 Pt 1):325-36.

55. Raisanen S, Georgiadis L, Harju M, Keski-Nisula L, Heinonen S. Risk factors and adverse pregnancy outcomes among births affected by velamentous umbilical cord insertion: A retrospective population-based register study. European Journal of Obstetrics Gynecology and Reproductive Biology. 2012;165(2):231-4.

56. Walker MG, Fitzgerald B, Keating S, Ray JG, Windrim R, Kingdom JCP. Sex-specific basis of severe placental dysfunction leading to extreme preterm delivery. Placenta. 2012;33(7):568-71.

57. Waszak M, Cieslik K, Pietryga M, Lewandowski J, Chuchracki M, Nowak-Markwitz E, et al. Effect of morphological and functional changes in the secundines on biometric parameters of newborns from dichorionic twin pregnancies. Ginekologia polska. 2016;87(11):755-62.

58. Yanaihara A, Hatakeyama S, Ohgi S, Motomura K, Taniguchi R, Hirano A, et al. Difference in the size of the placenta and umbilical cord between women with natural pregnancy and those with IVF pregnancy. Journal of Assisted Reproduction and Genetics. 2018;35(3):431-4.

59. Yerlikaya G, Pils S, Springer S, Chalubinski K, Ott J. Velamentous cord insertion as a risk factor for obstetric outcome: a retrospective case-control study. Archives of Gynecology and Obstetrics. 2016;293(5):975-81.
